# Ribosome Incorporation into Somatic Cells Promotes Lineage Transdifferentiation towards Multipotency

**DOI:** 10.1038/s41598-018-20057-1

**Published:** 2018-01-26

**Authors:** Naofumi Ito, Kaoru Katoh, Hiroko Kushige, Yutaka Saito, Terumasa Umemoto, Yu Matsuzaki, Hiroshi Kiyonari, Daiki Kobayashi, Minami Soga, Takumi Era, Norie Araki, Yasuhide Furuta, Toshio Suda, Yasuyuki Kida, Kunimasa Ohta

**Affiliations:** 1 Department of Developmental Neurobiology, Graduate School of Life Sciences, Kumamoto University, 1-1-1 Honjo, Chuo-ku, Kumamoto 860-8556 Japan; 2 Program for Leading Graduate Schools “HIGO Program”, Kumamoto University, 1-1-1 Honjo, Chuo-ku, Kumamoto 860-8556 Japan; 3 Biomedical Research Institute, National Institute of Advanced Industrial Science and Technology, Central 6, 1-1-1 Higashi, Tsukuba, Ibaraki 305-8566 Japan; 4 Biotechnology Research Institute for Drug Discovery, National Institute of Advanced Industrial Science and Technology (AIST), 1-1-1 Higashi, Tsukuba, Ibaraki 305-8565 Japan; 5 Biotechnology Research Institute for Drug Discovery, National Institute of Advanced Industrial Science and Technology (AIST), 2-4-7 Aomi, Koto-ku, Tokyo 135-0064 Japan; 6 International Research Center for Medical Science, Kumamoto University, 2-2-1 Honjo, Chuo-ku, Kumamoto City, 860-0811 Japan; 7 Animal Resource Development Unit and Genetic Engineering Team, RIKEN Center for Life Science Technologies, 2-2-3 Minatojima-minamimachi, Chuo-ku, Kobe, Hyogo 650-0047 Japan; 8 Department of Tumor Genetics and Biology, Graduate School of Life Sciences, Kumamoto University, 1-1-1 Honjo, Chuo-ku, Kumamoto 860-8556 Japan; 9 Department of Cell Modulation, Institute of Molecular Embryology and Genetics, Kumamoto University, 2-2-1 Honjo, Chuo-ku, Kumamoto 860-0811 Japan; 10 Cancer Science Institute of Singapore, National University of Singapore, Centre for Translational Medicine, 14 Medical Drive, 117599 Singapore, Singapore; 11 International Research Core for Stem Cell-based Developmental Medicine, Kumamoto University, 1-1-1 Honjo, Chuo-ku, Kumamoto 860-8556 Japan; 12 Japan Agency for Medical Research and Development (AMED), Tokyo, 100-0004 Japan

## Abstract

Recently, we reported that bacterial incorporation induces cellular transdifferentiation of human fibroblasts. However, the bacterium-intrinsic cellular- transdifferentiation factor remained unknown. Here, we found that cellular transdifferentiation is caused by ribosomes. Ribosomes, isolated from both prokaryotic and eukaryotic cells, induce the formation of embryoid body-like cell clusters. Numerous ribosomes are incorporated into both the cytoplasm and nucleus through trypsin-activated endocytosis, which leads to cell-cluster formation. Although ribosome-induced cell clusters (RICs) express several stemness markers and differentiate into derivatives of all three germ layers in heterogeneous cell populations, RICs fail to proliferate, alter the methylation states of pluripotent genes, or contribute to teratoma or chimera formation. However, RICs express markers of epithelial–mesenchymal transition without altering the cell cycle, despite their proliferation obstruction. These findings demonstrate that incorporation of ribosomes into host cells induces cell transdifferentiation and alters cellular plasticity.

## Introduction

Terminally differentiated somatic cells are considered to be stable. However, pluripotency can be achieved by transplanting the nuclei of frog somatic cells into eggs^1^. Furthermore, induced pluripotent stem (iPS) cells can be generated by forced expression of specific transcription factors^2^. A recent study showed that pluripotent stem cells can also be generated from mouse somatic cells by using a cocktail of small-molecule compounds^3^.

Humans contact microbiota immediately after birth and interact broadly with microbiota throughout life, such as during disease^4^, nutrient absorption^5^, and immune system development^6^. The microbial community in the human intestine has been widely analyzed, and lactic acid bacteria are common bacteria among the intestinal microbiota^7^ closely associated with homeostasis and immunity in humans^8^. Previously, we demonstrated that lactic acid bacteria incorporation into human dermal fibroblasts (HDFs) altered cellular fate and could differentiate into cells of all three germ layers^9^. Cell fate has been reported to be affected by microbiota: leprosy bacilli were observed to expand their infection by hijacking cellular reprogramming^10^, and the gut microbiota controls the development of neural glia in the host intestine^11^. Bacteria have been shown to affect human cellular differentiation, but the developmental effect of bacteria remains unclear because the bacterium-intrinsic transforming factors that covert somatic cells into cells that can differentiate into the three germ layers have not been identified.

In general, ribosomes are thought to function as the translational machinery in all organisms, but were recently been reported to control tumorigenesis, immune signaling, and development^12^. The phenomenon of a single protein in ribosome having a plurality of functions in addition to its original function is known as ‘moonlighting’^13,14^. Here, we report the differentiation of ribosome-induced cell clusters (RICs), dedifferentiated from the somatic cells, into the derivatives of the three germ-layer cells. Ribosomes were incorporated into cells through trypsin-activated endocytosis and generated cell clusters that were similar to embryoid bodies. The RICs expressed pluripotency markers and differentiated into cells derived from all three germ lineages upon conditional cultivation, although the phenotypes of cell growth, epigenetic demethylation, and teratoma and chimera formation differed from those of pluripotent stem cells. Furthermore, we demonstrated that these RICs impeded cell proliferation, similar to the ribosomal stress known as the starvation response^15^. Our data reveal unanticipated developmental plasticity of somatic cells conferred by universally present intrinsic ribosomes and a previously unknown avenue for acquiring stemness through communication between cells and bacteria.

## Results

### Cellular transdifferentiation is induced by ribosomes

**Figure 1:**
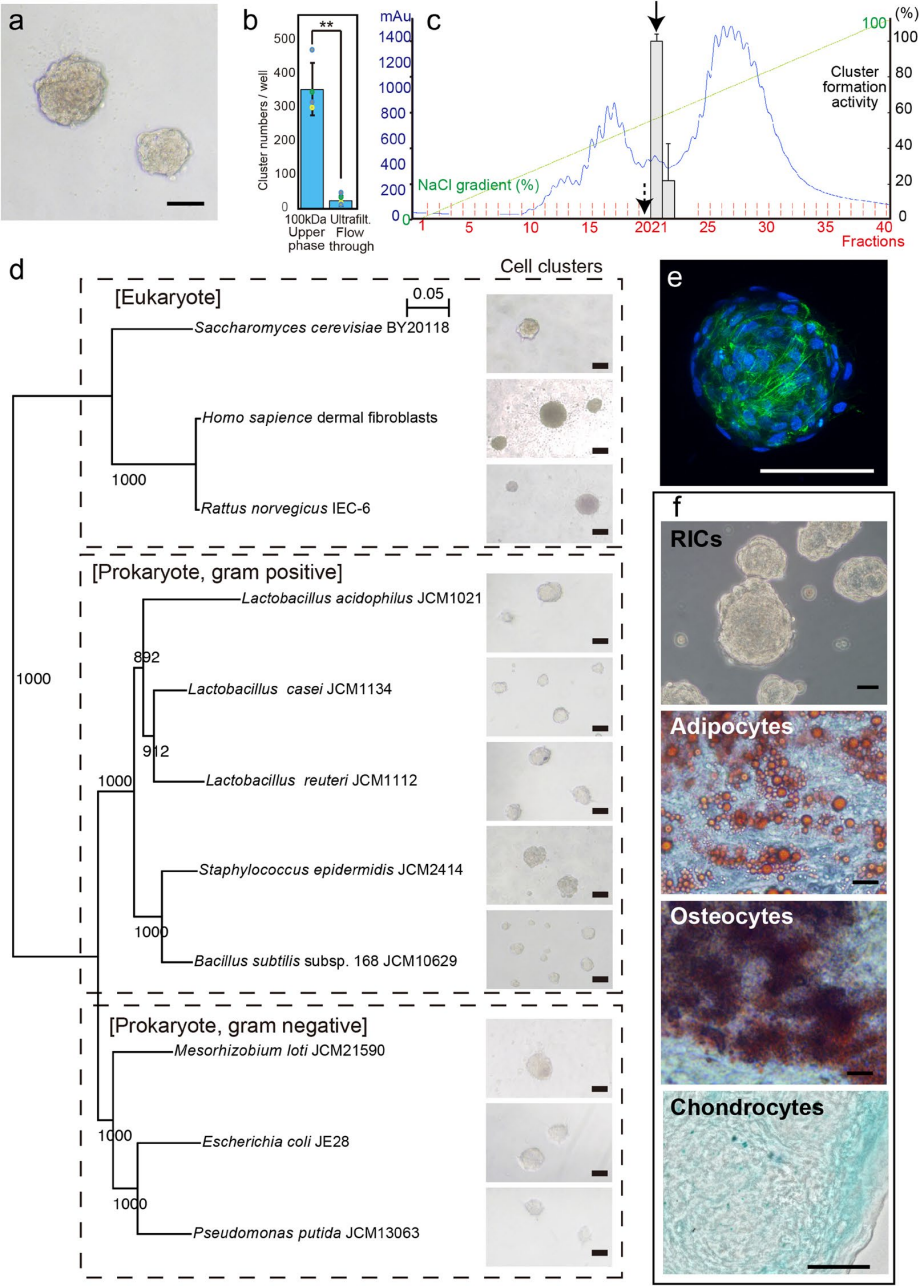
Formation and differentiation of ribosome-induced cell clusters (RICs) from HDFs. (**a**) Cell clusters induced by *Lactobacillus acidophilus* cell lysate. (**b**) Cluster formation of fractions of *L. acidophilus* lysate prepared by 100-kDa ultrafiltration. Upper panel shows cluster formation by the upper phase of filtration (>100 kDa MWCO) and lower panel shows cluster formation by the passed-through fraction (<100 kDa MWCO). Scores show the number of clusters. (**c**) Column-chromatographic separation of *L. acidophilus* lysate. Elution profile, shown in milli-absorbance units (mAU; absorbance at 280 nm), obtained with increasing NaCl concentrations. Arrow indicates the peak fraction showing the highest HDF cluster induction activity; dashed arrow indicates the preceding low-activity peak fraction. These 2 fractions were subjected to LC/MS/MS analysis (Table S1). (**d**) Cell clusters induced by ribosomes isolated from diverse organisms. Organisms and 16 S rRNA accession numbers are listed in Table S3. (**e**) Alexa Fluor 488 phalloidin staining of RICs (green). Nuclei were stained with Hoechst 33342 (blue). (See also Video S1.) (**f**) Cell clusters induced by commercially available *E. coli* ribosomes. RICs were transformed into adipocytes, osteocytes, and chondrocytes by cultivation under appropriate differentiation conditions. Bars = 100 µm (**a**) and (**e**). 50 µm (**f**).

We hypothesized that cell-cluster formation and lineage transdifferentiation activity were closely linked because embryonic stem cells, iPS cells, and bacterially reprogrammed cells clustered in the dedifferentiated state^16,17^. We prepared a *Lactobacillus acidophilus* cell lysate, mixed the lysate with trypsinized HDFs, and plated the cells on normal cell-culture plates, which resulted in the formation of specific cell clusters (Fig. 1a). Subsequently, we found that the >100-kDa fraction obtained from ultrafiltration of the lysate induced cell cluster formation (Fig. 1b). Next, we fractionated the *L. acidophilus* lysate by column chromatography (Fig. 1c) and analyzed the peak fraction showing the highest activity (arrow; Fraction 21 (F21)) and the preceding low-activity fraction (dashed arrow; F20) by comparative liquid chromatography/tandem mass spectrometry (LC/MS/MS). No molecules larger than 100 kDa were identified as single proteins among the 35 proteins specifically enriched and/or more highly enriched in F21 than in F20 (Table S1). Thus, we predicted that the transdifferentiation factor existed as a large complex and that the ribosome, which has a molecular mass of 2.7 MDa^18^, was the candidate transdifferentiation material; accordingly, we identified 8 ribosomal proteins. We could not find a protein that forms a protein complex exceeding 100 kDa in the list, as far as we know.

To verify that the ribosome functions as a cell-cluster-inducing factor and determine whether this phenomenon is caused only by ribosomes isolated from *L. acidophilus* (L-ribosomes), we purified L-ribosomes and several other types of ribosomes by conventional ultracentrifugation and then applied the L-ribosomes to HDFs as described above; all ribosomes induced substantial cell-cluster formation (Fig. 1d). Moreover, to demonstrate that cell-cluster formation was not caused by a contaminant derived from the centrifugal purification of ribosomes, we purified whole His6-tagged ribosomes (His-ribosomes) from genetically modified *Escherichia coli* JE28 cells^19^ by affinity chromatography. The purified His-ribosomes were applied to the HDFs as described above; this treatment also induced cell-cluster formation (Fig. 1d,e and Video S1). Furthermore, we found that cell clusters induced by ribosomes isolated from *L. acidophilus, Staphylococcus epidermis*, *Saccharomyces cerevisiae*, *Rattus norvegicus*, and *E. coli* JE28 were transformed into adipocytes when cultivated in differentiation media (Fig. S1a–f). It was reported that mesenchymal like stem cells and also few of HDFs possess differentiation activity to adipocyte, osteocyte and chondrocyte from mesenchymal like stem cells populations^20,21^. We demonstrated that HDF without ribosome treatment does not have differentiation activity to adipocytes, chondrocytes, cardiomyocytes and neurons under our culture conditions (Fig. S1g–m). Lastly, we tested commercially available ribosomes, supplied with *in vitro* translation kits, to confirm their effect on cellular transdifferentiation (Fig. 1f); these ribosomes also induced cell-cluster formation and the subsequent differentiation of the clustered cells into adipocytes, osteocytes, and chondrocytes. These results indicate that treatment of HDFs with ribosomes can induce several lineage-transformable cell clusters, and that this effect is independent of the phylogenetic relationship and structural variety of the ribosomes.

### Ribosomes are incorporated into the cytoplasm and nucleus through endocytosis

**Figure 2:**
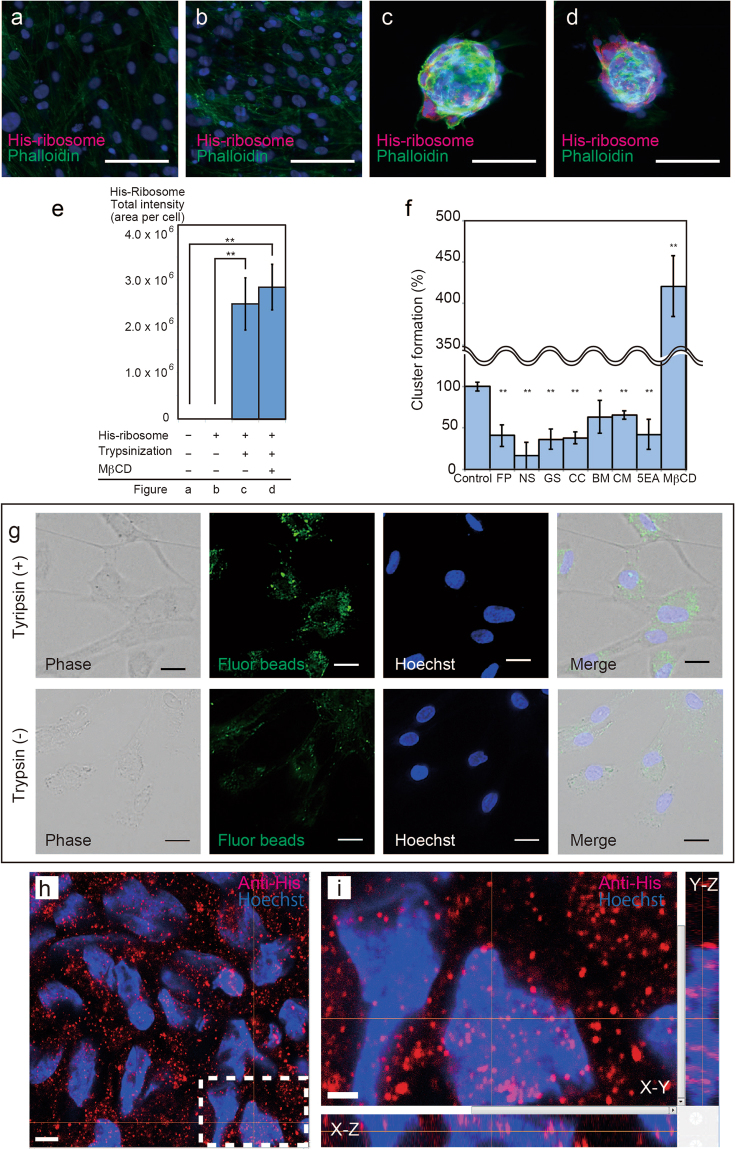
Ribosome incorporation and localization in HDFs. (**a**–**d**) Cytochemical analysis of RICs performed using an anti-His-tag antibody (magenta) and Alexa Fluor 488 phalloidin (green). (**a**) Normal HDFs. (**b**) Normal HDFs treated with His-ribosomes without trypsinization (negative control). (**c**) His-ribosome-incorporated cell clusters obtained with trypsinization. (**d**) His-ribosome-incorporated cell clusters obtained with trypsinization and methyl-β-cyclodextrin (MβCD) treatment. Nuclei were stained with Hoechst 33342 (blue). (**e**) Quantification of intracellular His-ribosomes (n = 4, **P < 0.01). Significant differences were not detected between A and B or C and D. (**f**) Summary of endocytosis inhibitor analysis (n ≥ 4, *P < 0.05, **P < 0.01, compared to control (i.e., no addition of inhibitors)). Detailed data are provided in Fig. S2. (**g**) Fluorescent bead incorporation in trypsinized (upper) and non-trypsinized HDFs (lower). (**h**) High-resolution confocal microscopy images of individual nuclei. His-ribosomes (red) and nuclei (blue) are shown. (**i**) Image focused on one anti-His signal in one nucleus, as indicated by the lines. See also Video S2. Bars = 100 µm (**a**–**d**), 20 µm (**g**), 5 µm (**h**), 2 µm (**i**).

We previously reported that cell-cluster formation did not occur in the absence of trypsin treatment^9^, and trypsin digestion was also reported to activate endocytosis in pancreatic acinar cells^22^; however, the effect of trypsinization on HDFs is unknown. We compared RIC formation conditions and visualized His-ribosomes. No signals were observed from normal HDFs (Fig. 2a). HDFs to which His-ribosomes were added without trypsinization also showed no His-ribosome signals (Fig. 2b). Only trypsinized HDFs supplemented with His-ribosomes formed RICs (Fig. 2c), and abundant ribosome incorporation was measured in these RICs (Fig. 2e). These data indicate that ribosome incorporation is activated by trypsinization and that incorporation of ribosomes is necessary for cell-cluster formation.

To determine how HDFs incorporate ribosomes, we tested the effects of the following endocytosis inhibitors: filipin (FP), nystatin (NS), genistein (GS), cytochalasin B (CC), bafilomycin A1 (BM), concanamycin A (CM), 5-(N-ethyl-N-isopropyl)-amiloride (5EA), chlorpromazine (CP), and methyl-β-cyclodextrin (MβCD) (Fig. 2f and Fig. S2). We found that FP, NS, GS, CC, BM, CM, and 5EA inhibited the formation of RICs, but we could not determine the effect of CP because it was highly cytotoxic. Unexpectedly, MβCD drastically enhanced ribosome incorporation and RIC formation (Fig. 2d–f and Fig. S2h). The reduced RICs formation by the endocytosis inhibitor treatments was not due to reduced cell viability because we observed that almost all inhibitors that significantly reduced numbers of RICs at the concentrations at which cell viability was not affected (Fig. 2f and Fig. S2). Next, to confirm the activation of endocytotic incorporation by trypsinization, we used microfluorobeads and found that these beads were potently incorporated into trypsinized cells (Fig. 2g). Finally, we observed RICs by confocal microscopy at the highest magnification available, and the obtained 3D image stacks clearly revealed that His-ribosomes were incorporated into the cytoplasmic and nuclear regions (Fig. 2h,i and Video S2). Thus, our results indicate that ribosomes are incorporated into the cytoplasmic and nuclear areas of HDFs through trypsin-activated endocytosis, which is critical for RIC formation.

### RICs express stemness-maintenance markers

**Figure 3:**
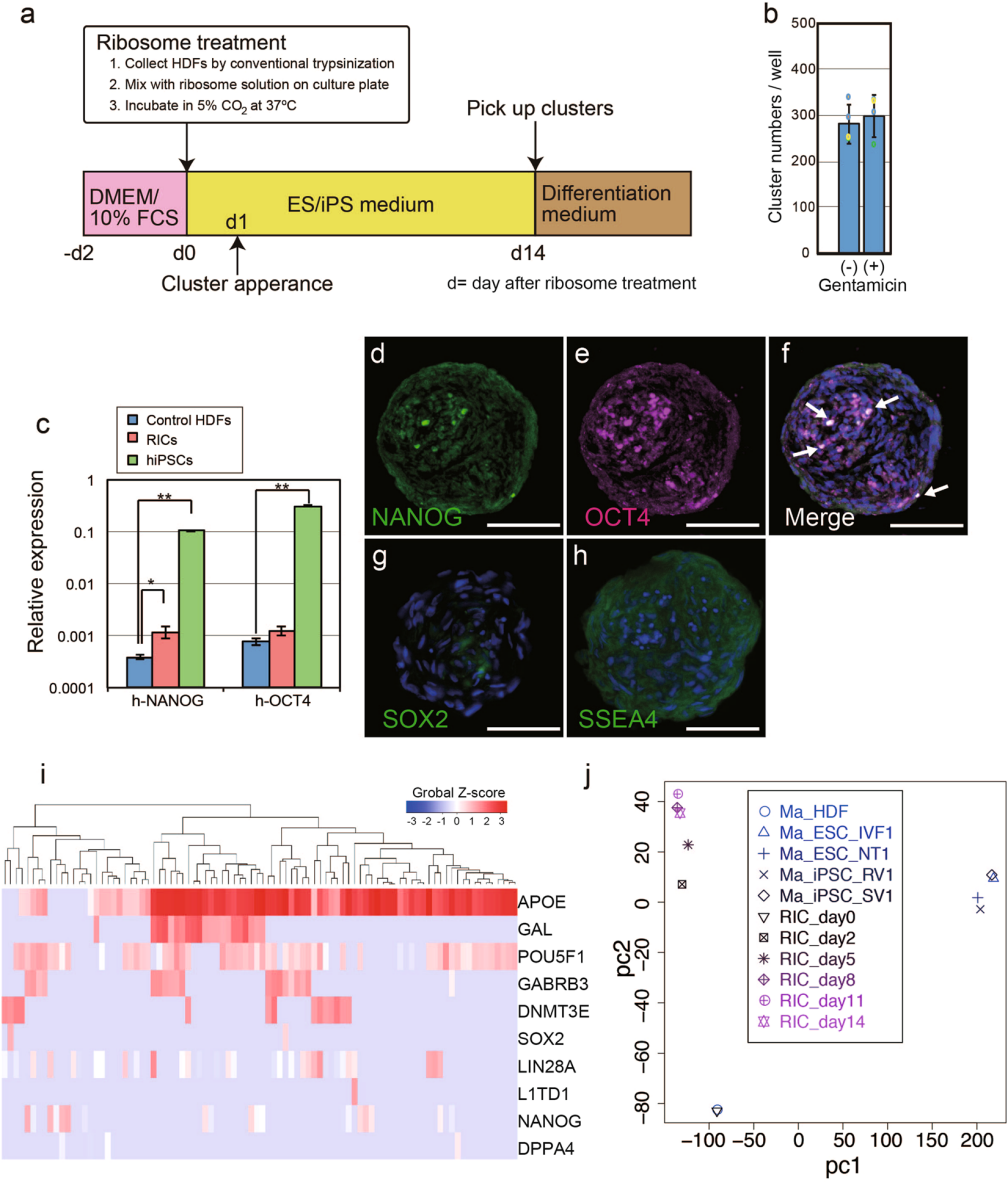
Stemness phenotype in RICs. (**a**) Schematic representation of RIC generation procedure. (**b**) Gentamicin treatment during cluster formation. (**c**) qPCR analysis of NANOG and OCT4 (n = 3, *P < 0.05). (**d**–**h**) Immunocytochemistry of RICs performed using antibodies against the stem cell maintenance markers (**d**) NANOG, (**e**) OCT4, (**g**) SOX2, and (**h**) SSEA4. (**f**) Merged image of images in (**d**) and (**e**). (**i**) Single-cell qPCR assay in RICs. (**j**) Principal component analysis between RICs and conventional pluripotent stem cells^27^. Bars = 50 µm (**d**–**h**).

The experimental protocol for generating RICs is summarized in Fig. 3a. HDFs were trypsinized and mixed with ribosomes on culture plates before they attached to the bottom of the plates. First, we examined the effect of translation by bacterial ribosomes in host HDFs for RIC formation (Fig. 3b). The antibiotic gentamicin, which inhibits protein translation by binding to a small subunit of bacterial ribosomes and not to eukaryotic ribosomes, did not affect cluster formation efficiency. Next, we investigated the potency of stemness against pluripotent stem cell classifications^23^. Quantitative PCR (qPCR) analysis revealed that NANOG, a stem cell gateway factor^24^, was expressed in RICs (Fig. 3c), and immunocytochemistry revealed that NANOG and the pluripotent transcription factors POU5F1 (OCT4), SOX2, and SSEA4 were specifically expressed in RICs in a mosaic pattern (Fig. 3d–h, Figs S3, S4). The results of transcriptome analysis showed that the expression involved in somatic stem-cell maintenance was altered in L-ribosome-induced cell clusters compared to the expression in non-induced HDFs (Fig. S5). Although NANOG was expressed in RICs, demethylation in the NANOG promoter was not observed (Fig. S6). Abnormal karyotype, that cause proliferation and pluripotency failure, is observed in about 12% of pluripotent stem cells^25^. We analyzed copy number variation to investigate the karyotype (Fig. S7), which indicated that a polymorphism was present in the 572-kb-long region of chromosome 17q21.31, although no other variants and chromosomal replications were detected.

We also investigated the expression diversity of His-ribosome-induced RICs in a single-cell qPCR assay with 10 stem cell primer pairs^26^ (Fig. 3i). The 99 cell expression patterns showed that stemness was heterogenous in the cell cluster. We also performed single cell transcription for the pluripotency markers and compared RICs population and normal HDFs (Fig. S8). Expression level diversity of the RICs population were highly diverse compared to normal HDFs. Some cells in RICs were indicated strong expression of the pluripotency makers. To elucidate how RICs alter their stemness, we performed RNA-seq analysis on days 0, 2, 5, 8, 11, and 14 after His-ribosome treatment. We compared the expression patterns with RICs and other types of pluripotent stem cells^27^. Principal component analysis showed that the RIC state differed from that of control HDF and that RICs formed clusters in different positions from conventional iPS cells and ES cells (Fig. 3j). RICs state was stable after 8 days of ribosomes treatment. RICs altered the expression of cellular adhesion- and extracellular matrix-related genes (Table S2). These results indicate that ribosomes stimulate cellular dedifferentiation and induce stemness marker expression in cell clusters.

### RICs differentiate into the three germ layers derived cells *in vitro*

**Figure 4:**
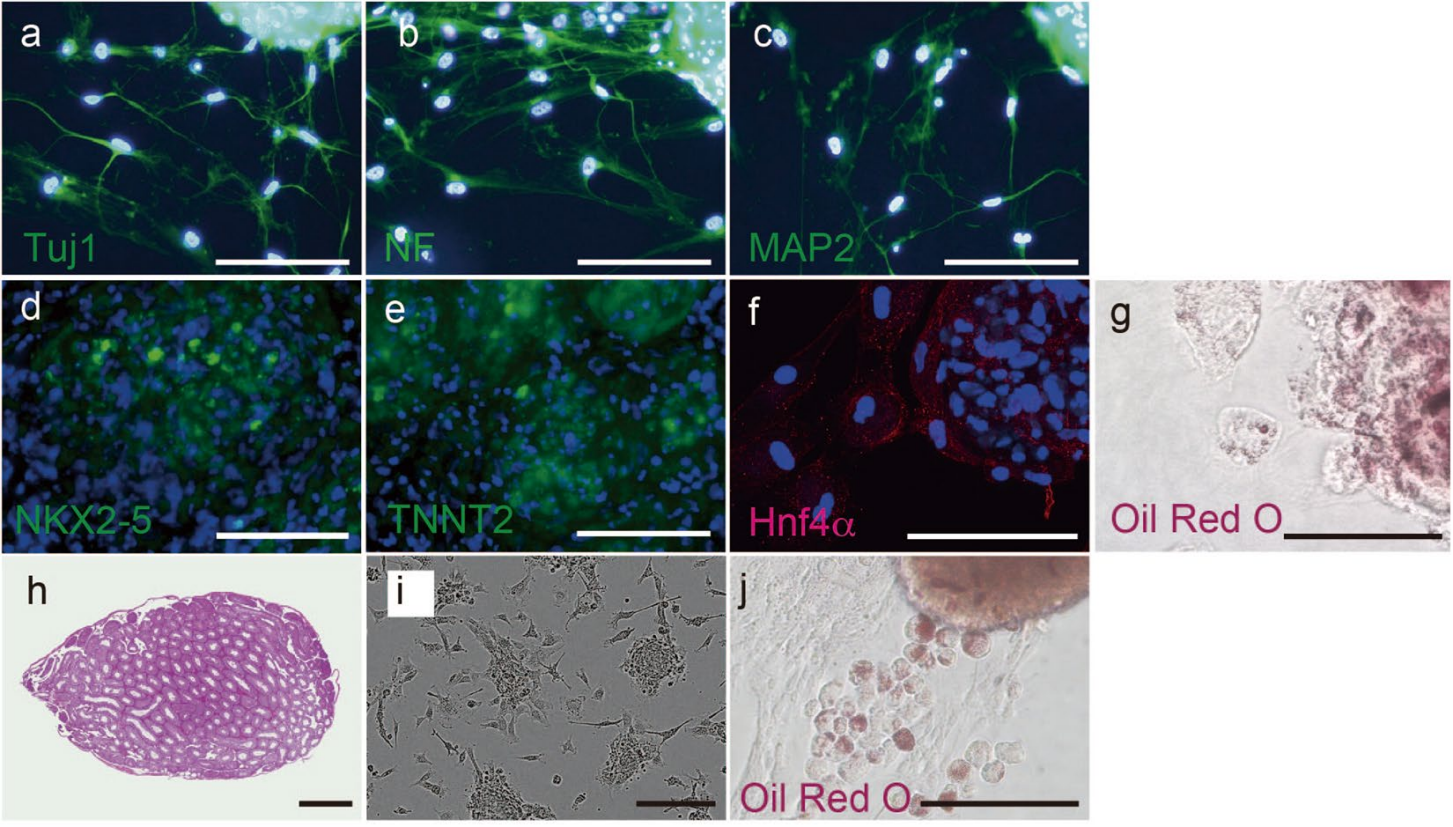
Differentiation properties of RICs. (**a**–**c**) Ectodermal neurons in RICs were detected using antibodies against the neural markers Tuj1, neurofilament (NF), and MAP2. (**d**,**e**) Staining with antibodies against the cardiomyocyte markers NKX2-5 and TNNT2. (**f**) Staining for the hepatocyte marker Hnf4α. (**g**) Oil Red O staining of hepatocytes induced from HDFs. (**h**) Testis injected with RICs. (**i**) RICs obtained from MEFs. (**j**) Oil Red O staining of hepatocytes induced from MEFs. All fluorescence images of nuclei were obtained from cells stained with Hoechst 33342 (blue). Bars = 100 µm (**a**–**g**,**i** and **j**), 1 mm (**h**).

Next, we performed *in vitro* differentiation assays and found that RICs differentiated into ectodermal neurons (transformation efficiency: 64%) (Fig. 4a–c), mesodermal cardiomyocytes (transformation efficiency: 22%) (Fig. 4d,e), and endodermal hepatocytes (transformation efficiency: 66%) (Fig. 4f,g). To evaluate *in vivo* tumor formation, we transplanted human RICs into a total of 6 testes of immunodeficient mice and observed no teratoma formation at 3 months (Fig. 4h).

We applied His-ribosomes to mouse embryonic fibroblasts (MEFs) and found that MEFs also formed cell clusters (Fig. 4i) and differentiated into oil-producing cells (Fig. 4j), indicating a hepatocyte phenotype^28^. We performed 8 trials of chimera formation assays with RICs generated from MEFs, and all 8 trials failed to indicate a contribution of RICs to the embryo under the conditions used in this study (data not shown). Collectively, our results show that ribosome treatment was effective in human and mouse cells and that RICs differentiated into the three germ-layer cells *in vitro*, while RICs differed from pluripotent stem cells in their ability to contribute to teratoma and chimera formation *in vivo*.

Ribosomes induce epithelial–mesenchymal transition (EMT)-like gene/protein expression coupled with impaired cell proliferation.

**Figure 5:**
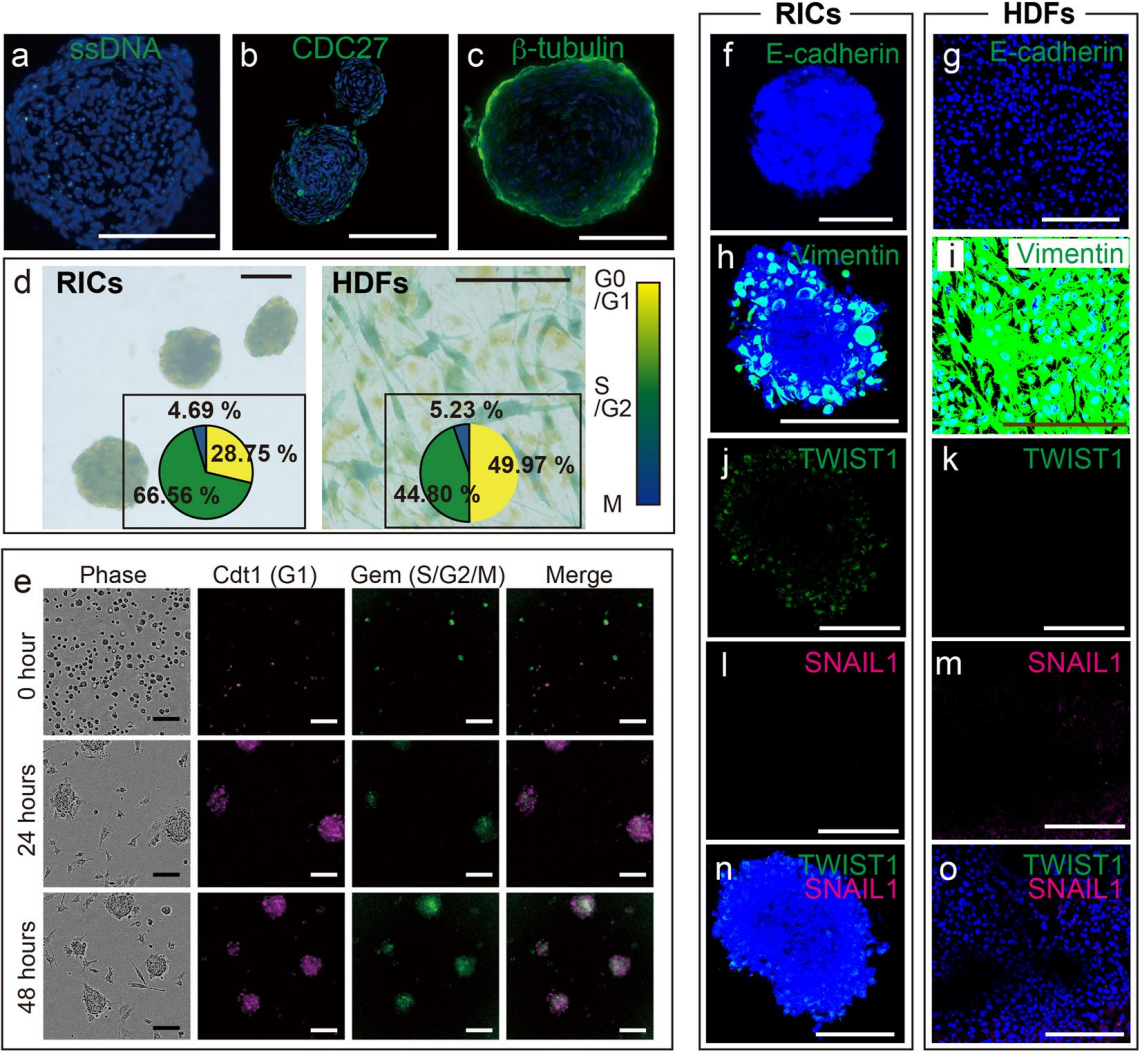
Cell cycle and mesenchymal properties of RICs. (**a**–**c**) Immunocytochemistry of RICs performed using (**a**) anti-single-strand DNA, (**b**) anti-CDC27, and (**c**) anti-β-tubulin antibodies. (**d**) Cell redox staining analysis. Small windows show colorimetric intensity ratio. (**e**) Cell cycle analysis of Fucci2-harboring MEFs. See also Video S3a,b. (**f**–**p**) Immunocytochemistry of RICs and normal HDFs performed using antibodies against the adhesion molecules E-cadherin (**f**,**g**) and vimentin (**h**,**i**). Epithelial–mesenchymal transition (EMT) markers are indicated as TWIST1 (**j**,**k**), SNAIL1 (**l**,**m**), and merged (**n**,**o**). Nuclei were stained with Hoechst 33342 (blue). Bars = 100 µm (**a**–**c**,**e**,**h**,**j**,**l**,**n**), 50 µm (**g**,**i**,**k**,**m**,**o**), 20 µm (**f**).

Treatment of HDFs with ribosomes immediately induced cell migration and cluster formation (Video S3a,b). The RICs were produced through cell aggregation, and cell division had clearly stopped in the RICs. To determine whether the RICs were undergoing apoptosis or cell proliferation arrest, we performed immunocytochemical analysis for the apoptosis marker single-stranded DNA, which revealed a low level of apoptosis (Fig. 5a). We also performed immunocytochemistry for the cell cycle markers CDC27 and β-tubulin, which showed mosaic staining patterns in RICs (Fig. 5b,c). Next, we analyzed the cell cycle in live RICs by cellular redox staining^29^, which indicated that the ratio of S/G2 phase cells was higher in RICs than in control HDFs (Fig. 5d). Finally, RICs generated from Fucci2-expressing MEFs showed mosaic patterns of cell clusters and an enrichment of S/G2M phase cells (Fig. 5e and Video S4a,b). Taken together, these results demonstrate that the cell cycle is not synchronized and that cell cycle progression is not arrested in RICs, indicating a state similar to ribosomal stress^30^.

The epithelial-mesenchymal transition (EMT) is known to play a role in embryonic development by transforming epithelial cells into mesenchymal cells, accompanied by a loss of cell adhesiveness^31^. Because of alterations in cell adhesion and the acquisition of differentiation ability into the three germ cell layers, similar to mesenchymal cells, we next compared the expression patterns of EMT markers in RICs and normal HDFs by immunocytochemistry (Fig. 5f–o). We found that the E-cadherin expression pattern did not change (Fig. 5f,g), although vimentin expression was changed in RICs (Fig. 5h,i). TWIST1, a regulator of morphogenesis and stem cell proliferation^32,33^, is expressed in RICs but not in HDFs (Fig. 5j,k,n,o), whereas the major EMT regulator SNAIL1 was not expressed in both cells (Fig. 5l,m,n,o). These data indicate that ribosomal treatment of HDFs affects expression of the EMT regulators vimentin and TWIST1 in RICs.

## Discussion

Our specific research goal is to identify cellular transdifferentiation factors in lactic acid bacteria. The cellular transdifferentiation by bacteria described previously was cell-type independent, indicating that a general underlying mechanism exists for lineage conversion by bacteria^34^. In this study, we found that ribosomes induce somatic cell transdifferentiation to restricted-multipotent cells. This can occur via donor bacteria that are swallowed and digested by host cells, which may induce ribosomal stress and stimulate cellular developmental plasticity.

Eubacteria are thought to have infected archaebacteria, after which genomic DNA was transferred between cells, resulting in the evolution of cells^35^. Recently, ribosomal proteins were reported to control not only protein translation, but also cellular proliferation and differentiation^36,37^. We suggest that ribosomal lineage conversion is involved in multicellular development by examining external organisms; other examples include cytoplasmic incompatibility caused by Wolbachia^38^, endophytic plant–microbe interactions^39^, and acquisition of chloroplasts and mitochondria through symbiogenesis^40^.

Our study showed that pluripotency marker NANOG and OCT4 was induced by ribosome incorporation and transfer cellular state that to be able to induce neuronal, muscle and hepatic cells marker expressed cells by *in vitro* culture. Unlike conventional pluripotent stem cells, expression of pluripotent markers in some of RICs consisting cells were few and failed to lead functional cell differentiation, teratoma formation, and chimeric mice generation. During iPS cell generation, pluripotent intermediate cells decreased in cell volume and underwent proliferation arrest^41^. Moreover, when a chemical approach involving small molecules was employed to reprogram mouse somatic cells into pluripotent stem cells, the cells passed through an extraembryonic endoderm-like state during completion of pluripotency^42^; incubation for 40 days with chemically defined factors caused the cells to transition from the extraembryonic endoderm-like state into pluripotent stem cells. Thus, it will be particularly interesting to extend the culture duration and investigate the molecular roadmap underlying ribosomal lineage transdifferentiation, which leads to a higher potential for developmental ability.

Based on global expression analysis, RICs alter cellular pluripotency, which differs from conventional pluripotent stem cells. In OCT4 and NANOG double-stained cells, we detected highly demethylated sequences in RICs and various gene expression patterns of pluripotent markers. PCA analysis by RNA-seq showed the RICs state is maturated at day 8 after ribosomes incorporation. This suggests that RICs aggregate and show heterogenous stemness. As described for iPS cell generation, elite or stochastic models of acquisition pluripotency have been evaluated previously^43,44^. Improvement in the expression of pluripotency markers and focused analysis of pluripotent cells will reveal whether the elite or stochastic model is better for RIC formation.

The EMT is essential for embryonic development, although it induces fibrosis and carcinoma generation^45,46^. We observed expression of EMT regulators in ribosome-induced cell clusters; however, it remains unclear whether cellular regulation and function were affected by the HDF phenotype. Because ribosomal transdifferentiation is simple *in vitro*, we will focus on cancer cell transdifferentiation to investigate cancer cell plasticity and develop new therapeutics. Cellular lineage transdifferentiation of cancer cell lines can be conducted to understand the bivalent phase of EMT between development and carcinogenesis.

Cellular transdifferentiation by ribosomes was achieved by trypsin digestion-activated endocytosis without genetic modification. The ribosome is a very large cellular component that can be isolated by centrifugation; thus, cellular transdifferentiation can be performed using the cells’ own ribosomes. These findings can be applied in regenerative medicine to avoid genetic modifications and immunogenic reactions. Furthermore, for *in vivo* transdifferentiation, this method can be applied to translocate ribosomes to a targeted area by using a bacterial vehicle^47,48^.

Although Waddington’s epigenetic concept is widely supported as the cause of hierarchical restrictions and robustness of cell differentiation during normal development, other mechanisms for overcoming cascade barriers to accommodate transdifferentiation and cellular reprogramming have been proposed^49^. Currently, cellular reprogramming through nuclear transfer into oocytes and the use of extrinsic stimuli such as transcription factors or small molecules is widely accepted; thus, future analysis of ribosomal transdifferentiation will provide a unique model for cellular lineage plasticity.

## Materials and Methods

### Cells and bacterial culture

All cell cultures were handled according to the guidelines of the Committee on Animal Research at the Kumamoto University and Institutional Committee of Laboratory Animal Experimentation of the RIKEN Center for Developmental Biology. All cells and bacterial strains are listed in Table S3. HDFs purchased from Cell Applications (San Diego, CA, USA) were used within 3 passages from culturing of a new vial. HDFs were maintained by Human dermal fibroblast growth medium kit for adult (cat. No. CA116K500a, Cell applications, Fig.S1f). MEFs were isolated from stage 14.5 days post-coitum (dpc) embryos of the R26p-Fucci2 transgenic line^50^. HDFs and MEFs were maintained at 37 °C in a humidified atmosphere containing 5% CO_2_.

### Ribosome-induced cell cluster (RIC) formation

For buffer desalting, the ribosome dilution buffer was replaced with PBS through ultrafiltration (Amicon Ultra 3000 Da MW; Merck, Darmstadt, Germany). Cells were collected using conventional trypsin digestion. The obtained cell suspensions were adjusted to approximately 2 × 10^5^ cells/mL in PluriSTEM Human ES/iPS Medium (Merck) and then applied to a 4-well plate containing the assay solutions. The plated cells were cultured for 14 days, with half of the medium replaced every 3–4 days. Transdifferentiation induction activities were calculated based on the number of cell clusters observed for approximately 2 × 10^4^ HDFs in each 96-well plate. All immunocytochemistry, expression, and fate-conversion experiments were performed using RICs that had been cultured for 14 days in PluriSTEM Human ES/iPS Medium.

### Identification of the transdifferentiation factor in *L. acidophilus*

#### Cluster formation activity

Cell cluster formation activity was evaluated using a cluster-formation assay (cluster formation counts/well); 20 µL of each cell fraction or chromatographic fraction was applied to 2 × 10^4^ HDFs cultured in a 96-well plate and cell clusters were counted after incubation for 24 h.

#### Cell disruption

Cultured *L. acidophilus* cells were collected by centrifugation, washed with cold PBS, resuspended in PBS, and disrupted by sonication (Output 6, duty 50%, 15 min on ice; Branson Sonifier 250D, Danbury, CT, USA). The suspensions were centrifuged at 10,000 × g for 30 min at 4 °C to remove cell debris. The supernatant in PBS after centrifugation was collected and used for cell cluster formation for Fig. 1a.

#### Identification of the transdifferentiation factor by ultrafiltration

The disrupted cell solution was applied to Amicon Ultra 100-kDa filters (Merck) and centrifuged at 6000 × g for 30 min at 4 °C. The upper phase and lower phase of the filter were adjusted to a concentration 1 µg/µL protein by PBS and assayed for cell cluster formation activity for Fig. 1b. Protein concentration was determined using a protein assay kit (Bio-Rad, Hercules, CA, USA).

#### Anion-exchange chromatography

*L. acidophilus* cells were collected and sonicated by the method described in “Cell disruption” section. The supernatant solution was salted out with ammonium sulfate (10–60%). Precipitates were collected by centrifugation, resuspended in 5 mL of TD buffer (10 mM Tris-HCl (pH 7.8) and 1 mM dithiothreitol (DTT)), and filtered through a 0.45-µm filter cassette. Solutions were purified by ultrafiltration (Amicon Ultra 100-kDa filters; Merck) and collected upper phase of the filtration cassette. Chromatography was performed on an AKTAprime chromatography system (GE Healthcare, Little Chalfont, UK) (Fig. 1c). The crude purified solution was run through a HiPrep Q FF 16/10 strong anion-exchange chromatography column (GE Healthcare) with TD buffer, and the NaCl concentration was linearly increased to 1 M. The elution procedures were performed in a 10-column-volume gradient. Chromatographic fractions were analyzed by LC/MS/MS as previously described^51^.

#### Cell cluster assay for the dissolving buffers

As negative control, we performed cell cluster assay by dissolving buffers (Fig. S9). The method was described in the section “cell cluster assay” and used dissolving buffers, PBS (Fig. S9a; control for Fig. 1a,b), TD (Fig. S9b. control for Fig. 1c) were used. No cluster was formed.

### Preparation of ribosomes

Prokaryotic ribosomes were purified by ultracentrifugation, as previously reported^52^. Briefly, a 1% volume of pre-cultured solutions (stationary-phase bacteria) was inoculated into new culture medium and incubated until the cells reached the late-stationary phase. The cultured cells were immediately chilled, collected by centrifugation, and washed with TMA-I buffer (10 mM Tris-HCl (pH 7.8), 30 mM NH_4_Cl, 10 mM MgCl_2_, and 6 mM 2-mercaptoethanol). All further operations were performed at 4 °C. The harvested cells were suspended in TMA-I buffer and disrupted by sonication. Cell debris was removed by centrifuging the suspension at 10,000 × g, and the supernatant was collected and centrifuged at 158,000 × g for 30 min; the obtained supernatant was collected and centrifuged at 116,000 × g for 6 h. The sediment was dissolved in 1 mL of TMA-I and layered on 30% sucrose containing TMA-I, and the tube was centrifuged at 158,000 × g for 15 h. The sucrose-cushion-purified 70 S ribosome precipitate was dissolved in TMA-I supplemented with 10% glycerol and rapidly chilled in liquid nitrogen. The samples were stored at −80 °C.

To purify eukaryotic ribosomes, ultracentrifugation was performed as described previously^53^. Briefly, cell pellets were suspended in HMK buffer [20 mM HEPES (pH 7.4), 100 mM KOAc, 7.5 mM Mg(OAc)_2_, 1 mM DTT, and 0.5 mM phenylmethylsulfonyl fluoride] and disrupted by vigorous shaking with glass beads (FastPrep; MP Bio Japan K.K., Tokyo, Japan). Cell debris and glass beads were removed by centrifugation and the supernatant was filtered through a 0.45-µm filter cassette. The crude cell lysate was centrifuged on a high-salt sucrose cushion (30% sucrose, 500 mM KOAc, 25 mM Mg(OAc)_2_, 1 mM DTT, and 0.5 mM phenylmethylsulfonyl fluoride) at 355,040 × g (TLA120.2; Beckman-Coulter, Brea, CA, USA) for 60 min. The sucrose-cushion-purified precipitate was dissolved in HMK buffer, aliquoted, and chilled in liquid nitrogen. The samples were stored at −80 °C.

Tetra-(His)6-tagged ribosomes isolated from *E. coli* JE28 were a kind gift from Dr. Ederth; the His-ribosomes were purified as described previously^19^. For affinity purification, a cOmplete His-tag purification column (Roche Diagnostics GmbH, Mannheim, Germany) was used with the AKTAprime chromatography system following the protocols described by the manufacturer and Ederth *et al*.

To analyze the activity of commercial ribosomes, we purchased ribosomes included in an *in vitro* protein expression system, PUREfrex 2.0 (PF201-0.2; GeneFrontier, Chiba, Japan).

To inhibit bacterial ribosomal translation, 50 µg/mL gentamicin (Sigma-Aldrich; St. Louis, MO, USA) was added to the cell culture medium.

### Immunocytochemistry

For immunocytochemistry, cell clusters were fixed with 4% paraformaldehyde for 15 min at room temperature (RT), washed with PBS, and treated with PBS containing 5% heat-inactivated goat serum and 0.1% Triton X-100 for 30 min at RT. The clusters were incubated with primary antibody overnight at 4 °C at the following dilutions: anti-OCT4 (1:200, R&D Systems, Minneapolis, MN, USA, MAB1759), anti-OCT4A (for Fig. S4, 1:200, Cell Signaling Technology (Danvers, MA, USA); #2840), anti-NANOG (1:150, Abcam (Cambridge, UK); ab21624), anti-TRA-1-60 (1:100, Molecular Probes, Sunnyvale, CA, USA, A24868), anti-SSEA4 (1:100, Molecular Probes, A24866), anti-SNAIL (1:100, Abcam; ab180714), anti-TWIST (1:50, Abcam; ab50887), anti-E-cadherin (1:50, Abcam; ab1416), anti-vimentin (1:50, Santa Cruz Biotechnology, Inc., Santa Cruz, CA, USA; sc-6260), anti-His (1:200, Abcam; ab18184), anti-β-tubulin (1:500, R&D Systems; MAB1195), anti-SOX2 (1:100, Molecular Probes, A24759), anti-desmin (1:200, Thermo Fisher Scientific, Inc., Waltham, MA, USA; RB-9014-R7), anti-neurofilament (1:100, Thermo Fisher Scientific, Inc., 13-0700), anti-β-tubulin (Tuj1, 1:1, Developmental Studies Hybridoma Bank, University of Iowa, Iowa City, IA, USA; E7- supernatant), anti-NKX2-5 (1:1000, Molecular probes, A25974) anti-TNNT2 (1:1000, Molecular probes, A25969) and anti-CDC27 (1:100, BD Biosciences, Franklin Lakes, NJ, USA), 610454). Alexa Fluor 488-conjugated phalloidin was purchased from Thermo Fisher Scientific, Inc. After 3 washes with PBS, the sections were incubated for 3 hours at RT with an anti-mouse IgG or IgM antibody conjugated with Cy3 or fluorescein isothiocyanate (FITC) (Jackson ImmunoResearch, West Grove, PA, USA), anti-rabbit IgG antibody conjugated with Cy3 or FITC (GE Healthcare), or anti-rat IgG or IgG antibody conjugated with Cy3 or FITC (Jackson ImmunoResearch); 4′,6-diamidino-2-phenylindole (DAPI) was included in the antibody dilutions. Images were constructed using ImageJ software (NIH, Bethesda, MD, USA). Hepatocyte formation was identified using an antibody against the human hepatic cell marker Hnf4α (1:200; Cell Signalling, 3113). Fluorescence images were captured using a BioRevo BZ-9000 microscope (Keyence, Osaka, Japan) and FV1200 confocal microscope (Olympus, Tokyo, Japan). Signals from the secondary antibody without the primary antibody were not detected (negative control; Fig. S4).

### Induction of RIC differentiation

*In vitro* assays for cell differentiation into adipocytes, chondrocytes, and osteoblasts were performed as previously described^9^ by using StemPro™ Adipogenesis Differentiation Kit (cat No. A1007001, Thermo Fisher Scientific, Inc.), StemPro™ Chondrogenesis Differentiation Kit (cat No. A1007101, Thermo Fisher Scientific, Inc.) and StemPro™ Osteogenesis Differentiation Kit (cat No. A1007201 Thermo Fisher Scientific, Inc.). Neural induction of RICs was performed using the Human ES/iPS Neurogenesis Kit (Millipore, Billerica, MA, USA). Briefly, RICs were cultured with Neural Induction Medium 1 (NIM1) containing GSK3, TGFβR, and AMPK inhibitors and the medium was changed to fresh NIM1 medium every other day for 5 days. Next, RICs were cultured with Neural Induction Medium 2 (NIM2) containing GSK3 and TGFβR inhibitors and the medium was changed to fresh NIM2 medium every other day for an additional 5 days. Finally, RICs were cultured with Neural Differentiation Medium containing adenosine 3′,5′-cyclic monophosphate, N6, O2′-dibutyryl-, sodium salt, and ascorbic acid 2-phosphate and the medium was changed to fresh medium every other day for 14 days.

RICs were also differentiated into cardiomyocytes by using the pluripotent stem cells Cardiomyocyte Differentiation Kit (Thermo Fisher Scientific, Inc.). Briefly, RICs were cultured on multi-well plates coated with Geltrex^TM^ (Thermo Fisher Scientific, Inc.) with Essential 8^TM^ Medium and the medium was changed to fresh Essential 8^TM^ Medium every other day for 3 days. Next, the culture medium was changed to Cardiomyocyte Differentiation Medium A containing BMP/activin activator and GSK3 inhibitor. After 2 days, the culture medium was changed to Cardiomyocyte Differentiation Medium B containing Wnt inhibitor and the medium was changed to fresh Cardiomyocyte Differentiation Medium B every other day for an additional 6 days.

RICs were differentiated into hepatocytes as previously escribed^54^. Briefly, mature HDFs, MEFs, and RICs were placed in hepatocyte differentiation basal medium containing 2 µM tauroursodeoxycholic acid; after incubation for 21 days, during which half of the medium was replaced every 2–3 days, the cells were subjected to immunostaining or Oil Red O staining.

The efficiency of transformation from RICs to differentiated cells was calculated by counting of marker-stained cells and calculating the ratio relative to the number of non-stained cells surrounding the RICs.

### Cell cycle analysis

Cell cycle progression was measured in live cells by using a CellClock assay kit (Biocolor Inc., Carrickfergus, UK) according to the manufacturer’s protocols. After 14 days of culture, RICs were analyzed using ImageJ software^55^. Thresholds were set at 140–165 (dark blue; M phase), 166–185 (green; G1/S phase), and 140–160 (yellow; G0/G1).

HDFs and MEFs were cultured with *L. acidophilus* ribosomes in PluriSTEM Human ES/iPS Medium (Merck), with half of the medium replaced every 2–3 days, and imaged using an IncuCyte ZOOM instrument (Essen BioScience, Ann Arbor, MI, USA). For imaging, we used the default software parameters for a 96-well plate with a 10× objective.

### Quantification of ribosome incorporation into RICs

We examined the cell clusters induced by His-ribosomes. After 14 days of incubation, the RICs that appeared were stained with anti-6× His (ab18184; Abcam), Alexa Fluor 488 phalloidin, or Hoechst 33342 as described in the immunocytochemistry section above. The stained cells were observed and quantified on 96-well microclear-bottom microplates (No. 655090; Greiner, Greinerstraße, Austria). The nucleus and cytoplasm were visualized from Hoechst 33342 and Alexa Fluor 488 phalloidin fluorescence signals, respectively. For all samples, we examined 4 wells (n = 4) and acquired 9 photographs per well by confocal stacking, and then quantified the signal intensity with an IN Cell Analyzer 6000 imaging cytometer system (GE Healthcare). The cytoplasm was defined by the Alexa Fluor 488 phalloidin signal and the nucleus by the Hoechst 33342 signal.

### Endocytosis inhibition assay

All chemicals used for the inhibition assay are listed in Table S4 and were purchased from Sigma-Aldrich. Because the endocytosis inhibitors were cytotoxic, cell viability assays for HDFs were performed using the thiazolyl blue tetrazolium bromide (MTT) assay following the manufacturer’s protocol (Sigma-Aldrich). HDFs were seeded at 2 × 10^4^ cells/well in 96-well plates together with 10 µg of ribosomes isolated from *L. acidophilus*. Cell clusters were counted after incubation for 24 h. All assays were conducted 4 times and the scores were analyzed using Student’s t test. The microfluorobead incorporation assay was performed using 50-nm-diameter microfluorobeads (Polysciences, Warrington, PA, USA) as previously described^56^.

### Confocal microscopy and enhancement of resolution

Images of cultured cells were recorded under a confocal microscope (A1; Nikon, Tokyo, Japan). The primary antibody for His-tagged ribosomes was anti-His tag antibody (Abcam, ab18184; 1:200), and the secondary antibody was Cy3-conjugated anti mouse-IgG antibody (Jackson ImmunoResearch, 115-165-166, 1:200).

The following is a list of instrument parts and settings used in this study. Laser lines at 405 and 561 nm were used for excitation of DAPI and Cy3, respectively. An oil-immersion objective lens (Apo TIRF 100× Oil, NA = 1.49; Nikon) was used to capture high-magnification images. The pixel size of the confocal images was set to 30 nm. The pinhole size was set to 0.3–0.6 AU. To enhance the resolution, confocal images were deconvolved by using NIS-Elements C-ER software (Nikon) in some cases. All images were recorded at RT (25.5 °C ± 0.5 °C).

### Transcriptome analysis

Total RNA for microarray analysis was extracted from RICs and control HDFs after 14 days of culture; we used TRIzol reagent (Thermo Fisher Scientific Inc.) with 1.0-mm glass beads (BSP-11079110W, WAKENBTECH, Shiga, Japan), a KONTES Homogenizer pestle (Kimble Chase, Vineland, NJ, USA), and a ReliaPrep RNA Cell Miniprep System (Promega, Madison, WI, USA) for purification. RNA quality was assessed using a Bioanalyzer (Agilent Technologies, Santa Clara, CA, USA). To generate Cy3-labeled cRNA, total RNA (150 ng) was reverse-transcribed using a Low-Input Quick Amp Labeling Kit, one-color (Agilent Technologies). The cRNA was fragmented and hybridized to the SurePrint G3 Human Gene Expression 8 × 60 K v2 chip (Agilent Technologies) according to the manufacturer’s protocol, with 3 technical replicates used for each cell type. Microarray chips were scanned using a DNA Microarray Scanner (Agilent Technologies). Microarray data were feature-extracted using Agilent Feature Extraction software (ver. 11.5.1.1). Microarray intensities were intra- and inter-array normalized using Limma package version 3.14.4^57^ in the R statistical computing environment. For visualization using heatmaps, normalized microarray intensities for each gene were further represented as z-scores among all cell types (Fig. S5). Information for gene function categories such as “somatic stem cell maintenance” was extracted from the Gene Ontology Database^58^.

Total RNA for RNA-seq analysis was extracted on days 0, 2, 5, 8, 11, and 14 after ribosome treatment of HDF clusters. RNA extraction and quality evaluation procedures were the same as those used for microarray analysis. RNA-seq library construction and sequencing were performed by Filgen (Nagoya, Japan). Briefly, Sequencing libraries were generated using NEBNext® Ultra™ RNA Library Prep Kit for Illumina® (NEB, USA) following manufacturer’s recommendations and index codes were added to attribute sequences to each sample. mRNA was purified from total RNA using poly-T oligo-attached magnetic beads. Fragmentation was carried out using divalent cations under elevated temperature in NEBNext First Strand Synthesis Reaction Buffer (5×). First strand cDNA was synthesized using random hexamer primer and M-MuLV Reverse Transcriptase (RNase H^−^). Second strand cDNA synthesis was subsequently performed using DNA Polymerase I and RNase H. Remaining overhangs were converted into blunt ends via exonuclease/polymerase activities. After adenylation of 3′ ends of DNA fragments, NEBNext Adaptor with hairpin loop structure were ligated to prepare for hybridization. In order to select cDNA fragments of preferentially 250–300 bp in length, the library fragments were purified with AMPure XP system (Beckman Coulter, Beverly, USA). Then 3 µl USER Enzyme (NEB, USA) was used with size-selected, adaptor-ligated cDNA at 37 °C for 15 min followed by 5 min at 95 °C before PCR. Then PCR was performed with Phusion High-Fidelity DNA polymerase, Universal PCR primers and Index (X) Primer. At last, PCR products were purified (AMPure XP system) and library quality was assessed on the Agilent Bioanalyzer 2100 system. Sequencing was performed on Illumina HiSeq. 4000 with RTA version 2.7.7 software for base calling.

RNA-seq data were filtered using the following criteria: (1) remove reads containing adapters; (2) remove reads containing N > 10%, where N represents a base that cannot be determined; (3) remove reads containing low-quality (Qscore ≤ 5) bases accounting for more than 50% of the total bases. Next, RNA-seq reads were mapped to the human transcriptome using Tophat2 program version 2.1.1^59^; the genes.gtf file in the Illumina iGenome hg19 was used for transcriptome annotation. The number of reads mapped for each gene was counted using HTSeq package version 0.7.2^60^. Differentially expressed genes between days 0 and 14 (false-discovery rate < 0.05) were detected using edgeR package version 3.16.5^61^, and GO enrichment analysis was performed using the DAVID web server version 6.8^62^. For principal component analysis, read counts were transformed into counts per million and then log2-transformed. To compare our RIC data and the ESCs/iPSCs data from Ma *et al*.^27^, batch effects were corrected using ComBat program in SVA package version 3.22.0^63^ so that HDFs from the both studies had concordant expression profiles.

The microarray data and the RNA-seq data were deposited in the NCBI Gene Expression Omnibus database with the accession number GSE99089 (https://www.ncbi.nlm.nih.gov/geo/query/acc.cgi?acc = GSE99089).

### qPCR analysis of gene expression

iPS cells were generated from human adipose-derived mesenchymal stem cells as described previously^64^. Samples were run in triplicate and expression was normalized to the levels of human Rplp0 (36b4). The samples were analyzed by qPCR performed using THUNDERBIRD SYBR qPCR Mix (TOYOBO, Osaka, Japan). Endogenous versus exogenous gene expression was quantified as previously reported^65^. Statistical comparisons were performed using Student’s t test. Error bars indicate the mean ± SEM.

For single-cell analysis, day 14 RICs were digested by trypsin (Cell Applications, San Diego, CA, USA) for 5 min at 37 °C. Digested RICs were dissociated by 23 G syringe pumping several times. Cells were passed through a 70 µm diameter cell strainer and clonally sorted into 96‐well PCR plates by BD FACS Aria II (BD Biosciences) with Hoechst-positive cells. First-strand cDNA was synthesized using CellsDirect One‐Step qRT‐PCR kits (Thermo Fisher Scientific, Inc.) and a Taqman probe and primer set for each gene (Table S5). The cDNA was preamplified by 20 cycles of PCR and real-time qPCR was performed in TaqMan Fast Advanced Master Mix (Life Technologies, Carlsbad, CA, USA) with Taq man probes using the BioMark^TM^ HD system (Fluidigm, South San Francisco, CA, USA). Expression levels of RICs and HDFs were normalized by Gapdh expression. The graph was depicted the Beeswarm plot superimposed on the Box plot by using R with Package beeswarm^66^.

### Bisulfite sequencing

Genomic DNA was isolated by using a DNeasy Blood & Tissue Kit (Qiagen, Hilden, Germany). Bisulfite conversion of genomic DNA was performed using a MethylEasyXeed Kit (TAKARA, Shiga, Japan) and the product was immediately amplified by using EpiTaq HS (TAKARA) with OCT4, NANOG, and LMNA primer pairs, which are listed in Table S6. Sequence data were analyzed on the QUMA server in RIKEN CDB^67^.

### Chromosome copy number variation analysis

Genomic DNA was extracted from cell clusters using a DNeasy Blood & Tissue Kit (Qiagen). The quality and quantity of genomic DNA were assessed using the Qubit Fluorometric Quantification assay kit (Thermo Fisher Scientific, Inc.). Hybridization scanning, conducted by the Support Center for Advanced Medical Sciences, Institute of Biomedical Sciences, Tokushima University Graduate School, was performed on a Cytoscan HD array (Affymetrix, Santa Clara, CA, USA), which integrated >2.6 million copy-number markers across the human genome. Scanning data from the Affymetrix GeneChip Command Console were analyzed using the Chromosome Analysis software suite (Affymetrix).

### Teratoma formation

Teratoma formation was performed according to the guidelines of the Committee on Animal Research at the Kumamoto University. RICs were collected by collagenase IV treatment and injected into the testes of NOD-SCID immunodeficient mice at 5 × 10^5^ cells per testis^68^. The 6 testes were collected approximately 12–16 weeks after injection and fixed in 10% formalin, and then processed for paraffin embedding and hematoxylin-eosin staining following standard procedures.

### Generation of mouse chimeras with RICs

Generation of mouse chimeras was approved by the Committee of RIKEN Kobe Branch, and performed in accordance with the RIKEN Kobe Branch animal experimentation guidelines. RICs derived from MEFs carrying the R26-H2B-EGFP reporter allele^69^ were injected into host CD1 embryos at the 8-cell or blastocyst stage, which were then transferred to 2.5 dpc pseudopregnant recipients. Injected embryos were dissected at 9.5–11.5 dpc, and chimerism was assessed based on the EGFP signal, representing the contribution of RICs, under a fluorescence stereoscope.

## Electronic supplementary material

Supplementary Information

videoS1

videoS2

videoS3a

videoS3b

videoS4a

videoS4b
