# Supplementary material for: Ribosome Incorporation into Somatic Cells Promotes Lineage Transdifferentiation towards Multipotency
Source: Sci Rep. 2018 Jan 26;8:1634. doi: 10.1038/s41598-018-20057-1 (PMC5786109; doi:10.1038/s41598-018-20057-1)
Supplement: Supplementary file 1 — Supplementary Information [file 41598_2018_20057_MOESM1_ESM.doc]

Ribosome Incorporation into Somatic Cells Promotes Lineage Transdifferentiation towards Multipotency

Naofumi Ito^1, 2^, Kaoru Katoh^3^, Hiroko Kushige^4^, Yutaka Saito^5^, Terumasa Umemoto^6^, Yu Matsuzaki^6^, Hiroshi Kiyonari^7^, Daiki Kobayashi^8^, Minami Soga^9^, Takumi Era^9^, Norie Araki^8^, Yasuhide Furuta^7^, Toshio Suda^6, 10^, Yasuyuki Kida^4^, and Kunimasa Ohta^1, 2, 11, 12^*

^1^Department of Developmental Neurobiology, Graduate School of Life Sciences, Kumamoto University, 1-1-1 Honjo, Chuo-ku, Kumamoto 860-8556, Japan

^2^Program for Leading Graduate Schools “HIGO Program”, Kumamoto University, 1-1-1 Honjo, Chuo-ku, Kumamoto 860-8556, Japan

^3^Biomedical Research Institute, National Institute of Advanced Industrial Science and Technology, Central 6, 1-1-1 Higashi, Tsukuba, Ibaraki 305-8566, Japan

^4^Biotechnology Research Institute for Drug Discovery, National Institute of Advanced Industrial Science and Technology (AIST), 1-1-1 Higashi, Tsukuba, Ibaraki 305-8565, Japan

^5^Biotechnology Research Institute for Drug Discovery, National Institute of Advanced Industrial Science and Technology (AIST), 2-4-7 Aomi, Koto-ku, Tokyo 135-0064, Japan

^6^International Research Center for Medical Science, Kumamoto University, 2-2-1 Honjo, Chuo-ku, Kumamoto City 860-0811, Japan

^7^Animal Resource Development Unit and Genetic Engineering Team, RIKEN Center for Life Science Technologies, 2-2-3 Minatojima-minamimachi, Chuo-ku, Kobe, Hyogo 650-0047, Japan

^8^Department of Tumor Genetics and Biology, Graduate School of Life Sciences, Kumamoto University, 1-1-1 Honjo, Chuo-ku, Kumamoto 860-8556, Japan

^9^Department of Cell Modulation, Institute of Molecular Embryology and Genetics, Kumamoto University, 2-2-1 Honjo, Chuo-ku, Kumamoto 860-0811, Japan

^10^Cancer Science Institute of Singapore, National University of Singapore, Centre for Translational Medicine,14 Medical Drive, 117599, Singapore

^11^International Research Core for Stem Cell-based Developmental Medicine, Kumamoto University, 1-1-1 Honjo, Chuo-ku, Kumamoto 860-8556, Japan

^12^Japan Agency for Medical Research and Development (AMED), Tokyo 100-0004, Japan

*Correspondence should be addressed to Kunimasa Ohta

Phone: (81)-96-373-5293 E-mail: [ohta9203@gpo.kumamoto-u.ac.jp](mailto:ohta9203@gpo.kumamoto-u.ac.jp)


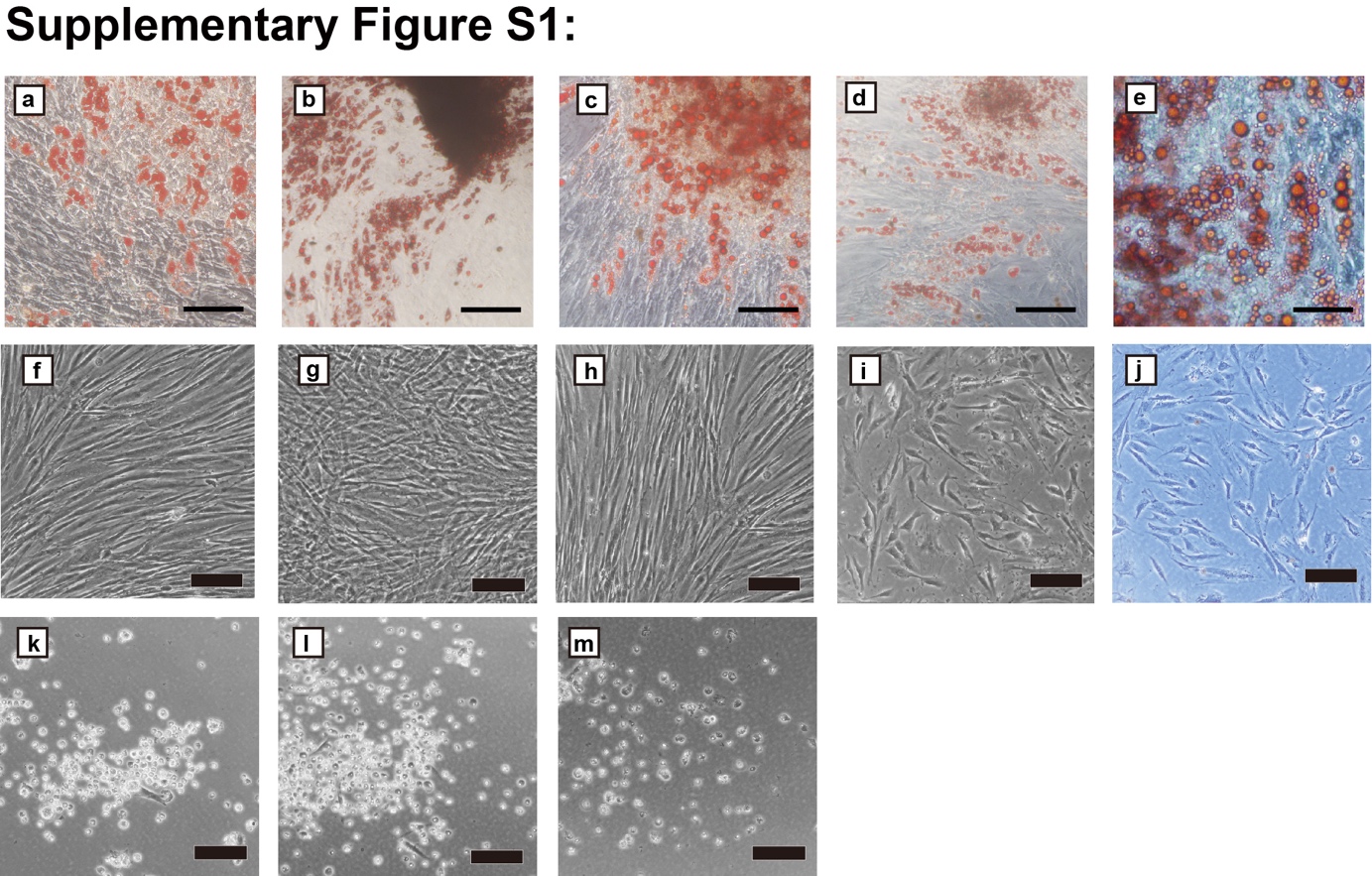


**Supplementary Figure S1.** Differentiation of ribosome-incorporated cell clusters into adipocytes. Various clusters generated using ribosomes isolated from the species listed below were cultured in adipocyte differentiation medium and stained with Oil Red O. Ribosomes were isolated from (a) *Lactobacillus acidophilus* (lactic acid-producing gram-positive bacterium), (b) *Staphylococcus epidermis* (grape-like-cluster-forming gram-positive bacterium), (c) *Saccharomyces cerevisiae* (budding yeast), (d) *Rattus norvegicus* IEC-6 cells (rat small intestine cells), and (e) *Escherichia coli* JE28 (MG1655 derivative gram-negative bacterium). (f) Conventional HDFs cultured in ’Human dermal fibroblast growth medium kit for adult’. (g) HDFs cultured in PluriSTEM Human ES/iPS Medium. (h) HDFs of dish ‘g’ subcultured using "human dermal fibroblast cell growth medium kit for adult". (i) HDFs of dish ‘g’ subcultured using " StemPro™ Adipogenesis Differentiation Kit". (j) Oil red O staining of dish ‘I’. (k) HDFs of dish ‘g’ subcultured using " StemPro™ Chondrogenesis Differentiation Kit". (l) HDFs of dish ‘g’ subcultured using " Human ES/iPS Neurogenesis Kit ". (m) HDFs of dish ‘g’ subcultured using ‘pluripotent stem cells Cardiomyocyte Differentiation Kit’. Bars = 100 μm (a, b, c, and d), 50 μm (e-m).


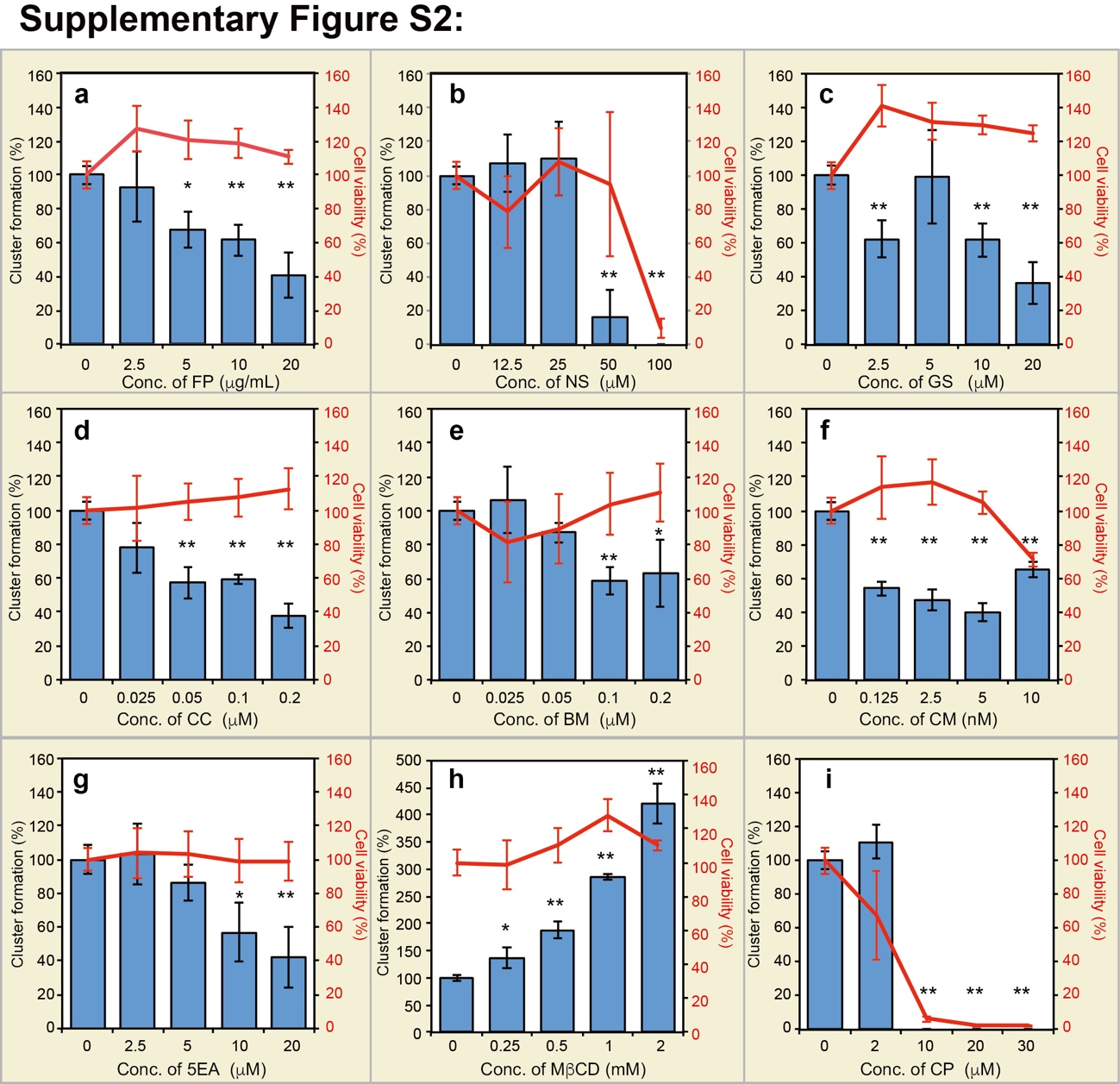


**Supplementary Figure S2.** Effect of endocytosis inhibitors on RIC formation. Quantification of the effect of inhibitors on RIC formation and cell viability (examined using the MTT assay) is shown as a percentage of the RIC number obtained without inhibitor treatment. (a) Filipin (FP), (b) nystatin (NS), (c) genistein (GS), (d) cytochalasin B (CC), (e) bafilomycin A1 (BM), (f) concanamycin A (CM), (g) 5-(N-ethyl-N-isopropyl)-amiloride (5NA), (h) methyl-β-cyclodextrin (MβCD), and (i) chlorpromazine (CP).

**
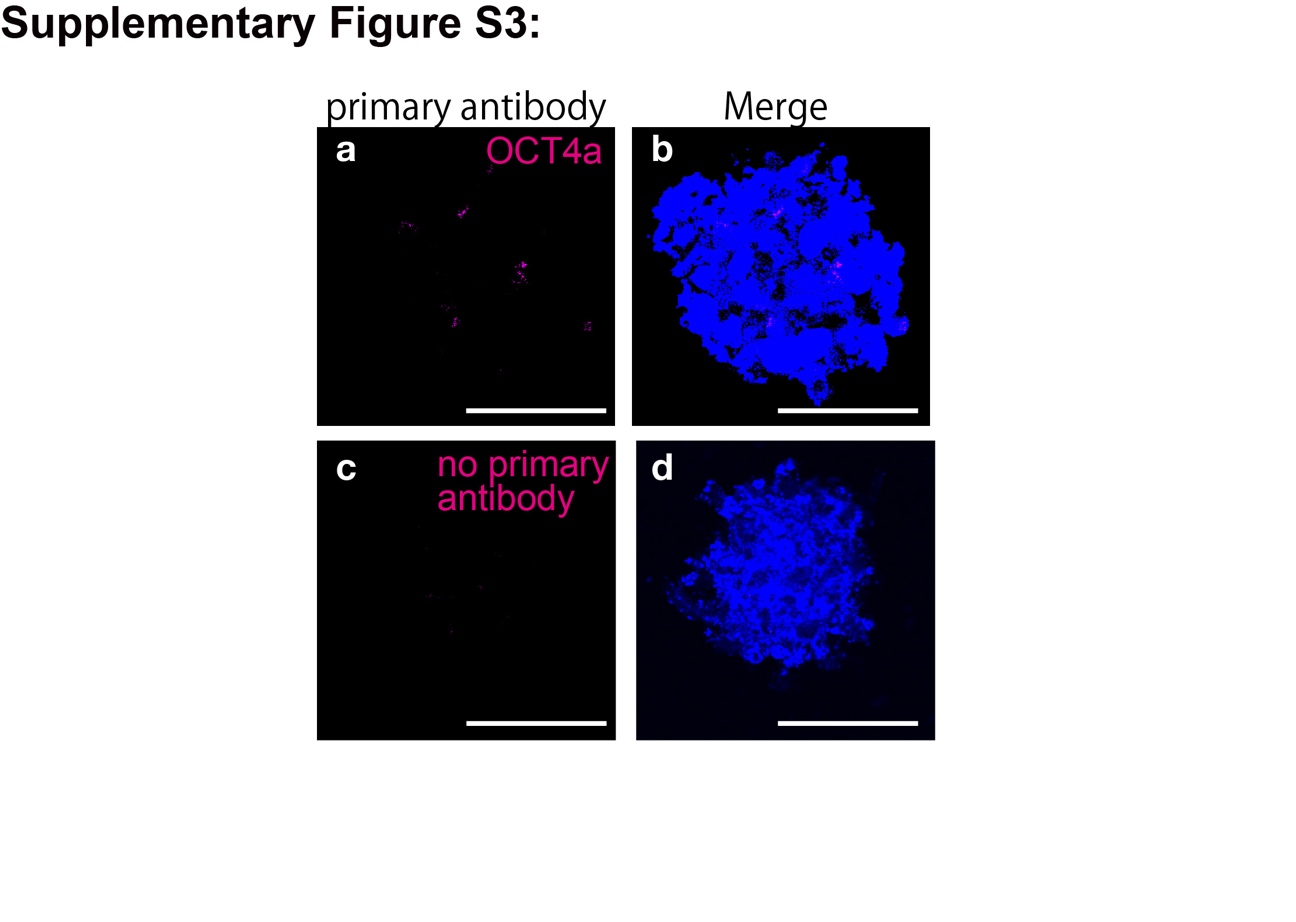
**

**Supplementary Figure S3.** Immunocytochemistry of RICs performed using antibodies against OCT4a. OCT4 has been reported to have transcriptional variation, and OCT4a has been shown to give rise to cellular pluripotency^1^. Fig. S3A and B show Oct4a signals (magenta). Fig. S3c and d show the respective negative controls without the primary antibody. Nuclei were stained with Hoechst 33342 (blue). Bars = 50 μm.

1 Wu, G. *et al.* Establishment of totipotency does not depend on Oct4A. *Nat Cell Biol* **15**, 1089-1097 (2013).


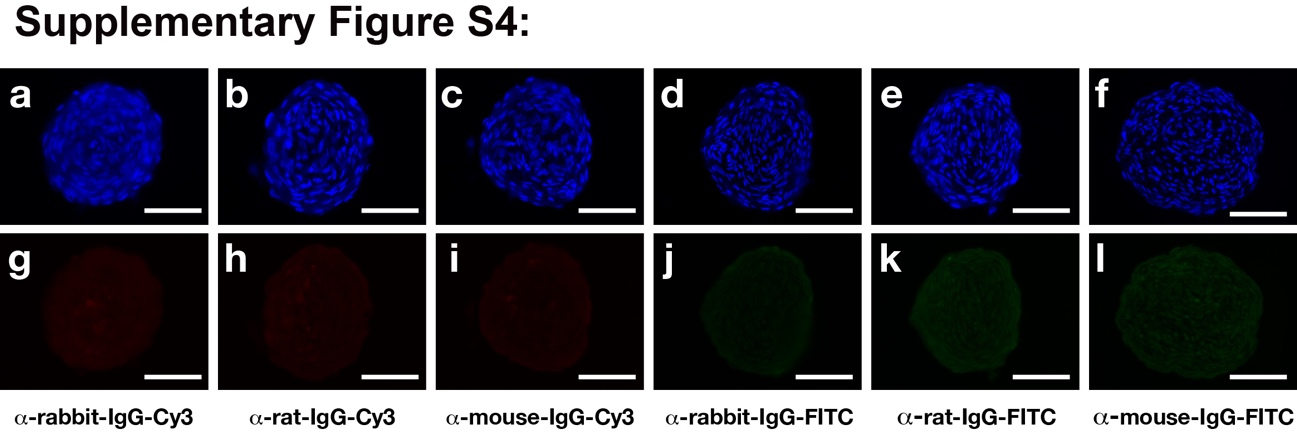


**Supplementary Figure S4.** Immunocytochemistry of RICs performed using a secondary antibody to evaluate non-specific binding. Nuclear staining with Hoechst 33342 (blue, Fig. S4a-f) and RIC immunostaining with Cy3 (Fig. S4g-i), and FITC (Fig. S4j-l) were performed using the same conditions shown in Fig.3 and Fig. 4. Bars = 100 μm.


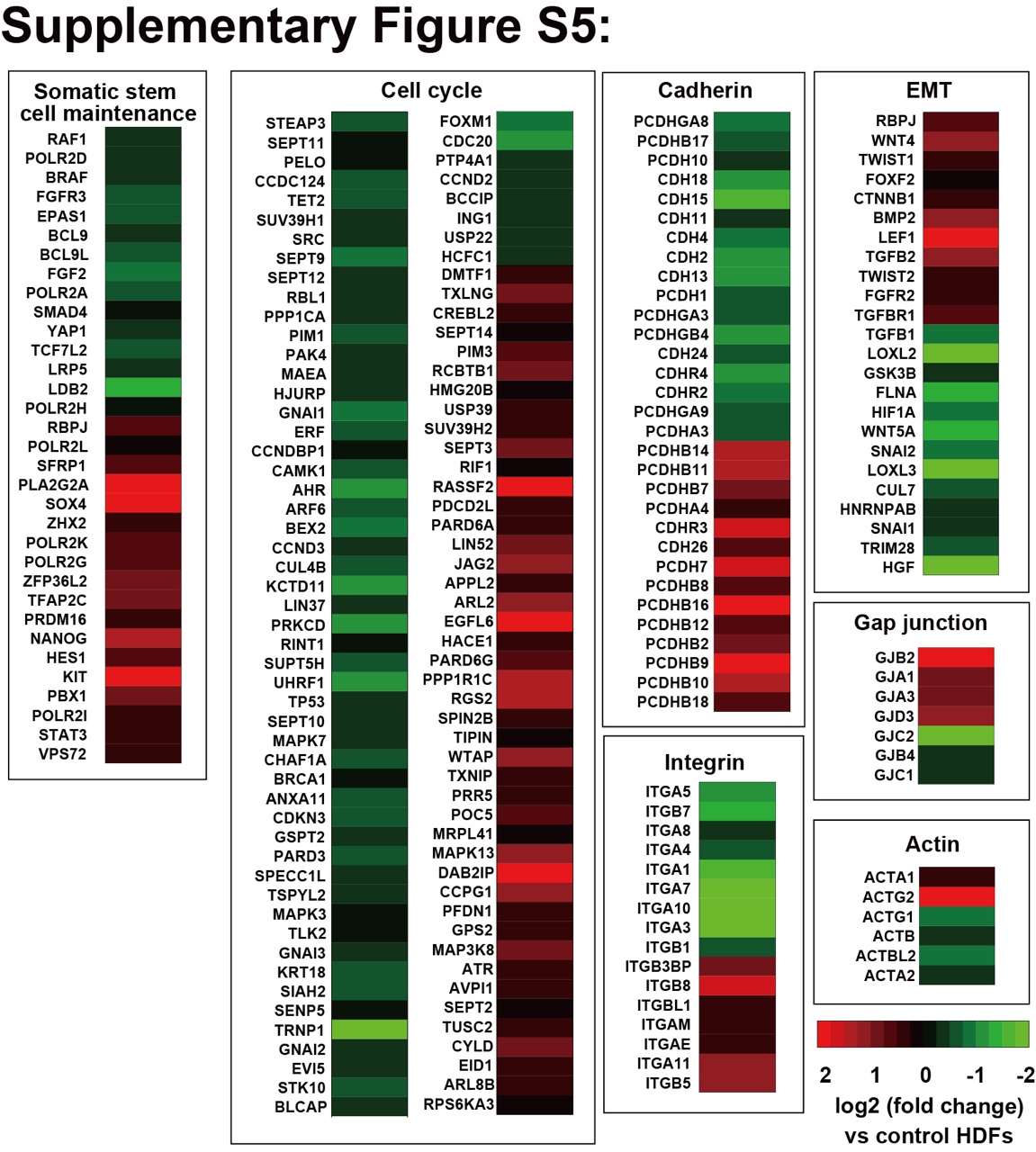


**Supplementary Figure S5.** Global gene expression profiles in L-ribosome-induced RICs for genes involved in stem cell maintenance, cell cycle, the epithelial–mesenchymal transition (EMT), and adhesive function. Fold change in expression of genes differentially expressed between L-ribosome-induced RICs and control HDFs (false-discovery rate < 0.05). For the analyses, n = 3.

**
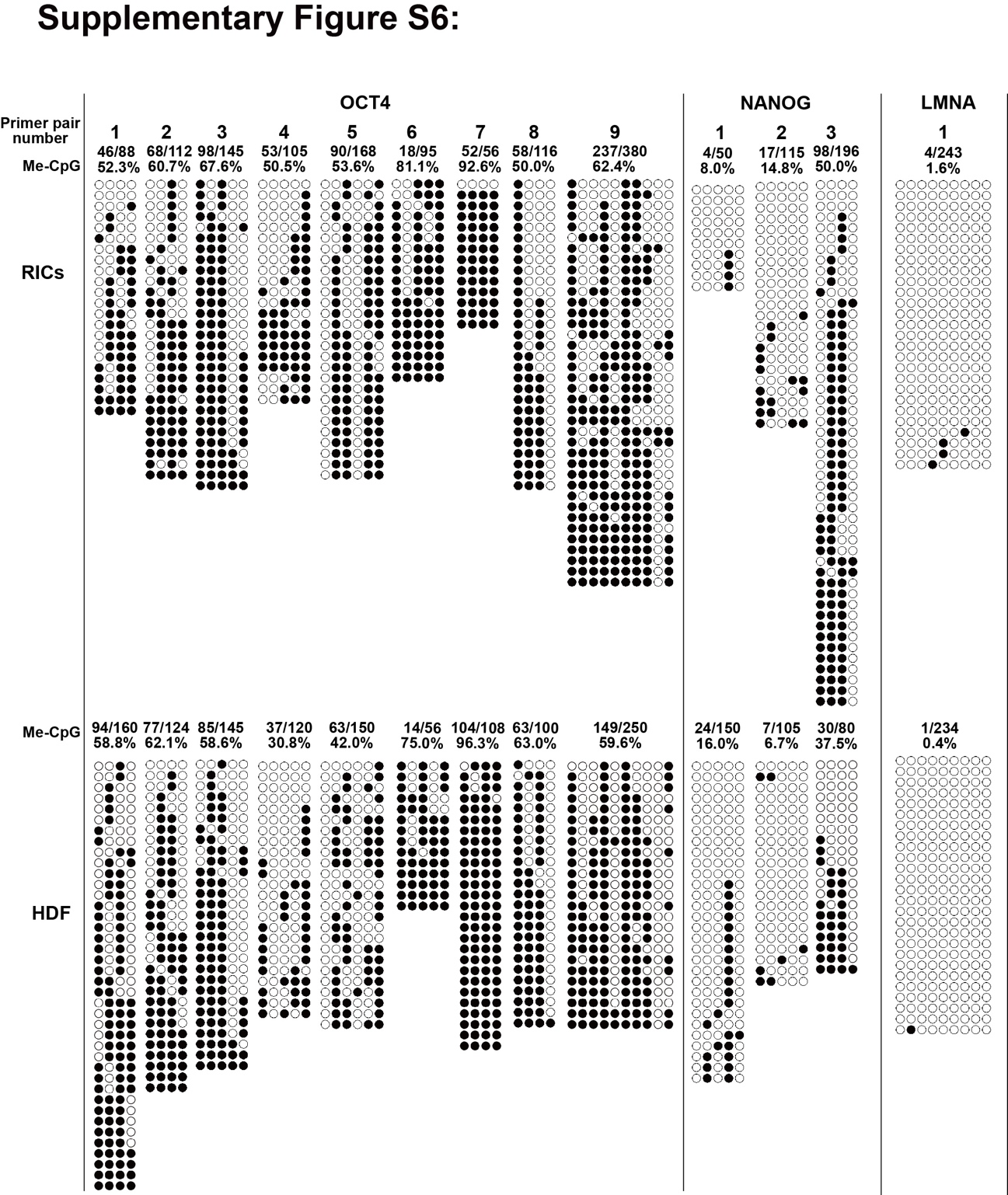
**

**Supplementary Figure S6.** Bisulfite sequencing of the NANOG and OCT4 translational start site. LMNA was used as a demethylated-gene control. Total methylated CpG (Me-CpG) counts and ratios are shown. Black circles indicate methylated cytosine residues and white circles indicate unmethylated cytosine residues. Primers are listed in Table S4.


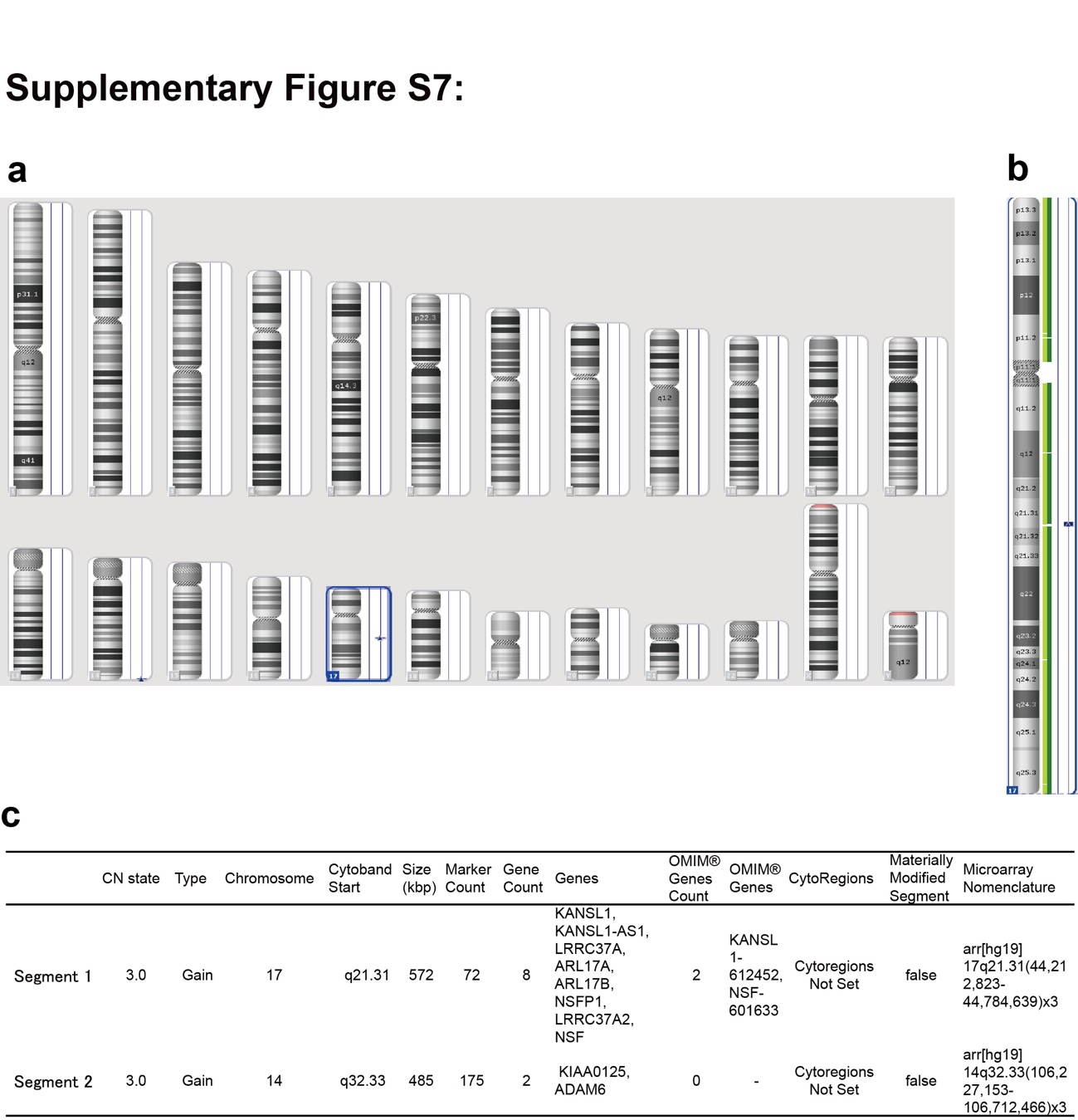


**Supplementary Figure S7.** Copy number variation analysis of RICs by using Cytoscan. (a) The triangle in the genomic representation indicates the duplicated area of the RICs genome (Chromosomes 14 and 17). (b) Chromosome 14q32.33 is a region that was triplicated in the algorithm in control analyses. (c) List of duplicated genes identified using Cytoscan.


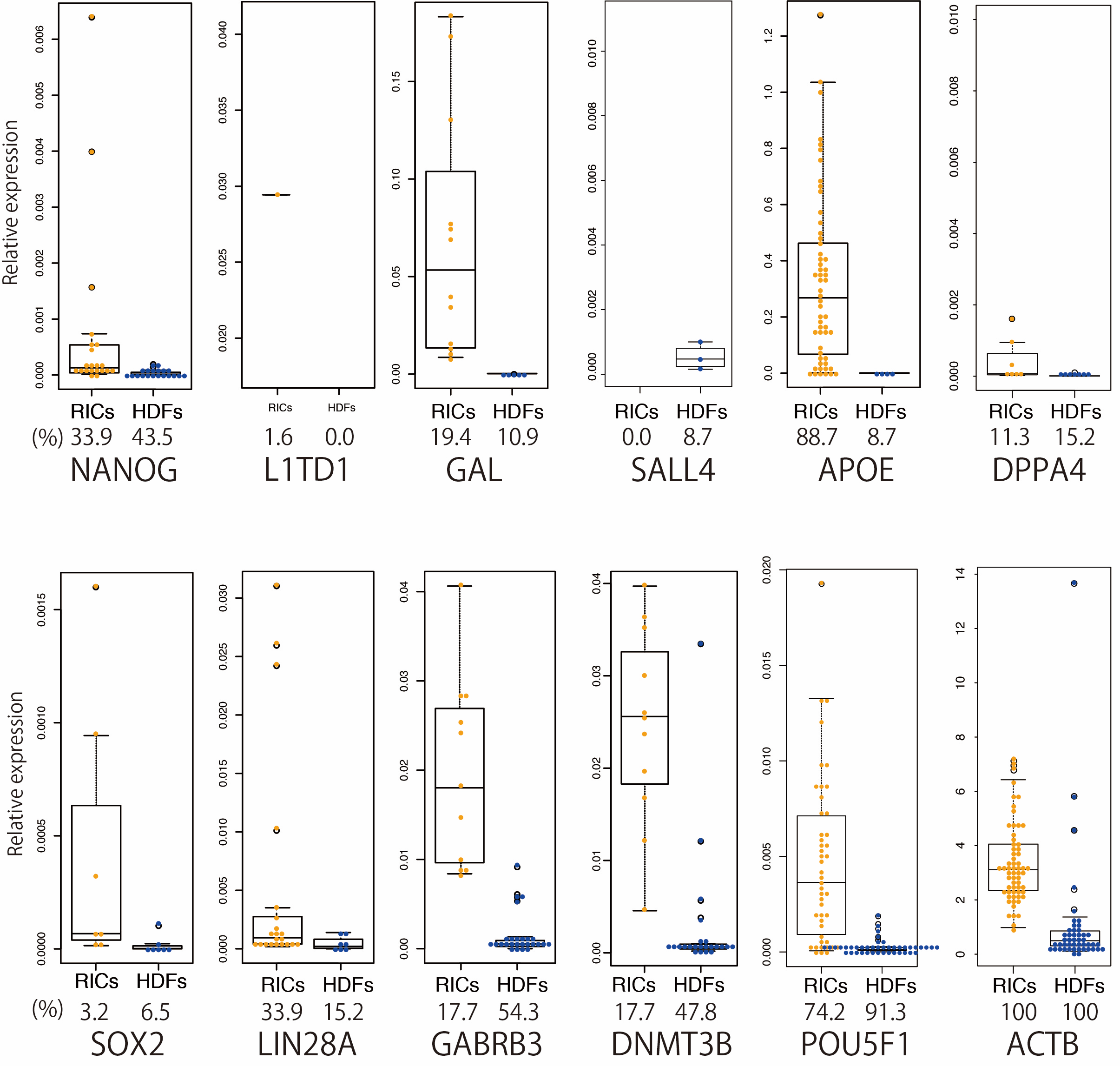


**Supplementary Figure S8.** Single cell qPCR assay compared with RICs and conventional HDFs. Dots indicate each single cell expression levels. Values are the gene expression levels normalized with GAPDH expression. The box plot shows the median and quartile values, whiskers show outliers within 1.5 times the interquartile range of the quartile. The percentage showing amplification numbers in GAPDH positive cells for the selected genes are shown below the plot. A dotless lane means that there was no cell in which expression was detected.


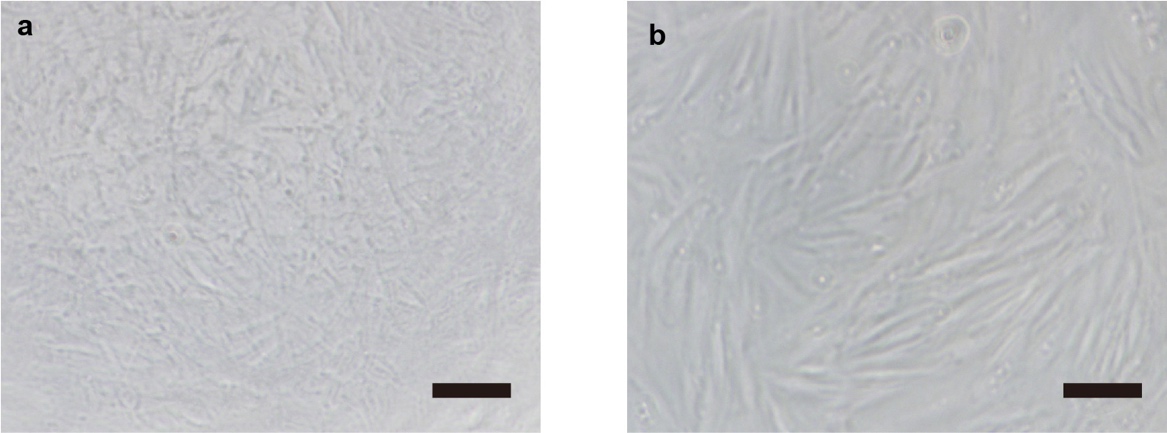


**Supplementary Figure S9.** Cell cluster formation assay using cell lysate prepared solution. HDFs were subcultured with (a) PBS and (b) TD buffer (10 mM Tris-HCl (pH 7.8) and 1 mM dithiothreitol). Bars = 100 µm.

**Supplementary Table S1:** Identification of proteins from chromatographically separated fractions determined based on LC/MS/MS analysis. Identification of proteins from chromatographically separated fractions determined based on LC/MS/MS analysis. The most active peak fraction (Fraction No. 21 (F21), grayed out) and the preceding low-activity peak (Fraction No. 20 (F20)) were compared, and protein counts were higher in F21 than in F20. The most active peak fraction (Fraction No. 21 (F21), grayed out) and the preceding low-activity peak (Fraction No. 20 (F20)) were compared, and protein counts were higher in F21 than in F20.

|  |  |  |  | MS score of Peptides F21 | | | | |
| --- | --- | --- | --- | --- | --- | --- | --- | --- |
| Accession | Name | Peptides F21 (95%) | Peptides F20 (95%) | Unused | Total | %Cov | %Cov (50) | %Cov (95) |
| sp|Q5FKM6|ENO_LACAC | Enolase OS=Lactobacillus acidophilus (strain ATCC 700396 / NCK56 / N2 / NCFM) GN=eno PE=3 SV=1 | 76 | 72 | 60.09 | 60.09 | 86.45 | 77.10 | 72.43 |
| sp|Q5FMJ3|GPMA_LACAC | 2,3-bisphosphoglycerate-dependent phosphoglycerate mutase OS=Lactobacillus acidophilus (strain ATCC 700396 / NCK56 / N2 / NCFM) GN=gpmA PE=3 SV=1 | 72 | 57 | 45.24 | 45.24 | 82.61 | 80.43 | 80.43 |
| tr|Q5FIR0|Q5FIR0_LACAC | Fructose-bisphosphate aldolase OS=Lactobacillus acidophilus (strain ATCC 700396 / NCK56 / N2 / NCFM) GN=fbaA PE=4 SV=1 | 47 | 37 | 46.78 | 46.78 | 80.53 | 80.53 | 78.22 |
| **sp|Q5FJM3|RS2_LACAC** | **30S ribosomal protein S2 OS=Lactobacillus acidophilus (strain ATCC 700396 / NCK56 / N2 / NCFM) GN=rpsB PE=3 SV=1** | **38** | **28** | **27.41** | **27.41** | **88.37** | **78.68** | **70.16** |
| sp|Q5FM96|RPOC_LACAC | DNA-directed RNA polymerase subunit beta' OS=Lactobacillus acidophilus (strain ATCC 700396 / NCK56 / N2 / NCFM) GN=rpoC PE=3 SV=1 | 34 | 33 | 70.98 | 70.98 | 44.12 | 41.41 | 34.35 |
| sp|Q93G07|CH60_LACAC | 60 kDa chaperonin OS=Lactobacillus acidophilus (strain ATCC 700396 / NCK56 / N2 / NCFM) GN=groL PE=3 SV=2 | 26 | 23 | 39.86 | 39.86 | 66.61 | 65.68 | 59.41 |
| tr|Q5FHQ7|Q5FHQ7_LACAC | Pyruvate oxidase OS=Lactobacillus acidophilus (strain ATCC 700396 / NCK56 / N2 / NCFM) GN=poxB PE=3 SV=1 | 22 | 21 | 37.68 | 37.68 | 58.40 | 50.58 | 43.76 |
| sp|Q5FKW9|THII_LACAC | Probable tRNA sulfurtransferase OS=Lactobacillus acidophilus (strain ATCC 700396 / NCK56 / N2 / NCFM) GN=thiI PE=3 SV=1 | 22 | 21 | 35.31 | 35.31 | 78.77 | 77.04 | 69.63 |
| tr|Q5FJI5|Q5FJI5_LACAC | Dihydroacetone kinase OS=Lactobacillus acidophilus (strain ATCC 700396 / NCK56 / N2 / NCFM) GN=LBA1310 PE=4 SV=1 | 20 | 16 | 31.41 | 31.41 | 49.91 | 47.74 | 44.48 |
| tr|Q5FME3|Q5FME3_LACAC | Dipeptidase OS=Lactobacillus acidophilus (strain ATCC 700396 / NCK56 / N2 / NCFM) GN=LBA0236 PE=4 SV=1 | 20 | 17 | 5.3 | 28.15 | 67.50 | 67.50 | 63.21 |
| sp|P35829|SLAP_LACAC | S-layer protein OS=Lactobacillus acidophilus (strain ATCC 700396 / NCK56 / N2 / NCFM) GN=slpA PE=1 SV=1 | 15 | 10 | 18.98 | 18.98 | 42.57 | 34.91 | 28.60 |
| tr|Q5FJG3|Q5FJG3_LACAC | Putative alkaline shock protein OS=Lactobacillus acidophilus (strain ATCC 700396 / NCK56 / N2 / NCFM) GN=LBA1334 PE=4 SV=1 | 15 | 11 | 12.96 | 12.96 | 79.17 | 79.17 | 79.17 |
| sp|Q5FL35|GLMM_LACAC | Phosphoglucosamine mutase OS=Lactobacillus acidophilus (strain ATCC 700396 / NCK56 / N2 / NCFM) GN=glmM PE=3 SV=1 | 13 | 9 | 15.93 | 15.93 | 33.63 | 30.09 | 28.54 |
| **sp|Q5FM93|RS7_LACAC** | **30S ribosomal protein S7 OS=Lactobacillus acidophilus (strain ATCC 700396 / NCK56 / N2 / NCFM) GN=rpsG PE=3 SV=1** | **13** | **9** | **0** | **15.72** | **70.51** | **64.10** | **59.62** |
| tr|Q5FHQ8|Q5FHQ8_LACAC | 6-phosphogluconate dehydrogenase, decarboxylating OS=Lactobacillus acidophilus (strain ATCC 700396 / NCK56 / N2 / NCFM) GN=LBA1973 PE=3 SV=1 | 13 | 9 | 15.57 | 15.57 | 41.76 | 35.12 | 32.55 |
| tr|Q5FKU6|Q5FKU6_LACAC | Cell-division initiation protein OS=Lactobacillus acidophilus (strain ATCC 700396 / NCK56 / N2 / NCFM) GN=LBA0816 PE=4 SV=1 | 10 | 8 | 10.68 | 10.68 | 41.95 | 36.70 | 32.58 |
| tr|Q5FMF6|Q5FMF6_LACAC | Cell separation protein OS=Lactobacillus acidophilus (strain ATCC 700396 / NCK56 / N2 / NCFM) GN=cdpA PE=4 SV=1 | 10 | 4 | 0 | 8.03 | 14.36 | 9.52 | 8.35 |
| tr|Q5FJI4|Q5FJI4_LACAC | Alkaline shock protein OS=Lactobacillus acidophilus (strain ATCC 700396 / NCK56 / N2 / NCFM) GN=LBA1311 PE=4 SV=1 | 10 | 4 | 2.41 | 2.41 | 51.67 | 51.67 | 33.33 |
| tr|F0TGI4|F0TGI4_LACA3 | Fructose-bisphosphate aldolase OS=Lactobacillus acidophilus (strain 30SC) GN=LAC30SC_08645 PE=4 SV=1 | 9 | 8 | 4 | 9.72 | 51.15 | 26.89 | 25.25 |
| tr|Q5FIG3|Q5FIG3_LACAC | Exodeoxyribonuclease OS=Lactobacillus acidophilus (strain ATCC 700396 / NCK56 / N2 / NCFM) GN=exoA PE=4 SV=1 | 8 | 5 | 7.04 | 7.04 | 45.85 | 41.50 | 24.11 |
| **sp|Q5FKX2|RS4_LACAC** | **30S ribosomal protein S4 OS=Lactobacillus acidophilus (strain ATCC 700396 / NCK56 / N2 / NCFM) GN=rpsD PE=3 SV=1** | **7** | **6** | **9.21** | **9.21** | **47.78** | **40.39** | **29.56** |
| **sp|Q5FMB6|RL31B_LACAC** | **50S ribosomal protein L31 type B OS=Lactobacillus acidophilus (strain ATCC 700396 / NCK56 / N2 / NCFM) GN=rpmE2 PE=3 SV=1** | **7** | **2** | **3.63** | **3.63** | **71.60** | **71.60** | **46.91** |
| **tr|F0TII1|F0TII1_LACA3** | **50S ribosomal protein L5 OS=Lactobacillus acidophilus (strain 30SC) GN=rplE PE=3 SV=1** | **6** | **5** | **0** | **8.99** | **52.22** | **42.22** | **29.44** |
| tr|Q5FMB1|Q5FMB1_LACAC | Putative uncharacterized protein OS=Lactobacillus acidophilus (strain ATCC 700396 / NCK56 / N2 / NCFM) GN=LBA0270 PE=4 SV=1 | 6 | 5 | 8.85 | 8.85 | 52.77 | 36.17 | 28.94 |
| **sp|Q5FM87|RL2_LACAC** | **50S ribosomal protein L2 OS=Lactobacillus acidophilus (strain ATCC 700396 / NCK56 / N2 / NCFM) GN=rplB PE=3 SV=1** | **5** | **3** | **4.7** | **4.71** | **24.46** | **15.83** | **15.83** |
| tr|Q5FIS9|Q5FIS9_LACAC | Putative serine protease OS=Lactobacillus acidophilus (strain ATCC 700396 / NCK56 / N2 / NCFM) GN=LBA1578 PE=4 SV=1 | 2 | 0 | 3.36 | 3.36 | 7.49 | 4.47 | 4.47 |
| sp|Q5FKD8|HSLU_LACAC | ATP-dependent protease ATPase subunit HslU OS=Lactobacillus acidophilus (strain ATCC 700396 / NCK56 / N2 / NCFM) GN=hslU PE=3 SV=1 | 2 | 0 | 1.32 | 1.32 | 7.72 | 4.29 | 4.29 |
| tr|Q5FLW1|Q5FLW1_LACAC | Thioredoxin OS=Lactobacillus acidophilus (strain ATCC 700396 / NCK56 / N2 / NCFM) GN=LBA0422 PE=3 SV=1 | 2 | 1 | 0.86 | 0.86 | 12.62 | 12.62 | 12.62 |
| tr|F0TGA2|F0TGA2_LACA3 | L-ldH OS=Lactobacillus acidophilus (strain 30SC) GN=LAC30SC_08225 PE=3 SV=1 | 1 | 0 | 2 | 2.00 | 20.72 | 8.22 | 8.22 |
| tr|Q5FL57|Q5FL57_LACAC | Putative gluconeogenesis factor OS=Lactobacillus acidophilus (strain ATCC 700396 / NCK56 / N2 / NCFM) GN=LBA0692 PE=3 SV=1 | 1 | 0 | 2 | 2.00 | 4.90 | 3.17 | 3.17 |
| sp|Q5FME6|PYRG_LACAC | CTP synthase OS=Lactobacillus acidophilus (strain ATCC 700396 / NCK56 / N2 / NCFM) GN=pyrG PE=3 SV=1 | 1 | 0 | 0 | 0.69 | 2.97 | 1.67 | 1.67 |
| **sp|Q5FM88|RL23_LACAC** | **50S ribosomal protein L23 OS=Lactobacillus acidophilus (strain ATCC 700396 / NCK56 / N2 / NCFM) GN=rplW PE=3 SV=1** | **1** | **0** | **0.62** | **0.62** | **40.00** | **21.00** | **10.00** |
| **sp|Q5FJK8|RL19_LACAC** | **50S ribosomal protein L19 OS=Lactobacillus acidophilus (strain ATCC 700396 / NCK56 / N2 / NCFM) GN=rplS PE=3 SV=1** | **1** | **0** | **0** | **0.15** | **43.48** | **36.52** | **9.57** |
| tr|Q5FMH5|Q5FMH5_LACAC | Aminopeptidase E OS=Lactobacillus acidophilus (strain ATCC 700396 / NCK56 / N2 / NCFM) GN=pepE PE=4 SV=1 | 1 | 0 | 0.12 | 0.13 | 8.68 | 2.28 | 2.28 |
| tr|F0TIA6|F0TIA6_LACA3 | HD superfamily phosphohydrolase OS=Lactobacillus acidophilus (strain 30SC) GN=LAC30SC_01105 PE=4 SV=1 | 1 | 0 | 0 | 0.10 | 2.64 | 2.64 | 2.64 |

**Supplementary Table S2.** Expression patterns of RICs by RNA-seq compared with Day 0 and Day 14 after His-ribosome induction. Differentially expressed genes between Day 0 and Day 14, and the results of GO enrichment analysis are shown.

Gene up

| GeneSymbol | description | log2FC | FDR |
| --- | --- | --- | --- |
| MAOB | monoamine oxidase B | 7.949028103 | 0 |
| EGFL6 | EGF-like-domain, multiple 6 | 6.893461378 | 0 |
| RPS4Y1 | ribosomal protein S4, Y-linked 1 | 6.886191238 | 0 |
| ITIH5 | inter-alpha-trypsin inhibitor heavy chain family, member 5 | 6.657431216 | 0 |
| FNDC1 | fibronectin type III domain containing 1 | 6.502932863 | 0 |
| DIO2 | deiodinase, iodothyronine, type II | 5.61955641 | 0 |
| SDK1 | sidekick homolog 1, cell adhesion molecule (chicken) | 5.007678875 | 0 |
| CH25H | cholesterol 25-hydroxylase | 5.003868313 | 0 |
| COL15A1 | collagen, type XV, alpha 1 | 4.830226961 | 0 |
| IL32 | interleukin 32 | 4.812635175 | 0 |
| TMEM176B | transmembrane protein 176B | 4.537110891 | 0 |
| TRIL | TLR4 interactor with leucine-rich repeats | 4.328410006 | 0 |
| POSTN | periostin, osteoblast specific factor | 4.25797033 | 0 |
| C3 | complement component 3 | 3.937406573 | 0 |
| ITGA11 | integrin, alpha 11 | 3.937313592 | 0 |
| STMN2 | stathmin-like 2 | 3.878811561 | 0 |
| IGFBP5 | insulin-like growth factor binding protein 5 | 3.781496944 | 0 |
| MT2A | metallothionein 2A | 3.766711668 | 0 |
| CILP | cartilage intermediate layer protein, nucleotide pyrophosphohydrolase | 3.714932929 | 0 |
| COMP | cartilage oligomeric matrix protein | 3.706581649 | 0 |
| AQP9 | aquaporin 9 | 3.676128199 | 0 |
| RASSF2 | Ras association (RalGDS/AF-6) domain family member 2 | 3.656841704 | 0 |
| PDPN | podoplanin | 3.603543161 | 0 |
| DPT | dermatopontin | 3.42834408 | 0 |
| PLXDC1 | plexin domain containing 1 | 3.41235356 | 0 |
| HLA-C | major histocompatibility complex, class I, C | 3.386190077 | 0 |
| CYP7B1 | cytochrome P450, family 7, subfamily B, polypeptide 1 | 3.195454178 | 0 |
| MAF | v-maf musculoaponeurotic fibrosarcoma oncogene homolog (avian) | 3.174484246 | 0 |
| SPON1 | spondin 1, extracellular matrix protein | 3.052737253 | 0 |
| MMP1 | matrix metallopeptidase 1 (interstitial collagenase) | 3.014576618 | 0 |
| HLA-A | major histocompatibility complex, class I, A | 2.78993185 | 0 |
| COL14A1 | collagen, type XIV, alpha 1 | 2.753380119 | 0 |
| LRRC15 | leucine rich repeat containing 15 | 2.732197884 | 0 |
| CFH | complement factor H | 2.722012508 | 0 |
| SLC39A8 | solute carrier family 39 (zinc transporter), member 8 | 2.606720281 | 0 |
| GJA1 | gap junction protein, alpha 1, 43kDa | 2.474254384 | 0 |
| FAM20A | family with sequence similarity 20, member A | 2.47354208 | 0 |
| IFI6 | interferon, alpha-inducible protein 6 | 2.432599441 | 0 |
| LOXL4 | lysyl oxidase-like 4 | 2.356753718 | 0 |
| BGN | biglycan | 2.204695917 | 0 |
| HLA-B | major histocompatibility complex, class I, B | 2.111063743 | 0 |
| TP53I11 | tumor protein p53 inducible protein 11 | 2.588242866 | 1.20433441830262E-319 |
| AQP1 | aquaporin 1 (Colton blood group) | 2.508468919 | 1.11648252490941E-311 |
| MGP | matrix Gla protein | 4.703857585 | 2.09364428290085E-311 |
| CFB | complement factor B | 2.090199729 | 3.50E-306 |
| BHLHE22 | basic helix-loop-helix family, member e22 | 4.719935123 | 3.22E-303 |
| CNTN1 | contactin 1 | 2.670545179 | 3.84E-302 |
| TIMP3 | TIMP metallopeptidase inhibitor 3 | 1.739064471 | 1.14E-301 |
| GAS7 | growth arrest-specific 7 | 2.665189328 | 3.05E-300 |
| OAS3 | 2'-5'-oligoadenylate synthetase 3, 100kDa | 2.885610426 | 7.88E-291 |
| DBC1 | deleted in bladder cancer 1 | 5.19329823 | 5.95E-290 |
| ADAMTS8 | ADAM metallopeptidase with thrombospondin type 1 motif, 8 | 3.489436588 | 2.23E-283 |
| CPE | carboxypeptidase E | 2.316955516 | 4.90E-282 |
| ELN | elastin | 2.130984178 | 4.71E-281 |
| AKR1C3 | aldo-keto reductase family 1, member C3 (3-alpha hydroxysteroid dehydrogenase, type II) | 2.031972373 | 6.26E-278 |
| GJB2 | gap junction protein, beta 2, 26kDa | 6.733845615 | 5.07E-269 |
| SEPP1 | selenoprotein P, plasma, 1 | 2.350569087 | 3.29E-263 |
| CACNA1G | calcium channel, voltage-dependent, T type, alpha 1G subunit | 3.4935169 | 3.98E-260 |
| GPR68 | G protein-coupled receptor 68 | 2.694925558 | 3.54E-259 |
| TMEM176A | transmembrane protein 176A | 4.285550169 | 4.61E-256 |
| SLC29A1 | solute carrier family 29 (nucleoside transporters), member 1 | 2.420189837 | 7.02E-252 |
| MXRA5 | matrix-remodelling associated 5 | 1.538808094 | 6.20E-249 |
| MT1E | metallothionein 1E | 2.635063347 | 5.28E-245 |
| NANOS1 | nanos homolog 1 (Drosophila) | 2.752645071 | 2.56E-244 |
| TNFSF10 | tumor necrosis factor (ligand) superfamily, member 10 | 4.04304394 | 3.30E-243 |
| CSGALNACT1 | chondroitin sulfate N-acetylgalactosaminyltransferase 1 | 4.081511195 | 8.76E-243 |
| LAMC3 | laminin, gamma 3 | 3.864589131 | 3.27E-242 |
| APOE | apolipoprotein E | 1.645829423 | 3.62E-238 |
| DUSP6 | dual specificity phosphatase 6 | 2.735945906 | 2.18E-235 |
| DDX3Y | DEAD (Asp-Glu-Ala-Asp) box polypeptide 3, Y-linked | 5.477968897 | 1.32E-232 |
| CXCL1 | chemokine (C-X-C motif) ligand 1 (melanoma growth stimulating activity, alpha) | 4.326845591 | 9.71E-231 |
| RARRES2 | retinoic acid receptor responder (tazarotene induced) 2 | 4.145880752 | 1.26E-229 |
| FIBIN | fin bud initiation factor homolog (zebrafish) | 1.84454517 | 1.29E-228 |
| CXCR7 | chemokine (C-X-C motif) receptor 7 | 2.795460693 | 2.79E-228 |
| EGR2 | early growth response 2 | 4.79162223 | 2.59E-227 |
| KIAA1324L | KIAA1324-like | 2.242451542 | 1.06E-225 |
| HR | hairless homolog (mouse) | 2.196586707 | 1.08E-222 |
| RGS4 | regulator of G-protein signaling 4 | 3.590619007 | 3.12E-222 |
| OLFML1 | olfactomedin-like 1 | 2.301247128 | 9.65E-220 |
| MOXD1 | monooxygenase, DBH-like 1 | 1.481432331 | 1.04E-219 |
| MAFB | v-maf musculoaponeurotic fibrosarcoma oncogene homolog B (avian) | 2.00077888 | 2.93E-219 |
| INHBB | inhibin, beta B | 2.807991886 | 1.86E-214 |
| PTGFRN | prostaglandin F2 receptor negative regulator | 1.869144693 | 1.06E-212 |
| SLC39A14 | solute carrier family 39 (zinc transporter), member 14 | 1.676266446 | 8.14E-211 |
| MX1 | myxovirus (influenza virus) resistance 1, interferon-inducible protein p78 (mouse) | 2.601694134 | 2.39E-208 |
| COL4A4 | collagen, type IV, alpha 4 | 5.071865811 | 1.21E-207 |
| TRIB2 | tribbles homolog 2 (Drosophila) | 2.888231761 | 1.76E-203 |
| UNC5B | unc-5 homolog B (C. elegans) | 2.024016449 | 3.90E-203 |
| OAS1 | 2'-5'-oligoadenylate synthetase 1, 40/46kDa | 4.559565456 | 3.27E-202 |
| PLXDC2 | plexin domain containing 2 | 6.237563664 | 1.10E-196 |
| APOD | apolipoprotein D | 1.628716667 | 6.28E-196 |
| ICAM1 | intercellular adhesion molecule 1 | 2.454434212 | 1.22E-195 |
| DDIT4 | DNA-damage-inducible transcript 4 | 1.747566394 | 1.25E-195 |
| IFI44L | interferon-induced protein 44-like | 3.991120658 | 5.86E-192 |
| HEPH | hephaestin | 2.196019262 | 2.38E-189 |
| PMEPA1 | prostate transmembrane protein, androgen induced 1 | 2.736980635 | 7.41E-188 |
| GFRA1 | GDNF family receptor alpha 1 | 4.808187384 | 7.11E-186 |
| PDGFRL | platelet-derived growth factor receptor-like | 2.056233921 | 2.26E-184 |
| FAM180B | family with sequence similarity 180, member B | 5.165510994 | 2.61E-184 |
| VCAM1 | vascular cell adhesion molecule 1 | 3.761105713 | 6.95E-183 |
| SH3PXD2B | SH3 and PX domains 2B | 1.480507377 | 1.29E-181 |
| TNFAIP6 | tumor necrosis factor, alpha-induced protein 6 | 2.592691652 | 5.55E-178 |
| TYMP | thymidine phosphorylase | 2.126355203 | 7.37E-178 |
| ANKH | ankylosis, progressive homolog (mouse) | 1.754444317 | 1.18E-177 |
| ADRA2A | adrenergic, alpha-2A-, receptor | 5.019239958 | 7.36E-177 |
| PSAT1 | phosphoserine aminotransferase 1 | 2.099419718 | 1.30E-174 |
| RGS3 | regulator of G-protein signaling 3 | 1.665729723 | 8.21E-173 |
| KHDRBS3 | KH domain containing, RNA binding, signal transduction associated 3 | 1.930011904 | 1.92E-168 |
| STON2 | stonin 2 | 2.348433109 | 1.92E-166 |
| CCL2 | chemokine (C-C motif) ligand 2 | 2.512847206 | 2.05E-166 |
| IGSF10 | immunoglobulin superfamily, member 10 | 1.951457313 | 3.78E-164 |
| ATP2A3 | ATPase, Ca++ transporting, ubiquitous | 2.816954088 | 5.54E-163 |
| OAS2 | 2'-5'-oligoadenylate synthetase 2, 69/71kDa | 2.241735685 | 1.34E-161 |
| ISM1 | isthmin 1 homolog (zebrafish) | 4.306074103 | 1.52E-161 |
| KDM5D | lysine (K)-specific demethylase 5D | 8.037957172 | 3.47E-161 |
| CHI3L1 | chitinase 3-like 1 (cartilage glycoprotein-39) | 1.633452333 | 1.61E-158 |
| CCDC80 | coiled-coil domain containing 80 | 1.246623863 | 2.42E-158 |
| TIMP1 | TIMP metallopeptidase inhibitor 1 | 1.253892448 | 1.93E-157 |
| MOB3B | MOB kinase activator 3B | 3.701102388 | 3.01E-157 |
| CYP26B1 | cytochrome P450, family 26, subfamily B, polypeptide 1 | 2.180571422 | 1.68E-156 |
| SLC40A1 | solute carrier family 40 (iron-regulated transporter), member 1 | 1.815257808 | 1.83E-156 |
| SLC43A2 | solute carrier family 43, member 2 | 2.622125425 | 3.34E-156 |
| IL16 | interleukin 16 | 2.471358956 | 1.15E-155 |
| MT1X | metallothionein 1X | 3.162148879 | 2.96E-154 |
| NCKAP5 | NCK-associated protein 5 | 4.047334422 | 3.23E-154 |
| FMOD | fibromodulin | 1.9741431 | 1.78E-152 |
| TNC | tenascin C | 1.193522937 | 5.55E-151 |
| RNF112 | ring finger protein 112 | 2.409757224 | 5.25E-150 |
| CXCL6 | chemokine (C-X-C motif) ligand 6 (granulocyte chemotactic protein 2) | 3.25611611 | 5.36E-150 |
| CDON | Cdon homolog (mouse) | 1.362455324 | 1.66E-148 |
| APOL4 | apolipoprotein L, 4 | 3.148552147 | 2.44E-147 |
| EGR1 | early growth response 1 | 1.566448044 | 1.89E-146 |
| RCAN2 | regulator of calcineurin 2 | 1.3970856 | 5.75E-145 |
| CCL8 | chemokine (C-C motif) ligand 8 | 6.163808662 | 1.67E-144 |
| AOX1 | aldehyde oxidase 1 | 1.340592415 | 1.77E-141 |
| WARS | tryptophanyl-tRNA synthetase | 1.520191683 | 4.00E-141 |
| ALDH1A1 | aldehyde dehydrogenase 1 family, member A1 | 4.113301671 | 5.21E-139 |
| TRPA1 | transient receptor potential cation channel, subfamily A, member 1 | 3.856788588 | 1.11E-138 |
| RASD1 | RAS, dexamethasone-induced 1 | 2.471464769 | 2.38E-138 |
| ITGB5 | integrin, beta 5 | 1.275756593 | 1.23E-137 |
| HLA-F | major histocompatibility complex, class I, F | 4.762942836 | 2.01E-137 |
| ROR2 | receptor tyrosine kinase-like orphan receptor 2 | 2.752250681 | 2.39E-137 |
| SGCD | sarcoglycan, delta (35kDa dystrophin-associated glycoprotein) | 1.498597933 | 4.68E-136 |
| C1QTNF1 | C1q and tumor necrosis factor related protein 1 | 1.496574681 | 4.89E-135 |
| CD74 | CD74 molecule, major histocompatibility complex, class II invariant chain | 2.902101834 | 5.02E-135 |
| SDK2 | sidekick homolog 2 (chicken) | 3.761190363 | 8.49E-134 |
| PHGDH | phosphoglycerate dehydrogenase | 1.527820391 | 1.50E-133 |
| XBP1 | X-box binding protein 1 | 1.358777182 | 1.39E-125 |
| OGN | osteoglycin | 2.978029176 | 8.06E-125 |
| LMO4 | LIM domain only 4 | 1.581034347 | 9.41E-125 |
| PI16 | peptidase inhibitor 16 | 7.109273229 | 1.28E-124 |
| SAT1 | spermidine/spermine N1-acetyltransferase 1 | 1.61373318 | 2.95E-123 |
| SOD2 | superoxide dismutase 2, mitochondrial | 1.074755975 | 1.09E-122 |
| PGF | placental growth factor | 1.750939552 | 2.90E-121 |
| HK2 | hexokinase 2 | 1.512180743 | 7.34E-121 |
| ASNS | asparagine synthetase (glutamine-hydrolyzing) | 1.920545978 | 7.52E-121 |
| LOC100506013 | uncharacterized LOC100506013 | 5.128804624 | 1.11E-120 |
| IFIT1 | interferon-induced protein with tetratricopeptide repeats 1 | 2.125313443 | 1.17E-120 |
| HSPA12B | heat shock 70kD protein 12B | 2.634035315 | 1.37E-120 |
| VIT | vitrin | 2.191638404 | 9.37E-119 |
| DCN | decorin | 1.141180346 | 1.35E-118 |
| MMP27 | matrix metallopeptidase 27 | 3.433073817 | 3.99E-118 |
| PODN | podocan | 1.208719313 | 3.01E-117 |
| VAV3 | vav 3 guanine nucleotide exchange factor | 4.193040426 | 6.78E-115 |
| LSP1 | lymphocyte-specific protein 1 | 1.953529219 | 2.48E-114 |
| TNFAIP2 | tumor necrosis factor, alpha-induced protein 2 | 1.206476395 | 2.76E-114 |
| SLC1A3 | solute carrier family 1 (glial high affinity glutamate transporter), member 3 | 1.39556685 | 3.14E-114 |
| GPR153 | G protein-coupled receptor 153 | 1.439348186 | 4.44E-114 |
| NLGN4Y | neuroligin 4, Y-linked | 7.681141294 | 2.89E-113 |
| SOX4 | SRY (sex determining region Y)-box 4 | 1.223077054 | 7.04E-113 |
| CDH23 | cadherin-related 23 | 4.405710796 | 1.43E-112 |
| SERPINE2 | serpin peptidase inhibitor, clade E (nexin, plasminogen activator inhibitor type 1), member 2 | 1.005756008 | 2.63E-112 |
| ADM2 | adrenomedullin 2 | 2.680105277 | 9.26E-111 |
| DPYSL3 | dihydropyrimidinase-like 3 | 1.110180122 | 1.93E-110 |
| AQPEP | laeverin | 3.272492672 | 7.35E-110 |
| C13orf33 | chromosome 13 open reading frame 33 | 1.304294201 | 3.90E-109 |
| RHOJ | ras homolog gene family, member J | 3.980405288 | 2.94E-108 |
| SLC38A5 | solute carrier family 38, member 5 | 2.171205439 | 1.34E-107 |
| NAMPT | nicotinamide phosphoribosyltransferase | 1.297214158 | 5.54E-107 |
| HTRA3 | HtrA serine peptidase 3 | 1.623352756 | 4.06E-106 |
| SLC3A2 | solute carrier family 3 (activators of dibasic and neutral amino acid transport), member 2 | 1.239923685 | 7.98E-105 |
| IPO5 | importin 5 | 1.040907687 | 2.39E-104 |
| XAF1 | XIAP associated factor 1 | 1.742633058 | 3.70E-104 |
| MMP2 | matrix metallopeptidase 2 (gelatinase A, 72kDa gelatinase, 72kDa type IV collagenase) | 1.115599599 | 4.27E-104 |
| SULF1 | sulfatase 1 | 1.627245909 | 2.60E-103 |
| STC1 | stanniocalcin 1 | 1.488558751 | 6.87E-103 |
| AKR1B1 | aldo-keto reductase family 1, member B1 (aldose reductase) | 1.154493381 | 2.48E-102 |
| ANGPTL1 | angiopoietin-like 1 | 2.595089722 | 4.58E-102 |
| SLC6A9 | solute carrier family 6 (neurotransmitter transporter, glycine), member 9 | 1.81100076 | 6.73E-101 |
| GUCY1A3 | guanylate cyclase 1, soluble, alpha 3 | 2.252788898 | 1.63E-100 |
| EMX2OS | EMX2 opposite strand/antisense RNA (non-protein coding) | 1.351022537 | 3.14E-100 |
| CFD | complement factor D (adipsin) | 1.143922765 | 2.00E-99 |
| HYOU1 | hypoxia up-regulated 1 | 1.247157663 | 6.95E-99 |
| TDO2 | tryptophan 2,3-dioxygenase | 2.952006576 | 8.02E-99 |
| CAPG | capping protein (actin filament), gelsolin-like | 1.256063206 | 8.40E-99 |
| USP9Y | ubiquitin specific peptidase 9, Y-linked | 6.044423887 | 1.37E-98 |
| BST2 | bone marrow stromal cell antigen 2 | 2.794675745 | 3.53E-98 |
| WTAP | Wilms tumor 1 associated protein | 1.147438431 | 1.13E-97 |
| COL6A6 | collagen, type VI, alpha 6 | 2.376741147 | 1.66E-97 |
| MTHFD2 | methylenetetrahydrofolate dehydrogenase (NADP+ dependent) 2, methenyltetrahydrofolate cyclohydrolase | 1.417964715 | 2.23E-97 |
| OLFML2A | olfactomedin-like 2A | 1.236454317 | 4.27E-97 |
| RGS16 | regulator of G-protein signaling 16 | 3.785536014 | 1.04E-96 |
| FBLN2 | fibulin 2 | 1.0259 | 5.17E-96 |
| SEMA3C | sema domain, immunoglobulin domain (Ig), short basic domain, secreted, (semaphorin) 3C | 1.148959453 | 7.59E-96 |
| IER3 | immediate early response 3 | 1.780678521 | 9.69E-96 |
| TWIST1 | twist homolog 1 (Drosophila) | 1.097629354 | 6.49E-95 |
| PLEKHG1 | pleckstrin homology domain containing, family G (with RhoGef domain) member 1 | 2.59843112 | 1.18E-94 |
| PRDX4 | peroxiredoxin 4 | 1.237093834 | 7.17E-94 |
| CD34 | CD34 molecule | 3.743434857 | 1.86E-93 |
| CNIH3 | cornichon homolog 3 (Drosophila) | 1.685846893 | 3.32E-93 |
| MEGF6 | multiple EGF-like-domains 6 | 1.502745285 | 3.47E-92 |
| OMD | osteomodulin | 3.409646024 | 6.82E-92 |
| PCK2 | phosphoenolpyruvate carboxykinase 2 (mitochondrial) | 1.540104515 | 1.66E-91 |
| FAM3C | family with sequence similarity 3, member C | 1.162416898 | 3.77E-91 |
| CPZ | carboxypeptidase Z | 1.233944595 | 2.79E-89 |
| PLXNA4 | plexin A4 | 2.150306247 | 4.62E-89 |
| PRRX2 | paired related homeobox 2 | 1.278390688 | 8.19E-89 |
| POU2F2 | POU class 2 homeobox 2 | 1.862832508 | 8.80E-89 |
| PREX2 | phosphatidylinositol-3,4,5-trisphosphate-dependent Rac exchange factor 2 | 3.326844176 | 9.51E-89 |
| RELB | v-rel reticuloendotheliosis viral oncogene homolog B | 1.711802446 | 3.08E-88 |
| IL34 | interleukin 34 | 2.289310601 | 1.29E-87 |
| BIRC3 | baculoviral IAP repeat containing 3 | 3.202974957 | 2.92E-87 |
| IFITM1 | interferon induced transmembrane protein 1 (9-27) | 1.08429471 | 4.30E-87 |
| ADAMDEC1 | ADAM-like, decysin 1 | 10.68580454 | 2.07E-86 |
| OLFML2B | olfactomedin-like 2B | 1.085424873 | 2.29E-86 |
| TMEM132A | transmembrane protein 132A | 1.514502745 | 3.61E-86 |
| CLIC2 | chloride intracellular channel 2 | 1.981145284 | 7.73E-86 |
| CGNL1 | cingulin-like 1 | 3.212151685 | 2.27E-85 |
| SUN2 | Sad1 and UNC84 domain containing 2 | 1.238688 | 4.67E-85 |
| PPFIBP2 | PTPRF interacting protein, binding protein 2 (liprin beta 2) | 1.980773398 | 6.92E-85 |
| GAS1 | growth arrest-specific 1 | 1.048675118 | 7.79E-85 |
| SHMT2 | serine hydroxymethyltransferase 2 (mitochondrial) | 1.085736066 | 3.09E-83 |
| MARS | methionyl-tRNA synthetase | 1.144584776 | 3.24E-83 |
| HERC2P2 | hect domain and RLD 2 pseudogene 2 | 1.599677295 | 6.43E-83 |
| RSAD2 | radical S-adenosyl methionine domain containing 2 | 4.007918911 | 7.71E-83 |
| PLAC9 | placenta-specific 9 | 1.500714761 | 1.01E-81 |
| PLK2 | polo-like kinase 2 | 1.135463911 | 2.03E-81 |
| MSC | musculin | 1.774949323 | 4.12E-81 |
| KIAA0226L | KIAA0226-like | 1.674324041 | 8.76E-81 |
| PRKY | protein kinase, Y-linked, pseudogene | 7.512015327 | 1.11E-80 |
| ACVR2A | activin A receptor, type IIA | 1.582496291 | 1.18E-80 |
| ZSWIM4 | zinc finger, SWIM-type containing 4 | 1.984031167 | 1.67E-80 |
| EDNRB | endothelin receptor type B | 2.358657325 | 2.84E-80 |
| IFI44 | interferon-induced protein 44 | 1.800840391 | 6.33E-80 |
| KIAA1462 | KIAA1462 | 1.319787885 | 1.99E-79 |
| CMKLR1 | chemokine-like receptor 1 | 1.779812134 | 1.10E-78 |
| BTN3A2 | butyrophilin, subfamily 3, member A2 | 1.437573945 | 1.27E-78 |
| FAM19A5 | family with sequence similarity 19 (chemokine (C-C motif)-like), member A5 | 6.972150527 | 1.43E-78 |
| SLC5A3 | solute carrier family 5 (sodium/myo-inositol cotransporter), member 3 | 1.114234228 | 1.79E-78 |
| STMN3 | stathmin-like 3 | 1.701739883 | 1.26E-77 |
| SLIT2 | slit homolog 2 (Drosophila) | 1.090152979 | 2.53E-77 |
| ETV5 | ets variant 5 | 1.483152274 | 3.12E-77 |
| TMEM158 | transmembrane protein 158 (gene/pseudogene) | 1.26705573 | 1.04E-76 |
| LMO2 | LIM domain only 2 (rhombotin-like 1) | 3.994650974 | 2.03E-76 |
| ECM2 | extracellular matrix protein 2, female organ and adipocyte specific | 1.283187411 | 3.78E-76 |
| ABI3BP | ABI family, member 3 (NESH) binding protein | 1.116588594 | 1.22E-75 |
| HS3ST6 | heparan sulfate (glucosamine) 3-O-sulfotransferase 6 | 7.070066809 | 7.81E-75 |
| JUNB | jun B proto-oncogene | 1.125219797 | 5.27E-74 |
| UNKL | unkempt homolog (Drosophila)-like | 1.245657373 | 6.26E-73 |
| TSPAN11 | tetraspanin 11 | 1.91123342 | 8.96E-72 |
| SLC15A3 | solute carrier family 15, member 3 | 1.533327279 | 1.48E-71 |
| EMID1 | EMI domain containing 1 | 4.935115864 | 4.14E-71 |
| LSAMP | limbic system-associated membrane protein | 1.260266039 | 4.94E-71 |
| SLC1A4 | solute carrier family 1 (glutamate/neutral amino acid transporter), member 4 | 2.055874375 | 2.32E-70 |
| FER1L6 | fer-1-like 6 (C. elegans) | 1.931239143 | 5.69E-70 |
| EFR3B | EFR3 homolog B (S. cerevisiae) | 3.797411658 | 6.79E-69 |
| NFKBIA | nuclear factor of kappa light polypeptide gene enhancer in B-cells inhibitor, alpha | 1.201845995 | 1.15E-68 |
| CDKN2B | cyclin-dependent kinase inhibitor 2B (p15, inhibits CDK4) | 1.276955065 | 3.62E-68 |
| PTN | pleiotrophin | 1.302475235 | 3.93E-68 |
| APOL1 | apolipoprotein L, 1 | 1.221949894 | 5.13E-68 |
| LRIG1 | leucine-rich repeats and immunoglobulin-like domains 1 | 1.011489806 | 5.99E-68 |
| DBP | D site of albumin promoter (albumin D-box) binding protein | 1.809601412 | 7.73E-68 |
| NCALD | neurocalcin delta | 1.765202568 | 1.07E-67 |
| SIPA1L2 | signal-induced proliferation-associated 1 like 2 | 1.285244736 | 1.10E-67 |
| IL20RB | interleukin 20 receptor beta | 1.352204965 | 1.67E-67 |
| SRPRB | signal recognition particle receptor, B subunit | 1.169902359 | 2.46E-67 |
| DNAJB9 | DnaJ (Hsp40) homolog, subfamily B, member 9 | 1.409538652 | 3.24E-67 |
| KIF26A | kinesin family member 26A | 4.235458022 | 6.46E-67 |
| TUSC1 | tumor suppressor candidate 1 | 1.479378271 | 7.65E-67 |
| COL8A2 | collagen, type VIII, alpha 2 | 1.492098489 | 1.48E-66 |
| RGS2 | regulator of G-protein signaling 2, 24kDa | 1.9544213 | 3.57E-66 |
| CCL7 | chemokine (C-C motif) ligand 7 | 7.396716018 | 4.87E-66 |
| ALDH1A2 | aldehyde dehydrogenase 1 family, member A2 | 3.069596214 | 5.93E-66 |
| ARRDC3 | arrestin domain containing 3 | 1.127096147 | 8.61E-66 |
| GBP3 | guanylate binding protein 3 | 1.472725211 | 1.66E-65 |
| HSD11B2 | hydroxysteroid (11-beta) dehydrogenase 2 | 4.570497093 | 2.22E-65 |
| SETBP1 | SET binding protein 1 | 1.157168712 | 3.49E-65 |
| LDB2 | LIM domain binding 2 | 1.440663826 | 5.56E-64 |
| PHEX | phosphate regulating endopeptidase homolog, X-linked | 3.053087307 | 7.80E-64 |
| FOXO1 | forkhead box O1 | 1.984352072 | 9.41E-64 |
| KRT10 | keratin 10 | 1.452752813 | 7.02E-63 |
| RORA | RAR-related orphan receptor A | 1.814547509 | 8.40E-63 |
| MT1M | metallothionein 1M | 2.816289292 | 8.71E-63 |
| TGFB3 | transforming growth factor, beta 3 | 1.566976597 | 1.37E-62 |
| CD97 | CD97 molecule | 1.070141667 | 1.77E-62 |
| HAO2 | hydroxyacid oxidase 2 (long chain) | 7.291616055 | 2.25E-62 |
| CTSS | cathepsin S | 1.80673246 | 2.47E-62 |
| TCEA1 | transcription elongation factor A (SII), 1 | 1.047783148 | 1.04E-61 |
| EML5 | echinoderm microtubule associated protein like 5 | 3.759696855 | 1.12E-61 |
| PDLIM4 | PDZ and LIM domain 4 | 1.262486293 | 2.96E-59 |
| RGMA | RGM domain family, member A | 1.352147432 | 3.40E-59 |
| DCLK1 | doublecortin-like kinase 1 | 1.303370608 | 8.57E-59 |
| TAP1 | transporter 1, ATP-binding cassette, sub-family B (MDR/TAP) | 1.173296169 | 8.59E-59 |
| GAP43 | growth associated protein 43 | 4.400245135 | 1.26E-58 |
| UBL3 | ubiquitin-like 3 | 1.126696234 | 3.07E-58 |
| FAM19A2 | family with sequence similarity 19 (chemokine (C-C motif)-like), member A2 | 2.795833064 | 4.04E-58 |
| LIMCH1 | LIM and calponin homology domains 1 | 2.777065779 | 1.46E-57 |
| ZFY | zinc finger protein, Y-linked | 7.865032669 | 8.19E-57 |
| RIN2 | Ras and Rab interactor 2 | 1.015340405 | 2.57E-56 |
| RSPO3 | R-spondin 3 | 1.138053212 | 2.88E-56 |
| GBP5 | guanylate binding protein 5 | 4.261090312 | 2.93E-56 |
| IGF1 | insulin-like growth factor 1 (somatomedin C) | 4.994811218 | 9.29E-56 |
| IRX1 | iroquois homeobox 1 | 1.635888752 | 1.10E-55 |
| SERPINI1 | serpin peptidase inhibitor, clade I (neuroserpin), member 1 | 2.66744074 | 1.86E-55 |
| GBP4 | guanylate binding protein 4 | 4.097502853 | 1.97E-55 |
| L1CAM | L1 cell adhesion molecule | 2.819552476 | 2.44E-55 |
| S100A16 | S100 calcium binding protein A16 | 1.028910331 | 3.01E-55 |
| ISG15 | ISG15 ubiquitin-like modifier | 1.483588374 | 3.49E-55 |
| LGALS9 | lectin, galactoside-binding, soluble, 9 | 2.261011107 | 4.77E-55 |
| MKX | mohawk homeobox | 1.823305221 | 1.74E-54 |
| IRF1 | interferon regulatory factor 1 | 1.21991552 | 1.79E-54 |
| TXLNG2P | taxilin gamma 2, pseudogene | 9.891215156 | 2.13E-54 |
| PEX5L | peroxisomal biogenesis factor 5-like | 3.805261837 | 2.25E-54 |
| PSME2 | proteasome (prosome, macropain) activator subunit 2 (PA28 beta) | 1.057503536 | 4.99E-54 |
| SLC12A7 | solute carrier family 12 (potassium/chloride transporters), member 7 | 1.382221316 | 8.63E-54 |
| CHAC1 | ChaC, cation transport regulator homolog 1 (E. coli) | 2.356466899 | 1.04E-53 |
| SUSD5 | sushi domain containing 5 | 1.960093716 | 1.18E-53 |
| TNFRSF12A | tumor necrosis factor receptor superfamily, member 12A | 1.076846438 | 1.42E-53 |
| SCARA5 | scavenger receptor class A, member 5 (putative) | 4.757707941 | 2.08E-53 |
| RPA2 | replication protein A2, 32kDa | 1.342767684 | 7.35E-53 |
| PDE1A | phosphodiesterase 1A, calmodulin-dependent | 3.616168481 | 9.67E-53 |
| LPL | lipoprotein lipase | 7.739151129 | 1.46E-52 |
| SEPT6 | septin 6 | 1.505484494 | 3.22E-52 |
| FMNL1 | formin-like 1 | 1.406413449 | 5.47E-52 |
| PRICKLE1 | prickle homolog 1 (Drosophila) | 2.219604546 | 8.36E-52 |
| HCK | hemopoietic cell kinase | 7.764159479 | 1.04E-51 |
| AGTRAP | angiotensin II receptor-associated protein | 1.159622071 | 1.23E-51 |
| PSMB9 | proteasome (prosome, macropain) subunit, beta type, 9 (large multifunctional peptidase 2) | 1.406778619 | 1.71E-51 |
| PYCR1 | pyrroline-5-carboxylate reductase 1 | 1.179043488 | 2.03E-51 |
| NFAM1 | NFAT activating protein with ITAM motif 1 | 6.873123879 | 4.54E-51 |
| GSTM3 | glutathione S-transferase mu 3 (brain) | 1.264570973 | 6.02E-51 |
| SAMD9L | sterile alpha motif domain containing 9-like | 1.120497266 | 9.94E-51 |
| GDF10 | growth differentiation factor 10 | 5.759756733 | 1.13E-50 |
| TP63 | tumor protein p63 | 3.004280821 | 2.41E-50 |
| IFIH1 | interferon induced with helicase C domain 1 | 1.586563536 | 5.06E-50 |
| FGL2 | fibrinogen-like 2 | 3.530438399 | 7.88E-50 |
| APLN | apelin | 1.376429008 | 9.52E-50 |
| IGFBP2 | insulin-like growth factor binding protein 2, 36kDa | 1.446676724 | 1.26E-49 |
| HUNK | hormonally up-regulated Neu-associated kinase | 1.88370913 | 1.38E-49 |
| KIAA1755 | KIAA1755 | 2.492254205 | 3.25E-49 |
| AVPR1A | arginine vasopressin receptor 1A | 5.360637623 | 3.41E-49 |
| MRPS6 | mitochondrial ribosomal protein S6 | 1.103827949 | 7.12E-49 |
| IL4I1 | interleukin 4 induced 1 | 1.957653117 | 9.45E-49 |
| IL6 | interleukin 6 (interferon, beta 2) | 2.916453904 | 1.06E-48 |
| SYT7 | synaptotagmin VII | 1.441516864 | 2.40E-48 |
| GTF3A | general transcription factor IIIA | 1.017601276 | 3.92E-48 |
| SYBU | syntabulin (syntaxin-interacting) | 1.791503482 | 4.65E-48 |
| IFI27 | interferon, alpha-inducible protein 27 | 1.9681265 | 5.73E-48 |
| JAM2 | junctional adhesion molecule 2 | 1.700509901 | 1.14E-47 |
| UPP1 | uridine phosphorylase 1 | 1.488410811 | 1.99E-47 |
| PHKG1 | phosphorylase kinase, gamma 1 (muscle) | 1.648082944 | 3.41E-47 |
| SNCAIP | synuclein, alpha interacting protein | 2.751319534 | 8.80E-47 |
| MRPL17 | mitochondrial ribosomal protein L17 | 1.147136253 | 9.06E-47 |
| TNFSF13B | tumor necrosis factor (ligand) superfamily, member 13b | 2.089356684 | 3.13E-46 |
| EMX2 | empty spiracles homeobox 2 | 1.152087117 | 3.30E-46 |
| JAK3 | Janus kinase 3 | 1.840982308 | 3.45E-46 |
| FABP5 | fatty acid binding protein 5 (psoriasis-associated) | 1.653082611 | 1.10E-45 |
| QPCT | glutaminyl-peptide cyclotransferase | 1.22899822 | 1.35E-45 |
| CMPK2 | cytidine monophosphate (UMP-CMP) kinase 2, mitochondrial | 3.486263393 | 1.43E-45 |
| FAM107B | family with sequence similarity 107, member B | 1.480861645 | 3.24E-45 |
| CLIC6 | chloride intracellular channel 6 | 3.858110638 | 1.74E-44 |
| DPYSL4 | dihydropyrimidinase-like 4 | 4.103454501 | 2.79E-44 |
| RASL12 | RAS-like, family 12 | 1.204440417 | 2.85E-44 |
| BHLHE41 | basic helix-loop-helix family, member e41 | 1.876808714 | 4.06E-44 |
| MCTP2 | multiple C2 domains, transmembrane 2 | 1.721651665 | 6.22E-44 |
| NDP | Norrie disease (pseudoglioma) | 3.625830709 | 7.45E-44 |
| IFNGR2 | interferon gamma receptor 2 (interferon gamma transducer 1) | 1.009078452 | 1.83E-43 |
| MEOX1 | mesenchyme homeobox 1 | 5.11847756 | 2.55E-43 |
| ZHX2 | zinc fingers and homeoboxes 2 | 1.179489959 | 2.89E-43 |
| COL23A1 | collagen, type XXIII, alpha 1 | 3.452001725 | 3.51E-43 |
| ADCYAP1R1 | adenylate cyclase activating polypeptide 1 (pituitary) receptor type I | 3.983077216 | 4.20E-43 |
| UST | uronyl-2-sulfotransferase | 1.002654412 | 4.44E-43 |
| SLC2A1 | solute carrier family 2 (facilitated glucose transporter), member 1 | 1.301438818 | 1.37E-42 |
| FAS | Fas (TNF receptor superfamily, member 6) | 1.030689964 | 1.64E-42 |
| BACE2 | beta-site APP-cleaving enzyme 2 | 1.044237223 | 4.16E-42 |
| SLCO4A1 | solute carrier organic anion transporter family, member 4A1 | 3.845731797 | 4.88E-42 |
| DUSP1 | dual specificity phosphatase 1 | 1.00694939 | 8.64E-42 |
| NDUFA4L2 | NADH dehydrogenase (ubiquinone) 1 alpha subcomplex, 4-like 2 | 1.740686011 | 9.79E-42 |
| TNFRSF1B | tumor necrosis factor receptor superfamily, member 1B | 1.184306281 | 1.14E-41 |
| ETV1 | ets variant 1 | 1.241518753 | 1.16E-41 |
| TRPV2 | transient receptor potential cation channel, subfamily V, member 2 | 1.335622589 | 1.42E-41 |
| HERC5 | hect domain and RLD 5 | 2.670347598 | 2.20E-41 |
| IL8 | interleukin 8 | 3.60400307 | 2.63E-41 |
| PCDH7 | protocadherin 7 | 1.490586751 | 3.14E-41 |
| KCNN4 | potassium intermediate/small conductance calcium-activated channel, subfamily N, member 4 | 4.025428476 | 3.14E-41 |
| PARP12 | poly (ADP-ribose) polymerase family, member 12 | 1.412080191 | 3.56E-40 |
| C2 | complement component 2 | 1.123977335 | 4.91E-40 |
| KAZALD1 | Kazal-type serine peptidase inhibitor domain 1 | 1.256835933 | 6.63E-40 |
| EGLN3 | egl nine homolog 3 (C. elegans) | 4.858402161 | 7.60E-40 |
| HSD17B10 | hydroxysteroid (17-beta) dehydrogenase 10 | 1.097327609 | 8.58E-40 |
| ST6GALNAC5 | ST6 (alpha-N-acetyl-neuraminyl-2,3-beta-galactosyl-1,3)-N-acetylgalactosaminide alpha-2,6-sialyltransferase 5 | 1.624502076 | 3.00E-39 |
| TMEM205 | transmembrane protein 205 | 1.076917672 | 4.01E-39 |
| ISOC2 | isochorismatase domain containing 2 | 1.021965547 | 4.06E-39 |
| NFKBIE | nuclear factor of kappa light polypeptide gene enhancer in B-cells inhibitor, epsilon | 1.590685144 | 6.83E-39 |
| MSRA | methionine sulfoxide reductase A | 1.611006468 | 1.25E-38 |
| SORCS2 | sortilin-related VPS10 domain containing receptor 2 | 2.311348987 | 1.30E-38 |
| IMP3 | IMP3, U3 small nucleolar ribonucleoprotein, homolog (yeast) | 1.087400186 | 1.62E-38 |
| ZC3H12A | zinc finger CCCH-type containing 12A | 1.392312449 | 3.08E-38 |
| IRX5 | iroquois homeobox 5 | 1.614412672 | 3.50E-38 |
| FNBP1L | formin binding protein 1-like | 1.399437775 | 3.65E-38 |
| RNF150 | ring finger protein 150 | 1.921745269 | 4.57E-38 |
| CTSH | cathepsin H | 1.876196705 | 6.41E-38 |
| PCDHGB6 | protocadherin gamma subfamily B, 6 | 1.749436723 | 7.91E-38 |
| CDK2AP2 | cyclin-dependent kinase 2 associated protein 2 | 1.354437928 | 1.08E-37 |
| TNFAIP3 | tumor necrosis factor, alpha-induced protein 3 | 1.683309897 | 1.31E-37 |
| ENPP4 | ectonucleotide pyrophosphatase/phosphodiesterase 4 (putative) | 1.943284903 | 1.70E-37 |
| PRKAG3 | protein kinase, AMP-activated, gamma 3 non-catalytic subunit | 5.625106451 | 5.02E-37 |
| USP32P2 | ubiquitin specific peptidase 32 pseudogene 2 | 3.274769909 | 2.06E-36 |
| IRF7 | interferon regulatory factor 7 | 1.395425779 | 2.24E-36 |
| TSHZ3 | teashirt zinc finger homeobox 3 | 1.012768313 | 2.45E-36 |
| RRAGD | Ras-related GTP binding D | 2.577655851 | 3.06E-36 |
| PSG1 | pregnancy specific beta-1-glycoprotein 1 | 1.931132345 | 5.82E-36 |
| SLC7A5 | solute carrier family 7 (amino acid transporter light chain, L system), member 5 | 1.050329644 | 1.17E-35 |
| IRX6 | iroquois homeobox 6 | 5.86835133 | 2.02E-35 |
| MSX1 | msh homeobox 1 | 1.054292311 | 2.63E-35 |
| UTY | ubiquitously transcribed tetratricopeptide repeat gene, Y-linked | 5.57574788 | 9.82E-35 |
| PITX1 | paired-like homeodomain 1 | 4.828632514 | 1.27E-34 |
| BPIFB4 | BPI fold containing family B, member 4 | 5.765501115 | 1.54E-34 |
| BCAS1 | breast carcinoma amplified sequence 1 | 1.638522604 | 1.56E-34 |
| FAM43B | family with sequence similarity 43, member B | 5.535278751 | 1.98E-34 |
| CEBPG | CCAAT/enhancer binding protein (C/EBP), gamma | 1.055035122 | 2.26E-34 |
| HES1 | hairy and enhancer of split 1, (Drosophila) | 3.292119868 | 5.55E-34 |
| EIF4EBP1 | eukaryotic translation initiation factor 4E binding protein 1 | 1.01282696 | 5.80E-34 |
| CCDC48 | coiled-coil domain containing 48 | 9.195814394 | 6.17E-34 |
| MAGEH1 | melanoma antigen family H, 1 | 1.027731016 | 8.50E-34 |
| IL15RA | interleukin 15 receptor, alpha | 1.253507022 | 9.62E-34 |
| MMP10 | matrix metallopeptidase 10 (stromelysin 2) | 6.202533189 | 1.08E-33 |
| GGT5 | gamma-glutamyltransferase 5 | 1.617761586 | 1.14E-33 |
| IL7 | interleukin 7 | 1.888995669 | 1.43E-33 |
| JHDM1D | jumonji C domain containing histone demethylase 1 homolog D (S. cerevisiae) | 1.098112136 | 3.30E-33 |
| LFNG | LFNG O-fucosylpeptide 3-beta-N-acetylglucosaminyltransferase | 1.258177052 | 4.21E-33 |
| ASTN1 | astrotactin 1 | 2.22410136 | 4.31E-33 |
| F13A1 | coagulation factor XIII, A1 polypeptide | 9.08667117 | 6.74E-33 |
| MX2 | myxovirus (influenza virus) resistance 2 (mouse) | 1.281758211 | 1.01E-32 |
| ADAM23 | ADAM metallopeptidase domain 23 | 1.488453979 | 1.06E-32 |
| ADAM22 | ADAM metallopeptidase domain 22 | 1.7665283 | 1.16E-32 |
| HERC6 | hect domain and RLD 6 | 1.365111733 | 1.72E-32 |
| ANKRD35 | ankyrin repeat domain 35 | 1.258260692 | 1.91E-32 |
| CXCL10 | chemokine (C-X-C motif) ligand 10 | 9.017826297 | 2.52E-32 |
| DDX58 | DEAD (Asp-Glu-Ala-Asp) box polypeptide 58 | 1.09402967 | 3.41E-32 |
| SEMA4D | sema domain, immunoglobulin domain (Ig), transmembrane domain (TM) and short cytoplasmic domain, (semaphorin) 4D | 2.243856925 | 5.07E-32 |
| DKK2 | dickkopf 2 homolog (Xenopus laevis) | 1.266003669 | 5.93E-32 |
| CMAHP | cytidine monophospho-N-acetylneuraminic acid hydroxylase, pseudogene | 1.909095886 | 1.51E-31 |
| PCSK1 | proprotein convertase subtilisin/kexin type 1 | 2.269490895 | 2.38E-31 |
| DOK6 | docking protein 6 | 1.430238133 | 4.03E-31 |
| PIK3R3 | phosphoinositide-3-kinase, regulatory subunit 3 (gamma) | 1.504170718 | 4.87E-31 |
| RFTN2 | raftlin family member 2 | 1.532247674 | 1.07E-30 |
| TRIB3 | tribbles homolog 3 (Drosophila) | 1.061080077 | 1.24E-30 |
| TSEN15 | tRNA splicing endonuclease 15 homolog (S. cerevisiae) | 1.163488601 | 1.43E-30 |
| SERTAD1 | SERTA domain containing 1 | 1.174347205 | 1.46E-30 |
| ZBTB46 | zinc finger and BTB domain containing 46 | 2.359695096 | 1.46E-30 |
| TLR4 | toll-like receptor 4 | 1.033947044 | 1.86E-30 |
| SEL1L3 | sel-1 suppressor of lin-12-like 3 (C. elegans) | 1.886908833 | 1.97E-30 |
| SDF2L1 | stromal cell-derived factor 2-like 1 | 1.2789177 | 2.21E-30 |
| C15orf59 | chromosome 15 open reading frame 59 | 1.938677347 | 2.40E-30 |
| ALPL | alkaline phosphatase, liver/bone/kidney | 2.067623247 | 3.09E-30 |
| SH3BP5 | SH3-domain binding protein 5 (BTK-associated) | 1.178158834 | 3.58E-30 |
| LOC100134229 | uncharacterized LOC100134229 | 1.932089343 | 5.15E-30 |
| CLCN4 | chloride channel 4 | 1.366172165 | 5.66E-30 |
| MEX3B | mex-3 homolog B (C. elegans) | 1.208416296 | 6.26E-30 |
| DTX4 | deltex homolog 4 (Drosophila) | 1.575740728 | 6.35E-30 |
| CIAPIN1 | cytokine induced apoptosis inhibitor 1 | 1.012282424 | 6.87E-30 |
| USP18 | ubiquitin specific peptidase 18 | 1.812033785 | 7.79E-30 |
| GMPPB | GDP-mannose pyrophosphorylase B | 1.251302569 | 7.98E-30 |
| LAPTM5 | lysosomal protein transmembrane 5 | 4.34191314 | 9.46E-30 |
| PCDH20 | protocadherin 20 | 5.274480106 | 1.31E-29 |
| MT1F | metallothionein 1F | 5.381301136 | 1.40E-29 |
| LOC401463 | uncharacterized LOC401463 | 3.158699337 | 1.55E-29 |
| MLXIPL | MLX interacting protein-like | 3.693849434 | 3.00E-29 |
| IL17RB | interleukin 17 receptor B | 1.554499372 | 4.21E-29 |
| FAM117A | family with sequence similarity 117, member A | 1.795085118 | 6.52E-29 |
| SPRY1 | sprouty homolog 1, antagonist of FGF signaling (Drosophila) | 1.106126419 | 1.02E-28 |
| LGI2 | leucine-rich repeat LGI family, member 2 | 4.544345573 | 1.29E-28 |
| C3orf39 | chromosome 3 open reading frame 39 | 1.346007291 | 2.34E-28 |
| LOC283070 | uncharacterized LOC283070 | 2.778380987 | 2.49E-28 |
| CYYR1 | cysteine/tyrosine-rich 1 | 1.270421549 | 3.03E-28 |
| CAP2 | CAP, adenylate cyclase-associated protein, 2 (yeast) | 2.232424909 | 4.98E-28 |
| IKBKE | inhibitor of kappa light polypeptide gene enhancer in B-cells, kinase epsilon | 1.171918763 | 5.34E-28 |
| TLR3 | toll-like receptor 3 | 2.009375665 | 1.40E-27 |
| GFRA2 | GDNF family receptor alpha 2 | 3.088102194 | 1.67E-27 |
| ABCA3 | ATP-binding cassette, sub-family A (ABC1), member 3 | 2.683740702 | 1.79E-27 |
| ST8SIA1 | ST8 alpha-N-acetyl-neuraminide alpha-2,8-sialyltransferase 1 | 1.142604984 | 2.04E-27 |
| SORT1 | sortilin 1 | 1.061948048 | 2.69E-27 |
| FXYD6 | FXYD domain containing ion transport regulator 6 | 4.667894723 | 3.78E-27 |
| GPX7 | glutathione peroxidase 7 | 1.137983708 | 5.26E-27 |
| PLA2G4C | phospholipase A2, group IVC (cytosolic, calcium-independent) | 1.624052554 | 5.49E-27 |
| CCPG1 | cell cycle progression 1 | 1.109679395 | 8.01E-27 |
| CCDC167 | coiled-coil domain containing 167 | 1.50388981 | 1.03E-26 |
| PRRG3 | proline rich Gla (G-carboxyglutamic acid) 3 (transmembrane) | 3.54083361 | 2.27E-26 |
| LARGE | like-glycosyltransferase | 1.064565662 | 2.29E-26 |
| TMEFF2 | transmembrane protein with EGF-like and two follistatin-like domains 2 | 2.990664112 | 2.77E-26 |
| TMEM100 | transmembrane protein 100 | 2.140278062 | 3.19E-26 |
| C13orf15 | chromosome 13 open reading frame 15 | 1.088384309 | 3.63E-26 |
| THSD7A | thrombospondin, type I, domain containing 7A | 2.147212996 | 4.28E-26 |
| ROBO2 | roundabout, axon guidance receptor, homolog 2 (Drosophila) | 1.114011216 | 4.66E-26 |
| ADAMTS10 | ADAM metallopeptidase with thrombospondin type 1 motif, 10 | 1.021296781 | 5.93E-26 |
| ELANE | elastase, neutrophil expressed | 2.215129839 | 6.51E-26 |
| KY | kyphoscoliosis peptidase | 3.097712331 | 6.99E-26 |
| KCND3 | potassium voltage-gated channel, Shal-related subfamily, member 3 | 2.471742456 | 2.07E-25 |
| LOC253039 | uncharacterized LOC253039 | 1.271555449 | 2.52E-25 |
| TAPBPL | TAP binding protein-like | 1.054378922 | 2.82E-25 |
| OLFM1 | olfactomedin 1 | 1.427150845 | 3.15E-25 |
| BDKRB1 | bradykinin receptor B1 | 1.488953305 | 3.91E-25 |
| SCUBE3 | signal peptide, CUB domain, EGF-like 3 | 1.383420648 | 4.71E-25 |
| STOML1 | stomatin (EPB72)-like 1 | 1.048275011 | 5.95E-25 |
| MAPT | microtubule-associated protein tau | 1.587327586 | 9.23E-25 |
| NPM3 | nucleophosmin/nucleoplasmin 3 | 1.00058027 | 9.89E-25 |
| ADCY4 | adenylate cyclase 4 | 1.523310731 | 1.64E-24 |
| GDF15 | growth differentiation factor 15 | 1.01036553 | 1.77E-24 |
| CARD6 | caspase recruitment domain family, member 6 | 1.03159576 | 2.12E-24 |
| HLA-DPA1 | major histocompatibility complex, class II, DP alpha 1 | 1.422035371 | 3.24E-24 |
| IFIT2 | interferon-induced protein with tetratricopeptide repeats 2 | 1.042539529 | 4.84E-24 |
| GRIP2 | glutamate receptor interacting protein 2 | 5.304692433 | 5.53E-24 |
| MOCOS | molybdenum cofactor sulfurase | 1.160396069 | 6.13E-24 |
| FAM5B | family with sequence similarity 5, member B | 3.339430347 | 8.09E-24 |
| LYPD3 | LY6/PLAUR domain containing 3 | 2.737916138 | 1.11E-23 |
| LOC100499467 | uncharacterized LOC100499467 | 2.136949465 | 1.25E-23 |
| COL4A3 | collagen, type IV, alpha 3 (Goodpasture antigen) | 5.199849815 | 2.68E-23 |
| FAM13C | family with sequence similarity 13, member C | 2.200213847 | 3.22E-23 |
| RENBP | renin binding protein | 1.433200214 | 3.57E-23 |
| SFXN5 | sideroflexin 5 | 1.065999757 | 3.77E-23 |
| STARD10 | StAR-related lipid transfer (START) domain containing 10 | 1.08269219 | 3.80E-23 |
| INMT | indolethylamine N-methyltransferase | 1.948311808 | 4.91E-23 |
| PRADC1 | protease-associated domain containing 1 | 1.199742334 | 5.35E-23 |
| MCTP1 | multiple C2 domains, transmembrane 1 | 2.088863236 | 7.42E-23 |
| HOXA11 | homeobox A11 | 1.016549682 | 8.22E-23 |
| SHC2 | SHC (Src homology 2 domain containing) transforming protein 2 | 1.970160372 | 9.27E-23 |
| FGF11 | fibroblast growth factor 11 | 3.482140123 | 1.04E-22 |
| UBXN2B | UBX domain protein 2B | 1.0465114 | 2.75E-22 |
| ENTPD7 | ectonucleoside triphosphate diphosphohydrolase 7 | 1.144442423 | 2.81E-22 |
| AFF2 | AF4/FMR2 family, member 2 | 1.835401884 | 3.30E-22 |
| HECW2 | HECT, C2 and WW domain containing E3 ubiquitin protein ligase 2 | 1.209919971 | 3.44E-22 |
| KCNG1 | potassium voltage-gated channel, subfamily G, member 1 | 2.017212418 | 4.90E-22 |
| C2orf88 | chromosome 2 open reading frame 88 | 1.308039413 | 7.38E-22 |
| KIF20A | kinesin family member 20A | 2.84957728 | 7.51E-22 |
| KIAA1211 | KIAA1211 | 2.290635104 | 1.07E-21 |
| PLCH1 | phospholipase C, eta 1 | 4.015849465 | 1.18E-21 |
| LOC100128252 | uncharacterized LOC100128252 | 1.172142661 | 1.26E-21 |
| C11orf96 | chromosome 11 open reading frame 96 | 2.539625069 | 1.60E-21 |
| DLX3 | distal-less homeobox 3 | 4.371010537 | 1.90E-21 |
| IL1RL1 | interleukin 1 receptor-like 1 | 2.649603833 | 2.83E-21 |
| ZNF385D | zinc finger protein 385D | 1.581580576 | 5.02E-21 |
| RAB33A | RAB33A, member RAS oncogene family | 2.206718324 | 5.32E-21 |
| DUOX1 | dual oxidase 1 | 2.852910868 | 5.70E-21 |
| TMEM30B | transmembrane protein 30B | 2.140176596 | 8.42E-21 |
| P2RY1 | purinergic receptor P2Y, G-protein coupled, 1 | 2.360047368 | 1.02E-20 |
| MFSD3 | major facilitator superfamily domain containing 3 | 1.086252381 | 5.13E-20 |
| ESR1 | estrogen receptor 1 | 1.220798535 | 6.99E-20 |
| RORB | RAR-related orphan receptor B | 2.813402974 | 6.99E-20 |
| PPIL1 | peptidylprolyl isomerase (cyclophilin)-like 1 | 1.061068046 | 7.36E-20 |
| RHBDF2 | rhomboid 5 homolog 2 (Drosophila) | 1.198139809 | 8.58E-20 |
| BMP6 | bone morphogenetic protein 6 | 1.947045437 | 9.58E-20 |
| TTTY15 | testis-specific transcript, Y-linked 15 (non-protein coding) | 4.012997646 | 1.26E-19 |
| GPM6B | glycoprotein M6B | 1.420022214 | 2.12E-19 |
| MKI67 | antigen identified by monoclonal antibody Ki-67 | 1.331732517 | 2.92E-19 |
| TSPAN13 | tetraspanin 13 | 1.964735339 | 3.42E-19 |
| RIPK3 | receptor-interacting serine-threonine kinase 3 | 1.04480636 | 3.69E-19 |
| LPAR4 | lysophosphatidic acid receptor 4 | 3.07936724 | 4.83E-19 |
| TK1 | thymidine kinase 1, soluble | 1.776862413 | 5.00E-19 |
| C8orf34 | chromosome 8 open reading frame 34 | 2.059504879 | 5.33E-19 |
| ABCB4 | ATP-binding cassette, sub-family B (MDR/TAP), member 4 | 1.204082442 | 9.91E-19 |
| EIF1AY | eukaryotic translation initiation factor 1A, Y-linked | 4.100740452 | 1.34E-18 |
| NXPH4 | neurexophilin 4 | 1.450593682 | 1.97E-18 |
| BCL11B | B-cell CLL/lymphoma 11B (zinc finger protein) | 3.094422944 | 2.68E-18 |
| RNF166 | ring finger protein 166 | 1.063230428 | 2.96E-18 |
| NMB | neuromedin B | 1.152445824 | 4.13E-18 |
| CLGN | calmegin | 1.027129086 | 4.25E-18 |
| CHDH | choline dehydrogenase | 1.003373032 | 4.58E-18 |
| ALS2CL | ALS2 C-terminal like | 1.44975915 | 4.92E-18 |
| MCHR1 | melanin-concentrating hormone receptor 1 | 4.113475422 | 8.13E-18 |
| CA11 | carbonic anhydrase XI | 1.863147469 | 1.15E-17 |
| GCH1 | GTP cyclohydrolase 1 | 1.494624149 | 1.20E-17 |
| KALRN | kalirin, RhoGEF kinase | 1.854387659 | 1.30E-17 |
| PDXP | pyridoxal (pyridoxine, vitamin B6) phosphatase | 1.140213726 | 1.37E-17 |
| BATF2 | basic leucine zipper transcription factor, ATF-like 2 | 1.928686174 | 1.40E-17 |
| LRRN3 | leucine rich repeat neuronal 3 | 2.438643851 | 2.73E-17 |
| BEND5 | BEN domain containing 5 | 3.648748023 | 3.85E-17 |
| TFEB | transcription factor EB | 1.229491114 | 3.87E-17 |
| ANLN | anillin, actin binding protein | 1.433449203 | 4.94E-17 |
| PDCD1LG2 | programmed cell death 1 ligand 2 | 1.195755405 | 6.95E-17 |
| TRIM9 | tripartite motif containing 9 | 2.115038085 | 7.21E-17 |
| GRIA4 | glutamate receptor, ionotrophic, AMPA 4 | 2.786153232 | 8.36E-17 |
| MAP7D2 | MAP7 domain containing 2 | 2.939118316 | 8.86E-17 |
| TNK1 | tyrosine kinase, non-receptor, 1 | 2.817498766 | 1.16E-16 |
| CEBPA | CCAAT/enhancer binding protein (C/EBP), alpha | 2.044499054 | 1.54E-16 |
| TYW1B | tRNA-yW synthesizing protein 1 homolog B (S. cerevisiae) | 2.641384112 | 2.38E-16 |
| NRXN2 | neurexin 2 | 1.671364124 | 2.70E-16 |
| LOC147727 | uncharacterized LOC147727 | 1.083437609 | 3.82E-16 |
| FRRS1 | ferric-chelate reductase 1 | 1.204113905 | 4.59E-16 |
| CCL5 | chemokine (C-C motif) ligand 5 | 2.434356626 | 4.63E-16 |
| NCAM2 | neural cell adhesion molecule 2 | 1.31944092 | 6.51E-16 |
| NAALAD2 | N-acetylated alpha-linked acidic dipeptidase 2 | 1.738812141 | 8.01E-16 |
| FUCA1 | fucosidase, alpha-L- 1, tissue | 1.398696848 | 8.14E-16 |
| C1QTNF4 | C1q and tumor necrosis factor related protein 4 | 3.049142398 | 8.28E-16 |
| LRRC16A | leucine rich repeat containing 16A | 1.109842892 | 8.29E-16 |
| GIMAP2 | GTPase, IMAP family member 2 | 2.102489653 | 9.77E-16 |
| PENK | proenkephalin | 2.170546359 | 1.08E-15 |
| MRAP2 | melanocortin 2 receptor accessory protein 2 | 2.682952331 | 1.24E-15 |
| H2AFY2 | H2A histone family, member Y2 | 1.232697879 | 1.44E-15 |
| CSF3 | colony stimulating factor 3 (granulocyte) | 3.071909583 | 1.56E-15 |
| ACN9 | ACN9 homolog (S. cerevisiae) | 1.177305018 | 1.67E-15 |
| LOC344887 | NmrA-like family domain containing 1 pseudogene | 2.463634314 | 1.79E-15 |
| INA | internexin neuronal intermediate filament protein, alpha | 1.598570571 | 1.86E-15 |
| TSPAN18 | tetraspanin 18 | 3.077145812 | 2.06E-15 |
| PALM2 | paralemmin 2 | 1.746006955 | 2.15E-15 |
| LINC00478 | long intergenic non-protein coding RNA 478 | 1.221959042 | 2.26E-15 |
| CAMK1D | calcium/calmodulin-dependent protein kinase ID | 2.0774817 | 2.37E-15 |
| SAMD12 | sterile alpha motif domain containing 12 | 2.590974533 | 2.54E-15 |
| PNMA2 | paraneoplastic antigen MA2 | 2.128654413 | 2.81E-15 |
| SLC7A2 | solute carrier family 7 (cationic amino acid transporter, y+ system), member 2 | 1.072723198 | 3.94E-15 |
| MLLT11 | myeloid/lymphoid or mixed-lineage leukemia (trithorax homolog, Drosophila); translocated to, 11 | 1.718959598 | 3.96E-15 |
| MIAT | myocardial infarction associated transcript (non-protein coding) | 1.123672745 | 6.77E-15 |
| GPR173 | G protein-coupled receptor 173 | 1.069565242 | 7.15E-15 |
| OASL | 2'-5'-oligoadenylate synthetase-like | 2.957859099 | 8.28E-15 |
| ABLIM1 | actin binding LIM protein 1 | 1.540091488 | 1.02E-14 |
| HLA-DRA | major histocompatibility complex, class II, DR alpha | 2.100323838 | 1.03E-14 |
| ACP5 | acid phosphatase 5, tartrate resistant | 2.947150583 | 1.06E-14 |
| NXT1 | NTF2-like export factor 1 | 1.030172907 | 1.40E-14 |
| BIRC5 | baculoviral IAP repeat containing 5 | 1.921491759 | 1.49E-14 |
| MTUS1 | microtubule associated tumor suppressor 1 | 1.473874852 | 1.73E-14 |
| DUSP23 | dual specificity phosphatase 23 | 1.094857581 | 2.33E-14 |
| C2orf74 | chromosome 2 open reading frame 74 | 1.249836663 | 3.21E-14 |
| CP | ceruloplasmin (ferroxidase) | 2.571644959 | 3.27E-14 |
| HTR2A | 5-hydroxytryptamine (serotonin) receptor 2A | 1.329223867 | 3.53E-14 |
| PLA2G2A | phospholipase A2, group IIA (platelets, synovial fluid) | 3.534186496 | 3.71E-14 |
| EDN1 | endothelin 1 | 2.38741591 | 3.73E-14 |
| CD58 | CD58 molecule | 1.182790611 | 3.74E-14 |
| CXCL3 | chemokine (C-X-C motif) ligand 3 | 2.791605863 | 4.51E-14 |
| SLC22A4 | solute carrier family 22 (organic cation/ergothioneine transporter), member 4 | 1.418982764 | 7.07E-14 |
| NR3C2 | nuclear receptor subfamily 3, group C, member 2 | 2.343931445 | 7.78E-14 |
| HSD11B1 | hydroxysteroid (11-beta) dehydrogenase 1 | 1.414841268 | 8.32E-14 |
| ARG2 | arginase, type II | 1.273871105 | 1.00E-13 |
| GEMIN2 | gem (nuclear organelle) associated protein 2 | 1.332886973 | 1.55E-13 |
| ZNF184 | zinc finger protein 184 | 1.046187651 | 1.72E-13 |
| ANKRD37 | ankyrin repeat domain 37 | 2.672756125 | 1.99E-13 |
| RAI2 | retinoic acid induced 2 | 2.335922862 | 2.09E-13 |
| MFSD2A | major facilitator superfamily domain containing 2A | 2.309627907 | 2.64E-13 |
| GRIK2 | glutamate receptor, ionotropic, kainate 2 | 1.046295747 | 2.64E-13 |
| MACROD2 | MACRO domain containing 2 | 1.505034816 | 2.97E-13 |
| IQGAP3 | IQ motif containing GTPase activating protein 3 | 1.985283045 | 3.17E-13 |
| PTGER3 | prostaglandin E receptor 3 (subtype EP3) | 2.46948665 | 3.41E-13 |
| LIMS2 | LIM and senescent cell antigen-like domains 2 | 1.334227691 | 4.23E-13 |
| RRAD | Ras-related associated with diabetes | 1.479082783 | 4.52E-13 |
| ASF1B | ASF1 anti-silencing function 1 homolog B (S. cerevisiae) | 2.432677852 | 5.29E-13 |
| PAPPA2 | pappalysin 2 | 2.00356939 | 6.83E-13 |
| ETV7 | ets variant 7 | 1.978831368 | 9.05E-13 |
| LOC550112 | uncharacterized LOC550112 | 1.252588489 | 1.37E-12 |
| CILP2 | cartilage intermediate layer protein 2 | 1.381836683 | 1.46E-12 |
| PTGS2 | prostaglandin-endoperoxide synthase 2 (prostaglandin G/H synthase and cyclooxygenase) | 1.088968844 | 1.46E-12 |
| FAM174B | family with sequence similarity 174, member B | 1.534593662 | 1.63E-12 |
| ZNF662 | zinc finger protein 662 | 1.428145268 | 1.71E-12 |
| SH3RF2 | SH3 domain containing ring finger 2 | 2.679836593 | 1.95E-12 |
| SPTLC3 | serine palmitoyltransferase, long chain base subunit 3 | 1.161478886 | 2.48E-12 |
| NTRK2 | neurotrophic tyrosine kinase, receptor, type 2 | 2.238104538 | 2.91E-12 |
| C15orf23 | chromosome 15 open reading frame 23 | 1.093575858 | 4.29E-12 |
| FRG1B | FSHD region gene 1 family, member B | 1.25461786 | 5.14E-12 |
| C20orf24 | chromosome 20 open reading frame 24 | 1.170648905 | 6.96E-12 |
| FGD3 | FYVE, RhoGEF and PH domain containing 3 | 2.609015835 | 7.39E-12 |
| LOC100128822 | uncharacterized LOC100128822 | 1.400816549 | 7.74E-12 |
| UCP2 | uncoupling protein 2 (mitochondrial, proton carrier) | 2.514364151 | 9.80E-12 |
| SLC16A4 | solute carrier family 16, member 4 (monocarboxylic acid transporter 5) | 1.411294051 | 9.81E-12 |
| CADM1 | cell adhesion molecule 1 | 3.168442973 | 1.28E-11 |
| TAS1R3 | taste receptor, type 1, member 3 | 2.089697465 | 1.67E-11 |
| RXFP1 | relaxin/insulin-like family peptide receptor 1 | 1.509682909 | 1.81E-11 |
| C8orf4 | chromosome 8 open reading frame 4 | 1.681797448 | 2.05E-11 |
| MIF4GD | MIF4G domain containing | 1.485376695 | 2.48E-11 |
| PODXL2 | podocalyxin-like 2 | 1.740656825 | 2.51E-11 |
| RRM2 | ribonucleotide reductase M2 | 1.350958052 | 2.88E-11 |
| KYNU | kynureninase | 1.584473961 | 3.78E-11 |
| CRYM | crystallin, mu | 2.007970902 | 3.81E-11 |
| DISP2 | dispatched homolog 2 (Drosophila) | 1.226522139 | 3.95E-11 |
| PIWIL2 | piwi-like 2 (Drosophila) | 2.389661527 | 4.17E-11 |
| CSTA | cystatin A (stefin A) | 1.764533315 | 5.16E-11 |
| GTSE1 | G-2 and S-phase expressed 1 | 1.797899986 | 9.33E-11 |
| NEURL1B | neuralized homolog 1B (Drosophila) | 1.926399944 | 1.38E-10 |
| PCSK1N | proprotein convertase subtilisin/kexin type 1 inhibitor | 1.823237404 | 1.73E-10 |
| CDC7 | cell division cycle 7 homolog (S. cerevisiae) | 1.081608808 | 1.75E-10 |
| ECE2 | endothelin converting enzyme 2 | 1.597782427 | 2.12E-10 |
| TMOD1 | tropomodulin 1 | 2.630748048 | 2.71E-10 |
| RGS7 | regulator of G-protein signaling 7 | 1.471778558 | 3.83E-10 |
| LOC100506930 | uncharacterized LOC100506930 | 1.769866601 | 3.92E-10 |
| THBS4 | thrombospondin 4 | 1.066745478 | 4.40E-10 |
| LINC00472 | long intergenic non-protein coding RNA 472 | 1.54440466 | 4.85E-10 |
| SLC2A5 | solute carrier family 2 (facilitated glucose/fructose transporter), member 5 | 1.466144926 | 5.02E-10 |
| ALX4 | ALX homeobox 4 | 2.076128101 | 5.06E-10 |
| NIM1 | serine/threonine-protein kinase NIM1 | 1.679326029 | 5.17E-10 |
| EXPH5 | exophilin 5 | 1.481765879 | 6.16E-10 |
| MCOLN3 | mucolipin 3 | 1.31546744 | 6.36E-10 |
| LOC154761 | uncharacterized LOC154761 | 1.053393462 | 6.45E-10 |
| EDIL3 | EGF-like repeats and discoidin I-like domains 3 | 1.295599256 | 6.53E-10 |
| KCNT2 | potassium channel, subfamily T, member 2 | 1.886974556 | 7.10E-10 |
| LOC100128361 | uncharacterized LOC100128361 | 2.034314835 | 8.91E-10 |
| ENPP5 | ectonucleotide pyrophosphatase/phosphodiesterase 5 (putative) | 1.145981548 | 1.52E-09 |
| RHBDL2 | rhomboid, veinlet-like 2 (Drosophila) | 2.208795958 | 2.24E-09 |
| IL1RN | interleukin 1 receptor antagonist | 1.909779303 | 2.29E-09 |
| CCDC152 | coiled-coil domain containing 152 | 1.825486774 | 2.47E-09 |
| KCNMB4 | potassium large conductance calcium-activated channel, subfamily M, beta member 4 | 2.090725743 | 2.62E-09 |
| CXCL2 | chemokine (C-X-C motif) ligand 2 | 1.973090988 | 2.97E-09 |
| KLF8 | Kruppel-like factor 8 | 1.374784675 | 3.62E-09 |
| RNF144B | ring finger protein 144B | 1.279513159 | 3.72E-09 |
| MYO3B | myosin IIIB | 1.728822389 | 5.33E-09 |
| CDC20 | cell division cycle 20 homolog (S. cerevisiae) | 1.447268651 | 5.52E-09 |
| BZRAP1 | benzodiazapine receptor (peripheral) associated protein 1 | 1.898675614 | 6.54E-09 |
| EGR3 | early growth response 3 | 1.014140389 | 7.44E-09 |
| NPAS3 | neuronal PAS domain protein 3 | 1.255337483 | 7.51E-09 |
| SEMA3G | sema domain, immunoglobulin domain (Ig), short basic domain, secreted, (semaphorin) 3G | 1.731093658 | 8.36E-09 |
| EREG | epiregulin | 1.534963593 | 8.59E-09 |
| STARD8 | StAR-related lipid transfer (START) domain containing 8 | 1.678749693 | 1.04E-08 |
| C11orf87 | chromosome 11 open reading frame 87 | 1.424490677 | 1.10E-08 |
| GRHL1 | grainyhead-like 1 (Drosophila) | 1.789620916 | 1.33E-08 |
| ZBED3 | zinc finger, BED-type containing 3 | 1.097620306 | 1.65E-08 |
| FREM1 | FRAS1 related extracellular matrix 1 | 1.485852012 | 2.36E-08 |
| LCP1 | lymphocyte cytosolic protein 1 (L-plastin) | 2.291536428 | 2.47E-08 |
| MAMDC4 | MAM domain containing 4 | 1.022276838 | 2.58E-08 |
| NTSR1 | neurotensin receptor 1 (high affinity) | 1.553971508 | 2.93E-08 |
| LOC390940 | uncharacterized protein ENSP00000244321 | 1.594968455 | 3.51E-08 |
| ID4 | inhibitor of DNA binding 4, dominant negative helix-loop-helix protein | 1.261594581 | 3.65E-08 |
| FAM158A | family with sequence similarity 158, member A | 1.309211276 | 3.83E-08 |
| ZNF439 | zinc finger protein 439 | 1.595540177 | 4.91E-08 |
| PTPRC | protein tyrosine phosphatase, receptor type, C | 8.196174581 | 5.48E-08 |
| IGSF3 | immunoglobulin superfamily, member 3 | 1.48303439 | 6.29E-08 |
| PSD4 | pleckstrin and Sec7 domain containing 4 | 1.320974974 | 7.61E-08 |
| CXCR4 | chemokine (C-X-C motif) receptor 4 | 6.663306009 | 8.88E-08 |
| SAMD5 | sterile alpha motif domain containing 5 | 1.11399762 | 1.04E-07 |
| PCDH1 | protocadherin 1 | 1.628192278 | 1.08E-07 |
| HOXD3 | homeobox D3 | 1.080838516 | 1.18E-07 |
| ASPN | asporin | 1.008468032 | 1.38E-07 |
| HOXA13 | homeobox A13 | 1.66135095 | 1.53E-07 |
| DHRS13 | dehydrogenase/reductase (SDR family) member 13 | 1.036644947 | 2.02E-07 |
| CDK1 | cyclin-dependent kinase 1 | 1.394978391 | 2.22E-07 |
| KCNA3 | potassium voltage-gated channel, shaker-related subfamily, member 3 | 1.947596735 | 2.32E-07 |
| TMEM102 | transmembrane protein 102 | 1.660759187 | 2.67E-07 |
| CLSPN | claspin | 1.414154529 | 2.79E-07 |
| KIFC1 | kinesin family member C1 | 1.496307015 | 3.01E-07 |
| DTWD2 | DTW domain containing 2 | 1.002333255 | 3.09E-07 |
| ZNF853 | zinc finger protein 853 | 1.650469804 | 3.35E-07 |
| CACNB4 | calcium channel, voltage-dependent, beta 4 subunit | 1.223491293 | 3.44E-07 |
| FAM78A | family with sequence similarity 78, member A | 1.117550362 | 3.73E-07 |
| ZNF467 | zinc finger protein 467 | 1.957989868 | 4.22E-07 |
| RAB3IP | RAB3A interacting protein (rabin3) | 1.227335867 | 4.73E-07 |
| TFAP2A | transcription factor AP-2 alpha (activating enhancer binding protein 2 alpha) | 1.324227876 | 6.08E-07 |
| KCNJ15 | potassium inwardly-rectifying channel, subfamily J, member 15 | 1.18590295 | 7.15E-07 |
| SLC17A7 | solute carrier family 17 (sodium-dependent inorganic phosphate cotransporter), member 7 | 1.028876161 | 9.38E-07 |
| SPAG1 | sperm associated antigen 1 | 1.125808185 | 9.66E-07 |
| KIF24 | kinesin family member 24 | 1.655866622 | 1.20E-06 |
| C1orf51 | chromosome 1 open reading frame 51 | 1.481225159 | 1.22E-06 |
| DBNDD1 | dysbindin (dystrobrevin binding protein 1) domain containing 1 | 1.651541022 | 1.37E-06 |
| TPK1 | thiamin pyrophosphokinase 1 | 1.496683932 | 1.66E-06 |
| ARHGEF37 | Rho guanine nucleotide exchange factor (GEF) 37 | 1.399321196 | 1.88E-06 |
| PNMAL1 | PNMA-like 1 | 1.278760578 | 2.12E-06 |
| ANKFN1 | ankyrin-repeat and fibronectin type III domain containing 1 | 1.415106907 | 2.60E-06 |
| RELT | RELT tumor necrosis factor receptor | 1.015018145 | 3.77E-06 |
| ADSSL1 | adenylosuccinate synthase like 1 | 1.077788312 | 3.89E-06 |
| MARK1 | MAP/microtubule affinity-regulating kinase 1 | 1.153950983 | 4.28E-06 |
| CEP55 | centrosomal protein 55kDa | 1.335756877 | 4.44E-06 |
| FIBCD1 | fibrinogen C domain containing 1 | 1.299320868 | 4.76E-06 |
| TMEM182 | transmembrane protein 182 | 1.432965522 | 5.55E-06 |
| TNFRSF10C | tumor necrosis factor receptor superfamily, member 10c, decoy without an intracellular domain | 1.286759224 | 6.38E-06 |
| VASH2 | vasohibin 2 | 1.158874409 | 8.01E-06 |
| LGALSL | lectin, galactoside-binding-like | 1.030267096 | 1.50E-05 |
| PLK1 | polo-like kinase 1 | 1.152126064 | 1.81E-05 |
| PBK | PDZ binding kinase | 1.475224636 | 1.98E-05 |
| C14orf80 | chromosome 14 open reading frame 80 | 1.185238789 | 1.98E-05 |
| AFF3 | AF4/FMR2 family, member 3 | 1.267512921 | 2.08E-05 |
| SLITRK4 | SLIT and NTRK-like family, member 4 | 1.706802688 | 2.18E-05 |
| ARHGDIB | Rho GDP dissociation inhibitor (GDI) beta | 2.25365757 | 2.29E-05 |
| PAQR4 | progestin and adipoQ receptor family member IV | 1.047797859 | 2.64E-05 |
| KIAA0664L3 | KIAA0664-like 3 | 1.295164551 | 2.68E-05 |
| CENPP | centromere protein P | 1.341524202 | 3.74E-05 |
| ZNF704 | zinc finger protein 704 | 1.071586755 | 3.90E-05 |
| ICOSLG | inducible T-cell co-stimulator ligand | 1.425744828 | 4.39E-05 |
| SERTAD4 | SERTA domain containing 4 | 1.234887796 | 4.84E-05 |
| RTP4 | receptor (chemosensory) transporter protein 4 | 1.245442252 | 5.59E-05 |
| PCDHB14 | protocadherin beta 14 | 1.232595925 | 7.09E-05 |
| SPOCK2 | sparc/osteonectin, cwcv and kazal-like domains proteoglycan (testican) 2 | 3.058609542 | 7.28E-05 |
| UBE2T | ubiquitin-conjugating enzyme E2T (putative) | 1.161361404 | 8.10E-05 |
| C21orf67 | chromosome 21 open reading frame 67 | 1.104818712 | 8.13E-05 |
| CECR1 | cat eye syndrome chromosome region, candidate 1 | 1.11898648 | 8.34E-05 |
| MTFP1 | mitochondrial fission process 1 | 1.151279855 | 8.96E-05 |
| TTC3P1 | tetratricopeptide repeat domain 3 pseudogene 1 | 1.216010455 | 8.96E-05 |
| PCDHB5 | protocadherin beta 5 | 1.195644497 | 9.41E-05 |
| DYSF | dysferlin, limb girdle muscular dystrophy 2B (autosomal recessive) | 1.2838316 | 0.000111954 |
| CAPS2 | calcyphosine 2 | 1.15605363 | 0.000178543 |
| SHCBP1 | SHC SH2-domain binding protein 1 | 1.114977135 | 0.000219729 |
| RAD54B | RAD54 homolog B (S. cerevisiae) | 1.162342374 | 0.000221309 |
| YY2 | YY2 transcription factor | 1.018793383 | 0.000249309 |
| SHROOM4 | shroom family member 4 | 1.141569216 | 0.000268873 |
| CACNB2 | calcium channel, voltage-dependent, beta 2 subunit | 1.022531982 | 0.00029667 |
| LOC388152 | uncharacterized LOC388152 | 1.125621051 | 0.000325258 |
| NR4A2 | nuclear receptor subfamily 4, group A, member 2 | 1.059723104 | 0.000338 |
| MIR600HG | MIR600 host gene (non-protein coding) | 1.023251619 | 0.000372448 |
| NDC80 | NDC80 homolog, kinetochore complex component (S. cerevisiae) | 1.112469426 | 0.000445168 |
| SIK1 | salt-inducible kinase 1 | 1.162950203 | 0.00045171 |
| TMSB15B | thymosin beta 15B | 1.003845004 | 0.00045596 |
| KIF2C | kinesin family member 2C | 1.140919248 | 0.00080047 |
| LOC646214 | p21 protein (Cdc42/Rac)-activated kinase 2 pseudogene | 1.019362523 | 0.001523309 |
| BUB1 | budding uninhibited by benzimidazoles 1 homolog (yeast) | 1.013935984 | 0.001816369 |
| ZSCAN16 | zinc finger and SCAN domain containing 16 | 1.126611631 | 0.001840966 |
| FOS | FBJ murine osteosarcoma viral oncogene homolog | 1.091194901 | 0.002695552 |
| FOSB | FBJ murine osteosarcoma viral oncogene homolog B | 2.041386811 | 0.004402728 |

Gene down

| GeneSymbol | description | log2FC | FDR |
| --- | --- | --- | --- |
| FBN2 | fibrillin 2 | -4.924705486 | 0 |
| GALNTL2 | UDP-N-acetyl-alpha-D-galactosamine:polypeptide N-acetylgalactosaminyltransferase-like 2 | -4.803000165 | 0 |
| HSPB7 | heat shock 27kDa protein family, member 7 (cardiovascular) | -4.194722935 | 0 |
| SLC7A14 | solute carrier family 7 (orphan transporter), member 14 | -3.463792514 | 0 |
| SCD | stearoyl-CoA desaturase (delta-9-desaturase) | -3.087685708 | 0 |
| SFRP1 | secreted frizzled-related protein 1 | -2.904106215 | 0 |
| LDLR | low density lipoprotein receptor | -2.674569685 | 0 |
| FASN | fatty acid synthase | -2.313621095 | 0 |
| HSPB6 | heat shock protein, alpha-crystallin-related, B6 | -2.283340363 | 0 |
| FADS1 | fatty acid desaturase 1 | -2.241638984 | 0 |
| ADAMTS1 | ADAM metallopeptidase with thrombospondin type 1 motif, 1 | -2.174280401 | 0 |
| IGFBP6 | insulin-like growth factor binding protein 6 | -2.118138562 | 0 |
| THBS1 | thrombospondin 1 | -2.114781794 | 0 |
| COL12A1 | collagen, type XII, alpha 1 | -2.000376295 | 0 |
| FADS2 | fatty acid desaturase 2 | -1.916019193 | 0 |
| TGFBI | transforming growth factor, beta-induced, 68kDa | -1.907715865 | 0 |
| VIM | vimentin | -1.901696046 | 9.42183186619257e-321 |
| CPNE7 | copine VII | -5.319175776 | 1.25E-303 |
| DHCR24 | 24-dehydrocholesterol reductase | -1.897838452 | 1.10E-299 |
| PCSK9 | proprotein convertase subtilisin/kexin type 9 | -4.241785349 | 1.25E-295 |
| VAT1L | vesicle amine transport protein 1 homolog (T. californica)-like | -5.875730241 | 3.73E-289 |
| ATP2B4 | ATPase, Ca++ transporting, plasma membrane 4 | -1.747940722 | 6.70E-286 |
| NOTCH3 | notch 3 | -2.606983524 | 1.71E-278 |
| AKAP12 | A kinase (PRKA) anchor protein 12 | -1.93152034 | 1.24E-275 |
| THSD4 | thrombospondin, type I, domain containing 4 | -3.757839438 | 2.56E-265 |
| LOX | lysyl oxidase | -1.896852033 | 4.83E-265 |
| CDH13 | cadherin 13, H-cadherin (heart) | -2.691177868 | 2.22E-264 |
| FABP3 | fatty acid binding protein 3, muscle and heart (mammary-derived growth inhibitor) | -2.872124375 | 2.14E-258 |
| WNT5A | wingless-type MMTV integration site family, member 5A | -2.172311698 | 4.01E-245 |
| S1PR3 | sphingosine-1-phosphate receptor 3 | -2.156666339 | 3.15E-242 |
| SBSN | suprabasin | -2.480314067 | 5.02E-239 |
| LPIN1 | lipin 1 | -1.9075842 | 2.79E-233 |
| DHCR7 | 7-dehydrocholesterol reductase | -2.347657078 | 3.74E-231 |
| HOXC8 | homeobox C8 | -2.309885319 | 1.26E-229 |
| PRSS12 | protease, serine, 12 (neurotrypsin, motopsin) | -2.095590896 | 9.46E-222 |
| NPTX1 | neuronal pentraxin I | -3.068832207 | 2.81E-218 |
| MALAT1 | metastasis associated lung adenocarcinoma transcript 1 (non-protein coding) | -2.170543079 | 2.23E-215 |
| FLNB | filamin B, beta | -1.678756427 | 8.86E-215 |
| MFI2 | antigen p97 (melanoma associated) identified by monoclonal antibodies 133.2 and 96.5 | -3.164313753 | 7.97E-210 |
| HMGCS1 | 3-hydroxy-3-methylglutaryl-CoA synthase 1 (soluble) | -2.189065709 | 2.46E-204 |
| PAPPA | pregnancy-associated plasma protein A, pappalysin 1 | -1.556308495 | 8.16E-204 |
| SHC3 | SHC (Src homology 2 domain containing) transforming protein 3 | -2.326739865 | 4.05E-203 |
| FZD6 | frizzled family receptor 6 | -2.517069005 | 6.21E-202 |
| MSMO1 | methylsterol monooxygenase 1 | -2.272910065 | 1.25E-199 |
| CLDN11 | claudin 11 | -1.384209456 | 1.57E-199 |
| MYOF | myoferlin | -1.413181737 | 3.76E-198 |
| TRHDE | thyrotropin-releasing hormone degrading enzyme | -3.987765719 | 6.56E-197 |
| INSIG1 | insulin induced gene 1 | -1.649373621 | 1.95E-191 |
| SYTL2 | synaptotagmin-like 2 | -2.475231675 | 3.88E-190 |
| DENND4C | DENN/MADD domain containing 4C | -1.790254204 | 2.89E-185 |
| ACE | angiotensin I converting enzyme (peptidyl-dipeptidase A) 1 | -2.22026589 | 1.39E-184 |
| SQLE | squalene epoxidase | -1.937816609 | 3.90E-184 |
| CYP51A1 | cytochrome P450, family 51, subfamily A, polypeptide 1 | -1.786991999 | 1.43E-183 |
| HEG1 | HEG homolog 1 (zebrafish) | -1.475048923 | 1.81E-181 |
| PCYOX1 | prenylcysteine oxidase 1 | -1.400354964 | 1.93E-179 |
| FLNC | filamin C, gamma | -1.485051978 | 3.12E-178 |
| RSPO4 | R-spondin 4 | -3.295420691 | 7.46E-177 |
| CD46 | CD46 molecule, complement regulatory protein | -1.501966452 | 9.10E-177 |
| IGFBP4 | insulin-like growth factor binding protein 4 | -1.380101311 | 3.56E-176 |
| STS | steroid sulfatase (microsomal), isozyme S | -1.826608408 | 1.19E-175 |
| EPHB6 | EPH receptor B6 | -2.400022686 | 4.44E-175 |
| FDPS | farnesyl diphosphate synthase | -1.704704952 | 2.31E-174 |
| MDK | midkine (neurite growth-promoting factor 2) | -1.87745192 | 1.18E-173 |
| LOC389831 | uncharacterized LOC389831 | -2.546764249 | 4.59E-172 |
| DMKN | dermokine | -1.916646424 | 7.92E-169 |
| CRIM1 | cysteine rich transmembrane BMP regulator 1 (chordin-like) | -1.555910014 | 1.81E-167 |
| SYNPO2 | synaptopodin 2 | -1.829267 | 1.78E-166 |
| PLIN4 | perilipin 4 | -2.868821277 | 2.12E-164 |
| ALPK2 | alpha-kinase 2 | -2.21976218 | 4.29E-164 |
| COLEC12 | collectin sub-family member 12 | -1.3332332 | 5.61E-163 |
| HMGCR | 3-hydroxy-3-methylglutaryl-CoA reductase | -1.77125077 | 2.74E-160 |
| F10 | coagulation factor X | -1.889118476 | 1.03E-159 |
| ADAMTSL1 | ADAMTS-like 1 | -1.467354194 | 1.53E-159 |
| ERAP2 | endoplasmic reticulum aminopeptidase 2 | -1.891801912 | 1.12E-157 |
| GPX3 | glutathione peroxidase 3 (plasma) | -3.080372484 | 1.15E-155 |
| STC2 | stanniocalcin 2 | -1.434819215 | 3.98E-155 |
| CFL2 | cofilin 2 (muscle) | -1.642045934 | 6.50E-153 |
| PEG10 | paternally expressed 10 | -2.508674719 | 1.86E-151 |
| BAALC | brain and acute leukemia, cytoplasmic | -1.723196931 | 7.87E-150 |
| ABCA8 | ATP-binding cassette, sub-family A (ABC1), member 8 | -2.60057917 | 4.72E-147 |
| ADAM12 | ADAM metallopeptidase domain 12 | -1.631402917 | 1.22E-144 |
| FDFT1 | farnesyl-diphosphate farnesyltransferase 1 | -1.668174442 | 6.86E-143 |
| CD109 | CD109 molecule | -1.529772987 | 7.72E-143 |
| LAMC1 | laminin, gamma 1 (formerly LAMB2) | -1.128400588 | 1.31E-142 |
| MEST | mesoderm specific transcript homolog (mouse) | -2.062682462 | 1.99E-135 |
| CLEC3B | C-type lectin domain family 3, member B | -1.827174792 | 7.12E-134 |
| UTRN | utrophin | -1.463965675 | 2.03E-132 |
| HSPA2 | heat shock 70kDa protein 2 | -3.20203913 | 1.05E-131 |
| IDI1 | isopentenyl-diphosphate delta isomerase 1 | -1.883157084 | 4.34E-131 |
| CDH2 | cadherin 2, type 1, N-cadherin (neuronal) | -2.849788035 | 4.19E-130 |
| PLEC | plectin | -1.246221946 | 1.20E-129 |
| NEXN | nexilin (F actin binding protein) | -2.57573844 | 1.31E-129 |
| AMOTL2 | angiomotin like 2 | -1.524477867 | 7.03E-129 |
| NRP1 | neuropilin 1 | -1.158396845 | 7.83E-128 |
| PDLIM1 | PDZ and LIM domain 1 | -1.606241418 | 4.66E-127 |
| CAPN2 | calpain 2, (m/II) large subunit | -1.126200411 | 7.61E-127 |
| ITM2C | integral membrane protein 2C | -1.303407696 | 4.13E-126 |
| NPR3 | natriuretic peptide receptor C/guanylate cyclase C (atrionatriuretic peptide receptor C) | -2.779906663 | 1.13E-125 |
| DKK3 | dickkopf 3 homolog (Xenopus laevis) | -1.378687312 | 1.69E-125 |
| TM4SF1 | transmembrane 4 L six family member 1 | -3.92080229 | 1.40E-121 |
| CTHRC1 | collagen triple helix repeat containing 1 | -1.293721477 | 1.42E-119 |
| AHNAK | AHNAK nucleoprotein | -1.092919347 | 6.57E-119 |
| MVD | mevalonate (diphospho) decarboxylase | -1.830942526 | 3.35E-115 |
| ADAM9 | ADAM metallopeptidase domain 9 | -1.077112926 | 4.94E-113 |
| NEAT1 | nuclear paraspeckle assembly transcript 1 (non-protein coding) | -1.021421669 | 2.51E-112 |
| FSTL3 | follistatin-like 3 (secreted glycoprotein) | -1.848539536 | 1.50E-111 |
| PABPC1 | poly(A) binding protein, cytoplasmic 1 | -1.055985965 | 1.72E-111 |
| NRN1 | neuritin 1 | -1.581806595 | 1.19E-109 |
| RHOQ | ras homolog gene family, member Q | -1.21771659 | 9.54E-109 |
| H6PD | hexose-6-phosphate dehydrogenase (glucose 1-dehydrogenase) | -1.147263909 | 9.56E-109 |
| FRAS1 | Fraser syndrome 1 | -1.437535923 | 1.97E-106 |
| CDC42EP3 | CDC42 effector protein (Rho GTPase binding) 3 | -1.545480519 | 3.05E-106 |
| MAP1A | microtubule-associated protein 1A | -1.089259667 | 5.42E-106 |
| SREBF2 | sterol regulatory element binding transcription factor 2 | -1.154195688 | 2.68E-105 |
| PEAR1 | platelet endothelial aggregation receptor 1 | -4.832601542 | 9.14E-105 |
| ITGA4 | integrin, alpha 4 (antigen CD49D, alpha 4 subunit of VLA-4 receptor) | -1.419440403 | 3.58E-104 |
| SHROOM3 | shroom family member 3 | -1.83564375 | 3.92E-104 |
| AP1S2 | adaptor-related protein complex 1, sigma 2 subunit | -1.561095705 | 1.15E-103 |
| SSC5D | scavenger receptor cysteine rich domain containing (5 domains) | -1.164697357 | 1.22E-102 |
| HIP1 | huntingtin interacting protein 1 | -1.607439032 | 5.36E-102 |
| ACACA | acetyl-CoA carboxylase alpha | -1.259491096 | 7.20E-102 |
| RALGPS2 | Ral GEF with PH domain and SH3 binding motif 2 | -1.896841598 | 1.76E-100 |
| PLCD3 | phospholipase C, delta 3 | -1.219036647 | 2.63E-100 |
| IGF2BP1 | insulin-like growth factor 2 mRNA binding protein 1 | -3.021478714 | 6.54E-100 |
| ADAM19 | ADAM metallopeptidase domain 19 | -2.731335577 | 8.36E-100 |
| ITGA2 | integrin, alpha 2 (CD49B, alpha 2 subunit of VLA-2 receptor) | -2.242917859 | 3.93E-98 |
| SERPINE1 | serpin peptidase inhibitor, clade E (nexin, plasminogen activator inhibitor type 1), member 1 | -1.069258585 | 1.23E-97 |
| SCN8A | sodium channel, voltage gated, type VIII, alpha subunit | -1.521702278 | 4.15E-97 |
| POLR2A | polymerase (RNA) II (DNA directed) polypeptide A, 220kDa | -1.076868131 | 6.83E-97 |
| NRBP2 | nuclear receptor binding protein 2 | -1.50116096 | 4.44E-96 |
| TRNP1 | TMF1-regulated nuclear protein 1 | -1.697574525 | 2.83E-95 |
| LOC100506548 | uncharacterized LOC100506548 | -1.435851784 | 8.26E-95 |
| QPRT | quinolinate phosphoribosyltransferase | -1.420582132 | 1.00E-94 |
| LSS | lanosterol synthase (2,3-oxidosqualene-lanosterol cyclase) | -1.133148941 | 1.42E-94 |
| TXNRD1 | thioredoxin reductase 1 | -1.06882069 | 1.96E-94 |
| MMP11 | matrix metallopeptidase 11 (stromelysin 3) | -1.552230083 | 2.15E-93 |
| BST1 | bone marrow stromal cell antigen 1 | -2.29707705 | 1.09E-92 |
| TLCD2 | TLC domain containing 2 | -1.620716284 | 2.13E-91 |
| LAYN | layilin | -1.279914109 | 2.56E-91 |
| NIPAL3 | NIPA-like domain containing 3 | -1.383327571 | 1.32E-90 |
| LIN28A | lin-28 homolog A (C. elegans) | -10.98856515 | 1.26E-89 |
| AHSA2 | AHA1, activator of heat shock 90kDa protein ATPase homolog 2 (yeast) | -1.395519404 | 2.00E-89 |
| COL21A1 | collagen, type XXI, alpha 1 | -1.56742427 | 3.69E-89 |
| PHLDB2 | pleckstrin homology-like domain, family B, member 2 | -1.684473788 | 5.18E-88 |
| TOX2 | TOX high mobility group box family member 2 | -3.192275129 | 6.27E-88 |
| ANGPT1 | angiopoietin 1 | -1.610901135 | 1.05E-87 |
| ALDH1A3 | aldehyde dehydrogenase 1 family, member A3 | -1.743595218 | 2.99E-86 |
| SCARB1 | scavenger receptor class B, member 1 | -1.636687218 | 5.58E-86 |
| MMAB | methylmalonic aciduria (cobalamin deficiency) cblB type | -1.793905033 | 7.80E-85 |
| PTPN14 | protein tyrosine phosphatase, non-receptor type 14 | -1.485052542 | 1.21E-84 |
| ADAMTSL4 | ADAMTS-like 4 | -1.269729258 | 1.39E-84 |
| ALCAM | activated leukocyte cell adhesion molecule | -1.400520952 | 5.49E-84 |
| MYL9 | myosin, light chain 9, regulatory | -1.020725484 | 7.03E-84 |
| CRLF1 | cytokine receptor-like factor 1 | -2.508052287 | 1.48E-83 |
| SNTB1 | syntrophin, beta 1 (dystrophin-associated protein A1, 59kDa, basic component 1) | -1.443785692 | 5.56E-83 |
| PTPRF | protein tyrosine phosphatase, receptor type, F | -1.162631449 | 5.07E-81 |
| PDE5A | phosphodiesterase 5A, cGMP-specific | -2.968756419 | 5.12E-81 |
| ATP1B1 | ATPase, Na+/K+ transporting, beta 1 polypeptide | -1.992815987 | 5.95E-81 |
| MYH10 | myosin, heavy chain 10, non-muscle | -1.085850147 | 9.18E-81 |
| GJC1 | gap junction protein, gamma 1, 45kDa | -1.529638445 | 1.33E-78 |
| NES | nestin | -1.384880189 | 3.73E-78 |
| CYTH3 | cytohesin 3 | -1.151163529 | 1.30E-77 |
| SECTM1 | secreted and transmembrane 1 | -1.070878875 | 2.10E-77 |
| RABGAP1 | RAB GTPase activating protein 1 | -1.150188655 | 5.40E-77 |
| DCBLD2 | discoidin, CUB and LCCL domain containing 2 | -1.11384355 | 5.95E-77 |
| LMOD1 | leiomodin 1 (smooth muscle) | -1.991813664 | 1.82E-76 |
| SAV1 | salvador homolog 1 (Drosophila) | -1.213585329 | 1.85E-76 |
| LOC541471 | uncharacterized LOC541471 | -2.032321024 | 3.29E-76 |
| ENPP1 | ectonucleotide pyrophosphatase/phosphodiesterase 1 | -2.11356656 | 1.06E-75 |
| AHRR | aryl-hydrocarbon receptor repressor | -1.122683299 | 2.85E-75 |
| MYADM | myeloid-associated differentiation marker | -1.094112601 | 2.89E-75 |
| NPR1 | natriuretic peptide receptor A/guanylate cyclase A (atrionatriuretic peptide receptor A) | -3.458547243 | 5.32E-74 |
| SC5DL | sterol-C5-desaturase (ERG3 delta-5-desaturase homolog, S. cerevisiae)-like | -1.285688199 | 7.19E-74 |
| SMAD9 | SMAD family member 9 | -2.752936834 | 1.52E-73 |
| LAMA3 | laminin, alpha 3 | -2.249860066 | 3.52E-73 |
| CLIP3 | CAP-GLY domain containing linker protein 3 | -1.039918075 | 7.31E-73 |
| IDH1 | isocitrate dehydrogenase 1 (NADP+), soluble | -1.063111416 | 1.44E-72 |
| ROCK2 | Rho-associated, coiled-coil containing protein kinase 2 | -1.142316304 | 1.51E-72 |
| AMOT | angiomotin | -1.751280435 | 6.37E-71 |
| NTNG1 | netrin G1 | -1.820952991 | 1.55E-70 |
| MASP1 | mannan-binding lectin serine peptidase 1 (C4/C2 activating component of Ra-reactive factor) | -1.066374046 | 2.37E-70 |
| PDCD4 | programmed cell death 4 (neoplastic transformation inhibitor) | -1.145351212 | 9.32E-70 |
| EHD3 | EH-domain containing 3 | -1.094119675 | 3.51E-68 |
| DYNC2H1 | dynein, cytoplasmic 2, heavy chain 1 | -1.109133931 | 1.29E-67 |
| GRK5 | G protein-coupled receptor kinase 5 | -1.548544438 | 1.33E-67 |
| C12orf75 | chromosome 12 open reading frame 75 | -1.049477635 | 5.41E-67 |
| SPOCD1 | SPOC domain containing 1 | -1.869192928 | 7.65E-67 |
| LRRFIP2 | leucine rich repeat (in FLII) interacting protein 2 | -1.097314256 | 8.25E-67 |
| C14orf49 | chromosome 14 open reading frame 49 | -1.460173304 | 2.66E-65 |
| STARD4 | StAR-related lipid transfer (START) domain containing 4 | -1.720949567 | 4.54E-65 |
| GNAI1 | guanine nucleotide binding protein (G protein), alpha inhibiting activity polypeptide 1 | -1.423568232 | 4.94E-65 |
| RAB27B | RAB27B, member RAS oncogene family | -3.509168873 | 6.72E-65 |
| F3 | coagulation factor III (thromboplastin, tissue factor) | -2.254660477 | 6.82E-65 |
| PCBP3 | poly(rC) binding protein 3 | -1.823792472 | 2.13E-64 |
| USP24 | ubiquitin specific peptidase 24 | -1.009199377 | 2.40E-64 |
| DSEL | dermatan sulfate epimerase-like | -1.003959719 | 5.08E-64 |
| ANKRD33B | ankyrin repeat domain 33B | -1.097409478 | 6.96E-64 |
| SEZ6L2 | seizure related 6 homolog (mouse)-like 2 | -1.685858415 | 4.84E-63 |
| AJUBA | ajuba LIM protein | -1.343485674 | 1.51E-62 |
| PERP | PERP, TP53 apoptosis effector | -1.149765165 | 2.06E-62 |
| ZFHX3 | zinc finger homeobox 3 | -1.284270519 | 3.56E-62 |
| TMEM47 | transmembrane protein 47 | -1.013101757 | 1.04E-61 |
| RASGRF2 | Ras protein-specific guanine nucleotide-releasing factor 2 | -3.334275642 | 1.15E-61 |
| ACSL3 | acyl-CoA synthetase long-chain family member 3 | -1.084185428 | 1.91E-61 |
| ARHGAP17 | Rho GTPase activating protein 17 | -1.066578844 | 2.92E-61 |
| MVK | mevalonate kinase | -1.752115474 | 4.59E-61 |
| GSTM2 | glutathione S-transferase mu 2 (muscle) | -1.372227061 | 3.32E-60 |
| IL13RA2 | interleukin 13 receptor, alpha 2 | -1.886139698 | 1.85E-59 |
| PCYT2 | phosphate cytidylyltransferase 2, ethanolamine | -1.271283311 | 5.47E-59 |
| PRKCA | protein kinase C, alpha | -1.050695383 | 6.05E-59 |
| PRKAR1B | protein kinase, cAMP-dependent, regulatory, type I, beta | -1.935494125 | 2.03E-58 |
| HSPA1A | heat shock 70kDa protein 1A | -1.093893799 | 2.45E-58 |
| HSPA1B | heat shock 70kDa protein 1B | -1.124365573 | 6.50E-58 |
| IRF4 | interferon regulatory factor 4 | -4.599532245 | 2.60E-57 |
| LOXL3 | lysyl oxidase-like 3 | -1.89379799 | 2.91E-57 |
| MICAL1 | microtubule associated monoxygenase, calponin and LIM domain containing 1 | -1.050454779 | 3.19E-57 |
| SKAP2 | src kinase associated phosphoprotein 2 | -1.22865839 | 1.80E-56 |
| FRMD3 | FERM domain containing 3 | -3.324245951 | 8.76E-56 |
| PRKD1 | protein kinase D1 | -1.736996239 | 3.70E-55 |
| SIPA1L1 | signal-induced proliferation-associated 1 like 1 | -1.210464773 | 4.56E-55 |
| FMN2 | formin 2 | -1.597376958 | 4.73E-55 |
| MEIS2 | Meis homeobox 2 | -1.175948702 | 5.12E-55 |
| MTSS1 | metastasis suppressor 1 | -1.570117746 | 5.61E-55 |
| ARRB1 | arrestin, beta 1 | -1.332199675 | 9.63E-55 |
| IGF2BP3 | insulin-like growth factor 2 mRNA binding protein 3 | -1.163509454 | 2.75E-54 |
| ACAT2 | acetyl-CoA acetyltransferase 2 | -1.742645424 | 4.47E-54 |
| ITGA6 | integrin, alpha 6 | -2.626406919 | 1.80E-53 |
| FAM189B | family with sequence similarity 189, member B | -1.173853228 | 3.13E-53 |
| POU5F1 | POU class 5 homeobox 1 | -6.267785255 | 1.28E-52 |
| CD36 | CD36 molecule (thrombospondin receptor) | -1.370388166 | 2.08E-52 |
| AIM1 | absent in melanoma 1 | -1.248776231 | 2.18E-52 |
| EPB41L1 | erythrocyte membrane protein band 4.1-like 1 | -1.137319938 | 7.42E-52 |
| ARID5A | AT rich interactive domain 5A (MRF1-like) | -1.285525051 | 1.01E-51 |
| KCNIP3 | Kv channel interacting protein 3, calsenilin | -1.554702749 | 1.25E-51 |
| C7orf41 | chromosome 7 open reading frame 41 | -1.121135651 | 1.78E-51 |
| GCNT1 | glucosaminyl (N-acetyl) transferase 1, core 2 | -1.510088746 | 1.36E-50 |
| HAND2 | heart and neural crest derivatives expressed 2 | -1.250399965 | 1.39E-50 |
| BCAM | basal cell adhesion molecule (Lutheran blood group) | -3.448873744 | 1.52E-50 |
| LOC100130872 | uncharacterized LOC100130872 | -2.254035949 | 3.75E-50 |
| PITX2 | paired-like homeodomain 2 | -1.563538843 | 5.04E-50 |
| B3GNT5 | UDP-GlcNAc:betaGal beta-1,3-N-acetylglucosaminyltransferase 5 | -1.706325612 | 1.54E-49 |
| HMGA2 | high mobility group AT-hook 2 | -1.572229319 | 3.20E-49 |
| SYNGR1 | synaptogyrin 1 | -1.497608594 | 3.98E-49 |
| DSP | desmoplakin | -1.779810215 | 4.08E-49 |
| ANKRD29 | ankyrin repeat domain 29 | -2.037822266 | 1.02E-48 |
| ENPEP | glutamyl aminopeptidase (aminopeptidase A) | -3.532817781 | 2.46E-48 |
| MAP7D3 | MAP7 domain containing 3 | -1.155436898 | 7.02E-48 |
| DOCK5 | dedicator of cytokinesis 5 | -1.218088624 | 1.55E-47 |
| GLIS3 | GLIS family zinc finger 3 | -1.436010103 | 2.06E-47 |
| ENDOD1 | endonuclease domain containing 1 | -1.302262219 | 6.72E-47 |
| AHR | aryl hydrocarbon receptor | -1.051700861 | 3.45E-46 |
| NUDT16 | nudix (nucleoside diphosphate linked moiety X)-type motif 16 | -1.023669344 | 7.37E-46 |
| JRK | jerky homolog (mouse) | -1.173586048 | 1.31E-45 |
| CD24 | CD24 molecule | -4.064686741 | 2.58E-45 |
| ATHL1 | ATH1, acid trehalase-like 1 (yeast) | -1.578888208 | 7.93E-45 |
| ANO4 | anoctamin 4 | -3.493679496 | 1.02E-44 |
| ACCN2 | amiloride-sensitive cation channel 2, neuronal | -1.973077587 | 1.08E-44 |
| GPRIN3 | GPRIN family member 3 | -3.503796994 | 1.49E-44 |
| ULK2 | unc-51-like kinase 2 (C. elegans) | -1.105608154 | 1.60E-44 |
| WNK4 | WNK lysine deficient protein kinase 4 | -5.690745775 | 1.62E-44 |
| LIFR | leukemia inhibitory factor receptor alpha | -1.31916231 | 2.03E-44 |
| CD4 | CD4 molecule | -4.252264379 | 3.34E-44 |
| EBF1 | early B-cell factor 1 | -1.096249183 | 3.55E-44 |
| GALNT12 | UDP-N-acetyl-alpha-D-galactosamine:polypeptide N-acetylgalactosaminyltransferase 12 (GalNAc-T12) | -2.056514713 | 6.33E-44 |
| DRP2 | dystrophin related protein 2 | -4.343199996 | 1.15E-43 |
| FMNL2 | formin-like 2 | -1.026685852 | 3.42E-43 |
| BVES | blood vessel epicardial substance | -1.161139533 | 3.80E-43 |
| PODXL | podocalyxin-like | -1.69168787 | 3.93E-43 |
| RAB3B | RAB3B, member RAS oncogene family | -1.097274594 | 5.29E-43 |
| SEMA7A | semaphorin 7A, GPI membrane anchor (John Milton Hagen blood group) | -1.316411257 | 8.22E-43 |
| PTCH1 | patched 1 | -2.211124236 | 1.17E-42 |
| LOC728392 | uncharacterized LOC728392 | -1.056548742 | 1.19E-42 |
| CRIP1 | cysteine-rich protein 1 (intestinal) | -1.822972253 | 2.11E-42 |
| GSTM5 | glutathione S-transferase mu 5 | -1.183220805 | 5.80E-42 |
| SMPDL3A | sphingomyelin phosphodiesterase, acid-like 3A | -1.422053513 | 3.82E-41 |
| PLXNB3 | plexin B3 | -1.100081773 | 5.05E-41 |
| ATOH8 | atonal homolog 8 (Drosophila) | -2.07680575 | 6.92E-41 |
| KCNQ3 | potassium voltage-gated channel, KQT-like subfamily, member 3 | -2.308437075 | 8.08E-41 |
| RHOBTB1 | Rho-related BTB domain containing 1 | -1.135795002 | 1.54E-40 |
| CUBN | cubilin (intrinsic factor-cobalamin receptor) | -1.613385356 | 1.72E-40 |
| ARNT2 | aryl-hydrocarbon receptor nuclear translocator 2 | -1.26510033 | 2.00E-40 |
| PGAM4 | phosphoglycerate mutase family member 4 | -6.371765429 | 2.41E-40 |
| KRT19 | keratin 19 | -3.738852341 | 5.51E-40 |
| LAMA5 | laminin, alpha 5 | -1.024471855 | 9.94E-40 |
| PCDH17 | protocadherin 17 | -7.128635992 | 3.72E-39 |
| MICA | MHC class I polypeptide-related sequence A | -1.285058439 | 5.34E-39 |
| HIP1R | huntingtin interacting protein 1 related | -1.384054209 | 6.17E-39 |
| ACSS1 | acyl-CoA synthetase short-chain family member 1 | -1.329914057 | 7.02E-39 |
| DKK1 | dickkopf 1 homolog (Xenopus laevis) | -1.254678637 | 9.46E-39 |
| PRUNE2 | prune homolog 2 (Drosophila) | -1.055155311 | 1.26E-38 |
| LRP11 | low density lipoprotein receptor-related protein 11 | -1.016822786 | 2.54E-38 |
| GATA2 | GATA binding protein 2 | -3.572969689 | 3.56E-38 |
| FBXO32 | F-box protein 32 | -1.00341253 | 7.08E-38 |
| C6orf132 | chromosome 6 open reading frame 132 | -1.567814942 | 1.01E-37 |
| ABCA7 | ATP-binding cassette, sub-family A (ABC1), member 7 | -1.330383469 | 1.12E-37 |
| RAB11FIP4 | RAB11 family interacting protein 4 (class II) | -3.227930543 | 2.26E-37 |
| SCN9A | sodium channel, voltage-gated, type IX, alpha subunit | -2.536777231 | 2.69E-37 |
| ULBP1 | UL16 binding protein 1 | -2.359882647 | 5.62E-37 |
| MAMLD1 | mastermind-like domain containing 1 | -1.381993148 | 6.97E-37 |
| FLJ23867 | uncharacterized protein FLJ23867 | -1.363347626 | 7.76E-37 |
| SHMT1 | serine hydroxymethyltransferase 1 (soluble) | -1.803420119 | 1.03E-36 |
| L1TD1 | LINE-1 type transposase domain containing 1 | -9.400404793 | 1.06E-36 |
| PNPLA3 | patatin-like phospholipase domain containing 3 | -1.765346874 | 1.55E-36 |
| FAM83H | family with sequence similarity 83, member H | -2.783184094 | 1.64E-36 |
| PPARG | peroxisome proliferator-activated receptor gamma | -1.242019812 | 1.96E-36 |
| GALNT6 | UDP-N-acetyl-alpha-D-galactosamine:polypeptide N-acetylgalactosaminyltransferase 6 (GalNAc-T6) | -1.397977331 | 3.72E-36 |
| ADRA1D | adrenergic, alpha-1D-, receptor | -3.327825234 | 6.36E-36 |
| PRRT2 | proline-rich transmembrane protein 2 | -1.275455624 | 1.83E-35 |
| APCDD1L | adenomatosis polyposis coli down-regulated 1-like | -1.352468827 | 1.90E-35 |
| MAOA | monoamine oxidase A | -1.647402823 | 1.97E-35 |
| SUSD1 | sushi domain containing 1 | -1.135733406 | 3.34E-35 |
| MLPH | melanophilin | -1.282768982 | 4.06E-35 |
| DOCK2 | dedicator of cytokinesis 2 | -2.2895607 | 1.02E-34 |
| SERINC2 | serine incorporator 2 | -1.181387999 | 1.40E-34 |
| ZBTB16 | zinc finger and BTB domain containing 16 | -5.647637152 | 1.64E-34 |
| FLJ42627 | uncharacterized LOC645644 | -1.082029305 | 2.60E-34 |
| FAM155A | family with sequence similarity 155, member A | -2.246628551 | 3.74E-34 |
| STON1 | stonin 1 | -1.169869873 | 1.18E-33 |
| LEPR | leptin receptor | -1.193905057 | 5.97E-33 |
| SV2A | synaptic vesicle glycoprotein 2A | -1.022536568 | 6.89E-33 |
| C22orf23 | chromosome 22 open reading frame 23 | -1.596675989 | 2.31E-32 |
| LOC283392 | uncharacterized LOC283392 | -2.714957904 | 2.77E-32 |
| SLC8A1 | solute carrier family 8 (sodium/calcium exchanger), member 1 | -2.140348001 | 3.39E-32 |
| GYG2 | glycogenin 2 | -4.124209076 | 3.69E-32 |
| GRIN3B | glutamate receptor, ionotropic, N-methyl-D-aspartate 3B | -2.794805128 | 3.76E-32 |
| FLT1 | fms-related tyrosine kinase 1 (vascular endothelial growth factor/vascular permeability factor receptor) | -3.281444029 | 7.42E-32 |
| BDNF | brain-derived neurotrophic factor | -3.53271756 | 7.96E-32 |
| CACNG7 | calcium channel, voltage-dependent, gamma subunit 7 | -3.72421533 | 9.01E-32 |
| HOXB5 | homeobox B5 | -2.213461629 | 9.72E-32 |
| ADAMTS15 | ADAM metallopeptidase with thrombospondin type 1 motif, 15 | -1.068800531 | 1.98E-31 |
| LINC00341 | long intergenic non-protein coding RNA 341 | -1.123256944 | 3.16E-31 |
| ACCS | 1-aminocyclopropane-1-carboxylate synthase homolog (Arabidopsis)(non-functional) | -1.627814593 | 3.87E-31 |
| C11orf41 | chromosome 11 open reading frame 41 | -1.840475785 | 5.31E-31 |
| DPPA4 | developmental pluripotency associated 4 | -9.124503834 | 5.38E-31 |
| ISYNA1 | inositol-3-phosphate synthase 1 | -1.438001417 | 7.65E-31 |
| ZNF462 | zinc finger protein 462 | -1.221401417 | 8.87E-31 |
| LOC285084 | uncharacterized LOC285084 | -1.238746752 | 1.78E-30 |
| SYT14 | synaptotagmin XIV | -2.892591255 | 2.49E-30 |
| DNMT3B | DNA (cytosine-5-)-methyltransferase 3 beta | -1.762977485 | 7.75E-30 |
| FGD4 | FYVE, RhoGEF and PH domain containing 4 | -1.283803868 | 8.01E-30 |
| TJP2 | tight junction protein 2 (zona occludens 2) | -1.0089161 | 1.09E-29 |
| LOC389906 | zinc finger protein 839 pseudogene | -1.411832874 | 1.11E-29 |
| ELOVL6 | ELOVL fatty acid elongase 6 | -1.250926573 | 1.23E-29 |
| C3orf64 | chromosome 3 open reading frame 64 | -1.18555831 | 1.65E-29 |
| FER1L4 | fer-1-like 4 (C. elegans) pseudogene | -2.104619313 | 1.80E-29 |
| VSIG10 | V-set and immunoglobulin domain containing 10 | -1.185224911 | 1.83E-29 |
| MCF2L | MCF.2 cell line derived transforming sequence-like | -5.460787995 | 2.04E-29 |
| EPDR1 | ependymin related protein 1 (zebrafish) | -1.089331957 | 2.12E-29 |
| ERMP1 | endoplasmic reticulum metallopeptidase 1 | -1.056715775 | 2.63E-29 |
| TMEM97 | transmembrane protein 97 | -1.212357374 | 3.49E-29 |
| RAB11FIP1 | RAB11 family interacting protein 1 (class I) | -1.781367496 | 3.91E-29 |
| LOC100507053 | uncharacterized LOC100507053 | -1.363817491 | 5.81E-29 |
| ST6GAL1 | ST6 beta-galactosamide alpha-2,6-sialyltranferase 1 | -2.376625946 | 6.63E-29 |
| SYNPO | synaptopodin | -1.051350243 | 1.05E-28 |
| LY6K | lymphocyte antigen 6 complex, locus K | -2.437061888 | 1.24E-28 |
| ABCA5 | ATP-binding cassette, sub-family A (ABC1), member 5 | -1.064545769 | 1.47E-28 |
| FAM101B | family with sequence similarity 101, member B | -1.26267548 | 1.54E-28 |
| GLIS1 | GLIS family zinc finger 1 | -2.538900607 | 1.73E-28 |
| TIPARP | TCDD-inducible poly(ADP-ribose) polymerase | -1.05906064 | 4.57E-28 |
| VWA1 | von Willebrand factor A domain containing 1 | -1.382599124 | 5.95E-28 |
| HTR7 | 5-hydroxytryptamine (serotonin) receptor 7 (adenylate cyclase-coupled) | -4.2330384 | 9.08E-28 |
| GDF5 | growth differentiation factor 5 | -1.172910646 | 1.66E-27 |
| APBB1IP | amyloid beta (A4) precursor protein-binding, family B, member 1 interacting protein | -1.179545858 | 3.25E-27 |
| LRRC8C | leucine rich repeat containing 8 family, member C | -1.406821143 | 4.66E-27 |
| MMP24 | matrix metallopeptidase 24 (membrane-inserted) | -1.275181182 | 4.97E-27 |
| B7H6 | B7 homolog 6 | -1.449537989 | 5.07E-27 |
| PRKAG2 | protein kinase, AMP-activated, gamma 2 non-catalytic subunit | -1.465414442 | 5.52E-27 |
| LOC730101 | uncharacterized LOC730101 | -1.059766754 | 6.90E-27 |
| CHRM2 | cholinergic receptor, muscarinic 2 | -6.520067674 | 7.48E-27 |
| CPA4 | carboxypeptidase A4 | -2.440322066 | 1.07E-26 |
| PRKX | protein kinase, X-linked | -1.066862582 | 1.57E-26 |
| SRRM3 | serine/arginine repetitive matrix 3 | -2.750284737 | 2.73E-26 |
| DES | desmin | -2.850262102 | 6.34E-26 |
| C12orf53 | chromosome 12 open reading frame 53 | -2.916060161 | 1.65E-25 |
| F11R | F11 receptor | -2.209247282 | 2.91E-25 |
| COL10A1 | collagen, type X, alpha 1 | -3.258590185 | 3.93E-25 |
| L3MBTL3 | l(3)mbt-like 3 (Drosophila) | -1.168595225 | 5.33E-25 |
| RAC2 | ras-related C3 botulinum toxin substrate 2 (rho family, small GTP binding protein Rac2) | -1.229387691 | 8.98E-25 |
| KIAA0922 | KIAA0922 | -1.131934685 | 1.06E-24 |
| NTM | neurotrimin | -1.913403399 | 1.12E-24 |
| ESRG | embryonic stem cell related (non-protein coding) | -4.838843337 | 1.44E-24 |
| ARHGAP6 | Rho GTPase activating protein 6 | -1.113168003 | 2.53E-24 |
| MIR143HG | MIR143 host gene (non-protein coding) | -1.374732626 | 2.67E-24 |
| PRICKLE4 | prickle homolog 4 (Drosophila) | -1.853031746 | 3.65E-24 |
| MAP1LC3A | microtubule-associated protein 1 light chain 3 alpha | -1.016307157 | 3.80E-24 |
| RIMS2 | regulating synaptic membrane exocytosis 2 | -2.543929555 | 4.45E-24 |
| AQP3 | aquaporin 3 (Gill blood group) | -1.677375217 | 4.62E-24 |
| RASSF3 | Ras association (RalGDS/AF-6) domain family member 3 | -1.159828419 | 6.81E-24 |
| LINC00346 | long intergenic non-protein coding RNA 346 | -1.643922224 | 8.72E-24 |
| CLDN6 | claudin 6 | -5.538976728 | 1.61E-23 |
| HORMAD1 | HORMA domain containing 1 | -5.507855316 | 4.24E-23 |
| RNF175 | ring finger protein 175 | -2.276287568 | 4.74E-23 |
| C5orf65 | chromosome 5 open reading frame 65 | -2.134026301 | 5.18E-23 |
| LOC79015 | uncharacterized LOC79015 | -2.615887797 | 5.68E-23 |
| FBXO41 | F-box protein 41 | -1.542729584 | 8.88E-23 |
| SRGN | serglycin | -1.948717058 | 1.82E-22 |
| POPDC3 | popeye domain containing 3 | -1.080971652 | 3.36E-22 |
| HOXB8 | homeobox B8 | -1.606651917 | 3.59E-22 |
| TNIK | TRAF2 and NCK interacting kinase | -1.301941247 | 3.80E-22 |
| PTPRQ | protein tyrosine phosphatase, receptor type, Q | -5.01858866 | 4.36E-22 |
| AQP7 | aquaporin 7 | -3.711442632 | 4.66E-22 |
| DHRS3 | dehydrogenase/reductase (SDR family) member 3 | -1.18841527 | 6.06E-22 |
| NFE2L3 | nuclear factor (erythroid-derived 2)-like 3 | -1.585525609 | 6.93E-22 |
| PCYOX1L | prenylcysteine oxidase 1 like | -1.305699692 | 9.98E-22 |
| LEPREL1 | leprecan-like 1 | -2.87009595 | 1.14E-21 |
| ECHDC3 | enoyl CoA hydratase domain containing 3 | -2.193454648 | 2.76E-21 |
| LOC100129387 | uncharacterized LOC100129387 | -1.36259469 | 2.93E-21 |
| HAND1 | heart and neural crest derivatives expressed 1 | -4.624682494 | 3.82E-21 |
| EIF5A2 | eukaryotic translation initiation factor 5A2 | -1.058433573 | 4.77E-21 |
| IL33 | interleukin 33 | -2.912444062 | 5.32E-21 |
| SERPINB7 | serpin peptidase inhibitor, clade B (ovalbumin), member 7 | -2.150808469 | 5.78E-21 |
| KIF1A | kinesin family member 1A | -5.332645581 | 1.29E-20 |
| HSPB3 | heat shock 27kDa protein 3 | -2.588149885 | 1.75E-20 |
| CSMD2 | CUB and Sushi multiple domains 2 | -1.352339561 | 1.85E-20 |
| PTGER2 | prostaglandin E receptor 2 (subtype EP2), 53kDa | -1.171497638 | 3.73E-20 |
| SLC16A5 | solute carrier family 16, member 5 (monocarboxylic acid transporter 6) | -1.296964421 | 3.95E-20 |
| B3GALT4 | UDP-Gal:betaGlcNAc beta 1,3-galactosyltransferase, polypeptide 4 | -1.543678438 | 6.67E-20 |
| EBF2 | early B-cell factor 2 | -1.544071717 | 9.18E-20 |
| LCNL1 | lipocalin-like 1 | -1.973071038 | 1.01E-19 |
| HDHD1 | haloacid dehalogenase-like hydrolase domain containing 1 | -1.02731379 | 1.09E-19 |
| GABRB3 | gamma-aminobutyric acid (GABA) A receptor, beta 3 | -2.556171557 | 1.10E-19 |
| TIAM2 | T-cell lymphoma invasion and metastasis 2 | -1.776377096 | 1.34E-19 |
| MGAT5B | mannosyl (alpha-1,6-)-glycoprotein beta-1,6-N-acetyl-glucosaminyltransferase, isozyme B | -2.969116031 | 1.51E-19 |
| RNF157 | ring finger protein 157 | -1.058269346 | 1.63E-19 |
| FOXF1 | forkhead box F1 | -2.902706679 | 1.92E-19 |
| SHANK3 | SH3 and multiple ankyrin repeat domains 3 | -1.008448808 | 3.66E-19 |
| HOXB7 | homeobox B7 | -1.073187309 | 5.49E-19 |
| OXCT2 | 3-oxoacid CoA transferase 2 | -2.205314533 | 6.11E-19 |
| TUFT1 | tuftelin 1 | -1.466183877 | 6.28E-19 |
| ANGPT2 | angiopoietin 2 | -3.603406546 | 6.49E-19 |
| C1orf213 | chromosome 1 open reading frame 213 | -1.641313217 | 7.81E-19 |
| FBXO27 | F-box protein 27 | -1.374283029 | 8.70E-19 |
| RGNEF | 190 kDa guanine nucleotide exchange factor | -1.607385696 | 1.40E-18 |
| MRVI1 | murine retrovirus integration site 1 homolog | -1.747485546 | 1.43E-18 |
| LOC440300 | chondroitin sulfate proteoglycan 4 pseudogene | -1.084432698 | 1.48E-18 |
| STAR | steroidogenic acute regulatory protein | -3.617745259 | 2.28E-18 |
| ITPRIPL1 | inositol 1,4,5-trisphosphate receptor interacting protein-like 1 | -2.438167524 | 2.48E-18 |
| AGAP11 | ankyrin repeat and GTPase domain Arf GTPase activating protein 11 | -1.708790432 | 2.52E-18 |
| PTPRJ | protein tyrosine phosphatase, receptor type, J | -1.364890699 | 2.94E-18 |
| CAMK2A | calcium/calmodulin-dependent protein kinase II alpha | -2.059881842 | 3.97E-18 |
| GREB1L | growth regulation by estrogen in breast cancer-like | -1.341232126 | 4.09E-18 |
| ANKDD1A | ankyrin repeat and death domain containing 1A | -1.068370174 | 4.80E-18 |
| NOX4 | NADPH oxidase 4 | -2.362464592 | 5.72E-18 |
| FERMT1 | fermitin family member 1 | -1.742916273 | 5.72E-18 |
| GPC4 | glypican 4 | -1.184496299 | 6.96E-18 |
| SIX1 | SIX homeobox 1 | -1.140540462 | 8.74E-18 |
| PGM2 | phosphoglucomutase 2 | -1.05952295 | 1.22E-17 |
| APBA2 | amyloid beta (A4) precursor protein-binding, family A, member 2 | -2.06845534 | 1.63E-17 |
| FAM167A | family with sequence similarity 167, member A | -2.976624524 | 1.85E-17 |
| PAQR8 | progestin and adipoQ receptor family member VIII | -1.209692862 | 2.17E-17 |
| COL9A3 | collagen, type IX, alpha 3 | -2.375952493 | 2.62E-17 |
| C9orf47 | chromosome 9 open reading frame 47 | -3.556584787 | 2.72E-17 |
| NPB | neuropeptide B | -2.357294477 | 3.38E-17 |
| CLMN | calmin (calponin-like, transmembrane) | -2.71760661 | 6.80E-17 |
| CDH24 | cadherin 24, type 2 | -1.544787489 | 7.86E-17 |
| EFNB3 | ephrin-B3 | -2.028448429 | 8.07E-17 |
| KIAA1683 | KIAA1683 | -2.350750499 | 8.92E-17 |
| NR4A3 | nuclear receptor subfamily 4, group A, member 3 | -1.167362265 | 1.42E-16 |
| SPESP1 | sperm equatorial segment protein 1 | -3.051341437 | 1.55E-16 |
| HCN2 | hyperpolarization activated cyclic nucleotide-gated potassium channel 2 | -1.767289097 | 1.56E-16 |
| PLEKHA6 | pleckstrin homology domain containing, family A member 6 | -2.333306759 | 3.15E-16 |
| KLHL29 | kelch-like 29 (Drosophila) | -1.075672591 | 3.16E-16 |
| SRD5A1 | steroid-5-alpha-reductase, alpha polypeptide 1 (3-oxo-5 alpha-steroid delta 4-dehydrogenase alpha 1) | -1.029311581 | 3.44E-16 |
| NUDT11 | nudix (nucleoside diphosphate linked moiety X)-type motif 11 | -1.19142782 | 4.11E-16 |
| ANXA3 | annexin A3 | -3.534920773 | 4.13E-16 |
| EVI2B | ecotropic viral integration site 2B | -1.874978128 | 5.01E-16 |
| TMEM51 | transmembrane protein 51 | -1.281404956 | 6.67E-16 |
| KRT8 | keratin 8 | -4.494318837 | 8.64E-16 |
| PAPLN | papilin, proteoglycan-like sulfated glycoprotein | -1.216945451 | 9.12E-16 |
| SLC4A3 | solute carrier family 4, anion exchanger, member 3 | -1.547335926 | 1.19E-15 |
| LPPR4 | lipid phosphate phosphatase-related protein type 4 | -1.369207035 | 1.22E-15 |
| MID2 | midline 2 | -1.212624013 | 2.10E-15 |
| EVI2A | ecotropic viral integration site 2A | -1.102347387 | 2.11E-15 |
| MST1 | macrophage stimulating 1 (hepatocyte growth factor-like) | -1.330169509 | 2.15E-15 |
| HOXC4 | homeobox C4 | -1.040252799 | 2.35E-15 |
| MAPK13 | mitogen-activated protein kinase 13 | -1.141408616 | 2.99E-15 |
| MET | met proto-oncogene (hepatocyte growth factor receptor) | -1.336241301 | 3.85E-15 |
| PM20D2 | peptidase M20 domain containing 2 | -1.016756384 | 3.97E-15 |
| TMEM154 | transmembrane protein 154 | -1.441121811 | 4.45E-15 |
| ADAM11 | ADAM metallopeptidase domain 11 | -2.583910105 | 6.36E-15 |
| LRRC8B | leucine rich repeat containing 8 family, member B | -1.268414465 | 8.74E-15 |
| LOC652276 | potassium channel tetramerisation domain containing 5 pseudogene | -1.120763548 | 8.85E-15 |
| TMCC3 | transmembrane and coiled-coil domain family 3 | -2.394544019 | 9.61E-15 |
| SEC31B | SEC31 homolog B (S. cerevisiae) | -1.025326848 | 9.67E-15 |
| SNCG | synuclein, gamma (breast cancer-specific protein 1) | -2.030891317 | 1.28E-14 |
| TMEM91 | transmembrane protein 91 | -1.467858104 | 1.56E-14 |
| GIPC3 | GIPC PDZ domain containing family, member 3 | -3.651392641 | 2.41E-14 |
| MECOM | MDS1 and EVI1 complex locus | -2.139842756 | 4.67E-14 |
| ELFN2 | extracellular leucine-rich repeat and fibronectin type III domain containing 2 | -2.489961465 | 5.00E-14 |
| SEC14L4 | SEC14-like 4 (S. cerevisiae) | -3.490331488 | 6.09E-14 |
| NFATC2 | nuclear factor of activated T-cells, cytoplasmic, calcineurin-dependent 2 | -3.470449529 | 6.14E-14 |
| MARCH4 | membrane-associated ring finger (C3HC4) 4 | -1.075097658 | 6.72E-14 |
| NCAM1 | neural cell adhesion molecule 1 | -1.015273741 | 8.02E-14 |
| IL11 | interleukin 11 | -2.380975842 | 1.04E-13 |
| FAM60A | family with sequence similarity 60, member A | -1.382323959 | 1.43E-13 |
| POLR3G | polymerase (RNA) III (DNA directed) polypeptide G (32kD) | -1.920726058 | 2.16E-13 |
| GRIN2D | glutamate receptor, ionotropic, N-methyl D-aspartate 2D | -1.092877095 | 2.64E-13 |
| SOCS2 | suppressor of cytokine signaling 2 | -1.142326162 | 3.08E-13 |
| ADAMTS6 | ADAM metallopeptidase with thrombospondin type 1 motif, 6 | -1.006356724 | 3.48E-13 |
| LOC401093 | uncharacterized LOC401093 | -1.584066308 | 3.87E-13 |
| HSD17B7 | hydroxysteroid (17-beta) dehydrogenase 7 | -1.174714401 | 3.89E-13 |
| CACNG8 | calcium channel, voltage-dependent, gamma subunit 8 | -3.29112156 | 4.63E-13 |
| TMEM130 | transmembrane protein 130 | -3.256559468 | 4.69E-13 |
| BAIAP2L1 | BAI1-associated protein 2-like 1 | -1.666270941 | 4.73E-13 |
| B4GALNT4 | beta-1,4-N-acetyl-galactosaminyl transferase 4 | -1.610210684 | 5.53E-13 |
| MATN3 | matrilin 3 | -2.454854083 | 6.88E-13 |
| KCNS2 | potassium voltage-gated channel, delayed-rectifier, subfamily S, member 2 | -1.032470979 | 8.07E-13 |
| SFRP4 | secreted frizzled-related protein 4 | -2.409485546 | 1.23E-12 |
| HS6ST3 | heparan sulfate 6-O-sulfotransferase 3 | -3.221806808 | 1.28E-12 |
| LINC00087 | long intergenic non-protein coding RNA 87 | -1.813359768 | 1.42E-12 |
| BDH1 | 3-hydroxybutyrate dehydrogenase, type 1 | -1.79671742 | 1.64E-12 |
| ADORA1 | adenosine A1 receptor | -1.510889835 | 2.18E-12 |
| SCARNA9 | small Cajal body-specific RNA 9 | -4.014094511 | 3.02E-12 |
| SIX2 | SIX homeobox 2 | -2.074902861 | 3.18E-12 |
| INHBA | inhibin, beta A | -1.344454687 | 3.42E-12 |
| SCAMP5 | secretory carrier membrane protein 5 | -2.282862728 | 4.12E-12 |
| CLTCL1 | clathrin, heavy chain-like 1 | -1.087352673 | 4.24E-12 |
| HSBP1L1 | heat shock factor binding protein 1-like 1 | -1.220633087 | 6.00E-12 |
| INSC | inscuteable homolog (Drosophila) | -2.623003533 | 6.61E-12 |
| SLC19A3 | solute carrier family 19, member 3 | -2.598862855 | 9.61E-12 |
| SYT1 | synaptotagmin I | -2.474373209 | 1.10E-11 |
| SARDH | sarcosine dehydrogenase | -2.940035337 | 1.25E-11 |
| E2F7 | E2F transcription factor 7 | -1.444958838 | 1.35E-11 |
| PKN3 | protein kinase N3 | -1.178051275 | 1.63E-11 |
| NACAD | NAC alpha domain containing | -1.15585657 | 1.86E-11 |
| NHSL1 | NHS-like 1 | -1.022102196 | 1.95E-11 |
| GNG7 | guanine nucleotide binding protein (G protein), gamma 7 | -1.807481573 | 2.59E-11 |
| CTF1 | cardiotrophin 1 | -1.019257458 | 3.34E-11 |
| FAM46B | family with sequence similarity 46, member B | -2.597753952 | 4.61E-11 |
| JAG2 | jagged 2 | -1.7664678 | 5.37E-11 |
| GFOD1 | glucose-fructose oxidoreductase domain containing 1 | -1.707927798 | 7.81E-11 |
| GPRC5A | G protein-coupled receptor, family C, group 5, member A | -2.035389992 | 7.95E-11 |
| CELSR3 | cadherin, EGF LAG seven-pass G-type receptor 3 (flamingo homolog, Drosophila) | -1.709340114 | 9.02E-11 |
| NETO2 | neuropilin (NRP) and tolloid (TLL)-like 2 | -1.326590515 | 1.38E-10 |
| C10orf114 | chromosome 10 open reading frame 114 | -1.400152164 | 1.59E-10 |
| RAPGEF3 | Rap guanine nucleotide exchange factor (GEF) 3 | -1.141316017 | 1.62E-10 |
| PRSS53 | protease, serine, 53 | -1.2946429 | 1.88E-10 |
| LTK | leukocyte receptor tyrosine kinase | -2.415526386 | 2.03E-10 |
| SNCA | synuclein, alpha (non A4 component of amyloid precursor) | -1.251238498 | 2.11E-10 |
| CLDN7 | claudin 7 | -2.637574632 | 2.59E-10 |
| NMNAT2 | nicotinamide nucleotide adenylyltransferase 2 | -2.396238574 | 3.04E-10 |
| LOC388630 | UPF0632 protein A | -2.212092455 | 3.12E-10 |
| CDCA7 | cell division cycle associated 7 | -1.803709269 | 3.99E-10 |
| C4orf49 | chromosome 4 open reading frame 49 | -1.504689998 | 4.39E-10 |
| KBTBD10 | kelch repeat and BTB (POZ) domain containing 10 | -1.5418687 | 4.45E-10 |
| COL9A2 | collagen, type IX, alpha 2 | -2.086913819 | 4.71E-10 |
| PPFIA4 | protein tyrosine phosphatase, receptor type, f polypeptide (PTPRF), interacting protein (liprin), alpha 4 | -1.091934988 | 5.65E-10 |
| SDPR | serum deprivation response | -1.314562492 | 6.45E-10 |
| TMEM155 | transmembrane protein 155 | -2.255499969 | 6.56E-10 |
| CCDC136 | coiled-coil domain containing 136 | -1.156853116 | 6.57E-10 |
| HVCN1 | hydrogen voltage-gated channel 1 | -1.933265808 | 7.14E-10 |
| LXN | latexin | -2.159597077 | 9.95E-10 |
| C1orf115 | chromosome 1 open reading frame 115 | -1.76254099 | 1.00E-09 |
| B4GALT6 | UDP-Gal:betaGlcNAc beta 1,4- galactosyltransferase, polypeptide 6 | -1.094435961 | 1.04E-09 |
| FLVCR1 | feline leukemia virus subgroup C cellular receptor 1 | -1.144945101 | 1.06E-09 |
| LOC100507588 | uncharacterized protein LOC100507588 | -1.761326507 | 1.28E-09 |
| PTPRZ1 | protein tyrosine phosphatase, receptor-type, Z polypeptide 1 | -2.294421649 | 1.40E-09 |
| ACVR2B | activin A receptor, type IIB | -1.065360451 | 1.91E-09 |
| PRG4 | proteoglycan 4 | -2.330562238 | 1.99E-09 |
| CENPV | centromere protein V | -2.112299773 | 2.23E-09 |
| FLJ10661 | family with sequence similarity 86, member A pseudogene | -2.198323773 | 2.26E-09 |
| PGAM2 | phosphoglycerate mutase 2 (muscle) | -1.940564871 | 2.30E-09 |
| RASL11A | RAS-like, family 11, member A | -1.632503502 | 2.43E-09 |
| TSTD1 | thiosulfate sulfurtransferase (rhodanese)-like domain containing 1 | -1.815665684 | 2.45E-09 |
| CYP3A5 | cytochrome P450, family 3, subfamily A, polypeptide 5 | -2.227806342 | 2.93E-09 |
| BCAS4 | breast carcinoma amplified sequence 4 | -1.068023906 | 3.41E-09 |
| SH3D21 | SH3 domain containing 21 | -1.277006149 | 3.90E-09 |
| PTPRD | protein tyrosine phosphatase, receptor type, D | -1.632110136 | 4.18E-09 |
| ROBO4 | roundabout homolog 4, magic roundabout (Drosophila) | -1.653221034 | 4.31E-09 |
| C6orf174 | chromosome 6 open reading frame 174 | -1.069426652 | 4.42E-09 |
| LGI4 | leucine-rich repeat LGI family, member 4 | -1.640552833 | 5.45E-09 |
| MGST2 | microsomal glutathione S-transferase 2 | -1.179039982 | 6.79E-09 |
| ICAM5 | intercellular adhesion molecule 5, telencephalin | -1.667950287 | 7.97E-09 |
| RTN4RL1 | reticulon 4 receptor-like 1 | -1.418403616 | 7.97E-09 |
| ABCA10 | ATP-binding cassette, sub-family A (ABC1), member 10 | -1.278860445 | 8.45E-09 |
| TRPC4 | transient receptor potential cation channel, subfamily C, member 4 | -1.65720792 | 8.47E-09 |
| C16orf74 | chromosome 16 open reading frame 74 | -1.38953983 | 8.72E-09 |
| RDH5 | retinol dehydrogenase 5 (11-cis/9-cis) | -1.289371983 | 8.75E-09 |
| EFCAB4B | EF-hand calcium binding domain 4B | -1.128529591 | 9.13E-09 |
| SLMO1 | slowmo homolog 1 (Drosophila) | -1.217841684 | 1.09E-08 |
| CHD7 | chromodomain helicase DNA binding protein 7 | -1.675195094 | 1.16E-08 |
| LINC00263 | long intergenic non-protein coding RNA 263 | -1.355314332 | 1.43E-08 |
| OPRL1 | opiate receptor-like 1 | -1.461021096 | 1.61E-08 |
| HOXC5 | homeobox C5 | -1.278249125 | 1.66E-08 |
| ARHGEF4 | Rho guanine nucleotide exchange factor (GEF) 4 | -1.545394653 | 1.78E-08 |
| AMIGO2 | adhesion molecule with Ig-like domain 2 | -1.30785818 | 1.94E-08 |
| PTCH2 | patched 2 | -1.794317929 | 3.72E-08 |
| OTUB2 | OTU domain, ubiquitin aldehyde binding 2 | -1.49839891 | 3.96E-08 |
| LIN7A | lin-7 homolog A (C. elegans) | -1.468884946 | 4.41E-08 |
| FLJ41484 | uncharacterized LOC650669 | -1.223788008 | 5.25E-08 |
| ASPHD1 | aspartate beta-hydroxylase domain containing 1 | -1.200114293 | 5.75E-08 |
| LOC100505633 | uncharacterized LOC100505633 | -1.386889884 | 7.24E-08 |
| SGK223 | homolog of rat pragma of Rnd2 | -1.784204782 | 7.52E-08 |
| IL31RA | interleukin 31 receptor A | -1.993232452 | 7.77E-08 |
| UNC13D | unc-13 homolog D (C. elegans) | -2.180838534 | 1.35E-07 |
| LOC81691 | exonuclease NEF-sp | -1.294840108 | 1.36E-07 |
| GSDMB | gasdermin B | -1.185516089 | 1.50E-07 |
| RBP5 | retinol binding protein 5, cellular | -1.523412249 | 1.53E-07 |
| CCDC85A | coiled-coil domain containing 85A | -1.034741711 | 2.02E-07 |
| SLC13A3 | solute carrier family 13 (sodium-dependent dicarboxylate transporter), member 3 | -1.068320989 | 2.05E-07 |
| CCDC88B | coiled-coil domain containing 88B | -1.501313974 | 2.19E-07 |
| TUBB2B | tubulin, beta 2B class IIb | -1.094199422 | 2.64E-07 |
| ST8SIA4 | ST8 alpha-N-acetyl-neuraminide alpha-2,8-sialyltransferase 4 | -1.497580627 | 2.79E-07 |
| CEND1 | cell cycle exit and neuronal differentiation 1 | -1.108859297 | 3.95E-07 |
| SEMA4G | sema domain, immunoglobulin domain (Ig), transmembrane domain (TM) and short cytoplasmic domain, (semaphorin) 4G | -1.211030891 | 4.88E-07 |
| WHAMMP2 | WAS protein homolog associated with actin, golgi membranes and microtubules pseudogene 2 | -1.043710151 | 4.92E-07 |
| ZNF793 | zinc finger protein 793 | -1.980864121 | 5.03E-07 |
| RASL10B | RAS-like, family 10, member B | -1.232624259 | 5.44E-07 |
| ALS2CR11 | amyotrophic lateral sclerosis 2 (juvenile) chromosome region, candidate 11 | -1.622644512 | 6.00E-07 |
| KATNAL2 | katanin p60 subunit A-like 2 | -1.010335696 | 6.08E-07 |
| PAIP2B | poly(A) binding protein interacting protein 2B | -1.740039331 | 6.79E-07 |
| PALMD | palmdelphin | -1.289055908 | 7.23E-07 |
| LOC151534 | uncharacterized LOC151534 | -1.007266751 | 8.20E-07 |
| ANKRD53 | ankyrin repeat domain 53 | -1.451640461 | 1.06E-06 |
| TOX | thymocyte selection-associated high mobility group box | -1.398697573 | 1.20E-06 |
| ATP8A1 | ATPase, aminophospholipid transporter (APLT), class I, type 8A, member 1 | -1.078111133 | 1.25E-06 |
| HOXA3 | homeobox A3 | -1.106825069 | 1.57E-06 |
| DNALI1 | dynein, axonemal, light intermediate chain 1 | -1.182693189 | 1.64E-06 |
| MARC2 | mitochondrial amidoxime reducing component 2 | -1.003077365 | 1.65E-06 |
| ETNK2 | ethanolamine kinase 2 | -1.605728613 | 1.79E-06 |
| GCK | glucokinase (hexokinase 4) | -1.715377637 | 1.94E-06 |
| FSD1 | fibronectin type III and SPRY domain containing 1 | -1.076308847 | 2.05E-06 |
| APOLD1 | apolipoprotein L domain containing 1 | -1.215019656 | 2.23E-06 |
| ZIC4 | Zic family member 4 | -2.211952225 | 2.29E-06 |
| LOC728377 | Rho guanine nucleotide exchange factor (GEF) 5 pseudogene | -1.615520587 | 2.32E-06 |
| SLC26A1 | solute carrier family 26 (sulfate transporter), member 1 | -1.001606918 | 2.41E-06 |
| MAP6 | microtubule-associated protein 6 | -1.172367755 | 2.58E-06 |
| CHST1 | carbohydrate (keratan sulfate Gal-6) sulfotransferase 1 | -1.836138462 | 2.85E-06 |
| HOXA7 | homeobox A7 | -1.420777384 | 3.02E-06 |
| LOC100288911 | uncharacterized LOC100288911 | -1.323070636 | 3.11E-06 |
| ARHGAP44 | Rho GTPase activating protein 44 | -1.500283203 | 3.68E-06 |
| LOC728730 | uncharacterized LOC728730 | -1.076561315 | 3.73E-06 |
| ANKRD5 | ankyrin repeat domain 5 | -1.090204977 | 3.78E-06 |
| RARRES1 | retinoic acid receptor responder (tazarotene induced) 1 | -1.777976618 | 3.97E-06 |
| ADHFE1 | alcohol dehydrogenase, iron containing, 1 | -1.203401554 | 4.37E-06 |
| TRIM7 | tripartite motif containing 7 | -1.218431832 | 4.38E-06 |
| ARHGEF5 | Rho guanine nucleotide exchange factor (GEF) 5 | -1.235109458 | 5.44E-06 |
| PAR5 | Prader-Willi/Angelman syndrome-5 | -1.22658986 | 5.55E-06 |
| NLGN1 | neuroligin 1 | -1.624766125 | 5.59E-06 |
| SYDE2 | synapse defective 1, Rho GTPase, homolog 2 (C. elegans) | -1.124267697 | 5.61E-06 |
| MGC16121 | uncharacterized protein MGC16121 | -1.462282543 | 7.60E-06 |
| MCM3AP-AS1 | MCM3AP antisense RNA 1 (non-protein coding) | -1.440961921 | 9.08E-06 |
| VSTM2L | V-set and transmembrane domain containing 2 like | -1.280078363 | 9.78E-06 |
| COL11A2 | collagen, type XI, alpha 2 | -1.285254288 | 1.06E-05 |
| SLC4A4 | solute carrier family 4, sodium bicarbonate cotransporter, member 4 | -1.322823479 | 1.10E-05 |
| ZNF804A | zinc finger protein 804A | -1.683284882 | 1.11E-05 |
| ZIC1 | Zic family member 1 | -1.672566484 | 1.25E-05 |
| DPY19L2P2 | dpy-19-like 2 pseudogene 2 (C. elegans) | -1.462758448 | 1.42E-05 |
| ZNF573 | zinc finger protein 573 | -1.062821792 | 1.48E-05 |
| GDPD3 | glycerophosphodiester phosphodiesterase domain containing 3 | -1.514582785 | 1.68E-05 |
| HSD17B8 | hydroxysteroid (17-beta) dehydrogenase 8 | -1.060423786 | 1.85E-05 |
| MASP2 | mannan-binding lectin serine peptidase 2 | -1.247744429 | 1.85E-05 |
| OVGP1 | oviductal glycoprotein 1, 120kDa | -1.234009627 | 2.02E-05 |
| FP588 | uncharacterized LOC92973 | -1.01750735 | 2.28E-05 |
| C17orf97 | chromosome 17 open reading frame 97 | -1.282333254 | 2.42E-05 |
| MURC | muscle-related coiled-coil protein | -1.245354805 | 2.43E-05 |
| PBX4 | pre-B-cell leukemia homeobox 4 | -1.503241108 | 2.82E-05 |
| CIB2 | calcium and integrin binding family member 2 | -1.191083538 | 2.88E-05 |
| LOC100287314 | uncharacterized LOC100287314 | -1.436055134 | 3.17E-05 |
| RNF207 | ring finger protein 207 | -1.190213206 | 3.18E-05 |
| LOC100133445 | uncharacterized LOC100133445 | -1.274396843 | 3.41E-05 |
| C11orf45 | chromosome 11 open reading frame 45 | -1.144178599 | 3.75E-05 |
| PIP5KL1 | phosphatidylinositol-4-phosphate 5-kinase-like 1 | -1.266037772 | 4.70E-05 |
| LOC284440 | uncharacterized LOC284440 | -1.068536745 | 5.63E-05 |
| RGS20 | regulator of G-protein signaling 20 | -1.187265027 | 7.19E-05 |
| TRPV4 | transient receptor potential cation channel, subfamily V, member 4 | -1.142806376 | 7.34E-05 |
| LOC100505702 | uncharacterized LOC100505702 | -1.231378643 | 7.48E-05 |
| TCTEX1D1 | Tctex1 domain containing 1 | -1.581171486 | 8.37E-05 |
| FAM86HP | family with sequence similarity 86, member A pseudogene | -1.490264618 | 9.22E-05 |
| PKDREJ | polycystic kidney disease (polycystin) and REJ homolog (sperm receptor for egg jelly homolog, sea urchin) | -1.356050257 | 0.000105193 |
| RGS17 | regulator of G-protein signaling 17 | -1.075985895 | 0.000107198 |
| ULBP3 | UL16 binding protein 3 | -1.044153974 | 0.000114215 |
| HOXA1 | homeobox A1 | -1.188425305 | 0.000164546 |
| RAPGEF5 | Rap guanine nucleotide exchange factor (GEF) 5 | -1.320125002 | 0.000189253 |
| ARMC4 | armadillo repeat containing 4 | -1.432646057 | 0.000198876 |
| CDNF | cerebral dopamine neurotrophic factor | -1.26281063 | 0.000246607 |
| KIRREL3 | kin of IRRE like 3 (Drosophila) | -1.118650753 | 0.000256845 |
| SNX32 | sorting nexin 32 | -1.200508799 | 0.000269536 |
| MST1P2 | macrophage stimulating 1 (hepatocyte growth factor-like) pseudogene 2 | -1.087378639 | 0.000342084 |
| KCNAB1 | potassium voltage-gated channel, shaker-related subfamily, beta member 1 | -1.041548728 | 0.000597042 |
| CERKL | ceramide kinase-like | -1.005631002 | 0.000670637 |
| LOC100289495 | uncharacterized LOC100289495 | -1.063622557 | 0.000710623 |
| GCNT4 | glucosaminyl (N-acetyl) transferase 4, core 2 | -1.116734398 | 0.000791479 |
| IL2RB | interleukin 2 receptor, beta | -1.190225049 | 0.001042901 |
| SLC37A1 | solute carrier family 37 (glycerol-3-phosphate transporter), member 1 | -1.190420266 | 0.001049285 |
| SLC9A3 | solute carrier family 9 (sodium/hydrogen exchanger), member 3 | -1.142713641 | 0.001178883 |
| GGT1 | gamma-glutamyltransferase 1 | -1.123665874 | 0.001342382 |
| NPW | neuropeptide W | -1.154553354 | 0.001517618 |
| SLC29A2 | solute carrier family 29 (nucleoside transporters), member 2 | -1.035339978 | 0.001592753 |
| LOC100506472 | uncharacterized LOC100506472 | -1.185753673 | 0.001995631 |
| CA13 | carbonic anhydrase XIII | -1.058728611 | 0.00214848 |
| FANK1 | fibronectin type III and ankyrin repeat domains 1 | -1.039004366 | 0.002478494 |
| ADAP1 | ArfGAP with dual PH domains 1 | -1.120131246 | 0.00323797 |
| LOC100272216 | uncharacterized LOC100272216 | -1.029094466 | 0.003778792 |
| PGR | progesterone receptor | -1.015980775 | 0.003784059 |
| FAT3 | FAT tumor suppressor homolog 3 (Drosophila) | -1.289334516 | 0.006528407 |
| NLRC3 | NLR family, CARD domain containing 3 | -1.002351409 | 0.006597389 |

1. GO_BiologicalProcess

| Category | Term | Genes | Fold Enrichment | FDR |
| --- | --- | --- | --- | --- |
| GOTERM_BP_DIRECT | GO:0030198~extracellular matrix organization | SPOCK2, TNC, ADAMTSL4, ELN, ITGA11, PRDX4, ITGB5, POSTN, DCN, VIT, ABI3BP, VCAM1, COL9A2, COL9A3, FOXF1, COMP, SERPINE1, TGFBI, LOX, FBN2, VWA1, THBS1, COL8A2, COL10A1, COL4A4, ICAM1, F11R, MATN3, COL4A3, RXFP1, EGFL6, ICAM5, OLFML2B, CCDC80, OLFML2A, ITGA2, ITGA4, ECM2, CSGALNACT1, COL14A1, LAMA3, BGN, ITGA6, LAMA5, LAMC3, KAZALD1, LAMC1, JAM2 | 3.303073519 | 6.41E-10 |
| GOTERM_BP_DIRECT | GO:0060337~type I interferon signaling pathway | EGR1, IFITM1, BST2, OAS3, HLA-A, RSAD2, OAS1, HLA-C, OAS2, HLA-B, HLA-F, IFIT2, OASL, IFIT1, IFI27, ISG15, IRF7, IRF1, IRF4, XAF1, MX1, MX2, IFI6 | 4.847088353 | 8.38E-07 |
| GOTERM_BP_DIRECT | GO:0009612~response to mechanical stimulus | CCL2, TNC, PPARG, CHI3L1, ASNS, BDKRB1, POSTN, FOSB, DCN, JUNB, INHBB, MEIS2, P2RY1, LRP11, TRPV4, PTCH1, IGFBP2, THBS1, ANGPT2, KALRN | 4.572050916 | 4.40E-05 |
| GOTERM_BP_DIRECT | GO:0001525~angiogenesis | SAT1, CCL2, NRP1, PTGS2, PGF, TNFRSF12A, LEPR, ENPEP, RORA, MMP2, PRKX, WARS, TYMP, HOXA3, APOD, HAND1, UNC5B, HAND2, XBP1, SERPINE1, TGFBI, HOXA7, ROBO4, PLCD3, ZC3H12A, ANGPT1, TMEM100, RAPGEF3, ANGPT2, COL8A2, PRKCA, FLT1, VAV3, APOLD1, COL15A1, PRKD1, THSD7A, EREG, LAMA5, PLXDC1, ADM2, TNFAIP2 | 2.540256092 | 9.34E-05 |
| GOTERM_BP_DIRECT | GO:0007155~cell adhesion | MTSS1, ATP1B1, FERMT1, L1CAM, BCAM, POSTN, EDIL3, PRKX, KIAA1462, TGFBI, COL12A1, ROBO2, SPON1, ADAM9, PRKCA, ICAM1, F11R, PTPRF, EGFL6, ICAM5, HES1, NCAM1, TNFAIP6, NCAM2, CD36, LAMC3, CD34, LSAMP, SUSD5, CNTN1, LAMC1, SEMA4D, ADAM12, CCL2, TNC, ITGA11, ITGB5, IL32, CDH2, ALCAM, VCAM1, COL6A6, COMP, CD4, CD24, THBS1, DPT, THBS4, COL4A3, ADAM23, PODXL, HCK, NLGN1, COL15A1, ITGA2, ITGA4, PCDH17, CDH13, OMD, LAMA3, NLGN4Y, ITGA6, CDON, CD58, ADAM22, NTM, MYH10 | 1.968770944 | 2.64E-04 |
| GOTERM_BP_DIRECT | GO:0006695~cholesterol biosynthetic process | MSMO1, MVD, CYP51A1, HMGCR, HMGCS1, FDPS, LSS, FDFT1, SQLE, DHCR7, INSIG1, MVK, IDI1, HSD17B7, DHCR24 | 5.324032974 | 4.68E-04 |
| GOTERM_BP_DIRECT | GO:0060333~interferon-gamma-mediated signaling pathway | ICAM1, HCK, OAS3, HLA-A, OAS1, HLA-C, HLA-B, OAS2, HLA-F, VCAM1, NCAM1, OASL, IRF7, MT2A, IRF1, HLA-DPA1, IRF4, CAMK2A, IFNGR2, HLA-DRA | 3.799309916 | 0.001159708 |
| GOTERM_BP_DIRECT | GO:0055114~oxidation-reduction process | PTGS2, UTY, KCNAB1, HMGCR, SNCA, PRDX4, HR, DUOX1, IL4I1, FDFT1, AKR1C3, MTHFD2, TDO2, MSRA, CH25H, GPX3, MICAL1, SRD5A1, LOXL4, LOXL3, GPX7, LOX, SARDH, GFOD1, DHCR24, KDM5D, SH3PXD2B, PCYOX1L, MOXD1, RDH5, PYCR1, DHRS3, MARC2, SQLE, H6PD, RRM2, HSD11B1, ASPHD1, HSD11B2, TXNRD1, PCYOX1, HSD17B10, ADHFE1, CYP51A1, EGLN3, ALDH1A1, VCAM1, ALDH1A2, DHCR7, ALDH1A3, CYP26B1, FASN, BDH1, HSD17B7, TYW1B, HSD17B8, NOX4, CHDH, MSMO1, DHRS13, NDUFA4L2, FADS1, SCD, MAOA, MAOB, FADS2, SOD2, FRRS1, VAT1L, CYP7B1, DIO2, AKR1B1, AOX1, PHGDH, HEPH, CP, CRYM | 1.754292847 | 0.002991269 |
| GOTERM_BP_DIRECT | GO:0001501~skeletal system development | ALPL, HOXA13, HOXA11, JAG2, SOX4, TP63, POSTN, ZBTB16, COL9A2, CHD7, COMP, COL12A1, COL11A2, ALX4, PITX1, COL10A1, SH3PXD2B, MATN3, CMKLR1, IGF1, NPR3, ANKH, RASSF2, GDF10, PHEX, IGFBP4, BMP6 | 2.658130332 | 0.014047194 |
| GOTERM_BP_DIRECT | GO:0006954~inflammatory response | CXCL1, RARRES2, CCL2, PTGS2, C3, LXN, CXCL3, CXCL2, CCL8, TLR3, BDKRB1, TLR4, GPR68, CXCL6, CCL5, IL34, ADORA1, CCL7, CXCL10, S1PR3, FOS, TNFRSF1B, CXCR4, SEMA7A, CYP26B1, SCN9A, ZC3H12A, FAS, THBS1, NOX4, F11R, PTGER2, IL6, GBP5, PTGER3, HCK, RELB, CHI3L1, NFAM1, TRIL, LGALS9, CHST1, PRKD1, TNFAIP6, GGT5, TNFRSF10C, RELT, AOX1, TNFAIP3, PLA2G4C, IGFBP4, CAMK1D, BMP6 | 1.886121796 | 0.021747609 |
| GOTERM_BP_DIRECT | GO:0045599~negative regulation of fat cell differentiation | WNT5A, IL6, ENPP1, TRIB3, FOXO1, RORA, TRIB2, MMP11, SOD2, GATA2, INSIG1, SORT1, ID4 | 4.174717919 | 0.066803125 |
| GOTERM_BP_DIRECT | GO:0009615~response to virus | IFIH1, IFITM1, BST2, OAS3, RSAD2, CCL8, OAS1, IFI44, OAS2, HMGA2, CCL5, DDX58, IFIT2, OASL, IFIT1, CXCR4, CHRM2, IRF7, MX1, MX2, IFNGR2, DCLK1 | 2.69751004 | 0.094005788 |
| GOTERM_BP_DIRECT | GO:0007162~negative regulation of cell adhesion | CDH13, CLDN7, PODXL, TNC, TGFBI, PLXNB3, ANGPT1, SEMA4D, ADAM22, JAM2, ADAMDEC1, ARHGDIB | 4.374340606 | 0.094006002 |
| GOTERM_BP_DIRECT | GO:0010628~positive regulation of gene expression | LDLR, STAR, TNC, VIM, MST1, IQGAP3, TLR3, GJA1, TLR4, HSPA1A, HSPA1B, RIMS2, ALDH1A2, HAND2, POU5F1, CD46, C1QTNF1, CYP26B1, ZC3H12A, TRPV4, DNMT3B, OLFM1, TWIST1, CDK1, IL6, ROCK2, RNF207, HMGA2, LGALS9, DDX58, INHBA, CD34, HOXD3, SFRP4, NTRK2, CNTN1, TFAP2A, ADM2, CTSH | 2.00768877 | 0.095685196 |
| GOTERM_BP_DIRECT | GO:0009952~anterior/posterior pattern specification | HOXA11, EMX2, ZBTB16, ACVR2A, ALDH1A2, ACVR2B, HOXC8, MSX1, HOXA3, HOXB7, HOXB8, HOXB5, CDON, HOXC4, HOXD3, HOXC5, HOXA7, ALX4 | 3.034698795 | 0.119320137 |
| GOTERM_BP_DIRECT | GO:0001666~response to hypoxia | ATP1B1, CCL2, PGF, ARNT2, TGFB3, EGLN3, POSTN, PDLIM1, MMP2, ADORA1, VCAM1, CXCR4, CD24, THBS1, ANGPT2, DNMT3B, EGR1, NOX4, NR4A2, APOLD1, ITGA2, CAPN2, DDIT4, SOD2, AGTRAP, AJUBA, PENK, UCP2, HSD11B2 | 2.274063697 | 0.121187686 |
| GOTERM_BP_DIRECT | GO:0050729~positive regulation of inflammatory response | WNT5A, LPL, CCL2, LDLR, IL1RL1, TLR3, ITGA2, CCL8, TLR4, IL33, CCL5, CCL7, IL17RB, MAPK13, SERPINE1, PLA2G2A, TRPV4 | 3.140936348 | 0.131285899 |
| GOTERM_BP_DIRECT | GO:0032496~response to lipopolysaccharide | ALPL, CXCL1, PTGS2, CXCL3, EDN1, CXCL2, SNCA, ACP5, BDKRB1, TLR4, DCN, CXCL6, CXCL10, GCH1, VCAM1, FOS, FAS, PTGER2, MAOB, ELANE, PCK2, JUNB, LGALS9, SOD2, TNFRSF10C, PENK, RELT, MGST2 | 2.302752473 | 0.133326396 |
| GOTERM_BP_DIRECT | GO:0045944~positive regulation of transcription from RNA polymerase II promoter | LMO2, LMO4, EDN1, ARNT2, FSTL3, TGFB3, TLR3, FOXO1, TLR4, RORA, ZIC1, IL11, CXCL10, PGR, GATA2, CDKN2B, FOXF1, SERPINE1, PITX1, TWIST1, PITX2, RELB, MLXIPL, SIX2, GRHL1, JUNB, AHR, HES1, MSX1, ARRB1, SIX1, TFAP2A, SLC40A1, WNT5A, TFEB, SOX4, NFKBIA, IL33, AHRR, MEIS2, HOXA7, P2RY1, CCPG1, MAFB, MET, NR4A2, NR4A3, SREBF2, NOTCH3, DDX58, DLX3, ITGA6, HOXB5, EBF2, EBF1, BMP6, NAMPT, E2F7, PPARG, TP63, RHOQ, FOS, POU5F1, ALX4, SERTAD1, EGR1, EGR2, ESR1, LDB2, FOSB, HMGA2, LPIN1, PRKD1, ACVR2A, MURC, INHBA, CSF3, GLIS3, GLIS1, DCN, RGMA, CHD7, HAND1, HAND2, XBP1, BCL11B, POU2F2, ETV1, ZC3H12A, NFATC2, ETV5, CEBPA, MAF, IL6, CEBPG, IGF1, CDH13, MEOX1, DBP, CDON, IRF7, ZBED3, IRF1, ZNF462, ID4, IRF4 | 1.457370358 | 0.135425065 |
| GOTERM_BP_DIRECT | GO:0032870~cellular response to hormone stimulus | SOCS2, PGF, ASNS, FOSB, SLIT2, JUNB, FOS, DUSP1, AVPR1A, ADRA2A, ROBO2, IGFBP2, SIK1 | 3.896403391 | 0.14113022 |
| GOTERM_BP_DIRECT | GO:0051216~cartilage development | WNT5A, SMAD9, EDN1, CHI3L1, ZBTB16, TIMP1, CSGALNACT1, HOXA3, HOXC4, HOXD3, SULF1, COL11A2, PITX1, BMP6, COL10A1 | 3.429038187 | 0.147740171 |
| GOTERM_BP_DIRECT | GO:0030334~regulation of cell migration | PLXNA4, LMO4, JAG2, LDB2, DPYSL3, NEXN, PRKX, AJUBA, DDX58, MMP10, LAMA3, SERPINE2, CXCR4, LAMA5, ROBO4, AMOT, PITX2 | 3.098491262 | 0.156119825 |
| GOTERM_BP_DIRECT | GO:0048661~positive regulation of smooth muscle cell proliferation | NAMPT, IL6, PTGS2, HMGCR, EDN1, ELANE, IGF1, ITGA2, NR4A3, CCL5, NOTCH3, CDH13, EREG, AKR1B1, THBS1 | 3.37188755 | 0.179694966 |
| GOTERM_BP_DIRECT | GO:0045766~positive regulation of angiogenesis | PRKCA, WNT5A, FLT1, PGF, C3, CHI3L1, AQP1, VASH2, ANXA3, PRKD1, GATA2, XBP1, CD34, F3, SERPINE1, ZC3H12A, ADM2, RAPGEF3, THBS1, CTSH, ANGPT2, TWIST1 | 2.580226995 | 0.184129791 |
| GOTERM_BP_DIRECT | GO:0006955~immune response | AQP9, IL16, TLR4, CXCL10, SEMA7A, SEMA3C, FAS, APLN, CMKLR1, PRG4, HLA-A, HLA-C, HLA-B, CTSS, PDCD1LG2, HLA-F, TNFRSF10C, CD36, TNFSF13B, RELT, HLA-DPA1, SEMA4D, HLA-DRA, CXCL1, CSF3, CCL2, ENPP1, C3, CXCL3, CXCL2, OAS3, CCL8, IL32, OAS1, OAS2, CXCL6, CCL5, CD74, TNFRSF1B, XBP1, CD4, THBS1, SECTM1, IL6, CRIP1, IL7, IL1RL1, CEBPG, IL1RN, IKBKE, TNFSF10, ADAMDEC1, IFI6, BMP6 | 1.729994563 | 0.190343957 |
| GOTERM_BP_DIRECT | GO:0060021~palate development | WNT5A, FRAS1, GABRB3, TIPARP, TGFB3, MSC, INHBA, ACVR2B, DHRS3, CHD7, MSX1, HAND2, INSIG1, TFAP2A, ALX4, COL11A2, TWIST1 | 3.016952019 | 0.218378506 |
| GOTERM_BP_DIRECT | GO:0007267~cell-cell signaling | NAMPT, NRP1, PGF, CTF1, NDP, EDN1, GDF5, FGF11, GJA1, CCL8, NMB, CXCL6, ENPEP, CCL5, ADORA1, CCL7, IL11, CXCL10, PGR, PCSK1, PCDH1, BDNF, ECE2, HCN2, TRHDE, EFNB3, BST2, IL7, FADS1, GJB2, TNFAIP6, INHBA, TNFSF10, EREG, GDF15, PHEX, ADRA1D | 1.96472188 | 0.240093674 |
| GOTERM_BP_DIRECT | GO:0042493~response to drug | PTGS2, STAR, PGF, ADCYAP1R1, SNCA, PPARG, HMGCS1, AQP1, MDK, ABCA3, FOS, SLC1A3, APOD, SEMA3C, PTN, SRD5A1, LOX, NFATC2, THBS1, DNMT3B, ICAM1, CDK1, LPL, SLC8A1, IL6, VAV3, TRPA1, MAOB, EMX2, ITGA2, FOSB, JUNB, ABCB4, SOD2, INHBA, SFRP1, FABP3, HSD11B2, PTCH1, IGFBP2, DUSP6, HTR2A | 1.863411541 | 0.258661422 |
| GOTERM_BP_DIRECT | GO:0019882~antigen processing and presentation | RAB3B, MICA, ULBP3, ULBP1, RELB, HLA-A, HLA-C, HLA-DPA1, HLA-B, CTSS, CTSH, CD74, RAB33A, HLA-DRA | 3.433194597 | 0.27537163 |
| GOTERM_BP_DIRECT | GO:0071549~cellular response to dexamethasone stimulus | EIF4EBP1, IL6, CCL2, STAR, FOXO1, SRD5A1, FBXO32, AQP1, DNMT3B, DDIT4 | 4.65087938 | 0.315817502 |
| GOTERM_BP_DIRECT | GO:0030335~positive regulation of cell migration | EDN1, LRRC15, CCL5, CCL7, GTSE1, SEMA3G, XBP1, FOXF1, SEMA7A, ADRA2A, SEMA3C, FAM83H, THBS1, PRKCA, F10, FLT1, PODXL, SUN2, IGF1, MYADM, TNFAIP6, CDH13, ITGA6, SEMA4G, F3, ROR2, SEMA4D, CTSH, ATP8A1 | 2.125755195 | 0.397040322 |
| GOTERM_BP_DIRECT | GO:0008217~regulation of blood pressure | TRHDE, PTGS2, PPARG, EDN1, NPR1, NPR3, SOD2, AGTRAP, GCH1, EDNRB, ACE, RENBP, CD34, GUCY1A3, ERAP2 | 3.112511585 | 0.445475365 |
| GOTERM_BP_DIRECT | GO:0043524~negative regulation of neuron apoptotic process | NES, NRP1, CCL2, GABRB3, STAR, GRIK2, CRLF1, GDF5, SNCA, TGFB3, NR4A2, BIRC5, NR4A3, MDK, SOD2, BDNF, UNC5B, APOE, VSTM2L, SIX1, NTRK2, ANGPT1, NRBP2 | 2.350103444 | 0.503267789 |
| GOTERM_BP_DIRECT | GO:0008299~isoprenoid biosynthetic process | MVD, HMGCR, FDPS, HMGCS1, MVK, IDI1, FDFT1 | 6.7437751 | 0.548015505 |
| GOTERM_BP_DIRECT | GO:0010811~positive regulation of cell-substrate adhesion | ITGA6, FBLN2, EGFL6, SPOCK2, FOXF1, CCDC80, PTN, EDIL3, ECM2, VIT, ABI3BP | 3.904290848 | 0.625619814 |
| GOTERM_BP_DIRECT | GO:0007399~nervous system development | CXCL1, CTF1, NDP, FGF11, GPM6B, L1CAM, NRN1, MDK, SLC7A5, ADORA1, FOS, EDNRB, PCDH1, BDNF, ZC3H12A, APBA2, RAPGEF5, PTN, ATOH8, CRIM1, OLFM1, DCLK1, EFNB3, STMN3, MAFB, NLGN1, DPYSL4, INSC, GAS7, HES1, CSGALNACT1, INHBA, CHRM2, LSAMP, ST8SIA4, GFRA1, SCN8A, GFRA2, KALRN | 1.832802989 | 0.651320472 |
| GOTERM_BP_DIRECT | GO:0043066~negative regulation of apoptotic process | CLDN7, IER3, SNCA, ARNT2, FOXO1, TP63, AQP1, PDCD4, ADORA1, CIAPIN1, IL31RA, EDNRB, CERKL, ANGPT1, FAS, TWIST1, DHCR24, CDK1, EGR3, SOCS2, NTSR1, HMGA2, FMN2, AMIGO2, MSX1, TFAP2A, SEMA4D, CTSH, SLC40A1, CAMK1D, WNT5A, LIMS2, NFKBIA, ASNS, CD74, TIMP1, XBP1, BCL11B, COMP, THBS1, IL6, IL2RB, LTK, IL7, HCK, IGF1, BIRC5, BIRC3, SOD2, SFRP1, DUSP1, PLK2, PLK1, UCP2, GRK5 | 1.630363211 | 0.73475794 |
| GOTERM_BP_DIRECT | GO:0030324~lung development | WNT5A, CEBPA, PDPN, FSTL3, CHI3L1, HES1, ALDH1A2, ACVR2B, EIF4EBP1, LAMA5, DHCR7, FOXF1, HSD11B1, PTN, HEG1, LOX | 2.839484253 | 0.741504753 |
| GOTERM_BP_DIRECT | GO:0022617~extracellular matrix disassembly | SH3PXD2B, ELANE, ELN, CTSS, DCN, CAPN2, MMP2, MMP1, TIMP1, MMP11, MMP10, LAMA3, CAPG, LAMC1, FBN2, LCP1 | 2.839484253 | 0.741504753 |
| GOTERM_BP_DIRECT | GO:0010595~positive regulation of endothelial cell migration | PRKCA, WNT5A, PRKD1, NRP1, ROCK2, EDN1, ZC3H12A, ANGPT1, SCARB1, ATOH8, THBS1, ANXA3 | 3.518491357 | 0.788642531 |
| GOTERM_BP_DIRECT | GO:0043065~positive regulation of apoptotic process | PNMA2, PTGS2, HIP1R, TNFRSF12A, ADAMTSL4, SAV1, SNCA, TGFB3, TLR3, FOXO1, SOX4, ZBTB16, MCF2L, ALDH1A2, TIAM2, ALDH1A3, PTN, FAS, FGD3, FGD4, ARHGEF4, NOX4, IL6, VAV3, NTSR1, HMGA2, SLIT2, IFIT2, TNFSF10, ITGA6, RASGRF2, SFRP1, DUSP1, SFRP4, MLLT11, RASSF2, CLIP3, DUSP6, KALRN, CAMK1D | 1.798340027 | 0.803918012 |
| GOTERM_BP_DIRECT | GO:0051607~defense response to virus | MICA, IFITM1, OAS3, RSAD2, TLR3, OAS1, IFI44L, OAS2, IL33, CXCL10, ISG15, ZC3H12A, MX1, MX2, POLR3G, PTPRC, IL6, BST2, HERC5, DDIT4, IFIT2, OASL, IFIT1, UNC13D, IRF1, GBP3 | 2.125310941 | 0.902170144 |
| GOTERM_BP_DIRECT | GO:0019221~cytokine-mediated signaling pathway | CEBPA, CSF3, ASPN, IL6, IL2RB, CCL2, PODN, RTN4RL1, SOCS2, HCK, DUOX1, LIFR, DCN, LRRC15, IL31RA, IL17RB, BGN, IL20RB, EREG, F3, IL15RA, IL13RA2 | 2.265084767 | 1.168595487 |
| GOTERM_BP_DIRECT | GO:0008284~positive regulation of cell proliferation | NAMPT, CLDN7, PGF, ARNT2, EDN1, IL11, IL31RA, CXCL10, AKR1C3, S1PR3, EDNRB, APLN, SERTAD1, NUDT16, CDC7, MLXIPL, LIFR, HES1, TNFSF13B, EREG, EIF5A2, CTSH, CSF3, MVD, CTF1, TNC, CRLF1, ST8SIA1, SOX4, NMB, IL34, TIMP1, ALDH1A2, HOXA3, RAC2, XBP1, ADRA2A, PTN, THBS1, IL6, SHMT2, FLT1, CCPG1, IL7, HCK, IGF1, BIRC5, CDC20, SFRP1, NTRK2, AVPR1A, ID4, GRK5, ADRA1D, HTR2A | 1.591878243 | 1.297564578 |
| GOTERM_BP_DIRECT | GO:0010759~positive regulation of macrophage chemotaxis | RARRES2, CCL2, CMKLR1, TRPV4, THBS1, CCL5 | 7.356845564 | 1.310167023 |
| GOTERM_BP_DIRECT | GO:0030574~collagen catabolic process | COL4A4, COL4A3, MMP27, COL15A1, CTSS, MMP2, MMP1, MMP11, MMP10, COL6A6, COL12A1, COL11A2, COL8A2, COL10A1 | 2.950401606 | 1.3306425 |
| GOTERM_BP_DIRECT | GO:0014070~response to organic cyclic compound | EDNRB, ICAM1, CDK1, NAMPT, MKI67, SFRP1, FADS1, TRPA1, ANGPT2, ACSL3, POLR2A, DUSP6 | 3.303073519 | 1.39915661 |
| GOTERM_BP_DIRECT | GO:0007517~muscle organ development | EGR3, CTF1, UTRN, ITGA11, IGF1, CXCL10, MURC, MSX1, BVES, XBP1, LAMA5, SGCD, ETV1, MKX, ALX4, ZFHX3, TWIST1 | 2.576273634 | 1.424123508 |
| GOTERM_BP_DIRECT | GO:0051384~response to glucocorticoid | ALPL, IL6, DUSP1, PTGS2, PAPPA, IL1RN, HSD11B2, FAS, IGFBP2, ABCA3, MDK, ANXA3, ADAM9, BMP6 | 2.905010812 | 1.550767015 |
| GOTERM_BP_DIRECT | GO:0060907~positive regulation of macrophage cytokine production | WNT5A, CD36, SEMA7A, TLR4, CD74 | 9.633964429 | 1.633546143 |
| GOTERM_BP_DIRECT | GO:0007229~integrin-mediated signaling pathway | ADAM11, VAV3, ADAM23, HCK, ITGA11, ITGB5, ITGA2, ITGA4, PRKD1, ITGA6, LAMA5, SEMA7A, ADAMTS10, ADAMTS1, ADAM22, ADAM12, ADAMDEC1, ADAM9 | 2.452281855 | 1.695275052 |
| GOTERM_BP_DIRECT | GO:0032355~response to estradiol | PTGS2, SOCS2, OPRL1, ADCYAP1R1, ARNT2, ESR1, POSTN, GJB2, ALDH1A2, IFI27, DUSP1, PENK, PTN, PTCH1, CD4, IGFBP2, DNMT3B | 2.519652235 | 1.824225917 |
| GOTERM_BP_DIRECT | GO:0009409~response to cold | LPL, FOS, IL6, HSPA2, LRP11, TRPA1, PPARG, PCSK1N, SOD2, CXCL10 | 3.746541722 | 1.840107364 |
| GOTERM_BP_DIRECT | GO:0006952~defense response | IL32, COLEC12, HLA-B, PSG1, CD74, IL17RB, IL31RA, INHBB, KCNN4, INHBA, TAP1, MX1, MX2, ICOSLG | 2.818294072 | 2.082113698 |
| GOTERM_BP_DIRECT | GO:0034605~cellular response to heat | MKI67, ST8SIA1, C8ORF4, TRPV4, HSPA1A, HSPA1B, THBS1, MYOF, SCARA5, CXCL10 | 3.645283838 | 2.26920047 |
| GOTERM_BP_DIRECT | GO:0045669~positive regulation of osteoblast differentiation | CEBPA, CTHRC1, IL6, IFITM1, GJA1, IGF1, TP63, PRKD1, ACVR2A, ACVR2B, ID4, FBN2, BMP6 | 2.922302544 | 2.419450915 |
| GOTERM_BP_DIRECT | GO:0033280~response to vitamin D | ALPL, STC2, PTGS2, STC1, CD4, AQP3, CXCL10 | 5.245158411 | 2.592998312 |
| GOTERM_BP_DIRECT | GO:0008584~male gonad development | WNT5A, STAR, HOXA11, ESR1, HMGCS1, FSTL3, PRDX4, TLR3, HMGA2, AKR1C3, INHBA, TNFSF10, COL9A3, SFRP1, MAMLD1, SRD5A1, PITX2 | 2.439237802 | 2.597875599 |
| GOTERM_BP_DIRECT | GO:0032869~cellular response to insulin stimulus | INHBB, CCL2, ENPP1, GCK, STAR, XBP1, UCP2, PPARG, DENND4C, TRIB3, FOXO1, PCSK9, RHOQ, SRD5A1, LPIN1 | 2.627444844 | 2.640927845 |
| GOTERM_BP_DIRECT | GO:0043547~positive regulation of GTPase activity | PREX2, ARHGAP17, RAB3IP, MCF2L, ARHGAP6, TIAM2, GRIN2D, STARD8, RAPGEF5, ANGPT1, RAPGEF3, SHC3, SHC2, ALS2CL, ARHGEF4, ICAM1, F11R, ARHGEF5, SIPA1L2, PSD4, NCAM1, RASGRF2, EREG, FNBP1L, ARRB1, RIN2, SEMA4D, KALRN, RALGPS2, CCL2, CCL8, CYTH3, CCL5, CCL7, ADAP1, ALDH1A1, DOCK2, AGAP11, PLEKHG1, ARHGAP44, CDC42EP3, CAMK2A, FGD3, FGD4, ARHGDIB, IL2RB, VAV3, ARHGEF37, RGS17, RGS16, DOCK5, RGS20, SFRP1, RGS2, ITGA6, RGS3, RGS4, DENND4C, RGS7, GFRA1, JAK3, GFRA2 | 1.480049757 | 3.567980179 |
| GOTERM_BP_DIRECT | GO:0045600~positive regulation of fat cell differentiation | SH3PXD2B, CEBPA, RARRES2, SFRP1, CMKLR1, XBP1, SAV1, PPARG, ZC3H12A, ZBTB16, HTR2A | 3.156660685 | 3.671788596 |
| GOTERM_BP_DIRECT | GO:0051056~regulation of small GTPase mediated signal transduction | ARHGEF4, RHOJ, VAV3, SIPA1L2, RHOQ, ARHGAP17, MCF2L, ARHGAP6, SYDE2, TIAM2, RAC2, RASGRF2, ARHGAP44, SIPA1L1, STARD8, RHOBTB1, AMOT, FGD3, ARHGDIB, FGD4, KALRN | 2.113720554 | 3.76276075 |
| GOTERM_BP_DIRECT | GO:0007204~positive regulation of cytosolic calcium ion concentration | MCHR1, PTGER2, PTGER3, OPRL1, EDN1, ESR1, GJA1, NMB, BDKRB1, S1PR3, GATA2, EDNRB, CD36, CXCR4, C1QTNF1, P2RY1, AVPR1A, TRPV4, CD24, CIB2, ADRA1D | 2.113720554 | 3.76276075 |
| GOTERM_BP_DIRECT | GO:0045071~negative regulation of viral genome replication | IFIT1, OASL, ISG15, BST2, IFITM1, OAS3, RSAD2, OAS1, CCL5, MX1 | 3.37188755 | 4.045468808 |
| GOTERM_BP_DIRECT | GO:0006366~transcription from RNA polymerase II promoter | LMO4, ARNT2, TP63, ZIC1, GATA2, FOS, POU5F1, FOXF1, ALX4, PITX1, PITX2, EGR1, EGR2, ESR1, SIX2, FOSB, GRHL1, HMGA2, MSC, JUNB, AHR, MSX1, ARRB1, SIX1, TFAP2A, NFE2L3, GLIS3, ETV7, GLIS1, SOX4, POLR2A, MEIS2, HAND1, HAND2, XBP1, BCL11B, POU2F2, HOXA7, ETV1, TCEA1, NFATC2, ETV5, MAF, CEBPA, MAFB, CEBPG, DLX3, DBP, MEOX1, IRF7, EBF2, HOXB5, EBF1, IRF1, IRF4, ZFHX3, PBX4 | 1.498616689 | 4.13317753 |
| GOTERM_BP_DIRECT | GO:0071526~semaphorin-plexin signaling pathway | NRP1, PLXNA4, SEMA4G, SEMA3G, SEMA7A, PLXNB3, MET, SEMA3C, SEMA4D | 3.678422782 | 4.266973629 |
| GOTERM_BP_DIRECT | GO:0035023~regulation of Rho protein signal transduction | ARHGEF4, VAV3, ARHGEF37, ARHGEF5, PREX2, MCF2L, MURC, PLEKHG1, TIAM2, RASGRF2, FGD3, FGD4, KALRN, ARHGDIB, ALS2CL | 2.497694482 | 4.313293423 |
| GOTERM_BP_DIRECT | GO:0071346~cellular response to interferon-gamma | WNT5A, CCL2, GBP5, STAR, CD58, EDN1, CCL8, TLR3, HLA-DPA1, CCL5, CCL7, LGALS9 | 2.839484253 | 5.050873499 |
| GOTERM_BP_DIRECT | GO:0008360~regulation of cell shape | FMNL1, CDC7, RHOJ, ICAM1, IL6, CCL2, SHROOM3, PDPN, HCK, PLXNB3, RHOQ, PALMD, CCL7, BVES, PALM2, PTN, SEMA4D, CDC42EP3, FGD3, MYH10, FGD4 | 2.02313253 | 6.262190057 |
| GOTERM_BP_DIRECT | GO:0030501~positive regulation of bone mineralization | ACVR2A, SLC8A1, ACVR2B, ISG15, TGFB3, TFAP2A, GPM6B, FBN2, BMP6 | 3.468227194 | 6.276088077 |
| GOTERM_BP_DIRECT | GO:0035116~embryonic hindlimb morphogenesis | CHD7, MSX1, AFF3, ZBTB16, ALX4, PITX1, PITX2, TWIST1 | 3.853585772 | 6.525614349 |
| GOTERM_BP_DIRECT | GO:0071407~cellular response to organic cyclic compound | ALPL, CEBPA, RGS20, SMAD9, CCL2, ITGA6, FOXF1, P2RY1, TIPARP, NFKBIA, CCL5, IGFBP5 | 2.743230549 | 6.637586005 |
| GOTERM_BP_DIRECT | GO:0008203~cholesterol metabolic process | CEBPA, CUBN, STAR, LDLR, LEPR, SREBF2, CYP7B1, APOL1, APOE, SQLE, CH25H, INSIG1, PCSK9 | 2.578502244 | 7.122457475 |
| GOTERM_BP_DIRECT | GO:0042102~positive regulation of T cell proliferation | HES1, VCAM1, PTPRC, IL6, CCDC88B, TNFSF13B, CD46, CD4, HLA-DPA1, JAK3, CCL5, PDCD1LG2 | 2.69751004 | 7.560487307 |
| GOTERM_BP_DIRECT | GO:0042472~inner ear morphogenesis | GATA2, CHD7, MAFB, ALDH1A3, SIX1, INSIG1, TFAP2A, ROR2, ZIC1, FZD6, PTPRQ | 2.853135619 | 7.848107366 |
| GOTERM_BP_DIRECT | GO:0071320~cellular response to cAMP | NOX4, HCN2, SLC8A1, AQP9, PENK, STAR, STC1, SRD5A1, RAPGEF3, AQP1, IGFBP5 | 2.853135619 | 7.848107366 |
| GOTERM_BP_DIRECT | GO:0008344~adult locomotory behavior | SEZ6L2, SNCG, HOXB8, GRIN2D, PREX2, SNCA, NR4A2, LGI4, ADAM22, NTSR1, TMOD1 | 2.853135619 | 7.848107366 |
| GOTERM_BP_DIRECT | GO:0090023~positive regulation of neutrophil chemotaxis | CXCL1, RAC2, CXCL3, CXCL2, CD74, CAMK1D, THBS4 | 4.291493246 | 7.861491989 |
| GOTERM_BP_DIRECT | GO:0046888~negative regulation of hormone secretion | IL6, EDN1, NMB, ADORA1, IL11 | 6.7437751 | 7.930515609 |
| GOTERM_BP_DIRECT | GO:0001938~positive regulation of endothelial cell proliferation | PRKCA, WNT5A, PRKD1, CDH13, EGR3, NRP1, CCL2, PGF, F3, PLXNB3, VASH2, THBS4, BMP6 | 2.541132647 | 8.017985125 |
| GOTERM_BP_DIRECT | GO:0071456~cellular response to hypoxia | ICAM1, SLC8A1, PTGS2, STC2, EDN1, MST1, RORA, AQP1, SLC29A1, FMN2, HYOU1, EIF4EBP1, SFRP1, PTN, STC1, TWIST1 | 2.247925033 | 8.214268392 |
| GOTERM_BP_DIRECT | GO:0007219~Notch signaling pathway | DTX4, CEBPA, CHAC1, JAG2, PGAM2, TP63, MDK, NOTCH3, HES1, S1PR3, KRT19, HOXD3, CNTN1, SUSD5, GRIP2, NEURL1B, TMEM100, PERP | 2.111094814 | 8.485225616 |
| GOTERM_BP_DIRECT | GO:0007416~synapse assembly | WNT5A, BDNF, PCDHB5, NLGN4Y, NRXN2, SPOCK2, SDK2, NLGN1, SDK1, PCDHB14, SHANK3, KIRREL3 | 2.653288564 | 8.576217565 |
| GOTERM_BP_DIRECT | GO:0010613~positive regulation of cardiac muscle hypertrophy | PRKCA, HAND2, EDN1, PDE5A, IGF1, NR4A3 | 5.057831325 | 8.737023481 |
| GOTERM_BP_DIRECT | GO:0070374~positive regulation of ERK1 and ERK2 cascade | PRKCA, NOX4, ABCA7, ICAM1, IL6, NRP1, CCL2, HMGCR, CHI3L1, CCL8, TLR4, CCL5, CD74, CCL7, LGALS9, CD36, HAND2, ARRB1, SEMA7A, P2RY1, PLA2G2A, TRPV4, ANGPT1, HTR2A | 1.84972117 | 9.308431763 |
| GOTERM_BP_DIRECT | GO:0016525~negative regulation of angiogenesis | COL4A3, CCL2, ROCK2, SULF1, AMOT, NPR1, PTN, DCN, THBS1, ANGPT2, THBS4, CXCL10 | 2.610493587 | 9.689235175 |
| GOTERM_BP_DIRECT | GO:0060395~SMAD protein signal transduction | INHBB, INHBA, FOS, SMAD9, VIM, GDF5, TGFB3, ROR2, GDF10, ATOH8, GDF15, BMP6 | 2.610493587 | 9.689235175 |
| GOTERM_BP_DIRECT | GO:0030217~T cell differentiation | EGR1, PTPRC, CHD7, LEPR, JAG2, SOX4, CD4, LFNG | 3.596680054 | 9.696778624 |
| GOTERM_BP_DIRECT | GO:0035924~cellular response to vascular endothelial growth factor stimulus | PRKD1, VCAM1, EGR3, NRP1, FLT1, XBP1, GAS1 | 4.104906583 | 9.87754323 |
| GOTERM_BP_DIRECT | GO:0007565~female pregnancy | NAMPT, STS, OVGP1, PGF, TGFB3, ITGA2, PSG1, FOSB, LGALS9, FOS, PAPPA, UCP2, HSD11B2, IGFBP2, IGFBP5 | 2.273182618 | 10.11073104 |
| GOTERM_BP_DIRECT | GO:0001558~regulation of cell growth | TMEM97, SOCS2, IGFBP6, NANOS1, IL17RB, EPB41L1, KAZALD1, H2AFY2, PAPPA2, HTRA3, IGFBP2, CRIM1, IGFBP4, IGFBP5 | 2.360321285 | 10.17761073 |
| GOTERM_BP_DIRECT | GO:0070509~calcium ion import | TRPC4, SLC8A1, GCK, CACNA1G, CACNB2, TRPV4 | 4.760311836 | 11.46936951 |
| GOTERM_BP_DIRECT | GO:0042475~odontogenesis of dentin-containing tooth | DLX3, ACVR2B, MSX1, HAND1, LAMA5, HAND2, BCL11B, TNC, JAG2, TP63, PITX2 | 2.69751004 | 11.63656093 |
| GOTERM_BP_DIRECT | GO:0010468~regulation of gene expression | ATP1B1, CRIP1, UTY, IL7, IGF1, AHR, MURC, POU5F1, APOE, DDX3Y, PHGDH, ZC3H12A, APBA2, SORT1, DNMT3B, SCARA5 | 2.158008032 | 11.84803189 |
| GOTERM_BP_DIRECT | GO:0071636~positive regulation of transforming growth factor beta production | PTGS2, CD34, CD46, LGALS9 | 8.991700134 | 12.12248543 |
| GOTERM_BP_DIRECT | GO:0070508~cholesterol import | CD36, STARD4, LDLR, SCARB1 | 8.991700134 | 12.12248543 |
| GOTERM_BP_DIRECT | GO:0031100~organ regeneration | CDK1, CCL2, MKI67, PGF, PPARG, NR4A3, LPIN1, ANGPT2, LCP1, ANXA3 | 2.869691532 | 12.14070521 |
| GOTERM_BP_DIRECT | GO:0034599~cellular response to oxidative stress | PRKD1, PYCR1, PENK, XBP1, SNCA, NR4A2, FOXO1, ZC3H12A, HSPA1A, HSPA1B, ETV5, GJB2 | 2.528915663 | 12.22275984 |
| GOTERM_BP_DIRECT | GO:0048704~embryonic skeletal system morphogenesis | HOXA3, HOXB7, HOXB8, HOXB5, HOXD3, SIX1, HOXA7, ALX4, FLVCR1 | 3.112511585 | 12.23216985 |
| GOTERM_BP_DIRECT | GO:0071356~cellular response to tumor necrosis factor | CEBPA, ICAM1, IL6, CCL2, EDN1, CHI3L1, CCL8, POSTN, RORA, PCK2, CCL5, CCL7, VCAM1, SFRP1, CD58, ZC3H12A, THBS1 | 2.084439576 | 12.38797276 |
| GOTERM_BP_DIRECT | GO:0050731~positive regulation of peptidyl-tyrosine phosphorylation | CSF3, ICAM1, IL6, NRP1, IGF1, CD74, IL11, CD36, CNTN1, CD4, ANGPT1, SEMA4D, HTR2A, THBS4 | 2.302752473 | 12.42148923 |
| GOTERM_BP_DIRECT | GO:0045444~fat cell differentiation | INHBB, CEBPA, EGR2, LRRC8C, NR4A2, GDF10, FOXO1, ID4, CLIP3, GRK5, NR4A3, HMGA2, IL11 | 2.401892502 | 12.45285237 |
| GOTERM_BP_DIRECT | GO:0016337~single organismal cell-cell adhesion | VCAM1, ICAM1, COL14A1, LIMS2, ITGA6, CD34, PDPN, ICAM5, FOXF1, CD58, DSP, CSTA, CD24, JAM2, COL8A2, CDH24 | 2.136641616 | 12.91549407 |
| GOTERM_BP_DIRECT | GO:0009887~organ morphogenesis | ABLIM1, ETV7, NRP1, CCL2, IL7, ELN, ITGA2, DCN, GATA2, EREG, COMP, PTCH1, BHLHE41, LFNG, TMEM176B | 2.199057098 | 13.35584575 |
| GOTERM_BP_DIRECT | GO:0009653~anatomical structure morphogenesis | IER3, HOXA11, MLXIPL, SIX2, IGF2BP3, GPC4, CSGALNACT1, HOXA1, S1PR3, DKK3, EREG, POU5F1, HOXB5, CLTCL1, PITX1 | 2.199057098 | 13.35584575 |
| GOTERM_BP_DIRECT | GO:0043087~regulation of GTPase activity | AJUBA, VAV3, BVES, SYDE2, STMN3, SIPA1L1, ARHGEF5, NTRK2, IQGAP3, FGD3, ADAP1, FGD4 | 2.490009268 | 13.64969035 |
| GOTERM_BP_DIRECT | GO:0001889~liver development | CEBPA, HES1, ALDH1A2, CADM1, DBP, XBP1, CEBPG, HMGCS1, PCSK9, PTN, SRD5A1, ASNS, SOD2 | 2.369434495 | 13.7898121 |
| GOTERM_BP_DIRECT | GO:0006909~phagocytosis | CDC7, ABCA7, GATA2, UNC13D, ICAM5, LEPR, ELANE, SYT7, ADORA1, ANXA3 | 2.809906292 | 13.85706768 |
| GOTERM_BP_DIRECT | GO:0030326~embryonic limb morphogenesis | FRAS1, DKK1, HOXA11, CYP26B1, GDF5, DYNC2H1, TP63, PTCH1, FBN2 | 3.034698795 | 14.1729691 |
| GOTERM_BP_DIRECT | GO:0006874~cellular calcium ion homeostasis | ATP1B1, CCL2, STC2, GRIK2, ELANE, CCL8, CCL5, CCL7, ATP2B4, APOE, ATP2A3, TRPV4, STC1, SV2A, HTR2A | 2.175411323 | 14.58504785 |
| GOTERM_BP_DIRECT | GO:0030500~regulation of bone mineralization | OMD, ENPP1, MGP, GJA1, ANKH, TWIST1 | 4.495850067 | 14.67485047 |
| GOTERM_BP_DIRECT | GO:0006935~chemotaxis | CXCL1, RARRES2, CCL2, CMKLR1, CXCL2, CCL8, L1CAM, CXCL6, CCL5, CCL7, LGALS9, CXCL10, LSP1, DOCK2, TYMP, RAC2, CXCR4, AMOT | 1.989966423 | 14.8573977 |
| GOTERM_BP_DIRECT | GO:0002040~sprouting angiogenesis | CDH13, NRP1, PGF, RSPO3, E2F7, ANGPT1, THBS1 | 3.776514056 | 14.86504952 |
| GOTERM_BP_DIRECT | GO:0051897~positive regulation of protein kinase B signaling | PTPRJ, CSF3, NOX4, IL6, F10, CHI3L1, AKR1C3, XBP1, F3, C1QTNF1, ANGPT1, THBS1, IGFBP5, HIP1 | 2.247925033 | 14.99548574 |
| GOTERM_BP_DIRECT | GO:0071222~cellular response to lipopolysaccharide | WNT5A, CSF3, ICAM1, IL6, CCL2, STAR, TLR4, CMPK2, CXCL10, EDNRB, TNFRSF1B, CD36, XBP1, SERPINE1, ZC3H12A, TNFAIP3, ADAM9 | 2.029100473 | 15.70803651 |
| GOTERM_BP_DIRECT | GO:2000505~regulation of energy homeostasis | CD36, LEPR, MLXIPL, FOXO1, NR4A3 | 5.619812584 | 15.90450382 |
| GOTERM_BP_DIRECT | GO:0043616~keratinocyte proliferation | CDH13, EREG, FERMT1, TP63, PTCH1 | 5.619812584 | 15.90450382 |
| GOTERM_BP_DIRECT | GO:0030195~negative regulation of blood coagulation | SERPINE2, CD34, APOE, EDN1, SERPINE1 | 5.619812584 | 15.90450382 |
| GOTERM_BP_DIRECT | GO:0048846~axon extension involved in axon guidance | ALCAM, FMOD, OGN, NRP1, SLIT2 | 5.619812584 | 15.90450382 |
| GOTERM_BP_DIRECT | GO:0055119~relaxation of cardiac muscle | GSTM2, ATP1B1, SLC8A1, RGS2, PDE5A | 5.619812584 | 15.90450382 |
| GOTERM_BP_DIRECT | GO:0030336~negative regulation of cell migration | PTPRJ, TMEFF2, PODN, IFITM1, BST2, PLXNB3, DPYSL3, FAM60A, SLIT2, SFRP1, SERPINE1, SULF1, PTN, STC1, IGFBP5 | 2.12961319 | 17.27232481 |
| GOTERM_BP_DIRECT | GO:0048843~negative regulation of axon extension involved in axon guidance | WNT5A, NRP1, SEMA4G, SEMA3G, SEMA7A, SEMA3C, SEMA4D | 3.631263516 | 17.84441953 |
| GOTERM_BP_DIRECT | GO:0001764~neuron migration | NRP1, TUBB2B, NR4A2, ASTN1, CELSR3, GJA1, MARK1, DDIT4, GATA2, MAPT, NTRK2, DCLK1, PITX2, TWIST1, KIRREL3, MYH10 | 2.055245745 | 17.86446574 |
| GOTERM_BP_DIRECT | GO:0001822~kidney development | TIPARP, FSTL3, SIX2, DCN, ALDH1A2, ACE, ACVR2B, SULF1, SIX1, PCSK9, TFAP2A, ADAMTS1, ROBO2, BMP6 | 2.195647707 | 17.91090114 |
| GOTERM_BP_DIRECT | GO:0045926~negative regulation of growth | MT1M, MT2A, MT1E, MT1X, MT1F, IGFBP5 | 4.259226379 | 18.34844612 |
| GOTERM_BP_DIRECT | GO:0001894~tissue homeostasis | CUBN, CD34, AKR1B1, NANOS1, ACACA, COL11A2 | 4.259226379 | 18.34844612 |
| GOTERM_BP_DIRECT | GO:0071294~cellular response to zinc ion | MT1M, MT2A, MT1E, HVCN1, MT1X, MT1F | 4.259226379 | 18.34844612 |
| GOTERM_BP_DIRECT | GO:0010886~positive regulation of cholesterol storage | LPL, CD36, SCARB1, SREBF2 | 7.707171543 | 19.28442536 |
| GOTERM_BP_DIRECT | GO:0071455~cellular response to hyperoxia | PPARG, FOXO1, FAS, DNMT3B | 7.707171543 | 19.28442536 |
| GOTERM_BP_DIRECT | GO:0043576~regulation of respiratory gaseous exchange | SLC5A3, NR4A2, NTSR1, APLN | 7.707171543 | 19.28442536 |
| GOTERM_BP_DIRECT | GO:0034340~response to type I interferon | IKBKE, SHMT2, ISG15, MX1 | 7.707171543 | 19.28442536 |
| GOTERM_BP_DIRECT | GO:0035810~positive regulation of urine volume | EDNRB, OPRL1, EDN1, NPR1, NPR3 | 5.187519308 | 21.03221288 |
| GOTERM_BP_DIRECT | GO:0006508~proteolysis | MASP1, MASP2, MMP27, MMP2, MMP1, MMP24, PAPPA, CD46, ERAP2, HTRA3, CFD, NUDT16, ADAM11, ELANE, CTSS, MMP11, ADAMTS6, MMP10, NAALAD2, ADAMTS8, BACE2, ADAMTS1, ADAM12, CTSH, ERMP1, ADAMTSL1, C3, ADAMTSL4, ADAMTS15, MST1, CPZ, PCSK1, ADAMTS10, FGL2, C2, RHBDF2, CPA4, PRSS53, TRHDE, ADAM23, CFB, PM20D2, KY, PAPLN, CAPN2, GGT5, SFRP1, THSD4, PAPPA2, ADAM22, PHEX, ADAMDEC1 | 1.402705221 | 21.03230688 |
| GOTERM_BP_DIRECT | GO:0014823~response to activity | CDK1, CCL2, STAR, EDN1, PTN, DNMT3B, ANGPT2, SOD2, BMP6 | 2.822975623 | 21.13115776 |
| GOTERM_BP_DIRECT | GO:0045785~positive regulation of cell adhesion | PRKCA, PTPRJ, VAV3, TMEM102, ITGA2, ANGPT1, CYTH3, CCL5, APBB1IP | 2.822975623 | 21.13115776 |
| GOTERM_BP_DIRECT | GO:0032728~positive regulation of interferon-beta production | DDX58, POLR3G, IFIH1, IRF7, IRF1, TLR3, TLR4 | 3.496772274 | 21.13923906 |
| GOTERM_BP_DIRECT | GO:0042476~odontogenesis | INHBA, TUFT1, TGFB3, AQP1, AQP3, PITX2, TWIST1 | 3.496772274 | 21.13923906 |
| GOTERM_BP_DIRECT | GO:0035987~endodermal cell differentiation | INHBA, LAMA3, COL12A1, ITGB5, ITGA4, HMGA2, MMP2 | 3.496772274 | 21.13923906 |
| GOTERM_BP_DIRECT | GO:0006633~fatty acid biosynthetic process | PRKAG3, LPL, MSMO1, XBP1, CH25H, PRKAG2, ACACA, FASN, ACSL3, HSD17B8 | 2.593759654 | 22.26519761 |
| GOTERM_BP_DIRECT | GO:0019233~sensory perception of pain | EDNRB, PTGS2, PENK, OPRL1, HOXB8, EDN1, TRPA1, P2RY1, SCN9A, BDKRB1 | 2.593759654 | 22.26519761 |
| GOTERM_BP_DIRECT | GO:0014911~positive regulation of smooth muscle cell migration | NOX4, NRP1, IGF1, ITGA2, POSTN, CCL5 | 4.04626506 | 22.46704167 |
| GOTERM_BP_DIRECT | GO:0007420~brain development | SYT1, NES, EGR2, STAR, SHROOM4, CADM1, KCNAB1, ARNT2, HMGCS1, MACROD2, AFF2, ZIC1, DDIT4, SLC17A7, ECE2, B3GNT5, APOD, H2AFY2, PHGDH, ROBO2, PTCH1, FAS, ACSL3, ZFHX3 | 1.703690552 | 22.6325504 |
| GOTERM_BP_DIRECT | GO:0008283~cell proliferation | CXCL1, CTF1, NDP, ELN, ENPEP, CD74, GPC4, KIF2C, SLC29A2, TGFBI, INSIG1, BUB1, IL15RA, RAPGEF3, BHLHE41, POLR3G, CDK1, COL4A3, CRIP1, LTK, MKI67, BST2, PDPN, PRG4, MET, SIX2, IGF1, ITGA2, DDIT4, CSGALNACT1, PRKD1, TNFSF13B, LAMA5, CD34, PLK1, SIX1, TXNRD1, ADRA1D, IGFBP4, MYH10 | 1.474049202 | 23.29623759 |
| GOTERM_BP_DIRECT | GO:0016339~calcium-dependent cell-cell adhesion via plasma membrane cell adhesion molecules | AJUBA, CDH13, PCDHB5, NLGN1, PCDHB14, CDH2, CDH23 | 3.37188755 | 24.73227696 |
| GOTERM_BP_DIRECT | GO:0007160~cell-matrix adhesion | LYPD3, EPDR1, ITGA11, ITGA2, ITGB5, BCAM, ITGA4, ECM2, VCAM1, ITGA6, CD34, FREM1, HOXD3, ADAM9 | 2.098063365 | 24.76955736 |
| GOTERM_BP_DIRECT | GO:0070434~positive regulation of nucleotide-binding oligomerization domain containing 2 signaling pathway | TLR4, HSPA1A, HSPA1B | 13.4875502 | 25.67486161 |
| GOTERM_BP_DIRECT | GO:0002486~antigen processing and presentation of endogenous peptide antigen via MHC class I via ER pathway, TAP-independent | HLA-A, HLA-C, HLA-B | 13.4875502 | 25.67486161 |
| GOTERM_BP_DIRECT | GO:0021773~striatal medium spiny neuron differentiation | INHBA, BCL11B, SHANK3 | 13.4875502 | 25.67486161 |
| GOTERM_BP_DIRECT | GO:0016477~cell migration | CTHRC1, CDK1, FLT1, PTPRF, USP9Y, PODXL, NANOS1, SIX2, BDKRB1, ENPEP, CDH2, MDK, HES1, LAMA5, JAK3, LAMC1, CD24, THBS1, NFATC2, USP24, LCP1, ADAM9 | 1.72515177 | 26.51283331 |
| GOTERM_BP_DIRECT | GO:0045721~negative regulation of gluconeogenesis | IL6, GCK, LEPR, MST1, SIK1 | 4.816982215 | 26.81303998 |
| GOTERM_BP_DIRECT | GO:0050965~detection of temperature stimulus involved in sensory perception of pain | LXN, NTSR1, ADORA1, HTR2A, MMP24 | 4.816982215 | 26.81303998 |
| GOTERM_BP_DIRECT | GO:0050892~intestinal absorption | F11R, CD36, ADRA2A, SCARB1, TJP2 | 4.816982215 | 26.81303998 |
| GOTERM_BP_DIRECT | GO:0030154~cell differentiation | ETV7, CADM1, PGF, ADCYAP1R1, JAG2, GPM6B, ZIC1, SLC7A5, MDK, IGSF10, PEG10, TYMP, DHCR7, ROBO4, ZC3H12A, SRD5A1, ANGPT1, ATOH8, BHLHE41, ASF1B, ETV5, HIP1, INA, SH3PXD2B, FLT1, RXFP1, EGFL6, HCK, APOLD1, COL15A1, MGP, INSC, MECOM, GAS7, FLNB, PTPRQ, THSD7A, INHBB, MURC, INHBA, RGS20, LAMA5, KAZALD1, SFRP4, TNK1, TNFAIP2, TMEM176B, DUSP6 | 1.401303917 | 27.13790127 |
| GOTERM_BP_DIRECT | GO:0010951~negative regulation of endopeptidase activity | COL4A3, BST2, SPOCK2, C3, LXN, CD109, PAPLN, SERPINI1, TIMP1, RENBP, SERPINE2, SERPINB7, SERPINE1, ITIH5, CSTA, PCSK1N, CRIM1 | 1.89494507 | 27.32047862 |
| GOTERM_BP_DIRECT | GO:0033993~response to lipid | GATA2, CD36, PPARG, NTSR1 | 6.7437751 | 27.74951826 |
| GOTERM_BP_DIRECT | GO:0071305~cellular response to vitamin D | SFRP1, PENK, TNC, PTN | 6.7437751 | 27.74951826 |
| GOTERM_BP_DIRECT | GO:0060993~kidney morphogenesis | GCNT4, SOX4, GCNT1, PRKX | 6.7437751 | 27.74951826 |
| GOTERM_BP_DIRECT | GO:0060100~positive regulation of phagocytosis, engulfment | GATA2, CD36, PPARG, ITGA2 | 6.7437751 | 27.74951826 |
| GOTERM_BP_DIRECT | GO:0048662~negative regulation of smooth muscle cell proliferation | OGN, APOD, PPARG, NPR1, NPR3, TNFAIP3, IGFBP5 | 3.255615566 | 28.59735001 |
| GOTERM_BP_DIRECT | GO:0090090~negative regulation of canonical Wnt signaling pathway | WNT5A, EGR1, CTHRC1, IGFBP6, FOXO1, CDH2, FZD6, PSMB9, DKK2, DKK3, RGS20, DKK1, SFRP1, PRICKLE1, APOE, PSME2, KIAA0922, SFRP4, ROR2, IGFBP2, IGFBP4 | 1.737659842 | 28.64372494 |
| GOTERM_BP_DIRECT | GO:0042127~regulation of cell proliferation | SAT1, PTGER2, TRNP1, SAV1, TNC, CXCL3, CXCL2, JAG2, EGLN3, TGFB3, NFKBIA, CXCL6, JUNB, CXCL10, TNFRSF10C, TNFRSF1B, CDCA7, RELT, LAMA5, DHCR7, PLCD3, TNK1, FAS | 1.676830566 | 29.8854005 |
| GOTERM_BP_DIRECT | GO:0001657~ureteric bud development | SPRY1, SMAD9, SFRP1, ARG2, SIX1, CRLF1, ROBO2, SLIT2 | 2.839484253 | 31.64568014 |
| GOTERM_BP_DIRECT | GO:0032720~negative regulation of tumor necrosis factor production | NLRC3, CD34, ACP5, ZC3H12A, TLR4, TNFAIP3, LGALS9, TWIST1 | 2.839484253 | 31.64568014 |
| GOTERM_BP_DIRECT | GO:0042157~lipoprotein metabolic process | LPL, APOL1, CUBN, APOE, APOLD1, PCSK9, SCARB1, APOL4 | 2.839484253 | 31.64568014 |
| GOTERM_BP_DIRECT | GO:0001942~hair follicle development | INHBA, DKK1, LAMA5, SAV1, CD109, LDB2, ALX4, FZD6 | 2.839484253 | 31.64568014 |
| GOTERM_BP_DIRECT | GO:0071345~cellular response to cytokine stimulus | CSF3, CXCR4, FOXF1, NFKBIA, DPYSL3, ITGA4 | 3.678422782 | 31.85628503 |
| GOTERM_BP_DIRECT | GO:0035019~somatic stem cell population maintenance | HES1, GATA2, SFRP1, POU5F1, ZHX2, SOX4, LDB2, HMGA2, LIN28A, DPPA4, POLR2A | 2.282508496 | 32.40633866 |
| GOTERM_BP_DIRECT | GO:0021510~spinal cord development | PLXDC1, PHGDH, SOX4, PTN, ROBO2, SRD5A1, ZIC1 | 3.147095047 | 32.69969634 |
| GOTERM_BP_DIRECT | GO:0044344~cellular response to fibroblast growth factor stimulus | EGR3, CCL2, SFRP1, STAR, SNCA, POSTN, CCL5 | 3.147095047 | 32.69969634 |
| GOTERM_BP_DIRECT | GO:0002474~antigen processing and presentation of peptide antigen via MHC class I | ACE, TAP1, HLA-A, HLA-C, ERAP2, HLA-B, HLA-F | 3.147095047 | 32.69969634 |
| GOTERM_BP_DIRECT | GO:0007193~adenylate cyclase-inhibiting G-protein coupled receptor signaling pathway | MCHR1, S1PR3, ADCY4, OPRL1, GNAI1, P2RY1, NPR3, ADORA1, HTR2A | 2.582722379 | 32.86646728 |
| GOTERM_BP_DIRECT | GO:0009267~cellular response to starvation | AKR1C3, INHBB, SFRP1, FADS1, FOXO1, PCSK9, SRD5A1, RRAGD, SREBF2 | 2.582722379 | 32.86646728 |
| GOTERM_BP_DIRECT | GO:0033627~cell adhesion mediated by integrin | ICAM1, ITGA6, ITGA11, ITGA2, ADAM9 | 4.495850067 | 33.11224699 |
| GOTERM_BP_DIRECT | GO:0008585~female gonad development | COL9A3, SFRP1, TIPARP, IDH1, PITX2 | 4.495850067 | 33.11224699 |
| GOTERM_BP_DIRECT | GO:0002244~hematopoietic progenitor cell differentiation | STON2, PTPRC, INHBA, BVES, SFRP1, PTPRZ1, HERC6, TMEM91, FSTL3, ANLN, PTPRQ | 2.247925033 | 35.10959743 |
| GOTERM_BP_DIRECT | GO:0032570~response to progesterone | FOS, PTGER2, CCL2, TGFB3, PTN, FOSB, THBS1, GJB2 | 2.766676964 | 35.31211411 |
| GOTERM_BP_DIRECT | GO:0030890~positive regulation of B cell proliferation | PTPRC, VAV3, TNFSF13B, BST1, IL7, TLR4, NFATC2, CD74 | 2.766676964 | 35.31211411 |
| GOTERM_BP_DIRECT | GO:0045668~negative regulation of osteoblast differentiation | SFRP1, HAND2, GDF10, PTCH1, RORB, SEMA4D, TWIST1, IGFBP5 | 2.766676964 | 35.31211411 |
| GOTERM_BP_DIRECT | GO:0007165~signal transduction | GABRB3, PGF, IQGAP3, FGF11, TLR3, RRAD, ADORA1, CXCL10, PGR, UNC5B, GRIN2D, STARD8, IL15RA, RAPGEF3, FAS, MX1, PDPN, TNFAIP6, ARRB1, CD34, PDE5A, RIPK3, ROR2, KALRN, CCL2, CCL8, MDK, CD74, CCL7, ALCAM, RAC2, ADRA2A, ANKDD1A, CDC42EP3, IL1RL1, MET, NR4A2, TNFSF10, PENK, ULK2, CLTCL1, NAMPT, NRP1, IGFBP6, PPARG, GJA1, BCAM, ARHGAP17, SKAP2, KCNIP3, ARHGAP6, PKN3, EVI2A, TMEM102, MICAL1, SHC3, APLN, ANGPT2, GPR173, NRXN2, ESR1, CLIC2, NFAM1, HMGA2, ARRDC3, FLNB, PRKD1, HUNK, ACVR2B, TNFRSF10C, TNFSF13B, RIN2, TXNRD1, RASD1, ICOSLG, CXCL1, SPOCK2, C3, SAV1, NDP, NR3C2, NMB, CXCL6, APBB1IP, GPRC5A, ARHGAP44, BCL11B, PDE1A, CD4, SECTM1, IL2RB, CAP2, LTK, TRHDE, COL15A1, IGF1, SH3BP5, RASSF3, LSP1, SYDE2, PTCH2, IGFBP2, GDF15, IGFBP4, IGFBP5 | 1.219804282 | 35.95989767 |
| GOTERM_BP_DIRECT | GO:0035456~response to interferon-beta | IKBKE, BST2, IFITM1, XAF1 | 5.994466756 | 37.04424236 |
| GOTERM_BP_DIRECT | GO:0001778~plasma membrane repair | DYSF, SYT7, MYOF, MYH10 | 5.994466756 | 37.04424236 |
| GOTERM_BP_DIRECT | GO:0002480~antigen processing and presentation of exogenous peptide antigen via MHC class I, TAP-independent | HLA-A, HLA-C, HLA-B, HLA-F | 5.994466756 | 37.04424236 |
| GOTERM_BP_DIRECT | GO:0043030~regulation of macrophage activation | SLC7A2, SNCA, RORA, CD74 | 5.994466756 | 37.04424236 |
| GOTERM_BP_DIRECT | GO:0007169~transmembrane receptor protein tyrosine kinase signaling pathway | MTSS1, FLT1, LTK, HCK, MET, IL31RA, NTRK2, ROR2, ANGPTL1, TNK1, CD4, SHC3, SHC2, GFRA2 | 1.966934404 | 37.37139338 |
| GOTERM_BP_DIRECT | GO:0007156~homophilic cell adhesion via plasma membrane adhesion molecules | PCDHB5, CADM1, PCDH20, PLXNB3, SDK2, SDK1, CELSR3, PCDHGB6, PCDHB14, PCDH7, CDH2, PCDH17, AMIGO2, CDH13, PCDH1, FAT3, ROBO2, CDH24, CDH23, KIRREL3 | 1.707284836 | 37.59984238 |
| GOTERM_BP_DIRECT | GO:0032868~response to insulin | EGR1, IL6, TNFSF10, EGR2, FADS1, FABP3, HSD11B2, SORT1, TRPV4, IGFBP2, GCNT1 | 2.214373914 | 37.89485015 |
| GOTERM_BP_DIRECT | GO:0046426~negative regulation of JAK-STAT cascade | ASPN, BGN, PODN, SOCS2, RTN4RL1, DCN, HMGA2, LRRC15 | 2.69751004 | 39.11148823 |
| GOTERM_BP_DIRECT | GO:0007158~neuron cell-cell adhesion | NCAM2, NLGN4Y, NRXN2, NLGN1, ASTN1 | 4.214859438 | 39.76287049 |
| GOTERM_BP_DIRECT | GO:0048168~regulation of neuronal synaptic plasticity | EGR2, STAR, APOE, CAMK2A, KALRN | 4.214859438 | 39.76287049 |
| GOTERM_BP_DIRECT | GO:0001974~blood vessel remodeling | ACE, ACVR2B, CHD7, BGN, HOXA3, RSPO3, SEMA3C | 2.950401606 | 41.43957414 |
| GOTERM_BP_DIRECT | GO:0008285~negative regulation of cell proliferation | CXCL1, PODN, RARRES1, IFITM1, PTGS2, E2F7, IGFBP6, TGFB3, SOX4, ZBTB16, SKAP2, ADORA1, ALDH1A2, WARS, SPRY1, SERPINE2, CDKN2B, BCL11B, DPT, DHCR24, CEBPA, NOX4, PTPRJ, COL4A3, IL6, TP53I11, CLMN, PTPN14, SOD2, CDH13, INHBA, MSX1, ADAMTS8, SFRP1, EREG, SFRP4, IRF1, FABP3, TFAP2A, ROR2, ADAMTS1 | 1.396438278 | 41.99938975 |
| GOTERM_BP_DIRECT | GO:0007422~peripheral nervous system development | SLC5A3, EDNRB, EGR3, EGR2, SCN8A, SERPINI1 | 3.37188755 | 42.3179822 |
| GOTERM_BP_DIRECT | GO:0032526~response to retinoic acid | DKK1, DUSP1, PPARG, PTCH1, IGFBP2, CTSH, AQP3, BMP6 | 2.631717112 | 43.01036418 |
| GOTERM_BP_DIRECT | GO:0043408~regulation of MAPK cascade | INHBB, INHBA, GDF5, TGFB3, GDF10, CD24, GDF15, BMP6 | 2.631717112 | 43.01036418 |
| GOTERM_BP_DIRECT | GO:0072012~glomerulus vasculature development | HES1, ANGPT1, ANGPT2 | 10.11566265 | 43.35902211 |
| GOTERM_BP_DIRECT | GO:0055118~negative regulation of cardiac muscle contraction | PDE5A, ZC3H12A, ADORA1 | 10.11566265 | 43.35902211 |
| GOTERM_BP_DIRECT | GO:0003253~cardiac neural crest cell migration involved in outflow tract morphogenesis | HAND2, PITX2, TWIST1 | 10.11566265 | 43.35902211 |
| GOTERM_BP_DIRECT | GO:0006564~L-serine biosynthetic process | SHMT2, PHGDH, PSAT1 | 10.11566265 | 43.35902211 |
| GOTERM_BP_DIRECT | GO:0097113~AMPA glutamate receptor clustering | APOE, NLGN1, SHANK3 | 10.11566265 | 43.35902211 |
| GOTERM_BP_DIRECT | GO:0021615~glossopharyngeal nerve morphogenesis | HOXA3, PLXNA4, HOXD3 | 10.11566265 | 43.35902211 |
| GOTERM_BP_DIRECT | GO:0015758~glucose transport | PRKAG3, SLC2A5, GCK, EDN1, SLC2A1, HK2, FABP5 | 2.860995497 | 45.97407124 |
| GOTERM_BP_DIRECT | GO:0071276~cellular response to cadmium ion | AKR1C3, STAR, MT1E, MT1X, MT1F | 3.96692653 | 46.57886094 |
| GOTERM_BP_DIRECT | GO:0033138~positive regulation of peptidyl-serine phosphorylation | WNT5A, PRKD1, CSF3, IL6, ATP2B4, ARRB1, NTRK2, SNCA, ANGPT1, RAPGEF3, IL11 | 2.119472174 | 46.60969312 |
| GOTERM_BP_DIRECT | GO:0040037~negative regulation of fibroblast growth factor receptor signaling pathway | WNT5A, SPRY1, SULF1, THBS1 | 5.39502008 | 46.63539713 |
| GOTERM_BP_DIRECT | GO:0016126~sterol biosynthetic process | MSMO1, SQLE, CH25H, PRKAG2 | 5.39502008 | 46.63539713 |
| GOTERM_BP_DIRECT | GO:2000406~positive regulation of T cell migration | TMEM102, ITGA4, CCL5, CXCL10 | 5.39502008 | 46.63539713 |
| GOTERM_BP_DIRECT | GO:0006884~cell volume homeostasis | SLC12A7, KCNN4, TRPV4, AQP1 | 5.39502008 | 46.63539713 |
| GOTERM_BP_DIRECT | GO:0045616~regulation of keratinocyte differentiation | ROCK2, CD109, GRHL1, AQP3 | 5.39502008 | 46.63539713 |
| GOTERM_BP_DIRECT | GO:0097191~extrinsic apoptotic signaling pathway | INHBA, TNFRSF1B, TNFRSF12A, KRT8, MLLT11, TLR3, IL33, FAS | 2.569057181 | 46.97251149 |
| GOTERM_BP_DIRECT | GO:0001503~ossification | IGSF10, IFITM1, CLEC3B, KAZALD1, RASSF2, FSTL3, MGP, SORT1, STC1, GPM6B, COL11A2, TWIST1 | 2.02313253 | 47.15164509 |
| GOTERM_BP_DIRECT | GO:0046718~viral entry into host cell | NCAM1, ICAM1, F11R, EFNB3, LDLR, CD46, ITGB5, ITGA2, SCARB1, HSPA1A, HSPA1B, HTR2A | 2.02313253 | 47.15164509 |
| GOTERM_BP_DIRECT | GO:0032092~positive regulation of protein binding | WNT5A, CSF3, CTHRC1, ACE, EPHB6, PLK2, HIP1R, ARRB1, TRIB3, LFNG | 2.211073803 | 48.5737171 |
| GOTERM_BP_DIRECT | GO:0007275~multicellular organism development | LMO2, FAM3C, LEPR, EDIL3, PRRX2, MCTP2, HOXC4, PIWIL2, FLVCR1, BST2, EGFL6, BST1, ZFY, CECR1, GRHL1, HMGA2, MMP11, HUNK, FMN2, TNFRSF10C, RELT, SORT1, ROR2, RPS4Y1, SSC5D, CSF3, WNT5A, TSHZ3, HOXA11, SPESP1, PAQR8, IGSF10, HOXA1, TNFRSF1B, HOXA3, FAT3, ASF1B, ARHGDIB, FZD6, DKK2, DKK3, SFRP1, HOXB7, DBP, HOXB8, MEOX1, EBF2, KAZALD1, EBF1, SFRP4, ADRA1D | 1.320278427 | 49.45545931 |
| GOTERM_BP_DIRECT | GO:0097190~apoptotic signaling pathway | PRKCA, IFI27, TNFRSF1B, RELT, MLLT11, RIPK3, TLR3, TLR4, FAS, ADORA1, HIP1 | 2.089620454 | 49.58487752 |
| GOTERM_BP_DIRECT | GO:0070098~chemokine-mediated signaling pathway | CXCL1, CCL2, CMKLR1, CXCR4, CXCL3, CXCL2, CCL8, CXCL6, CCL5, CCL7, CXCL10 | 2.089620454 | 49.58487752 |
| GOTERM_BP_DIRECT | GO:0071347~cellular response to interleukin-1 | ICAM1, IL6, CCL2, SFRP1, EDN1, CHI3L1, CCL8, ZC3H12A, RORA, CCL5, CCL7 | 2.089620454 | 49.58487752 |
| GOTERM_BP_DIRECT | GO:0050900~leukocyte migration | ICAM1, F11R, ATP1B1, PODXL, SLC3A2, L1CAM, ITGA4, SLC7A5, MMP1, CD74, ITGA6, CD34, CD58, ANGPT1, JAM2, ANGPT2 | 1.768859043 | 49.86372559 |
| GOTERM_BP_DIRECT | GO:0043410~positive regulation of MAPK cascade | TNFRSF1B, IL6, FLT1, RELT, CDON, C1QTNF1, NTRK2, IGF1, FAS, CDH2, IGFBP4, IL11 | 1.998155585 | 49.91873256 |
| GOTERM_BP_DIRECT | GO:0034097~response to cytokine | ALDH1A2, FOS, IFI27, RELB, LIFR, ACP5, TMEM102, JUNB, TIMP1 | 2.334383689 | 50.12193626 |
| GOTERM_BP_DIRECT | GO:0030155~regulation of cell adhesion | PTPRJ, ICAM1, LAMA3, LAMA5, ROCK2, JAG2, SERPINI1, PRKX | 2.509311665 | 50.95987669 |
| GOTERM_BP_DIRECT | GO:0048538~thymus development | HES1, HOXA3, MAFB, HAND2, LMO4, BCL11B, SIX1, RIPK3 | 2.509311665 | 50.95987669 |
| GOTERM_BP_DIRECT | GO:0048010~vascular endothelial growth factor receptor signaling pathway | PRKD1, VAV3, NRP1, CCL2, FLT1, ROCK2, XBP1, MAPK13, PGF, SULF1, SHC2 | 2.060597947 | 52.56861415 |
| GOTERM_BP_DIRECT | GO:0051930~regulation of sensory perception of pain | EDNRB, GRIN2D, EDN1, TMEM100, NTSR1, ADORA1 | 3.112511585 | 53.14543348 |
| GOTERM_BP_DIRECT | GO:0032757~positive regulation of interleukin-8 production | DDX58, SERPINE1, TLR3, TLR4, HSPA1A, HSPA1B | 3.112511585 | 53.14543348 |
| GOTERM_BP_DIRECT | GO:0001709~cell fate determination | GATA2, EBF2, CYP26B1, PTCH1, PTCH2 | 3.746541722 | 53.36976401 |
| GOTERM_BP_DIRECT | GO:0045089~positive regulation of innate immune response | POLR3G, GBP5, EREG, CCL5, MMP2 | 3.746541722 | 53.36976401 |
| GOTERM_BP_DIRECT | GO:0051412~response to corticosterone | FOS, STAR, MAOB, AVPR1A, FOSB | 3.746541722 | 53.36976401 |
| GOTERM_BP_DIRECT | GO:0033189~response to vitamin A | ALDH1A2, FADS1, PPARG, DNMT3B, PITX2 | 3.746541722 | 53.36976401 |
| GOTERM_BP_DIRECT | GO:0060384~innervation | CHD7, SERPINE2, GABRB3, SULF1, LRIG1 | 3.746541722 | 53.36976401 |
| GOTERM_BP_DIRECT | GO:0002576~platelet degranulation | RARRES2, FAM3C, F13A1, TGFB3, IGF1, PCDH7, TIMP3, TIMP1, CD36, CLEC3B, SERPINE1, THBS1, CFD, SRGN | 1.833259251 | 54.20180619 |
| GOTERM_BP_DIRECT | GO:0006865~amino acid transport | SLC38A5, SLC1A4, PDPN, SLC7A2, SLC3A2, SLC7A5, SLC43A2 | 2.69751004 | 55.08921101 |
| GOTERM_BP_DIRECT | GO:0001890~placenta development | DLX3, PEG10, E2F7, NDP, PPARG, ETNK2, DCN | 2.69751004 | 55.08921101 |
| GOTERM_BP_DIRECT | GO:0006629~lipid metabolic process | HSD17B10, LPL, LDLR, FADS1, CPNE7, IL1RN, PPARG, HMGCS1, FADS2, GDPD3, ACAT2, SREBF2, ABCB4, CD36, APOD, CH25H, PLCD3, APOL4, FABP5 | 1.632251298 | 55.88486741 |
| GOTERM_BP_DIRECT | GO:0061045~negative regulation of wound healing | HMGCR, SERPINE1, CD109, GJA1 | 4.904563709 | 56.00879234 |
| GOTERM_BP_DIRECT | GO:0048266~behavioral response to pain | SCN9A, THBS1, VWA1, THBS4 | 4.904563709 | 56.00879234 |
| GOTERM_BP_DIRECT | GO:0002064~epithelial cell development | SHROOM3, ADAMTSL4, ESR1, TP63 | 4.904563709 | 56.00879234 |
| GOTERM_BP_DIRECT | GO:0042981~regulation of apoptotic process | EGR1, GDF5, ESR1, TGFB3, TP63, BIRC5, GAS1, BIRC3, CARD6, RASSF3, INHBB, TNFRSF10C, TNFRSF1B, RELT, DUSP1, SDF2L1, ROBO4, GDF10, FAS, PERP, GDF15, ALX4, BMP6, HIP1 | 1.519723966 | 57.11389649 |
| GOTERM_BP_DIRECT | GO:0032436~positive regulation of proteasomal ubiquitin-dependent protein catabolic process | CEBPA, RNF144B, RNF166, PLK2, PRICKLE1, PLK1, TRIB3, HSPA1A, IL33, TRIB2 | 2.107429719 | 58.25168907 |
| GOTERM_BP_DIRECT | GO:0060349~bone morphogenesis | DHRS3, MSX1, CYP26B1, TFAP2A, ACP5, PAPPA2 | 2.997233378 | 58.45876658 |
| GOTERM_BP_DIRECT | GO:0014065~phosphatidylinositol 3-kinase signaling | LTK, XBP1, EDN1, PREX2, IGF1, HTR2A | 2.997233378 | 58.45876658 |
| GOTERM_BP_DIRECT | GO:0032755~positive regulation of interleukin-6 production | DDX58, WNT5A, IL6, CD36, MAPK13, TLR3, TLR4, IL33 | 2.397786702 | 58.85535667 |
| GOTERM_BP_DIRECT | GO:0008015~blood circulation | COL4A3, CHD7, HTR7, ELN, AVPR1A, GUCY1A3, MYOF, CXCL10 | 2.397786702 | 58.85535667 |
| GOTERM_BP_DIRECT | GO:0010629~negative regulation of gene expression | STC2, LDLR, TIPARP, ESR1, IQGAP3, GJA1, MYADM, LGALS9, PGR, ACE, SFRP1, HAND2, POU5F1, CD34, CD46, ZC3H12A, OLFM1 | 1.673637616 | 59.2603997 |
| GOTERM_BP_DIRECT | GO:0032354~response to follicle-stimulating hormone | PAPPA, SRD5A1, ASNS | 8.09253012 | 59.72450942 |
| GOTERM_BP_DIRECT | GO:0010828~positive regulation of glucose transport | C3, CLIP3, NR4A3 | 8.09253012 | 59.72450942 |
| GOTERM_BP_DIRECT | GO:1900227~positive regulation of NLRP3 inflammasome complex assembly | CD36, GBP5, TLR4 | 8.09253012 | 59.72450942 |
| GOTERM_BP_DIRECT | GO:0071504~cellular response to heparin | EGR1, SFRP1, SLIT2 | 8.09253012 | 59.72450942 |
| GOTERM_BP_DIRECT | GO:0002934~desmosome organization | DSP, PERP, GRHL1 | 8.09253012 | 59.72450942 |
| GOTERM_BP_DIRECT | GO:2001199~negative regulation of dendritic cell differentiation | ZBTB46, TMEM176B, TMEM176A | 8.09253012 | 59.72450942 |
| GOTERM_BP_DIRECT | GO:0009597~detection of virus | DDX58, IFIH1, TLR3 | 8.09253012 | 59.72450942 |
| GOTERM_BP_DIRECT | GO:0042403~thyroid hormone metabolic process | GCNT4, DIO2, CRYM | 8.09253012 | 59.72450942 |
| GOTERM_BP_DIRECT | GO:0010757~negative regulation of plasminogen activation | SERPINE2, SERPINE1, THBS1 | 8.09253012 | 59.72450942 |
| GOTERM_BP_DIRECT | GO:0097114~NMDA glutamate receptor clustering | APOE, NLGN1, SHANK3 | 8.09253012 | 59.72450942 |
| GOTERM_BP_DIRECT | GO:0035860~glial cell-derived neurotrophic factor receptor signaling pathway | SULF1, GFRA1, GFRA2 | 8.09253012 | 59.72450942 |
| GOTERM_BP_DIRECT | GO:0007263~nitric oxide mediated signal transduction | CD36, APOE, GUCY1A3, RASD1, MT1X | 3.549355316 | 59.95516016 |
| GOTERM_BP_DIRECT | GO:0043536~positive regulation of blood vessel endothelial cell migration | PRKCA, PRKD1, AMOT, ANGPT1, THBS1 | 3.549355316 | 59.95516016 |
| GOTERM_BP_DIRECT | GO:0001659~temperature homeostasis | FOXO1, NTSR1, ARRDC3, ADORA1, HTR2A | 3.549355316 | 59.95516016 |
| GOTERM_BP_DIRECT | GO:0032689~negative regulation of interferon-gamma production | IL20RB, IL1RL1, TLR4, IL33, LGALS9, PDCD1LG2 | 2.890189329 | 63.5859367 |
| GOTERM_BP_DIRECT | GO:0010033~response to organic substance | WNT5A, CRIP1, AQP9, SQLE, TRPA1, TIMP3 | 2.890189329 | 63.5859367 |
| GOTERM_BP_DIRECT | GO:0021983~pituitary gland development | ALDH1A2, GATA2, MSX1, SRD5A1, HMGA2, PITX1 | 2.890189329 | 63.5859367 |
| GOTERM_BP_DIRECT | GO:0043388~positive regulation of DNA binding | HES1, CEBPG, IGF1, ITGA2, IRF4, PITX2 | 2.890189329 | 63.5859367 |
| GOTERM_BP_DIRECT | GO:0006749~glutathione metabolic process | GSTM2, GGT5, GSTM3, CLIC6, IDH1, CLIC2, GGT1, GSTM5, SOD2 | 2.167641997 | 64.09467369 |
| GOTERM_BP_DIRECT | GO:0042733~embryonic digit morphogenesis | WNT5A, HAND2, HOXA11, ROR2, GJA1, ZBTB16, ALX4, TWIST1, FLVCR1 | 2.167641997 | 64.09467369 |
| GOTERM_BP_DIRECT | GO:0001570~vasculogenesis | FOXF1, NTRK2, TIPARP, AMOT, HEG1, TMEM100, JUNB, PITX2, GJC1 | 2.167641997 | 64.09467369 |
| GOTERM_BP_DIRECT | GO:0050680~negative regulation of epithelial cell proliferation | WNT5A, MTSS1, CDKN2B, EREG, SFRP1, SAV1, GDF5, PTN, PTCH1 | 2.167641997 | 64.09467369 |
| GOTERM_BP_DIRECT | GO:0006869~lipid transport | ABCA8, ABCA10, APOL1, APOD, APOE, APOLD1, TMEM30B, ANO4, ABCA3, APOL4, ABCA5 | 1.952145424 | 64.25999145 |
| GOTERM_BP_DIRECT | GO:0006816~calcium ion transport | KCNN4, TRPC4, CACNG8, ATP2A3, CACNG7, SLC3A2, CCL8, TRPV4, CCL5, CAMK2A, CDH23 | 1.952145424 | 64.25999145 |
| GOTERM_BP_DIRECT | GO:0044267~cellular protein metabolic process | SNCAIP, IGFBP6, SNCA, CECR1, IGF1, ABCA3, MMP2, MMP1, PAPPA, TGFBI, PAPPA2, IGFBP2, CTSH, IGFBP4, IGFBP5 | 1.714519093 | 64.63542611 |
| GOTERM_BP_DIRECT | GO:0032727~positive regulation of interferon-alpha production | DDX58, IFIH1, IRF7, TLR4 | 4.495850067 | 64.73297859 |
| GOTERM_BP_DIRECT | GO:0030208~dermatan sulfate biosynthetic process | BGN, UST, DSEL, DCN | 4.495850067 | 64.73297859 |
| GOTERM_BP_DIRECT | GO:0036066~protein O-linked fucosylation | ADAMTSL1, ADAMTSL4, THBS1, SPON1 | 4.495850067 | 64.73297859 |
| GOTERM_BP_DIRECT | GO:0009435~NAD biosynthetic process | NAMPT, NMNAT2, KYNU, QPRT | 4.495850067 | 64.73297859 |
| GOTERM_BP_DIRECT | GO:0071872~cellular response to epinephrine stimulus | ATP2B4, STAR, SNCA, SRD5A1 | 4.495850067 | 64.73297859 |
| GOTERM_BP_DIRECT | GO:0032695~negative regulation of interleukin-12 production | CMKLR1, ACP5, JAK3, THBS1 | 4.495850067 | 64.73297859 |
| GOTERM_BP_DIRECT | GO:0007252~I-kappaB phosphorylation | IKBKE, PRDX4, TLR3, TLR4 | 4.495850067 | 64.73297859 |
| GOTERM_BP_DIRECT | GO:0031581~hemidesmosome assembly | LAMA3, ITGA6, LAMC1, PLEC | 4.495850067 | 64.73297859 |
| GOTERM_BP_DIRECT | GO:0015807~L-amino acid transport | SLC7A2, SLC7A5, SLC7A14, SLC43A2 | 4.495850067 | 64.73297859 |
| GOTERM_BP_DIRECT | GO:0035815~positive regulation of renal sodium excretion | EDNRB, EDN1, AVPR1A, NPR1 | 4.495850067 | 64.73297859 |
| GOTERM_BP_DIRECT | GO:0035385~Roundabout signaling pathway | FMOD, OGN, ROBO2, SLIT2 | 4.495850067 | 64.73297859 |
| GOTERM_BP_DIRECT | GO:0043032~positive regulation of macrophage activation | WNT5A, IL1RL1, IL33, THBS1 | 4.495850067 | 64.73297859 |
| GOTERM_BP_DIRECT | GO:0043116~negative regulation of vascular permeability | PTPRJ, AMOT, ANGPT1, SLIT2 | 4.495850067 | 64.73297859 |
| GOTERM_BP_DIRECT | GO:0045664~regulation of neuron differentiation | BCL11B, CDON, SIX1, NLGN1, ZFHX3 | 3.37188755 | 66.17719008 |
| GOTERM_BP_DIRECT | GO:0042474~middle ear morphogenesis | MSX1, EDN1, SIX1, INSIG1, SIX2 | 3.37188755 | 66.17719008 |
| GOTERM_BP_DIRECT | GO:0046697~decidualization | STC2, PTGS2, STC1, JUNB, GJB2 | 3.37188755 | 66.17719008 |
| GOTERM_BP_DIRECT | GO:0006833~water transport | AQP9, PDPN, AQP7, AQP1, AQP3 | 3.37188755 | 66.17719008 |
| GOTERM_BP_DIRECT | GO:0070588~calcium ion transmembrane transport | SLC8A1, TRPC4, CACNG8, CACNG7, TRPV2, TRPA1, CACNB2, GRIN3B, CACNB4, ATP2B4, MCOLN3, ATP2A3, CACNA1G, TRPV4, PKDREJ | 1.70011137 | 66.82296009 |
| GOTERM_BP_DIRECT | GO:0043401~steroid hormone mediated signaling pathway | PGR, PPARG, NR3C2, NR4A2, ESR1, PAQR8, RORB, RORA, NR4A3 | 2.12961319 | 67.38771299 |
| GOTERM_BP_DIRECT | GO:0060291~long-term synaptic potentiation | SERPINE2, PLK2, NTRK2, SNCA, NLGN1, PTN, SHANK3 | 2.484548721 | 68.03762943 |
| GOTERM_BP_DIRECT | GO:0001568~blood vessel development | DLX3, ALDH1A2, FOXF1, DHCR7, FOXO1, AHR, FLVCR1 | 2.484548721 | 68.03762943 |
| GOTERM_BP_DIRECT | GO:0006469~negative regulation of protein kinase activity | ASPN, PTPRC, IL6, PODN, RTN4RL1, SOCS2, PRKAG2, TRIB3, DCN, LRRC15, TRIB2, SH3BP5, BGN | 1.771092451 | 69.89798098 |
| GOTERM_BP_DIRECT | GO:0010862~positive regulation of pathway-restricted SMAD protein phosphorylation | INHBB, ACVR2A, INHBA, GDF5, TGFB3, GDF10, GDF15, BMP6 | 2.247925033 | 69.95138091 |
| GOTERM_BP_DIRECT | GO:0001701~in utero embryonic development | RARRES2, ARNT2, EDN1, JAG2, TGFB3, GJA1, CHD7, MSX1, HAND2, FOXF1, AMOT, APBA2, HEG1, PTCH1, ETNK2, ANGPT1, TMEM100, PITX2, FLVCR1, TWIST1, MYH10 | 1.514644675 | 70.04164718 |
| GOTERM_BP_DIRECT | GO:0007568~aging | IL6, CCL2, HMGCR, FADS1, SNCA, TGFB3, DCN, TIMP1, VCAM1, EDNRB, FOS, TNFRSF1B, APOD, PENK, UCP2, P2RY1, IGFBP2, HTR2A, IGFBP5 | 1.553111841 | 70.8346629 |
| GOTERM_BP_DIRECT | GO:0030308~negative regulation of cell growth | DCBLD2, PTPRJ, BST2, ENPP1, PPARG, GJA1, NPR1, BDKRB1, SLIT2, INHBA, MSX1, SFRP1, SERPINE2, TNK1, SERTAD1 | 1.672010355 | 71.0441069 |
| GOTERM_BP_DIRECT | GO:0010976~positive regulation of neuron projection development | WNT5A, PRKD1, RGMA, IL6, LTK, STMN2, NTRK2, PLXNB3, CNTN1, PTN, DPYSL3, CAMK1D | 1.818546094 | 71.22253251 |
| GOTERM_BP_DIRECT | GO:0035249~synaptic transmission, glutamatergic | SLC17A7, SLC1A4, CNIH3, GRIK2, SHC3 | 3.211321476 | 71.90987241 |
| GOTERM_BP_DIRECT | GO:0048844~artery morphogenesis | NOTCH3, HES1, HOXA1, NRP1, APOE | 3.211321476 | 71.90987241 |
| GOTERM_BP_DIRECT | GO:0035902~response to immobilization stress | FOS, SLC8A1, LRP11, PPARG, SOD2 | 3.211321476 | 71.90987241 |
| GOTERM_BP_DIRECT | GO:0097150~neuronal stem cell population maintenance | HES1, IGF2BP1, FOXO1, CDH2, MMP24 | 3.211321476 | 71.90987241 |
| GOTERM_BP_DIRECT | GO:0031016~pancreas development | HES1, ALDH1A2, ACVR2B, MEIS2, FOXF1 | 3.211321476 | 71.90987241 |
| GOTERM_BP_DIRECT | GO:0042346~positive regulation of NF-kappaB import into nucleus | PTGS2, C8ORF4, TLR3, TLR4, LGALS9 | 3.211321476 | 71.90987241 |
| GOTERM_BP_DIRECT | GO:0030199~collagen fibril organization | FMOD, COL14A1, COL12A1, LOX, COL11A2, MMP11, DPT | 2.420842344 | 71.96402535 |
| GOTERM_BP_DIRECT | GO:0000302~response to reactive oxygen species | NOX4, APOD, APOE, GPX3, TXNRD1, GPX7, SOD2 | 2.420842344 | 71.96402535 |
| GOTERM_BP_DIRECT | GO:0008277~regulation of G-protein coupled receptor protein signaling pathway | RGS20, RGS2, RGS3, RGS4, GRK5, RGS16, GNG7 | 2.420842344 | 71.96402535 |
| GOTERM_BP_DIRECT | GO:0050901~leukocyte tethering or rolling | VCAM1, PODXL2, ITGA4, GCNT1 | 4.150015446 | 72.49874943 |
| GOTERM_BP_DIRECT | GO:0050806~positive regulation of synaptic transmission | SYT1, SLC1A3, CCL2, GRIK2 | 4.150015446 | 72.49874943 |
| GOTERM_BP_DIRECT | GO:0009992~cellular water homeostasis | AQP9, AQP7, AQP1, AQP3 | 4.150015446 | 72.49874943 |
| GOTERM_BP_DIRECT | GO:0007512~adult heart development | CHD7, HAND2, GJA1, MYH10 | 4.150015446 | 72.49874943 |
| GOTERM_BP_DIRECT | GO:0006957~complement activation, alternative pathway | C3, CFB, CFH, CFD | 4.150015446 | 72.49874943 |
| GOTERM_BP_DIRECT | GO:0045019~negative regulation of nitric oxide biosynthetic process | ATP2B4, CD34, ACP5, ZC3H12A | 4.150015446 | 72.49874943 |
| GOTERM_BP_DIRECT | GO:0015793~glycerol transport | AQP9, AQP7, AQP1, AQP3 | 4.150015446 | 72.49874943 |
| GOTERM_BP_DIRECT | GO:0051926~negative regulation of calcium ion transport | ICAM1, PTGS2, ADRA2A, STC1 | 4.150015446 | 72.49874943 |
| GOTERM_BP_DIRECT | GO:0071318~cellular response to ATP | CCL2, PTGS2, PDXP, CIB2 | 4.150015446 | 72.49874943 |
| GOTERM_BP_DIRECT | GO:0006809~nitric oxide biosynthetic process | SLC7A2, ARG2, RORA, GCH1 | 4.150015446 | 72.49874943 |
| GOTERM_BP_DIRECT | GO:0098586~cellular response to virus | IKBKE, PENK, ZC3H12A, LGALS9 | 4.150015446 | 72.49874943 |
| GOTERM_BP_DIRECT | GO:0045776~negative regulation of blood pressure | OPRL1, BDKRB1, ADM2, ADORA1, APLN, GCH1 | 2.69751004 | 72.98647269 |
| GOTERM_BP_DIRECT | GO:0032480~negative regulation of type I interferon production | DDX58, IKBKE, IFIH1, ISG15, HERC5, TNFAIP3 | 2.69751004 | 72.98647269 |
| GOTERM_BP_DIRECT | GO:0042572~retinol metabolic process | AKR1C3, ALDH1A1, ALDH1A2, DHRS3, ALDH1A3, RDH5 | 2.69751004 | 72.98647269 |
| GOTERM_BP_DIRECT | GO:2000379~positive regulation of reactive oxygen species metabolic process | NOX4, AKR1C3, CD36, RIPK3, ZC3H12A, THBS1 | 2.69751004 | 72.98647269 |
| GOTERM_BP_DIRECT | GO:0009058~biosynthetic process | GMPPB, SPTLC3, ACCS, FASN, PCYT2, FDFT1 | 2.69751004 | 72.98647269 |
| GOTERM_BP_DIRECT | GO:0019800~peptide cross-linking via chondroitin 4-sulfate glycosaminoglycan | BGN, SPOCK2, DCN | 6.7437751 | 73.07191492 |
| GOTERM_BP_DIRECT | GO:2000347~positive regulation of hepatocyte proliferation | XBP1, PTN, TNFAIP3 | 6.7437751 | 73.07191492 |
| GOTERM_BP_DIRECT | GO:0061299~retina vasculature morphogenesis in camera-type eye | RHOJ, NRP1, NDP | 6.7437751 | 73.07191492 |
| GOTERM_BP_DIRECT | GO:0006929~substrate-dependent cell migration | FMNL1, ITGA11, ITGA2 | 6.7437751 | 73.07191492 |
| GOTERM_BP_DIRECT | GO:0042985~negative regulation of amyloid precursor protein biosynthetic process | ABCA7, BACE2, ITM2C | 6.7437751 | 73.07191492 |
| GOTERM_BP_DIRECT | GO:0060536~cartilage morphogenesis | MSX1, HAND1, HAND2 | 6.7437751 | 73.07191492 |
| GOTERM_BP_DIRECT | GO:2000052~positive regulation of non-canonical Wnt signaling pathway | WNT5A, SFRP1, RSPO3 | 6.7437751 | 73.07191492 |
| GOTERM_BP_DIRECT | GO:0010042~response to manganese ion | PTGS2, ADAM9, SOD2 | 6.7437751 | 73.07191492 |
| GOTERM_BP_DIRECT | GO:0060087~relaxation of vascular smooth muscle | RGS2, GUCY1A3, ADORA1 | 6.7437751 | 73.07191492 |
| GOTERM_BP_DIRECT | GO:0002819~regulation of adaptive immune response | IRF7, RIPK3, IRF1 | 6.7437751 | 73.07191492 |
| GOTERM_BP_DIRECT | GO:0007185~transmembrane receptor protein tyrosine phosphatase signaling pathway | PTPRD, PTPRF, PTN | 6.7437751 | 73.07191492 |
| GOTERM_BP_DIRECT | GO:0003158~endothelium development | CD34, GJA1, SLC40A1 | 6.7437751 | 73.07191492 |
| GOTERM_BP_DIRECT | GO:0002686~negative regulation of leukocyte migration | HOXA7, IL33, ADORA1 | 6.7437751 | 73.07191492 |
| GOTERM_BP_DIRECT | GO:0071560~cellular response to transforming growth factor beta stimulus | WNT5A, NOX4, SFRP1, PENK, STAR, CLEC3B, EDN1, POSTN | 2.202049012 | 73.32413248 |
| GOTERM_BP_DIRECT | GO:0043406~positive regulation of MAP kinase activity | AJUBA, NOX4, FLT1, EDN1, ELANE, PDE5A, ADRA2A, CD24, HTR2A | 2.057422912 | 73.57138189 |
| GOTERM_BP_DIRECT | GO:0042593~glucose homeostasis | CEBPA, IL6, LEPR, PPARG, MLXIPL, SOX4, NMB, OAS1, GCK, ADRA2A, TRPV4, PTCH1, IGFBP5 | 1.736021313 | 74.38112816 |
| GOTERM_BP_DIRECT | GO:0042060~wound healing | WNT5A, DCBLD2, IL6, EREG, TNC, TGFB3, DSP, SCARB1, LOX, DCN, TIMP1 | 1.854538153 | 74.82861563 |
| GOTERM_BP_DIRECT | GO:0010508~positive regulation of autophagy | PLK2, XBP1, TFEB, FOXO1, ZC3H12A, DCN, MID2 | 2.360321285 | 75.63261389 |
| GOTERM_BP_DIRECT | GO:0048468~cell development | MAF, INHBB, INHBA, GDF5, GDF10, GDF15, GJC1 | 2.360321285 | 75.63261389 |
| GOTERM_BP_DIRECT | GO:0098869~cellular oxidant detoxification | GSTM2, PTGS2, APOE, GPX3, PRDX4, DUOX1, CLIC2, TXNRD1, GPX7, MGST2 | 1.926792886 | 75.80771921 |
| GOTERM_BP_DIRECT | GO:0051289~protein homotetramerization | SHMT1, SHMT2, GBP5, GPX3, TRPA1, ACACA, TP63, TK1, SOD2 | 2.02313253 | 76.42612548 |
| GOTERM_BP_DIRECT | GO:0050679~positive regulation of epithelial cell proliferation | CYP7B1, IL6, SFRP1, IGF1, NR4A3, LAMC1, CCL5, TWIST1, BMP6 | 2.02313253 | 76.42612548 |
| GOTERM_BP_DIRECT | GO:0007157~heterophilic cell-cell adhesion via plasma membrane cell adhesion molecules | ALCAM, VCAM1, ICAM1, AMIGO2, PTPRD, CADM1, NLGN1, CDH2 | 2.158008032 | 76.49317922 |
| GOTERM_BP_DIRECT | GO:0035556~intracellular signal transduction | PRKAG3, CXCL1, ADCY4, TUFT1, EDN1, PREX2, PRKAG2, MCF2L, PKN3, TIAM2, WNK4, PLCD3, GUCY1A3, RAPGEF5, RAPGEF3, SIK1, SHC2, DCLK1, PRKCA, ARHGEF4, TNIK, SOCS2, ARHGEF5, MLXIPL, NPR1, NFAM1, MARK1, SH3BP5, PRKD1, HUNK, FMN2, DUSP1, MAPK13, RGS7, JAK3, PLA2G4C, NRBP2, KALRN, IGFBP5 | 1.305246794 | 76.88821734 |
| GOTERM_BP_DIRECT | GO:0051000~positive regulation of nitric-oxide synthase activity | APOE, ESR1, SCARB1, NPR3, GCH1 | 3.065352318 | 77.06447689 |
| GOTERM_BP_DIRECT | GO:0030032~lamellipodium assembly | AJUBA, ABLIM1, ARHGEF4, CDH13, VAV3, FGD4 | 2.610493587 | 77.14838851 |
| GOTERM_BP_DIRECT | GO:0010332~response to gamma radiation | PTPRC, CCL2, TP63, CCL7, SOD2, CXCL10 | 2.610493587 | 77.14838851 |
| GOTERM_BP_DIRECT | GO:0001933~negative regulation of protein phosphorylation | ZBED3, SNCA, CD109, ZC3H12A, ANGPT1, BDKRB1, MICAL1, MYADM, SLIT2 | 1.989966423 | 79.10431947 |
| GOTERM_BP_DIRECT | GO:0072001~renal system development | ITGA6, FOXF1, EMX2, PTCH1 | 3.853585772 | 79.13207926 |
| GOTERM_BP_DIRECT | GO:0030194~positive regulation of blood coagulation | CD36, ENPP4, SERPINE1, THBS1 | 3.853585772 | 79.13207926 |
| GOTERM_BP_DIRECT | GO:0042554~superoxide anion generation | NOX4, EDN1, DUOX1, SOD2 | 3.853585772 | 79.13207926 |
| GOTERM_BP_DIRECT | GO:0010875~positive regulation of cholesterol efflux | ABCA7, APOE, NFKBIA, PTCH1 | 3.853585772 | 79.13207926 |
| GOTERM_BP_DIRECT | GO:0048148~behavioral response to cocaine | SNCA, SDK1, HTR2A, KALRN | 3.853585772 | 79.13207926 |
| GOTERM_BP_DIRECT | GO:0071277~cellular response to calcium ion | WNT5A, AKR1C3, SYT1, FOS, EDN1, CPNE7, FOSB, JUNB | 2.115694149 | 79.44158151 |
| GOTERM_BP_DIRECT | GO:0006813~potassium ion transport | KCNMB4, KCNJ15, KCNN4, KCND3, ATP1B1, KCNS2, KCNQ3, KCNAB1, KCNA3, AQP1, KCNG1 | 1.809305515 | 79.44481598 |
| GOTERM_BP_DIRECT | GO:0007268~chemical synaptic transmission | SNCG, KCNMB4, SLC12A7, SYT1, PTPRD, PCDHB5, NRXN2, PTPRF, GRIK2, OPRL1, CACNB2, PCDHB14, GRIA4, CACNB4, NTSR1, GJC1, NPTX1, KCNQ3, SLC1A3, PENK, GRIN2D, HTR7, CACNA1G, APBA2, HTR2A | 1.404953146 | 80.03473667 |
| GOTERM_BP_DIRECT | GO:1902476~chloride transmembrane transport | SLC17A7, SLC1A4, SLC12A7, APOL1, GABRB3, CLIC6, CLIC2, ANO4, PCYOX1, SLC26A1, SLC4A3, CLCN4 | 1.740329058 | 80.10216254 |
| GOTERM_BP_DIRECT | GO:0001649~osteoblast differentiation | ALPL, SFRP1, PENK, TNC, SEMA7A, FASN, ITGA11, GJA1, GDF10, JUNB, TWIST1, BMP6, IGFBP5 | 1.685943775 | 80.42918313 |
| GOTERM_BP_DIRECT | GO:0006694~steroid biosynthetic process | STAR, CYP51A1, SRD5A1, LSS, FDFT1, HSD17B8 | 2.528915663 | 80.90413556 |
| GOTERM_BP_DIRECT | GO:0045907~positive regulation of vasoconstriction | ICAM1, PTGS2, AVPR1A, GJA1, ADRA1D, HTR2A | 2.528915663 | 80.90413556 |
| GOTERM_BP_DIRECT | GO:0031663~lipopolysaccharide-mediated signaling pathway | CCL2, HCK, NFKBIA, SCARB1, TLR4, CCL5 | 2.528915663 | 80.90413556 |
| GOTERM_BP_DIRECT | GO:0035115~embryonic forelimb morphogenesis | ALDH1A2, MSX1, HOXA11, TFAP2A, ALX4, TWIST1 | 2.528915663 | 80.90413556 |
| GOTERM_BP_DIRECT | GO:0050873~brown fat cell differentiation | CEBPA, RARRES2, ITGA6, RGS2, PTGS2, EBF2 | 2.528915663 | 80.90413556 |
| GOTERM_BP_DIRECT | GO:0007507~heart development | SH3PXD2B, CRIP1, KCNAB1, PPARG, EDN1, SOX4, GJA1, ARMC4, SOD2, GATA2, ACVR2B, ECE2, BVES, HAND1, HAND2, FOXF1, PTN, HEG1, ADAM19, LOX | 1.474049202 | 81.19675292 |
| GOTERM_BP_DIRECT | GO:0006919~activation of cysteine-type endopeptidase activity involved in apoptotic process | COL4A3, IFI27, TNFSF10, HIP1R, F3, PPARG, SNCA, EGLN3, FAS, CTSH, HIP1 | 1.787506653 | 81.55720921 |
| GOTERM_BP_DIRECT | GO:0001934~positive regulation of protein phosphorylation | RARRES2, TNIK, FAM20A, ROCK2, C3, OPRL1, IL34, EDNRB, ACVR2A, ARRB1, XBP1, NTRK2, P2RY1, CLIP3, SEMA4D | 1.59301774 | 82.0724497 |
| GOTERM_BP_DIRECT | GO:0071333~cellular response to glucose stimulus | SLC29A1, NOX4, ICAM1, STAR, XBP1, UCP2, SOX4, PCK2 | 2.075007723 | 82.15773329 |
| GOTERM_BP_DIRECT | GO:0051091~positive regulation of sequence-specific DNA binding transcription factor activity | DDX58, IL6, SAV1, CEBPG, NDP, ARHGEF5, EDN1, PPARG, ESR1, NFAM1, HMGA2, ANXA3, MID2 | 1.669887168 | 82.24443271 |
| GOTERM_BP_DIRECT | GO:0009791~post-embryonic development | ACVR2B, DHCR7, TIPARP, SCN9A, NR4A2, HEG1, SEMA3C, ETNK2, ALX4, SOD2 | 1.847609617 | 82.8400333 |
| GOTERM_BP_DIRECT | GO:2000427~positive regulation of apoptotic cell clearance | CCL2, C3, C2 | 5.780378657 | 82.96129688 |
| GOTERM_BP_DIRECT | GO:0060732~positive regulation of inositol phosphate biosynthetic process | ADCYAP1R1, SNCA, NTSR1 | 5.780378657 | 82.96129688 |
| GOTERM_BP_DIRECT | GO:0046850~regulation of bone remodeling | LEPR, GJA1, SYT7 | 5.780378657 | 82.96129688 |
| GOTERM_BP_DIRECT | GO:0043305~negative regulation of mast cell degranulation | FOXF1, LGALS9, IL13RA2 | 5.780378657 | 82.96129688 |
| GOTERM_BP_DIRECT | GO:0044539~long-chain fatty acid import | CD36, FABP3, ACSL3 | 5.780378657 | 82.96129688 |
| GOTERM_BP_DIRECT | GO:0044321~response to leptin | STAR, LEPR, EDN1 | 5.780378657 | 82.96129688 |
| GOTERM_BP_DIRECT | GO:0019885~antigen processing and presentation of endogenous peptide antigen via MHC class I | TAP1, HLA-A, ERAP2 | 5.780378657 | 82.96129688 |
| GOTERM_BP_DIRECT | GO:0060740~prostate gland epithelium morphogenesis | CYP7B1, TNC, ID4 | 5.780378657 | 82.96129688 |
| GOTERM_BP_DIRECT | GO:1902259~regulation of delayed rectifier potassium channel activity | KCNS2, KCNAB1, KCNG1 | 5.780378657 | 82.96129688 |
| GOTERM_BP_DIRECT | GO:1901385~regulation of voltage-gated calcium channel activity | CACNB2, CACNB4, AHNAK | 5.780378657 | 82.96129688 |
| GOTERM_BP_DIRECT | GO:0045606~positive regulation of epidermal cell differentiation | SFRP4, PTCH1, PTCH2 | 5.780378657 | 82.96129688 |
| GOTERM_BP_DIRECT | GO:0071918~urea transmembrane transport | AQP9, AQP7, AQP3 | 5.780378657 | 82.96129688 |
| GOTERM_BP_DIRECT | GO:0042908~xenobiotic transport | ABCA8, SLC2A1, ABCB4 | 5.780378657 | 82.96129688 |
| GOTERM_BP_DIRECT | GO:0045650~negative regulation of macrophage differentiation | INHBA, GATA2, ZBTB46 | 5.780378657 | 82.96129688 |
| GOTERM_BP_DIRECT | GO:0010642~negative regulation of platelet-derived growth factor receptor signaling pathway | PTPRJ, APOD, SNCA | 5.780378657 | 82.96129688 |
| GOTERM_BP_DIRECT | GO:0045656~negative regulation of monocyte differentiation | ZBTB46, HOXA7, GPR68 | 5.780378657 | 82.96129688 |
| GOTERM_BP_DIRECT | GO:0043567~regulation of insulin-like growth factor receptor signaling pathway | IGFBP6, IGFBP2, IGFBP4 | 5.780378657 | 82.96129688 |
| GOTERM_BP_DIRECT | GO:0014009~glial cell proliferation | PENK, LGI4, SOX4 | 5.780378657 | 82.96129688 |
| GOTERM_BP_DIRECT | GO:0071639~positive regulation of monocyte chemotactic protein-1 production | TRPV4, LGALS9, TWIST1 | 5.780378657 | 82.96129688 |
| GOTERM_BP_DIRECT | GO:0000122~negative regulation of transcription from RNA polymerase II promoter | E2F7, SNCA, PPARG, EDN1, TP63, FOXO1, GATA2, EDNRB, HOXC8, PCBP3, POU5F1, FOXF1, TRPV4, BHLHE41, PITX2, TWIST1, EGR1, RELB, ELANE, MLXIPL, ESR1, ZHX2, FOSB, HMGA2, MSC, JUNB, HES1, ACVR2B, CD36, MSX1, SIX1, TFAP2A, SEMA4D, NFE2L3, GLIS3, TSHZ3, ETV7, GLIS1, TRIB3, ZBTB16, AHRR, MEIS2, HAND1, XBP1, HOXA7, NFATC2, DNMT3B, MAF, CEBPA, NR4A2, NR4A3, SREBF2, NOTCH3, AJUBA, IFI27, DKK1, PLK1, IRF7, H2AFY2, PTCH1, ID4, ZFHX3, CRYM, BMP6 | 1.198893351 | 84.20037344 |
| GOTERM_BP_DIRECT | GO:0002931~response to ischemia | EGR1, HYOU1, EIF4EBP1, BVES, HK2, CAMK2A | 2.452281855 | 84.23870621 |
| GOTERM_BP_DIRECT | GO:2000679~positive regulation of transcription regulatory region DNA binding | HAND2, IGF1, HMGA2, TWIST1 | 3.596680054 | 84.58424713 |
| GOTERM_BP_DIRECT | GO:0006636~unsaturated fatty acid biosynthetic process | FADS1, SCD, FADS2, ELOVL6 | 3.596680054 | 84.58424713 |
| GOTERM_BP_DIRECT | GO:0046928~regulation of neurotransmitter secretion | KCNMB4, SNCG, SNCAIP, CAMK2A | 3.596680054 | 84.58424713 |
| GOTERM_BP_DIRECT | GO:0048485~sympathetic nervous system development | PLXNA4, HAND2, TP63, SOX4 | 3.596680054 | 84.58424713 |
| GOTERM_BP_DIRECT | GO:0014850~response to muscle activity | ADSSL1, ITGA2, POSTN, SRD5A1 | 3.596680054 | 84.58424713 |
| GOTERM_BP_DIRECT | GO:0051044~positive regulation of membrane protein ectodomain proteolysis | TNFRSF1B, APOE, ADRA2A, ADAM9 | 3.596680054 | 84.58424713 |
| GOTERM_BP_DIRECT | GO:0045429~positive regulation of nitric oxide biosynthetic process | ICAM1, IL6, PTGS2, EDN1, ESR1, TLR4, SOD2 | 2.195647707 | 84.88138832 |
| GOTERM_BP_DIRECT | GO:0034260~negative regulation of GTPase activity | F11R, SPRY1, ARRB1, IPO5, PLXNB3, AMOT, SLIT2 | 2.195647707 | 84.88138832 |
| GOTERM_BP_DIRECT | GO:0009636~response to toxic substance | CDK1, FOS, PENK, MAOB, SCN9A, ASNS, FAS, CCL5, DNMT3B, INMT, AHR | 1.745447673 | 85.36959938 |
| GOTERM_BP_DIRECT | GO:0034341~response to interferon-gamma | KYNU, BST2, IFITM1, SNCA, GCH1 | 2.809906292 | 85.47499957 |
| GOTERM_BP_DIRECT | GO:0045671~negative regulation of osteoclast differentiation | SFRP1, MAFB, FSTL3, TLR3, TLR4 | 2.809906292 | 85.47499957 |
| GOTERM_BP_DIRECT | GO:0030866~cortical actin cytoskeleton organization | FMNL1, FMNL2, EPB41L1, ROCK2, RHOQ | 2.809906292 | 85.47499957 |
| GOTERM_BP_DIRECT | GO:0042632~cholesterol homeostasis | TMEM97, LPL, LDLR, APOE, XBP1, FABP3, PCSK9, SCARB1, CD24 | 1.896686747 | 86.00182158 |

1. GO_MolecularFunction

| Category | Term | Genes | Fold Enrichment | FDR |
| --- | --- | --- | --- | --- |
| GOTERM_MF_DIRECT | GO:0008201~heparin binding | FMOD, CCL2, NRP1, LXN, PGF, ADAMTS15, CCL8, POSTN, CXCL6, MDK, ABI3BP, CCL7, CXCL10, OGN, RSPO4, SERPINE2, APOE, RSPO3, COMP, CFH, PTN, THBS1, THBS4, LPL, PTPRF, ELANE, CECR1, CCDC80, ECM2, SLIT2, ADAMTS8, SFRP1, CLEC3B, ADAMTS1, PTCH1 | 2.997336648 | 1.92E-05 |
| GOTERM_MF_DIRECT | GO:0004222~metalloendopeptidase activity | ADAMTSL4, ADAMTS15, MMP27, MMP2, MMP1, MMP24, ECE2, PAPPA, ADAMTS10, ADAM9, ADAM11, ADAM23, PAPLN, MMP11, ADAMTS6, MMP10, ADAMTS8, THSD4, PAPPA2, ADAMTS1, ADAM19, ADAM22, PHEX, ADAM12, ADAMDEC1 | 3.031440352 | 0.002673286 |
| GOTERM_MF_DIRECT | GO:0005178~integrin binding | COL4A3, ICAM1, ADAM11, ICAM5, EGFL6, ADAM23, UTRN, ITGA2, IGF1, EDIL3, ECM2, VCAM1, S1PR3, ADAMTS8, ITGA6, LAMA5, SEMA7A, TGFBI, ADAM22, THBS1, CIB2, THBS4, ADAM9 | 3.001414657 | 0.009070523 |
| GOTERM_MF_DIRECT | GO:0050840~extracellular matrix binding | BGN, FBLN2, SPOCK2, OLFML2B, TGFBI, ELN, ADAMTS15, OLFML2A, DCN, THBS1, SSC5D | 5.797046703 | 0.012062881 |
| GOTERM_MF_DIRECT | GO:0001077~transcriptional activator activity, RNA polymerase II core promoter proximal region sequence-specific binding | ARNT2, SOX4, TP63, ZIC1, PGR, FOS, GATA2, MEIS2, HAND2, BCL11B, POU2F2, HOXA7, ETV1, NFATC2, PITX1, PITX2, CEBPA, EGR1, EGR2, MAFB, CEBPG, NR4A2, ESR1, SIX2, FOSB, NR4A3, GRHL1, HMGA2, JUNB, DLX3, DBP, MEOX1, EBF2, SIX1, EBF1, IRF1, TFAP2A, IRF4 | 2.206272012 | 0.012879831 |
| GOTERM_MF_DIRECT | GO:0016491~oxidoreductase activity | HSD17B10, CYP3A5, ADHFE1, SNCA, PRDX4, EGLN3, HR, IL4I1, FDFT1, ALDH1A1, AKR1C3, ALDH1A2, ALDH1A3, FASN, MICAL1, SARDH, BDH1, GFOD1, NOX4, MSMO1, DHRS13, FADS1, SCD, MAOA, MAOB, VAT1L, SQLE, RRM2, HAO2, AKR1B1, AOX1, HSD11B1, TXNRD1, HEPH | 2.329358766 | 0.013167527 |
| GOTERM_MF_DIRECT | GO:0008083~growth factor activity | CXCL1, CSF3, PGF, NDP, GDF5, JAG2, TGFB3, FGF11, IL34, MDK, IL11, TIMP1, OGN, BDNF, TYMP, CDNF, PTN, THBS4, IL6, IL7, CECR1, IGF1, INHBB, INHBA, DKK1, EREG, GDF10, GDF15, BMP6 | 2.452846922 | 0.025988527 |
| GOTERM_MF_DIRECT | GO:0008237~metallopeptidase activity | ERMP1, ADAM11, TRHDE, ADAMTSL1, ADAM23, ENPEP, MMP2, ADAMTS6, ACE, NAALAD2, ADAMTS8, PAPPA, ADAMTS10, ADAMTS1, ERAP2, PAPPA2, ADAM12, ADAM9 | 3.04491342 | 0.100457087 |
| GOTERM_MF_DIRECT | GO:0005201~extracellular matrix structural constituent | COL4A4, COL4A3, MATN3, ELN, COL15A1, CHI3L1, MGP, BGN, COL14A1, FBLN2, COMP, CD4, LAMC1, FBN2, COL11A2, COL8A2 | 3.272145765 | 0.123318055 |
| GOTERM_MF_DIRECT | GO:0005125~cytokine activity | WNT5A, CSF3, NAMPT, IL16, FAM3C, NDP, CTF1, EDN1, GDF5, CRLF1, TGFB3, IL32, IL33, IL34, IL11, TIMP1, C1QTNF4, SECTM1, IL6, IL7, IL1RN, INHBB, INHBA, TNFSF10, TNFSF13B, GDF10, GDF15, BMP6 | 2.179881198 | 0.300893871 |
| GOTERM_MF_DIRECT | GO:0043236~laminin binding | LYPD3, ITGA6, ITGA2, BCAM, CTSS, THBS1, LRRC15, SSC5D, ADAM9 | 4.93275974 | 0.448628629 |
| GOTERM_MF_DIRECT | GO:0005518~collagen binding | ASPN, PODN, ITGA11, ITGA2, CTSS, DCN, LRRC15, ABI3BP, COL14A1, C1QTNF1, COMP, TGFBI, SRGN, ADAM9 | 3.197159091 | 0.516077634 |
| GOTERM_MF_DIRECT | GO:0030169~low-density lipoprotein particle binding | CDH13, CD36, LDLR, PCSK9, SCARB1, COLEC12, THBS1 | 6.394318182 | 0.682070977 |
| GOTERM_MF_DIRECT | GO:0042605~peptide antigen binding | TAP1, HLA-A, HLA-C, HLA-DPA1, HLA-B, SLC7A5, HLA-DRA, HLA-F, DHCR24 | 4.404249768 | 1.056220675 |
| GOTERM_MF_DIRECT | GO:0001730~2'-5'-oligoadenylate synthetase activity | OASL, OAS3, OAS1, OAS2 | 13.70211039 | 2.364141232 |
| GOTERM_MF_DIRECT | GO:0001618~virus receptor activity | ICAM1, F11R, LDLR, EFNB3, ITGA2, ITGB5, HSPA1A, HSPA1B, NCAM1, CXCR4, CD46, CD4, SCARB1, HTR2A | 2.740422078 | 2.383295968 |
| GOTERM_MF_DIRECT | GO:0019955~cytokine binding | NRP1, POU5F1, CXCR4, CRLF1, ELANE, CD74, IL31RA | 5.048145933 | 2.841534334 |
| GOTERM_MF_DIRECT | GO:0001968~fibronectin binding | CCDC80, FSTL3, ITGA4, CTSS, THBS1, LRRC15, SSC5D, IGFBP5 | 4.216033966 | 3.326927234 |
| GOTERM_MF_DIRECT | GO:0005509~calcium ion binding | ASPN, SYT1, MASP1, LDLR, MASP2, SUSD1, SNCA, JAG2, MMP27, DUOX1, EDIL3, SYT7, MMP1, MMP24, KCNIP3, PCDH1, DYSF, MCTP2, CIB2, CDH24, CDH23, MATN3, F10, SCUBE3, PCDHB5, EGFL6, NCALD, MGP, PCDHGB6, PCDH7, SLIT2, MCTP1, MMP11, RAB11FIP4, MMP10, PLA2G2A, LCP1, PKDREJ, EPDR1, ENPP1, SPOCK2, PCDH20, PCDHB14, CDH2, GCH1, MYL9, CAPS2, PRRG3, FAT3, COMP, PLCH1, HEG1, FBN2, THBS1, EHD3, THBS4, SLC8A1, S100A16, CUBN, MEX3B, CELSR3, PCDH17, CAPN2, ANXA3, NOTCH3, CDH13, CLGN, CLEC3B, FBLN2, SULF1, SYT14, SYTL2, MEGF6 | 1.395054475 | 5.482501398 |
| GOTERM_MF_DIRECT | GO:0071813~lipoprotein particle binding | CDH13, CD36, APOE, MAPT | 10.96168831 | 5.504548221 |
| GOTERM_MF_DIRECT | GO:0005102~receptor binding | CXCL1, MTSS1, RARRES2, CCL2, NXPH4, CADM1, C3, IGFBP6, GJA1, HSPA1A, HSPA1B, CXCL10, PGR, ALCAM, GSTM2, SERPINE2, RSPO3, SERPINE1, IDH1, ANGPTL1, APLN, ANGPT2, LPL, PTPRD, HCK, HLA-A, HLA-B, HLA-F, TNFSF10, LAMA3, TNFSF13B, CD58, HAO2, GFRA1, TNK1, JAK3, SEMA4D, PCSK1N, IGFBP2, ICOSLG, IGFBP4 | 1.591463246 | 5.745386069 |
| GOTERM_MF_DIRECT | GO:0004896~cytokine receptor activity | IL20RB, IL1RL1, F3, LIFR, IL15RA, CD74, IL17RB, IL31RA, IL13RA2 | 3.425527597 | 5.965496081 |
| GOTERM_MF_DIRECT | GO:0008191~metalloendopeptidase inhibitor activity | COL4A3, BST2, LXN, SPOCK2, TIMP3, TIMP1 | 5.138291396 | 7.14619258 |
| GOTERM_MF_DIRECT | GO:0043565~sequence-specific DNA binding | PPARG, FOXO1, TP63, RORB, PRRX2, RORA, KCNIP3, PGR, FOS, HOXC8, POU5F1, HOXC4, FOXF1, HOXC5, MKX, PITX1, PITX2, EGR1, BATF2, EMX2, ESR1, FOSB, GRHL1, HES1, MSX1, HOXD3, SIX1, TFAP2A, ZSCAN16, NFE2L3, IRX6, IRX5, HOXA13, HOXA11, IRX1, NR3C2, HOXA1, HOXA3, MEIS2, XBP1, BCL11B, POU2F2, HOXA7, ETV1, MAF, CEBPG, NR4A2, NR4A3, DLX3, HOXB7, HOXB8, MEOX1, IRF1, IRF4, PBX4 | 1.454857281 | 7.795492651 |
| GOTERM_MF_DIRECT | GO:0030020~extracellular matrix structural constituent conferring tensile strength | COL9A2, COL9A3, COL12A1, COL11A2 | 9.13474026 | 10.17178993 |
| GOTERM_MF_DIRECT | GO:0097110~scaffold protein binding | ATP2B4, NLGN4Y, KRT8, VIM, P2RY1, CACNA1G, NLGN1, GJA1, DSP, SHANK3 | 2.854606331 | 11.04877518 |
| GOTERM_MF_DIRECT | GO:0005539~glycosaminoglycan binding | PODXL2, BGN, SERPINE2, SPOCK2, DCN, VIT | 4.56737013 | 12.09743665 |
| GOTERM_MF_DIRECT | GO:0004872~receptor activity | ICAM1, CUBN, CADM1, LDLR, RTN4RL1, CMKLR1, ADCYAP1R1, CRLF1, NLGN1, TLR3, ITGB5, TLR4, PAQR4, NOTCH3, EPHB6, NLGN4Y, CD46, P2RY1, ROBO4, AMOT, GUCY1A3, CD4, FAS, SV2A, SEMA4D, SLC40A1, GFRA2 | 1.704870878 | 13.02694557 |
| GOTERM_MF_DIRECT | GO:0031994~insulin-like growth factor I binding | ITGA6, IGFBP6, IGFBP2, IGFBP4, IGFBP5 | 5.709212662 | 13.25743744 |
| GOTERM_MF_DIRECT | GO:0005089~Rho guanyl-nucleotide exchange factor activity | ARHGEF4, PLEKHG1, VAV3, TIAM2, RASGRF2, ARHGEF37, ARHGEF5, PREX2, MCF2L, FGD3, FGD4, ALS2CL, KALRN | 2.313343313 | 14.48960153 |
| GOTERM_MF_DIRECT | GO:0001758~retinal dehydrogenase activity | AKR1C3, ALDH1A1, ALDH1A2, ALDH1A3 | 7.829777365 | 16.30434686 |
| GOTERM_MF_DIRECT | GO:0005216~ion channel activity | TRPV2, TRPA1, CACNA1G, TRPV4, SCN8A, HVCN1, FXYD6, CLCN4, GJC1 | 2.86788357 | 17.16755458 |
| GOTERM_MF_DIRECT | GO:0019838~growth factor binding | RHBDF2, ACVR2A, ACVR2B, NRP1, FLT1, DUSP1, LIFR | 3.55239899 | 17.42861805 |
| GOTERM_MF_DIRECT | GO:0004860~protein kinase inhibitor activity | SH3BP5, ASPN, BGN, PODN, SOCS2, RTN4RL1, TRIB3, DCN, LRRC15, TRIB2 | 2.635021229 | 18.00099153 |
| GOTERM_MF_DIRECT | GO:0050839~cell adhesion molecule binding | VCAM1, CLDN7, PTPRD, CADM1, CPE, NLGN4Y, NRXN2, NLGN1, DSP, POSTN, ITGA4 | 2.431019585 | 20.08973172 |
| GOTERM_MF_DIRECT | GO:0046977~TAP binding | HLA-A, HLA-C, HLA-B | 13.70211039 | 22.09888705 |
| GOTERM_MF_DIRECT | GO:0042803~protein homodimerization activity | NAMPT, KYNU, CADM1, MASP1, PTGS2, GRIK2, PGF, HMGCR, HIP1R, PRDX4, GSTM2, GSTM3, TRIM9, APOE, CIB2, BHLHE41, PITX2, TWIST1, NUDT16, STC2, BST2, MLXIPL, CECR1, ZHX2, OLFML2A, MECOM, NTSR1, SLIT2, INHBB, HES1, RAB11FIP4, RENBP, HSPB6, TFAP2A, ENPP1, MVD, ACCS, NDP, HMGCS1, ASNS, ZBTB16, CCL5, GCH1, HAND1, HAND2, XBP1, ALDH1A3, TAP1, ADRA2A, FASN, IDH1, SCARB1, CD4, CAMK2A, HIP1, CEBPA, SHMT1, ADSSL1, S100A16, CUBN, MAOB, NR4A2, BIRC5, NPR3, NR4A3, MID2, CDH13, NTRK2, QPRT, CRYM | 1.313900996 | 22.58288719 |
| GOTERM_MF_DIRECT | GO:0030676~Rac guanyl-nucleotide exchange factor activity | ARHGEF4, DOCK2, VAV3, PREX2, KALRN | 4.893610853 | 22.64275305 |
| GOTERM_MF_DIRECT | GO:0001786~phosphatidylserine binding | SYT1, SDPR, SYTL2, SCARB1, SYT7, THBS1, MARK1, GAP43 | 2.962618463 | 23.26454301 |
| GOTERM_MF_DIRECT | GO:0031995~insulin-like growth factor II binding | IGFBP6, IGFBP2, IGFBP4, IGFBP5 | 6.851055195 | 23.6799379 |
| GOTERM_MF_DIRECT | GO:0038191~neuropilin binding | SEMA4G, SEMA3G, SEMA7A, SEMA3C, SEMA4D | 4.56737013 | 28.18144568 |
| GOTERM_MF_DIRECT | GO:0005543~phospholipid binding | F10, SNCA, PSD4, DYSF, MAP1LC3A, APOE, ARHGAP44, F3, SDPR, PLA2G2A, GRK5, PLA2G4C, MYOF | 2.071249245 | 30.27447664 |
| GOTERM_MF_DIRECT | GO:0005215~transporter activity | ABCA8, RBP5, ABCA7, ABCA10, CUBN, AQP9, SLCO4A1, CPNE7, AQP7, STARD10, SEC14L4, AQP1, ABCA3, AQP3, ABCA5, APOD, TAP1, FABP3, SCARB1, SLC13A3, SLC15A3, FABP5, KIF20A, FLVCR1 | 1.627973512 | 30.69808553 |
| GOTERM_MF_DIRECT | GO:0000982~transcription factor activity, RNA polymerase II core promoter proximal region sequence-specific binding | FOS, MSX1, BATF2, IRF7, TFAP2A, FOSB | 3.57446358 | 31.40522838 |
| GOTERM_MF_DIRECT | GO:0045236~CXCR chemokine receptor binding | CXCL1, CXCL3, CXCL2, CXCL6 | 6.08982684 | 31.95552973 |
| GOTERM_MF_DIRECT | GO:0008009~chemokine activity | CXCL1, CCL2, CXCL3, CXCL2, CCL8, CXCL6, CCL5, CCL7, CXCL10 | 2.516714153 | 33.07578877 |
| GOTERM_MF_DIRECT | GO:0015250~water channel activity | AQP9, PDPN, AQP7, AQP1, AQP3 | 4.281909497 | 34.134835 |
| GOTERM_MF_DIRECT | GO:0008134~transcription factor binding | LMO4, PPARG, NFKBIA, RORB, RORA, TRIB2, GATA2, FOS, MEIS2, HAND1, HAND2, POU5F1, HOXA7, GPX3, NFATC2, TWIST1, PITX2, CEBPA, MAFB, CEBPG, MLXIPL, ESR1, SIX2, FOSB, HMGA2, JUNB, AHR, HES1, CD34, ARRB1, IRF4 | 1.495652895 | 34.97991789 |
| GOTERM_MF_DIRECT | GO:0003779~actin binding | ABLIM1, MLPH, UTRN, ANLN, ACE, CXCR4, SNTB1, TRPV4, MICAL1, LMOD1, PLEC, SYNPO, FGD4, FMNL2, CAP2, BAIAP2L1, NCALD, CLMN, MYO3B, SYNPO2, FLNB, SHANK3, LSP1, FMN2, EPB41L1, LIMCH1, CAPG, LCP1, MYH10, TMOD1 | 1.478645006 | 41.82814575 |
| GOTERM_MF_DIRECT | GO:0070888~E-box binding | LMO2, HAND2, ATOH8, BHLHE41, AHR, TWIST1, SREBF2 | 2.821022727 | 43.51412329 |
| GOTERM_MF_DIRECT | GO:0071837~HMG box domain binding | EGR2, HOXA3, MEOX1, HOXC4, ALX4 | 3.806141775 | 46.7104227 |
| GOTERM_MF_DIRECT | GO:0043394~proteoglycan binding | CECR1, CTSS, THBS1, SLIT2 | 4.982585596 | 49.54970787 |
| GOTERM_MF_DIRECT | GO:0017046~peptide hormone binding | EDNRB, INHBA, AVPR1A, NPR1, NPR3, SLC40A1 | 3.04491342 | 51.28187154 |
| GOTERM_MF_DIRECT | GO:0004879~RNA polymerase II transcription factor activity, ligand-activated sequence-specific DNA binding | PPARG, NR4A2, ESR1, RORB, RORA, NR4A3, AHR | 2.664299242 | 52.07499777 |
| GOTERM_MF_DIRECT | GO:0004551~nucleotide diphosphatase activity | CILP2, ENPP1, CILP | 8.221266234 | 53.52552782 |
| GOTERM_MF_DIRECT | GO:0016641~oxidoreductase activity, acting on the CH-NH2 group of donors, oxygen as acceptor | LOXL4, LOXL3, LOX | 8.221266234 | 53.52552782 |
| GOTERM_MF_DIRECT | GO:0016740~transferase activity | MOCOS, SHMT1, SHMT2, BST1, SPTLC3, FDPS, OAS3, OAS1, OAS2, BIRC3, FDFT1, OASL, GALNT12 | 1.855494115 | 53.80404344 |
| GOTERM_MF_DIRECT | GO:0003707~steroid hormone receptor activity | PGR, PPARG, NR3C2, NR4A2, ESR1, PAQR8, RORB, RORA, NR4A3 | 2.202124884 | 56.0434921 |
| GOTERM_MF_DIRECT | GO:0000975~regulatory region DNA binding | IRF7, IRF1, IRF4, HMGA2 | 4.56737013 | 58.05886831 |
| GOTERM_MF_DIRECT | GO:0017154~semaphorin receptor activity | NRP1, PLXNA4, PLXNB3, MET | 4.56737013 | 58.05886831 |
| GOTERM_MF_DIRECT | GO:0015254~glycerol channel activity | AQP9, AQP7, AQP1, AQP3 | 4.56737013 | 58.05886831 |
| GOTERM_MF_DIRECT | GO:0001158~enhancer sequence-specific DNA binding | GATA2, ZNF704, POU5F1, XBP1, LMO4 | 3.425527597 | 59.16471628 |
| GOTERM_MF_DIRECT | GO:0051015~actin filament binding | FMNL1, ADSSL1, SHROOM3, SHROOM4, HIP1R, UTRN, CACNB2, NEXN, FLNC, AJUBA, CFL2, TRPV4, LCP1, TMOD1, HIP1, MYH10 | 1.660861865 | 61.95535609 |
| GOTERM_MF_DIRECT | GO:0005044~scavenger receptor activity | ENPP1, PRG4, COLEC12, LOXL4, LOXL3, SSC5D, SCARA5, PRSS12 | 2.283685065 | 62.17794284 |
| GOTERM_MF_DIRECT | GO:0005516~calmodulin binding | SYT1, SLC8A1, PHKG1, IQGAP3, RRAD, SYT7, RGS16, KCNN4, KCNQ3, ATP2B4, RASGRF2, RGS2, RGS4, PDE1A, SNTB1, TRPV4, MAP6, CAMK2A, GAP43, CAMK1D, MYH10 | 1.52245671 | 63.33015405 |
| GOTERM_MF_DIRECT | GO:0044212~transcription regulatory region DNA binding | WNT5A, CEBPA, EGR2, SNCA, ARID5A, PPARG, TFEB, TP63, GRHL1, AHR, SREBF2, FOS, HAND1, HAND2, ARRB1, XBP1, POU5F1, BCL11B, SIX1, H2AFY2, TFAP2A, ETV5, ZFHX3 | 1.479570605 | 64.91056976 |
| GOTERM_MF_DIRECT | GO:0004602~glutathione peroxidase activity | GSTM2, GPX3, CLIC2, GPX7, MGST2 | 3.262407236 | 64.99818276 |
| GOTERM_MF_DIRECT | GO:0000978~RNA polymerase II core promoter proximal region sequence-specific DNA binding | ARNT2, ZIC1, KCNIP3, PGR, FOS, CHD7, MEIS2, HAND1, BCL11B, POU2F2, HOXA7, ETV1, BHLHE41, NFATC2, PITX1, PITX2, CEBPA, EGR2, BATF2, MAFB, MLXIPL, ESR1, SIX2, FOSB, NR4A3, GRHL1, JUNB, SREBF2, MEOX1, EBF2, SIX1, IRF1, TFAP2A, IRF4, NFE2L3 | 1.350912292 | 65.09563026 |
| GOTERM_MF_DIRECT | GO:0015026~coreceptor activity | RGMA, ACVR2A, NRP1, CXCR4, CD4, ITGA4 | 2.740422078 | 65.82700924 |
| GOTERM_MF_DIRECT | GO:0015179~L-amino acid transmembrane transporter activity | SLC7A2, SLC7A5, SLC7A14, SLC43A2 | 4.216033966 | 65.93559347 |
| GOTERM_MF_DIRECT | GO:0008131~primary amine oxidase activity | VCAM1, MAOA, MAOB | 6.851055195 | 66.91225169 |
| GOTERM_MF_DIRECT | GO:0005372~water transmembrane transporter activity | AQP9, PDPN, AQP1 | 6.851055195 | 66.91225169 |
| GOTERM_MF_DIRECT | GO:0010997~anaphase-promoting complex binding | CLSPN, PLK1, CDC20 | 6.851055195 | 66.91225169 |
| GOTERM_MF_DIRECT | GO:0004035~alkaline phosphatase activity | ALPL, CILP2, CILP | 6.851055195 | 66.91225169 |
| GOTERM_MF_DIRECT | GO:0070324~thyroid hormone binding | ALDH1A3, CTSH, CRYM | 6.851055195 | 66.91225169 |
| GOTERM_MF_DIRECT | GO:0016614~oxidoreductase activity, acting on CH-OH group of donors | CHDH, AOX1, DHCR24 | 6.851055195 | 66.91225169 |
| GOTERM_MF_DIRECT | GO:0005262~calcium channel activity | TRPC4, MCOLN3, CACNG7, TRPV2, TRPA1, CACNB2, TRPV4, GRIN3B, CACNB4, PKDREJ | 1.957444341 | 67.8795342 |
| GOTERM_MF_DIRECT | GO:0003725~double-stranded RNA binding | DDX58, IFIH1, OASL, VIM, OAS3, SLC3A2, TLR3, OAS1, OAS2 | 2.021622844 | 71.66899935 |
| GOTERM_MF_DIRECT | GO:0042043~neurexin family protein binding | CPE, NLGN4Y, NLGN1, SYTL2 | 3.914888683 | 72.96228421 |
| GOTERM_MF_DIRECT | GO:0044183~protein binding involved in protein folding | CLGN, HSPA1A, HSPA1B, CD74 | 3.914888683 | 72.96228421 |
| GOTERM_MF_DIRECT | GO:0034185~apolipoprotein binding | LPL, MAPT, PCSK9, SCARB1 | 3.914888683 | 72.96228421 |
| GOTERM_MF_DIRECT | GO:0000989~transcription factor activity, transcription factor binding | TP63, FOXO1, LDB2, HMGA2 | 3.914888683 | 72.96228421 |
| GOTERM_MF_DIRECT | GO:0005096~GTPase activator activity | RABGAP1, PREX2, ARHGAP17, ADAP1, ALDH1A1, DOCK2, ARHGAP6, AGAP11, TIAM2, ARHGAP44, STARD8, CDC42EP3, ARHGDIB, ALS2CL, VAV3, SIPA1L2, RGS17, RGS16, RGS20, SYDE2, RGS2, ARRB1, RGS3, SIPA1L1, RGS4, RIN2, RGS7, KALRN | 1.37512219 | 74.59731908 |
| GOTERM_MF_DIRECT | GO:0030215~semaphorin receptor binding | SEMA4G, SEMA3G, SEMA7A, SEMA3C, SEMA4D | 2.97871965 | 75.36320062 |
| GOTERM_MF_DIRECT | GO:0008289~lipid binding | PTGS2, BPIFB4, ARHGEF5, APOLD1, STARD10, SEC14L4, S1PR3, CD36, APOL1, STARD4, APOD, FNBP1L, APOE, STARD8, FABP3, APOL4, FABP5 | 1.542621699 | 76.23870358 |
| GOTERM_MF_DIRECT | GO:0015204~urea transmembrane transporter activity | AQP9, AQP7, AQP3 | 5.872333024 | 77.51890443 |
| GOTERM_MF_DIRECT | GO:0016004~phospholipase activator activity | ARHGAP6, CCL8, CCL5 | 5.872333024 | 77.51890443 |
| GOTERM_MF_DIRECT | GO:0008603~cAMP-dependent protein kinase regulator activity | PRKAR1B, PRKAG2, CXCL10 | 5.872333024 | 77.51890443 |
| GOTERM_MF_DIRECT | GO:0005085~guanyl-nucleotide exchange factor activity | ARHGEF4, RALGPS2, VAV3, ARHGEF5, DOCK5, MCF2L, DOCK2, RASGRF2, TIAM2, RAPGEF5, RAPGEF3, FGD3, FGD4, KALRN | 1.625674114 | 78.01554251 |
| GOTERM_MF_DIRECT | GO:0001046~core promoter sequence-specific DNA binding | MEOX1, PPARG, ESR1, SOX4, RORA, NR4A3, ZFHX3 | 2.23057611 | 78.62341025 |
| GOTERM_MF_DIRECT | GO:0070402~NADPH binding | HMGCR, KCNAB1, FASN, SRD5A1 | 3.653896104 | 79.01939431 |
| GOTERM_MF_DIRECT | GO:0030552~cAMP binding | HCN2, BVES, PRKAR1B, POPDC3, RAPGEF3 | 2.854606331 | 79.77152219 |
| GOTERM_MF_DIRECT | GO:0030165~PDZ domain binding | F11R, ACVR2A, ATP2B4, CADM1, GRIK2, SLC9A3, SLC22A4, SNTB1, NLGN1, GJA1, CRIM1 | 1.752595515 | 80.50111649 |
| GOTERM_MF_DIRECT | GO:0042626~ATPase activity, coupled to transmembrane movement of substances | ABCA8, ABCA7, ABCA10, TAP1, ABCA3, ABCA5, ABCB4 | 2.179881198 | 81.54225639 |
| GOTERM_MF_DIRECT | GO:0008270~zinc ion binding | LMO2, LMO4, SNCA, PPARG, UTRN, PDLIM4, MMP27, PDLIM1, RORB, RORA, ENPEP, MMP2, MMP1, MMP24, PGR, GATA2, ZNF184, PAPPA, SMPDL3A, TRIM9, TRIM7, ERAP2, MICAL1, NEURL1B, KDM5D, EGR1, PRKCA, CECR1, ESR1, RNF175, ZSWIM4, MMP11, MMP10, ADAMTS6, ADAMTS8, CPE, LIMCH1, ADAMTS1, TNFAIP3, ABLIM1, IFIH1, ADAMTSL1, LIMS2, ENPP1, CA13, ADAMTS15, NANOS1, NR3C2, OAS1, OAS2, LIN28A, GCH1, CPZ, TK1, ACE, PEG10, RNF166, MT1E, ADAMTS10, TCEA1, CD4, XAF1, UNKL, MT1F, DTX4, CPA4, RNF144B, CRIP1, MT1M, TRHDE, MEX3B, NR4A2, BIRC5, PAPLN, RNF207, NR4A3, BIRC3, MARCH4, MT1X, SHANK3, MID2, RNF112, AJUBA, DDX58, QPCT, VAT1L, PRICKLE4, RNF150, PRICKLE1, DRP2, MT2A, RNF157, ZNF385D, SH3RF2, PAPPA2, PHEX, ZFHX3, ADAMDEC1 | 1.148679913 | 81.56923345 |

1. GO_CellularComponent

| Category | Term | Genes | Fold Enrichment | FDR |
| --- | --- | --- | --- | --- |
| GOTERM_CC_DIRECT | GO:0005886~plasma membrane | SLC9A3, L1CAM, GRIN3B, GRIN2D, SERPINE1, FAS, CDH24, CDH23, GNG7, PTPRJ, PTPRF, ROCK2, PDPN, ZHX2, LIFR, COLEC12, MARK1, PDCD1LG2, F3, ROR2, HLA-DPA1, HLA-DRA, HSD17B10, RALGPS2, CYP51A1, GNAI1, PCDH20, HMGCS1, NFKBIA, CACNB2, CACNB4, ADAP1, SLC29A1, SLC29A2, RAC2, CDC42EP3, IL1RN, GAS1, PCDH17, PENK, RGS2, RGS3, RGS4, AVPR1A, RGS7, SYTL2, LDLR, KCNAB1, TRPV2, MAPT, SLC22A4, TRPV4, TMEM102, GUCY1A3, ANGPT1, TMEM100, ANGPT2, KCNG1, FMNL1, STS, CACNG8, CMKLR1, CACNG7, SDK2, ARHGEF5, SLC3A2, SDK1, RFTN2, FLNC, FLNB, MYADM, FMN2, NAALAD2, EPB41L1, PLXDC1, CLIC6, SORT1, GRIP2, RASD1, ALPL, C3, PCDHB14, GPR68, C2ORF88, DISP2, ITM2C, PCSK9, HSD17B7, PRSS12, HSD17B8, SLC8A1, OPRL1, IGF1, CAPN2, RASSF3, LSP1, CDH13, FAM155A, GFRA1, TAPBPL, PERP, PHLDB2, GFRA2, HTR2A, MICA, IL16, GRIK2, TGFB3, JAG2, RRAD, ENPEP, SLC7A5, IL17RB, SEZ6L2, SLC2A5, UNC5B, SLC2A1, TGFBI, IL15RA, MTUS1, FLVCR1, KCND3, BST2, BST1, HLA-A, PDXP, PSD4, SIX2, HLA-C, HLA-B, NTSR1, HLA-F, NCAM1, NCAM2, RASGRF2, ARRB1, PTGFRN, SLC40A1, ABCA8, ABCA7, IFITM1, TNFRSF12A, KCNA3, ABCA3, P2RY1, ADRA2A, TMEM30B, HCN2, MSMO1, VAV3, CUBN, ADAM23, MET, DOCK5, ANKH, SHANK3, ABCB4, THSD7A, SLC7A2, PRKAR1B, CACNA1G, SYT14, RHBDL2, SLC13A3, ADAM22, SCN8A, ADRA1D, MYH10, RHOJ, CLDN7, LRRC8B, LRRC8C, LEPR, PREX2, DUOX1, RHOQ, MFSD2A, SKAP2, GPC4, SLC1A4, SPRY1, PCDH1, SLC1A3, RASL10B, ANO4, AHNAK, SLC43A2, PTGER2, PTGER3, ESR1, PCDH7, ULBP3, TNFSF13B, CHRM2, ULBP1, CNTN1, JAM2, PMEPA1, GAP43, LCP1, VIM, CD109, CDH2, VCAM1, RGMA, CD4, NFATC2, SECTM1, FLT1, TSPAN13, ANXA3, AJUBA, DENND4C, ADCY4, SYT1, ATP1B1, CADM1, AQP9, UTRN, SNCA, GDF5, LPAR4, AQP7, GGT1, SYT7, AQP1, ADORA1, AQP3, S1PR3, ATP2B4, PRRT2, APOE, CD46, SEMA7A, DYNC2H1, ERAP2, RAPGEF3, SLC12A7, F11R, F10, EFNB3, TRPA1, APOLD1, KRT19, CD36, CD34, HTR7, PLA2G2A, BTN3A2, ENPP5, KCNMB4, ENPP1, GSDMB, ENPP4, ITGA11, OAS3, AKAP12, BDKRB1, NRN1, CD74, EPHB6, ACE, RHBDF2, TRPC4, S100A16, IL1RL1, CFB, CELSR3, GPR153, NOTCH3, GGT5, SFRP1, KIAA0922, CD58, HEPH, GRK5, CLTCL1, MCHR1, KCNJ15, NRP1, GJA1, TAS1R3, SLC26A1, HVCN1, MCF2L, KCNIP3, EDNRB, GPR173, ICAM1, ADAM11, RXFP1, PCDHB5, ICAM5, NFAM1, ARRDC3, SLIT2, AMIGO2, TNFRSF10C, SEMA4G, IL20RB, SGCD, SEMA4D, ADAM12, SLC39A14, RAB3B, LIMS2, GPM6B, SLC19A3, ZBTB16, CLDN11, GJC1, FAT3, PLIN4, FASN, APBA2, ETV5, PLEC, CAP2, BAIAP2L1, NLGN1, NPR1, LY6K, GRIA4, NPR3, GJB2, AGTRAP, FZD6, DKK1, CDON, DRP2, PTCH1, TJP2, PLXNA4, GABRB3, TLR4, MMP2, IL31RA, DYSF, MCOLN3, SLC4A4, SLC4A3, SCAMP5, SLCO4A1, PLXNB3, CPE, FNBP1L, LSAMP, LRP11, IGSF3, RIPK3, DSP, CLIP3, WNT5A, FRAS1, SLC38A5, ADCYAP1R1, ITGB5, PAQR8, MRAP2, FXYD6, CSMD2, KCNS2, PLCH1, RHOBTB1, SCARB1, TMEM97, LPL, PODXL, CPNE7, ITGA2, ITGA4, RAB33A, LIN7A, DIO2, ITGA6, PHEX, IFI6, SLC5A3, BCAM, ARHGAP17, KCNQ3, CXCR4, SV2A, SHC3, IFNGR2, SHC2, KIRREL3, EFR3B, PRKCA, COL23A1, NRXN2, PCDHGB6, PRKD1, ACVR2A, ACVR2B, BVES, PCYOX1, ICOSLG, TRIB3, CYTH3, GPRC5A, TNFRSF1B, PLCD3, SLC39A8, PALM2, ZC3H12A, MYOF, CAMK2A, CRIM1, PTPRC, PTPRD, IL2RB, LTK, RGS16, UBL3, SLC17A7, KCNN4, RGS20, TMEM47, SLC6A9, SULF1, NTM, MGST2, ATP8A1 | 1.419397811 | 3.22E-13 |
| GOTERM_CC_DIRECT | GO:0005615~extracellular space | CTHRC1, LYPD3, MICA, IL16, MASP1, PGF, EDN1, GDF5, SNCA, FSTL3, TGFB3, GGT1, POSTN, MMP2, IL11, CXCL10, OGN, SERPINE2, APOD, PAPPA, CDNF, APOE, SEMA7A, SERPINE1, TGFBI, CFH, IL15RA, COL12A1, LGI4, LOX, CFD, MTUS1, ADAM9, SPON1, INA, STC2, CECR1, KRT10, TNFAIP6, CD36, CPE, F3, PLA2G2A, STC1, LAMC1, CSTA, SSC5D, TNFAIP2, WNT5A, PODN, CCL2, ENPP1, MST1, OAS3, CCL8, IL32, IL33, IL34, CCL5, NRN1, TIMP3, ABCA3, ABI3BP, CCL7, TIMP1, ACE, ANGPTL1, GCNT1, SRGN, OLFM1, CPA4, LPL, CFB, PODXL, IL1RN, CHI3L1, PPFIBP2, SERPINI1, ECM2, LGALS9, TNFSF10, OMD, COL14A1, APOL1, SFRP1, NLGN4Y, LAMA5, SFRP4, FABP3, PCSK1N, APOL4, BMP6, PRKAG3, NAMPT, NRP1, PRKAG2, IGFBP6, PRDX4, MCF2L, GPC4, MTHFD2, SEMA3G, SMPDL3A, GPX3, SEMA3C, LOXL4, ANGPT1, LOXL3, VWA1, APLN, ANGPT2, IL13RA2, ICAM1, EGFL6, ELANE, CILP, CTSS, SLIT2, INHBB, RAB11FIP4, MMP10, SEMA4G, EREG, TNFSF13B, PLXDC1, CFL2, SERPINB7, SEMA4D, CTSH, LCP1, ALPL, CSF3, CXCL1, FMOD, C3, TNC, CXCL3, CTF1, NDP, CXCL2, CRLF1, ADAMTS15, CD109, DCN, CXCL6, CPZ, VCAM1, PCSK1, C1QTNF4, COMP, C1QTNF1, PTN, PCSK9, C2, THBS1, THBS4, DPT, SECTM1, IL6, FLT1, IL7, COL15A1, NLGN1, IGF1, DPYSL3, DKK2, ELFN2, CDH13, DKK3, DKK1, CLEC3B, AKR1B1, SULF1, GDF10, CP, GDF15, IGFBP2, IGFBP4 | 1.823027818 | 2.10E-12 |
| GOTERM_CC_DIRECT | GO:0005578~proteinaceous extracellular matrix | ASPN, CTHRC1, COL21A1, POSTN, MMP2, MMP1, MMP24, GPC4, OGN, CILP2, EMID1, TGFBI, LOX, COL11A2, SPON1, COL10A1, MATN3, OLFML2B, CILP, OLFML2A, MGP, SLIT2, MMP11, MMP10, ADAMTS6, ADAMTS8, BGN, LAMC3, ADAMTS1, ALPL, WNT5A, FMOD, PODN, ADAMTSL1, SPOCK2, ADAMTS15, ELN, VIT, TIMP3, TIMP1, CPZ, COL9A2, COL9A3, COL6A6, COMP, ADAMTS10, FBN2, COL8A2, DPT, COL4A4, COL4A3, PTPRZ1, IL1RL1, CHI3L1, COL15A1, PAPLN, ECM2, OMD, COL14A1, SFRP1, FBLN2 | 3.14004542 | 2.91E-12 |
| GOTERM_CC_DIRECT | GO:0031012~extracellular matrix | ASPN, RARRES2, COL21A1, MMP27, TGFB3, POSTN, EDIL3, MMP2, MMP1, MMP24, OGN, SERPINE2, APOE, SBSN, SERPINE1, TGFBI, COL12A1, SPON1, ADAM11, CILP, MGP, FLNB, MMP11, MMP10, ADAMTS8, BGN, F3, DSP, LAMC1, CSTA, SSC5D, FRAS1, FMOD, ADAMTSL4, NDP, TNC, VIM, DCN, TIMP3, ABI3BP, COMP, ADAMTS10, FBN2, THBS1, COL8A2, DPT, THBS4, PLEC, LPL, NES, COL15A1, COL14A1, SFRP1, LAMA5, FBLN2, CLEC3B, CDON, THSD4 | 2.703193731 | 1.16E-08 |
| GOTERM_CC_DIRECT | GO:0009986~cell surface | MICA, NRP1, LDLR, TRPV2, SLC9A3, TGFB3, TLR3, L1CAM, TLR4, IL17RB, SLC1A4, KCNQ3, SLC1A3, CXCR4, CD46, TRPV4, TMEM102, ROBO2, FAS, ADAM9, PTPRJ, ICAM1, SCUBE3, BST2, PLXNB3, ELANE, HLA-A, SLC3A2, HLA-C, HLA-B, NFAM1, NTSR1, SLIT2, HLA-F, NCAM1, ACVR2A, BGN, CD36, F3, BACE2, IGSF3, SORT1, HLA-DPA1, PTGFRN, HLA-DRA, WNT5A, DCBLD2, ABCA7, PPFIA4, ENPP1, RTN4RL1, TNFRSF12A, ADCYAP1R1, NDP, ADAMTS15, CD109, ITGB5, CD74, VCAM1, RGMA, EPHB6, HSPA2, P2RY1, PCSK9, PTN, SCARB1, CD24, THBS1, PTPRC, LPL, TRPC4, MET, NLGN1, LY6K, ITGA2, ITGA4, FZD6, NLGN4Y, SFRP1, ITGA6, LAYN, CD58, SULF1, SFRP4, AMOT, SCARA5 | 2.188971231 | 1.26E-08 |
| GOTERM_CC_DIRECT | GO:0005576~extracellular region | RARRES2, IL16, MASP1, TUFT1, MASP2, PGF, C9ORF47, F13A1, EDN1, GDF5, SNCA, FSTL3, TGFB3, FGF11, MMP2, MMP1, IL11, CXCL10, IL17RB, OLFML1, OGN, BDNF, SERPINE2, ISG15, APOD, PAPPA, APOE, SERPINE1, TGFBI, CFH, COL12A1, LGI4, LGI2, LOX, HTRA3, CFD, COL10A1, TMEFF2, MATN3, F10, STC2, SCUBE3, OLFML2B, CA11, APOLD1, CECR1, HLA-C, VASH2, LAMC3, PDGFRL, PLA2G2A, LAMC1, WNT5A, ENPP5, CCL2, BPIFB4, ELN, MST1, OAS1, IL33, IL34, CCL5, MDK, TIMP3, CCL7, TIMP1, FAM19A5, EPHB6, ACE, HEG1, LFNG, SRGN, LPL, NPB, OVGP1, PRSS53, ADAM23, CFB, MET, NOTCH3, HYOU1, TNFSF10, PRADC1, OMD, LAMA3, COL14A1, APOL1, SFRP1, PENK, LAMA5, NPW, SFRP4, ADM2, PAPPA2, C11ORF45, APOL4, BMP6, NXPH4, COL21A1, FAM3C, LEPR, IGFBP6, IL4I1, RSPO4, DMKN, RSPO3, GPX3, ITIH5, ANGPT1, LOXL3, GPX7, COL11A2, APLN, GFOD1, ANGPT2, IL13RA2, KIRREL3, PCYOX1L, PRG4, ELANE, CTSS, PSG1, SLIT2, MMP11, INHBB, MMP10, INHBA, BGN, EREG, TNFSF13B, PLXDC1, ADAM12, CSF3, CXCL1, FMOD, EPDR1, C3, TNC, CXCL3, CTF1, CXCL2, CRLF1, NMB, TMEM155, DCN, CXCL6, ISM1, IGSF10, PRRG3, COL9A2, TNFRSF1B, COL9A3, COL6A6, FNDC1, COMP, C1QTNF1, C2, FBN2, FIBIN, THBS1, COL8A2, CRIM1, THBS4, COL4A4, COL4A3, IL6, DHRS13, IL7, COL15A1, LY6K, IGF1, PLAC9, DKK2, DKK3, DKK1, FBLN2, CLEC3B, VSTM2L, CP, MEGF6, GDF15, IGFBP2, ADAMDEC1, IGFBP4, IGFBP5 | 1.585209774 | 1.95E-07 |
| GOTERM_CC_DIRECT | GO:0005887~integral component of plasma membrane | PLXNA4, MICA, CADM1, GABRB3, AQP9, GRIK2, LPAR4, JAG2, TLR3, TLR4, AQP7, ENPEP, AQP1, SLC7A5, ADORA1, AQP3, IL17RB, S1PR3, ATP2B4, SLC2A5, CD46, SLC2A1, FAS, SLC4A4, SLC4A3, FLVCR1, PTPRJ, SLC12A7, EFNB3, PTPRF, BST2, PDPN, SLCO4A1, TRPA1, PLXNB3, HLA-A, LIFR, HLA-C, HLA-B, NTSR1, TMEM130, PODXL2, CD36, RELT, CD34, HTR7, ROR2, HLA-DPA1, SLC40A1, HLA-DRA, DCBLD2, SLC38A5, KCNMB4, ENPP1, ADCYAP1R1, CACNB2, BDKRB1, ALCAM, SLC29A1, EPHB6, SLC29A2, LAPTM5, P2RY1, SCN9A, ADRA2A, SCARB1, HCN2, TRPC4, ADAM23, PODXL, MET, ANKH, ABCB4, TNFSF10, NLGN4Y, ATP2A3, SLC7A2, CD58, NTRK2, AVPR1A, SLC13A3, PHEX, SCARA5, CLCN4, ADRA1D, SLC5A3, MCHR1, KCNJ15, LDLR, TRPV2, LRRC8C, GJA1, MFSD2A, BCAM, HVCN1, SLC26A1, MMP24, TSPAN11, SLC1A4, GPC4, EDNRB, PCDH1, SLC22A4, TRPV4, EVI2B, IFNGR2, SLC43A2, ICAM1, PTGER2, PTGER3, ICAM5, CMKLR1, PCDH7, SLC7A14, PRKD1, ACVR2A, TNFRSF10C, ACVR2B, EREG, CHRM2, SEMA4D, TM4SF1, JAM2, SLC39A14, MFSD3, SLC19A3, GPR68, GPRC5A, TNFRSF1B, C1QTNF1, TAP1, SLC39A8, DCLK1, PTPRC, PTPRD, SLC8A1, IL2RB, TRHDE, LTK, FLT1, PTPRZ1, OPRL1, NLGN1, FADS2, NPR1, TSPAN13, NPR3, TSPAN18, FZD6, SLC16A5, SLC6A9, SLC16A4, CDON, PERP, HTR2A | 1.511179827 | 1.90E-04 |
| GOTERM_CC_DIRECT | GO:0070062~extracellular exosome | SNCG, ATP1B1, RARRES2, RARRES1, CADM1, FAM20A, MASP2, SLC9A3, UTRN, PGAM2, GGT1, SYT7, EDIL3, AQP1, OGN, APOD, APOE, CD46, PGAM4, SBSN, SERPINE1, DYNC2H1, CFH, COL12A1, FAS, RAPGEF3, PI16, RAB27B, CFD, GNG7, ADAM9, PTPRJ, F11R, TNIK, PTPRF, LIFR, TMEM132A, KRT10, COLEC12, PDCD1LG2, PGM2, KRT19, F3, PLA2G2A, LAMC1, HLA-DRA, RBP5, GNAI1, ENPP4, MST1, ACP5, RIMS2, ACAT2, CD74, ALCAM, SERINC2, ACE, RAC2, IDH1, FGL2, PABPC1, ST6GAL1, S100A16, CFB, MAOB, IL1RN, DUSP23, ACACA, CHI3L1, SERPINI1, LGALS9, HYOU1, LAMA3, COL14A1, SFRP1, LAMA5, CD58, QPRT, PAPPA2, CLTCL1, CRYM, FAM3C, LXN, GJA1, LRRC15, MMP24, MSRA, CILP2, SEMA3G, SMPDL3A, DMKN, GPX3, SEMA3C, ANGPT1, VWA1, FMNL1, ICAM1, ELANE, SDK1, SLC3A2, FLNB, MYADM, SLIT2, BGN, CLIC6, PLXDC2, HAO2, ALPL, RAB3B, EPDR1, C3, CLDN11, ITM2C, FASN, C2, THBS1, PLEC, THBS4, MARS, SHMT1, BCAS1, SHMT2, TRHDE, BAIAP2L1, COL15A1, NPR3, PCK2, CAPN2, MXRA5, MID2, SOD2, QPCT, LSP1, CDH13, FBLN2, CLEC3B, CAPG, AOX1, PHGDH, GFRA1, CP, PSAT1, ENPEP, SLC7A5, LOC81691, GSTM2, GSTM3, DYSF, SLC2A5, TIAM2, TGFBI, SLC2A1, ROBO4, ROBO2, SLC4A4, TMEM205, BST2, BST1, NCALD, PDXP, HLA-A, MGP, HLA-C, HLA-B, NCAM1, RENBP, RELT, CPE, FNBP1L, MARC2, DSP, MVK, CSTA, TNFAIP3, KALRN, WNT5A, PPIL1, ITGB5, TIMP3, TIMP1, GMPPB, DOCK2, SCARB1, ANGPTL1, MEST, ARHGDIB, LPL, CUBN, VAV3, PODXL, CPNE7, ENDOD1, ITGA4, LIN7A, PSMB9, ABCB4, THSD7A, TNFSF10, OMD, PRADC1, DNAJB9, THSD4, FABP3, SLC13A3, PCSK1N, FABP5, MYH10, RHOJ, NAMPT, MLPH, IGFBP6, PRDX4, RHOQ, BCAM, AKR1C3, SLC1A4, GPC4, WARS, DES, PCBP3, CXCR4, SH3D21, LOXL4, AHNAK, PRKCA, CDK1, CILP, EML5, PSME2, CFL2, CNTN1, TXNRD1, PCYOX1, CTSH, LCP1, ICOSLG, RTN4RL1, VIM, CDH2, GPRC5A, CPZ, ALDH1A1, VCAM1, RGMA, HSPA2, ALDH1A3, COMP, KRT8, MYOF, CRIM1, DPT, PTPRC, SECTM1, PTPRD, PM20D2, GDPD3, UBL3, FUCA1, ANXA3, H2AFY2, AKR1B1, GDF15, IGFBP2, ATP8A1 | 1.295638875 | 0.004419882 |
| GOTERM_CC_DIRECT | GO:0005604~basement membrane | FRAS1, COL4A3, EGFL6, TNC, CCDC80, TIMP3, TIMP1, LAMA3, ITGA6, LAMC3, LAMA5, FREM1, TGFBI, PTN, ADAMTS1, LAMC1, VWA1, COL8A2, THBS4 | 3.317931372 | 0.015336883 |
| GOTERM_CC_DIRECT | GO:0005614~interstitial matrix | KAZALD1, TNC, ADAMTSL4, CCDC80, ECM2, VWA1, VIT, ABI3BP | 8.489605777 | 0.017399671 |
| GOTERM_CC_DIRECT | GO:0005581~collagen trimer | COL4A4, COL4A3, CTHRC1, COL23A1, COL21A1, COL15A1, COLEC12, MMP1, TIMP1, COL9A2, COL14A1, CD36, COL6A6, EMID1, C1QTNF1, COL12A1, LOX, COL11A2, COL8A2, COL10A1 | 2.999045519 | 0.039122563 |
| GOTERM_CC_DIRECT | GO:0042383~sarcolemma | KCND3, ATP1B1, SLC8A1, UTRN, FLNC, AQP1, VCAM1, KRT19, BGN, BVES, DYSF, DES, SLC2A5, KRT8, SNTB1, SGCD, CIB2, AHNAK, PLEC | 3.083724451 | 0.044362766 |
| GOTERM_CC_DIRECT | GO:0009897~external side of plasma membrane | RTN4RL1, LDLR, ASTN1, BCAM, TLR4, ENPEP, CD74, IL31RA, CXCL10, VCAM1, ALCAM, GPC4, ACE, SEMA7A, HEG1, CD4, FAS, THBS1, ICAM1, PTPRC, IL2RB, IL6, PDPN, IL1RL1, NLGN1, ITGA2, NCAM1, CDH13, CD36, ITGA6, CD34, AMOT | 2.072579814 | 0.222749735 |
| GOTERM_CC_DIRECT | GO:0045121~membrane raft | RTN4RL1, GNAI1, RHOQ, KCNA3, GJA1, GPM6B, EDNRB, TNFRSF1B, UNC5B, SDPR, ANGPT1, CD4, FAS, CD24, AHNAK, ICAM1, PTPRC, BST2, PODXL, RFTN2, NFAM1, CAPN2, BIRC3, NTSR1, MYADM, ABCB4, CD36, SULF1, CNTN1, CLIP3 | 2.009069328 | 0.629739055 |
| GOTERM_CC_DIRECT | GO:0016021~integral component of membrane | PEAR1, SLC9A3, STOML1, L1CAM, GRIN3B, GRIN2D, CH25H, FAS, CDH24, ADAM9, CDH23, PTPRJ, TMEFF2, PTPRF, PDPN, LIFR, TMEM132A, CCDC136, COLEC12, TMEM130, PDCD1LG2, PTPRQ, F3, BACE2, HSD11B1, HSD11B2, ROR2, HLA-DPA1, ADAMTS1, HLA-DRA, RTP4, MGAT5B, CYP51A1, PCDH20, ACP5, CCDC167, SORCS2, FAM19A5, ALCAM, SYBU, NDUFA4L2, MAOA, MAOB, CCDC80, GAS1, PCDH17, CYP7B1, CLGN, APOL1, APCDD1L, AVPR1A, APOL4, LDLR, KCNAB1, TRPV2, LRRC15, ST6GALNAC5, SLC22A4, TRPV4, TMEM102, GUCY1A3, TMEM100, IL13RA2, FMNL1, STS, CMKLR1, CLMN, CACNG7, SDK2, SLC3A2, SDK1, RNF175, FLNB, MYADM, NAALAD2, SQLE, TLCD2, PLXDC1, PLXDC2, SORT1, ALPL, PCDHB14, DISP2, GPR68, ITM2C, DHCR7, ACSL3, GALNT12, HSD17B7, SLC8A1, ABCA10, OPRL1, FADS1, COL15A1, FADS2, MARCH4, FAM155A, AMOT, TAPBPL, PHLDB2, MICA, GRIK2, HMGCR, ENPEP, SLC7A5, FDFT1, IL17RB, SEZ6L2, SLC2A5, UNC5B, SLC2A1, IL15RA, ELOVL6, FLVCR1, TMEM205, BST2, SPTLC3, HLA-A, HLA-C, HLA-B, PNPLA3, HLA-F, NCAM1, NCAM2, RELT, MARC2, PTGFRN, SLC40A1, NETO2, ABCA8, ABCA7, MAMDC4, IFITM1, TNFRSF12A, ASTN1, ST8SIA1, LRIG1, IL33, ABCA3, ABCA5, PRUNE2, ECE2, TMEM30B, B4GALT6, MSMO1, CCPG1, ADAM23, MET, FDPS, ANKH, ABCB4, THSD7A, TNFSF10, RNF150, FREM1, CACNA1G, SYT14, RHBDL2, SLC13A3, ADAM22, SCN8A, CEND1, ADRA1D, CLDN7, IER3, LRRC8B, CLDN6, LRRC8C, LEPR, DUOX1, MFSD2A, SYNGR1, TSPAN11, SLC1A4, FRMD3, PCDH1, SLC1A3, DSEL, ANO4, SLC43A2, PTGER2, PTGER3, ESR1, SRPRB, PCDH7, DHRS3, TNFSF13B, DPY19L2P2, TM4SF1, JAM2, PMEPA1, PKDREJ, HS3ST6, GALNT6, CDH2, VCAM1, PRRG3, CD4, B4GALNT4, FBN2, TYW1B, NOX4, SECTM1, RNF144B, TSPAN13, GDPD3, TSPAN18, RCAN2, RNF112, CYP3A5, ADCY4, SYT1, RARRES1, LYPD3, AQP9, CADM1, B3GALT4, LPAR4, AQP7, GGT1, SYT7, AQP1, ADORA1, AQP3, BDNF, ATP2B4, PRRT2, CD46, INSIG1, ERAP2, PI16, F11R, TRPA1, APOLD1, CD36, CD34, FER1L4, FER1L6, ASPHD1, BTN3A2, ENPP5, ERMP1, GCNT4, ENPP1, ENPP4, OAS3, BDKRB1, TMCC3, CD74, SFXN5, SERINC2, EPHB6, ACE, GCNT1, RHBDF2, TRPC4, ST6GAL1, IL1RL1, SCD, CELSR3, GPR153, NOTCH3, GGT5, IFI27, C1ORF115, SFRP1, ATP2A3, KIAA0922, CD58, SFRP4, NTRK2, HEPH, CLCN4, MCHR1, KCNJ15, NRP1, SUSD1, TAS1R3, SLC26A1, HVCN1, EDNRB, DHCR24, GPR173, ICAM1, ADAM11, KIAA1324L, RXFP1, PCDHB5, ICAM5, CYYR1, NFAM1, AMIGO2, TNFRSF10C, ANKRD29, SEMA4G, IL20RB, SGCD, SUSD5, ADAM19, ADAM12, SLC39A14, GPM6B, SLC19A3, SEC14L4, CLDN11, GJC1, FAT3, XBP1, POPDC3, PTPRZ1, LRRN3, NPR1, GRIA4, NPR3, GJB2, AGTRAP, FZD6, FAM180B, SLC16A5, SAMD5, SLC16A4, LAYN, MTFP1, PTCH1, PTCH2, SLC15A3, GREB1L, TMEM176B, TMEM176A, PLXNA4, GABRB3, FAM189B, TLR3, IL31RA, DYSF, MCOLN3, PAPPA, C2ORF74, ROBO4, ROBO2, SLC4A4, SLC4A3, SCAMP5, SLCO4A1, PLXNB3, FIBCD1, MOXD1, SLITRK4, KCNT2, LSAMP, LRP11, IGSF3, FRAS1, WNT5A, SLC38A5, ADCYAP1R1, PAQR8, MRAP2, FXYD6, CSMD2, PAQR4, KCNS2, B3GNT5, ENTPD7, HEG1, SCARB1, MEST, TMEM97, ADSSL1, TP53I11, UST, PODXL, TMEM91, ITGA4, DIO2, ITGA6, UCP2, PHEX, IFI6, SLC5A3, UTY, MRVI1, BCAM, MCTP2, EVI2A, CXCR4, NIPAL3, SRD5A1, EVI2B, SV2A, IFNGR2, C11ORF87, KIRREL3, VSIG10, COL23A1, NRXN2, PCDHGB6, SEL1L3, MCTP1, CHST1, ACVR2B, BVES, TMEM182, ICOSLG, TMEM158, GPRC5A, TNFRSF1B, TMEM154, TAP1, SLC39A8, HS6ST3, MYOF, CRIM1, ZNF804A, GIMAP2, PTPRC, FAM174B, PTPRD, LTK, TMEM51, SLC17A7, FRRS1, ELFN2, KCNN4, TMEM47, ITPRIPL1, MGST2, ATP8A1 | 1.143622084 | 0.726599129 |
| GOTERM_CC_DIRECT | GO:0016323~basolateral plasma membrane | CLDN7, ATP1B1, AQP9, LDLR, ENPP1, CADM1, LEPR, CDH2, AQP1, ADORA1, AQP3, SLC29A1, ATP2B4, SLC29A2, CD46, SLC2A1, P2RY1, ADRA2A, SLC4A4, ADAM9, TRPC4, CA11, LIN7A, ARRB1, DSP, HEPH, SLC40A1 | 2.069341408 | 0.820005715 |
| GOTERM_CC_DIRECT | GO:0005901~caveola | ATP1B1, TRPC4, PTGS2, HCK, ADCYAP1R1, CDH13, BVES, ATP2B4, SDPR, SLC2A1, SCARB1, PTCH1, MYOF, HTR2A | 2.971362022 | 0.974430775 |
| GOTERM_CC_DIRECT | GO:0005788~endoplasmic reticulum lumen | WNT5A, ADAMTSL1, PTGS2, COL21A1, ADAMTSL4, COL9A2, COL9A3, COL12A1, CD4, ERAP2, GPX7, COL11A2, THBS1, COL8A2, COL10A1, SPON1, COL4A4, COL4A3, STS, F10, COL23A1, COL15A1, RDH5, HYOU1, COL14A1, DNAJB9, H6PD, SDF2L1 | 2.011859702 | 0.990698785 |
| GOTERM_CC_DIRECT | GO:0031225~anchored component of membrane | ALPL, LPL, BST2, BST1, CD109, LY6K, NRN1, GPC4, NCAM1, RGMA, CDH13, TNFRSF10C, LSAMP, SEMA7A, CD58, CNTN1, GFRA1, GFRA2, NTM | 2.319615738 | 1.789228755 |
| GOTERM_CC_DIRECT | GO:0005925~focal adhesion | LIMS2, NRP1, TNC, VIM, ITGA11, AKAP12, FERMT1, GJA1, ITGB5, HSPA1A, L1CAM, PDLIM1, HSPA1B, CDH2, APBB1IP, ALCAM, RAC2, CD46, STARD8, SNTB1, TRPV4, PABPC1, EHD3, AHNAK, PLEC, ADAM9, NOX4, ICAM1, PTPRC, FLT1, HCK, ITGA2, SYNPO2, ITGA4, NEXN, CAPN2, FLNC, FLNB, AJUBA, CDH13, HYOU1, ITGA6, LAYN, GRK5, PHLDB2, LCP1 | 1.623012869 | 2.008789301 |
| GOTERM_CC_DIRECT | GO:0048471~perinuclear region of cytoplasm | SNCG, MLPH, HIP1R, SNCA, PPARG, MRVI1, TLR4, BDNF, APOD, PKN3, SRD5A1, TMEM100, MX1, APLN, PRKCA, TNIK, STC2, STMN2, HERC5, RAB11FIP4, INHBB, FMN2, INHBA, TNFSF13B, CD34, PLA2G2A, TSTD1, SORT1, GBP4, GBP3, RASD1, RAB3B, CCL2, GALNT6, NFKBIE, NANOS1, IGF2BP1, HSPA1A, OAS2, HSPA1B, ITM2C, ALDH1A2, TNFRSF1B, KCNS2, MEIS2, MT1E, PCSK9, PTN, ACSL3, EHD3, MT1F, NOX4, MT1M, CHI3L1, ITGA2, CDC20, RNF207, MT1X, CDH13, AKR1B1, MT2A, HEPH, PTCH1, MAP6, PHEX | 1.443984879 | 3.937644628 |
| GOTERM_CC_DIRECT | GO:0030864~cortical actin cytoskeleton | CAP2, SHROOM4, MLPH, UTRN, SLC2A1, TRPV4, RAPGEF3, CDH2, CAPN2, MYADM | 3.208281253 | 4.529380573 |
| GOTERM_CC_DIRECT | GO:0005667~transcription factor complex | LMO2, HOXA11, LMO4, E2F7, ARNT2, TP63, PDLIM1, GATA2, FOS, HAND2, POU5F1, FOXF1, ALX4, PITX1, PITX2, SMAD9, MAFB, MLXIPL, LDB2, NR4A3, AHR, JUNB, AJUBA, SIX1, ZFHX3 | 1.786996035 | 9.214547938 |
| GOTERM_CC_DIRECT | GO:0014069~postsynaptic density | NETO2, CAP2, HIP1R, GRIK2, CACNG8, NLGN1, ADORA1, SHANK3, LIN7A, RGS20, NLGN4Y, ARRB1, DRP2, SIPA1L1, MAPT, P2RY1, NTRK2, GRIP2, PTCH1, CAMK2A, GAP43, DCLK1, KALRN, SYNPO | 1.799427311 | 10.08812022 |
| GOTERM_CC_DIRECT | GO:0005923~bicellular tight junction | CGNL1, CLDN7, F11R, CLDN6, ADCYAP1R1, ARHGAP17, CLDN11, AMOTL2, LIN7A, DDX58, BVES, PLXDC1, WNK4, AMOT, JAM2, TJP2, SYNPO | 2.07544566 | 10.1843705 |
| GOTERM_CC_DIRECT | GO:0015629~actin cytoskeleton | ABLIM1, MTSS1, SHROOM4, SNCA, ANLN, MSRA, ARHGAP6, NFATC2, CDC42EP3, AHNAK, SYNPO, FGD4, BAIAP2L1, PDXP, ACACA, SYNPO2, FLNB, NOTCH3, DDX58, LSP1, FMN2, CFL2, HSPB7, LCP1, MAP7D3, KALRN, MYH10 | 1.70863052 | 11.43354927 |
| GOTERM_CC_DIRECT | GO:0012507~ER to Golgi transport vesicle membrane | SEC31B, CNIH3, HLA-A, HLA-C, HLA-DPA1, HLA-B, CD74, HLA-DRA, HLA-F, SREBF2 | 2.653001805 | 15.58429143 |
| GOTERM_CC_DIRECT | GO:0030027~lamellipodium | ABLIM1, SLC39A14, STMN2, PDPN, PODXL, PDXP, IGF2BP1, DPYSL3, CDH2, APBB1IP, RAB3IP, AJUBA, DYSF, TIAM2, RAC2, AMOT, TRPV4, RAPGEF3, FGD3, FGD4, MYH10 | 1.810673732 | 15.82477224 |
| GOTERM_CC_DIRECT | GO:0030175~filopodium | VCAM1, DNALI1, ITGA6, TIAM2, PDPN, PODXL, UTRN, IGF2BP1, TRPV4, RAPGEF3, LCP1, FGD4 | 2.331652291 | 16.97473622 |
| GOTERM_CC_DIRECT | GO:0046658~anchored component of plasma membrane | LYPD3, RTN4RL1, ULBP3, ULBP1, NTNG1, GAS1, NRN1 | 3.448902347 | 17.94027396 |
| GOTERM_CC_DIRECT | GO:0071556~integral component of lumenal side of endoplasmic reticulum membrane | HLA-A, HLA-C, HLA-DPA1, HLA-B, CD74, HLA-DRA, HLA-F | 3.32997468 | 20.91934795 |
| GOTERM_CC_DIRECT | GO:0030666~endocytic vesicle membrane | WNT5A, CD36, CACNG8, SCARB1, COLEC12, PTCH1, HLA-DPA1, GRIA4, CAMK2A, CD74, HLA-DRA | 2.299268231 | 25.17433782 |
| GOTERM_CC_DIRECT | GO:0043235~receptor complex | NRP1, FLT1, LDLR, ADCYAP1R1, LEPR, NR3C2, LIFR, NPR1, ITGB5, PEX5L, NOTCH3, ACVR2A, ACVR2B, PLXDC1, NTRK2, ADRA2A, LOXL4 | 1.846656375 | 26.70587921 |
| GOTERM_CC_DIRECT | GO:0005764~lysosome | LDLR, EPDR1, SNCA, IL4I1, GJA1, ACP5, SYT7, RRAGD, ITM2C, ABCA5, ACE, LAPTM5, CXCR4, PCSK9, TMEM97, STS, HCK, CTSS, ARRDC3, CAPN2, UNC13D, CD34, PCYOX1, TNFAIP3, CTSH, HLA-DRA | 1.587105505 | 28.36653691 |
| GOTERM_CC_DIRECT | GO:0030018~Z disc | SLC8A1, KY, SYNPO2, NEXN, FLNC, FLNB, MYL9, MURC, KRT19, DES, ATP2B4, CFL2, KRT8, FBXO32, SCN8A, SYNPO | 1.870591103 | 29.01990929 |
| GOTERM_CC_DIRECT | GO:0005911~cell-cell junction | PTPRJ, F11R, TRPC4, CADM1, IQGAP3, AQP7, CDH2, MYADM, AQP3, LIN7A, AJUBA, PRKD1, KIAA1462, TMEM47, PCDH1, EPB41L1, KRT8, SLC2A1, HEG1, SV2A, CDH24 | 1.684347658 | 29.98453187 |
| GOTERM_CC_DIRECT | GO:0016010~dystrophin-associated glycoprotein complex | KRT19, KRT8, UTRN, SNTB1, SGCD | 4.311127933 | 30.45001843 |
| GOTERM_CC_DIRECT | GO:0043198~dendritic shaft | SLC8A1, CNIH3, NLGN1, GRIP2, NTSR1, KIRREL3, HTR2A | 3.017789553 | 31.21694629 |
| GOTERM_CC_DIRECT | GO:1903561~extracellular vesicle | OGN, ATP1B1, SERPINE2, FBLN2, APOE, COL12A1, GRIA4, EDIL3, LFNG | 2.48320969 | 31.99578814 |
| GOTERM_CC_DIRECT | GO:0000942~condensed nuclear chromosome outer kinetochore | PLK1, BUB1, NDC80 | 10.34670704 | 34.3075353 |
| GOTERM_CC_DIRECT | GO:0031233~intrinsic component of external side of plasma membrane | F10, F3, ADAM9 | 10.34670704 | 34.3075353 |
| GOTERM_CC_DIRECT | GO:0032280~symmetric synapse | PENK, CHRM2, NTSR1 | 10.34670704 | 34.3075353 |
| GOTERM_CC_DIRECT | GO:0005916~fascia adherens | DES, GJA1, DSP, CDH2 | 5.518243755 | 36.66782786 |
| GOTERM_CC_DIRECT | GO:0016324~apical plasma membrane | ABCA7, ATP1B1, SHROOM3, SHROOM4, SLC9A3, DUOX1, GJA1, CDH2, ENPEP, AQP1, SLC7A5, AMOTL2, SLC29A1, SLC2A5, P2RY1, SLC2A1, SLC22A4, TRPV4, RAB27B, NOX4, TNIK, CUBN, BST2, PODXL, SLC3A2, FZD6, ABCB4, CD36, CD34, STC1, IGFBP2 | 1.469635364 | 38.10455968 |
| GOTERM_CC_DIRECT | GO:0043195~terminal bouton | SYT1, GRIK2, NTRK2, SNCA, SV2A, SYT7, NTSR1, SYNGR1, ADORA1, PRSS12 | 2.225098288 | 39.18595169 |
| GOTERM_CC_DIRECT | GO:0030054~cell junction | STON2, ABCA7, SYT1, NAMPT, GABRB3, GRIK2, SNCA, UTRN, FERMT1, GRIN3B, SYT7, NRN1, RIMS2, SYNGR1, IL31RA, PRRT2, CXCR4, TRIM9, ARHGAP44, GRIN2D, HOXC5, CAMK2A, OLFM1, SCAMP5, SH3PXD2B, F11R, CACNG8, ARHGEF5, SDK2, NLGN1, SDK1, RGS17, GRIA4, SHANK3, SLC17A7, TMEM47, NLGN4Y, CNIH3, CHRM2, DRP2, SIPA1L1, DSP, PERP, TJP2, GAP43 | 1.352510724 | 40.36674291 |
| GOTERM_CC_DIRECT | GO:0005783~endoplasmic reticulum | PTGS2, FAM20A, HMGCR, ADORA1, KCNIP3, FDFT1, OGN, SEZ6L2, FOS, CERKL, MCTP2, APOD, CDNF, UBXN2B, APOE, EMID1, INSIG1, ERAP2, ELOVL6, GPX7, SV2A, TMEM100, IFNGR2, DHCR24, PRKCA, STS, STC2, HLA-A, TMEM132A, HLA-C, HLA-B, NTSR1, HLA-F, MCTP1, SQLE, BACE2, ULBP1, PLA2G2A, SLC39A14, CYP51A1, RSAD2, OAS1, BDKRB1, MRAP2, OAS2, VCAM1, RGMA, XBP1, DHCR7, PCSK9, PTN, THBS1, FIBIN, ACSL3, HSD17B7, OLFM1, MEST, THBS4, COL4A3, MSMO1, CUBN, SCD, CHI3L1, CAPN2, SREBF2, HYOU1, IFIT2, DNAJB9, CLGN, ATP2A3, KIAA0922, SULF1, PHEX, MGST2, ATP8A1 | 1.2496023 | 40.84606433 |
| GOTERM_CC_DIRECT | GO:0005829~cytosol | MOCOS, STAR, SNCA, PGAM2, SYT7, AMOTL2, TPK1, SERPINE2, ISG15, PGAM4, WNK4, CH25H, DYNC2H1, FAS, ROCK2, NUDT11, MECOM, PGM2, KIF26A, NFKBIE, GSDMB, HMGCS1, OAS3, NFKBIA, ACP5, IGF2BP1, OAS1, HSPA1A, HSPA1B, OAS2, CACNB4, IGF2BP3, PEX5L, TK1, EPHB6, RAC2, IDH1, PABPC1, CDC42EP3, S100A16, SMAD9, DUSP23, ACACA, CDC20, NDC80, NOTCH3, OASL, SFRP1, RGS2, RGS3, RGS4, NTRK2, RGS7, TNK1, QPRT, PLA2G4C, IDI1, CLTCL1, TMOD1, NRP1, KCNAB1, PPARG, FERMT1, TP63, GJA1, MCF2L, GTSE1, KCNIP3, MTHFD2, MSRA, USP18, AP1S2, MAP1LC3A, MAPT, LMOD1, DHCR24, ARHGEF4, FMNL1, FMNL2, PHKG1, HERC6, HERC5, FLNC, FLNB, FMN2, EPB41L1, RRM2, RPS4Y1, GRIP2, SORT1, EIF5A2, NXT1, RAB3B, PPFIA4, RABGAP1, LIMS2, CA13, HK2, STARD10, ZBTB16, APBB1IP, GCH1, XBP1, PDE1A, BUB1, FASN, PLEC, MARS, POLR3G, SHMT1, BAIAP2L1, BIRC5, CAPN2, BIRC3, INMT, MAPK13, MT2A, AOX1, PHGDH, AMOT, PSAT1, TJP2, HTR2A, DUSP6, SNCAIP, IL16, IQGAP3, FOXO1, SLC7A5, GSTM5, GSTM2, EIF4EBP1, GSTM3, CDKN2B, TIAM2, UBXN2B, STARD8, SLC2A1, MX1, MX2, SOCS2, CHAC1, NCALD, RELB, PDXP, MLXIPL, CECR1, DDIT4, RENBP, RASGRF2, ARRB1, PDE5A, RIPK3, MVK, CLIP3, TNFAIP3, NRBP2, KALRN, IFIH1, MVD, IL32, LIN28A, RRAGD, CMPK2, MYL9, DOCK2, ISYNA1, PLCH1, RHOBTB1, FGD3, UNKL, ARHGDIB, FGD4, ADSSL1, OVGP1, CUBN, VAV3, FDPS, SREBF2, PSMB9, DDX58, IKBKE, PRICKLE1, PLK2, PLK1, PRKAR1B, FABP3, FABP5, MYH10, RHOJ, SAT1, PRKAG3, NAMPT, IER3, KYNU, KIF24, PRKAG2, PRDX4, RHOQ, ARHGAP17, SKAP2, PDCD4, RAB3IP, AKR1C3, FOS, KIF2C, WARS, ARHGAP6, TDO2, SPRY1, DES, PCBP3, POU5F1, SDPR, SHC3, SHC2, AHNAK, EFR3B, PRKCA, CDK1, LPIN1, PRKD1, PSME2, TXNRD1, CTSH, LCP1, SAV1, VIM, EGLN3, UPP1, TRIB3, ASNS, CYTH3, ALDH1A1, ALDH1A2, TYMP, HSPA2, ARHGAP44, ALDH1A3, PLCD3, ETNK2, XAF1, NFATC2, PIK3R3, CAMK2A, EHD3, DTX4, RNF144B, GEMIN2, HCK, CENPP, DPYSL4, DPYSL3, GYG2, AJUBA, IFIT2, IFIT1, SYDE2, GCK, IRF7, ZBED3, AKR1B1, IRF1, DENND4C, IRF4, JAK3 | 1.106978008 | 41.82886544 |
| GOTERM_CC_DIRECT | GO:0001725~stress fiber | NOX4, ABLIM1, SHROOM4, AMOT, FLNB, LCP1, SYNPO, MYL9, MYH10 | 2.299268231 | 44.26961998 |
| GOTERM_CC_DIRECT | GO:0042612~MHC class I protein complex | HLA-A, HLA-C, HLA-B, HLA-F | 5.016585232 | 44.99976872 |
| GOTERM_CC_DIRECT | GO:0031594~neuromuscular junction | DES, SERPINE2, UTRN, PTN, POSTN, SV2A, SYNGR1, CIB2, MYH10 | 2.257463354 | 47.47027564 |
| GOTERM_CC_DIRECT | GO:0043034~costamere | KRT19, KRT8, FLNC, AHNAK, PLEC | 3.630423523 | 48.1949241 |
| GOTERM_CC_DIRECT | GO:0005856~cytoskeleton | CMAHP, SHROOM3, HIP1R, VIM, UTRN, AKAP12, PDLIM1, APBB1IP, RAB3IP, TRIB2, FRMD3, DOCK2, TRIM9, SYBU, SNTB1, ZC3H12A, FAM83H, FGD3, PLEC, DHCR24, HIP1, ARHGDIB, TNIK, BAIAP2L1, HCK, PTPN14, PDXP, KY, NEXN, FLNC, MARK1, EPB41L1, FNBP1L, SIPA1L1, DSP, SGCD, JAK3 | 1.375842446 | 49.29769174 |
| GOTERM_CC_DIRECT | GO:0000139~Golgi membrane | B3GALT4, TLR3, GJA1, ST6GALNAC5, AP1S2, SLC2A1, DSEL, IL15RA, IFNGR2, DHCR24, SCAMP5, NMNAT2, GBP5, HLA-A, TMEM132A, HLA-C, RNF175, HLA-B, TMEM130, CHST1, HLA-F, NCAM1, ARRB1, HLA-DPA1, GBP4, GBP3, PMEPA1, HLA-DRA, GCNT4, ABCA7, HS3ST6, MGAT5B, GALNT6, ST8SIA1, CYTH3, CD74, ABCA5, ECE2, B3GNT5, SYBU, B4GALT6, GALNT12, GCNT1, SRGN, ST6GAL1, UST, MARCH4, AGTRAP, SREBF2, NOTCH3, CSGALNACT1, CNIH3, ST8SIA4, TAPBPL, ATP8A1 | 1.283855357 | 49.79542658 |
| GOTERM_CC_DIRECT | GO:0005819~spindle | KIFC1, SNCG, BIRC5, CDC20, RAB11FIP4, FMN2, ANKRD53, PLK1, SEPT6, CLTCL1, MTUS1, MAP7D3, SHCBP1, MYH10, KIF20A | 1.710199511 | 55.96883151 |
| GOTERM_CC_DIRECT | GO:0043197~dendritic spine | SLC8A1, ARHGAP44, HIP1R, ARRB1, SIPA1L1, NLGN1, IGF2BP1, PALMD, NTSR1, ADORA1, SHANK3, MYH10, SYNPO | 1.79342922 | 57.63202712 |
| GOTERM_CC_DIRECT | GO:0043005~neuron projection | STON2, SYT1, CADM1, PTPRF, STMN3, STAR, PTGS2, OPRL1, STMN2, ADCYAP1R1, VIM, BDKRB1, NMB, RGS17, SHANK3, LIN7A, FOS, CDH13, RGMA, ATP2B4, SLC1A3, RGS2, BCL11B, FAS, SV2A | 1.455233058 | 59.43916551 |
| GOTERM_CC_DIRECT | GO:0042613~MHC class II protein complex | HLA-A, HLA-C, HLA-DPA1, CD74, HLA-DRA | 3.13536577 | 65.39670859 |
| GOTERM_CC_DIRECT | GO:0005789~endoplasmic reticulum membrane | CYP3A5, SEC31B, PTGS2, LRRC8B, HMGCR, LRRC8C, MRVI1, GJA1, TLR3, LSS, MFSD2A, FDFT1, SEZ6L2, CH25H, INSIG1, IL15RA, ERAP2, SRD5A1, ELOVL6, MX1, PCYT2, IFNGR2, DHCR24, STS, SPTLC3, TMEM132A, SRPRB, RNF175, LPIN1, PNPLA3, MOXD1, RDH5, FMN2, DHRS3, RASGRF2, SQLE, HSD11B1, PLA2G2A, HSD11B2, SORT1, PTGFRN, EIF5A2, ERMP1, MFSD3, CYP51A1, NR3C2, RSAD2, MRAP2, GJC1, XBP1, DHCR7, CYP26B1, TAP1, CD4, ACSL3, HSD17B7, MEST, NOX4, RHBDF2, MSMO1, FADS1, SCD, FADS2, SREBF2, AGTRAP, NOTCH3, CYP7B1, DNAJB9, CLGN, ATP2A3, CNIH3, SDF2L1, TAPBPL, PLA2G4C, MGST2 | 1.200314042 | 66.10561102 |
| GOTERM_CC_DIRECT | GO:0030659~cytoplasmic vesicle membrane | SNCA, GPRC5A, AGTRAP, FZD6, FMN2, ECE2, AP1S2, DYSF, ARRB1, ULK2, ENTPD7, DENND4C, IL15RA, MYOF, IFNGR2 | 1.642334451 | 66.23288374 |
| GOTERM_CC_DIRECT | GO:0005794~Golgi apparatus | SYT1, LDLR, FAM20A, FAM3C, SNCA, IGFBP6, PPARG, FSTL3, GJA1, KCNIP3, ST6GALNAC5, SPRY1, AP1S2, CERKL, PKN3, UBXN2B, APOE, CD46, EMID1, DYNC2H1, MTUS1, NMNAT2, STS, STC2, BST2, STMN3, STMN2, BEND5, HLA-A, ESR1, TMEM132A, HLA-C, HLA-B, MECOM, NTSR1, GRHL1, PRKD1, CD36, CPE, BACE2, IPO5, TFAP2A, SORT1, ADAM19, PTGFRN, SLC39A14, GLIS3, ABCA7, GALNT6, NFKBIE, ST8SIA1, RSAD2, ITM2C, VCAM1, FASN, PCSK9, FIBIN, ACSL3, FGD3, SRGN, HIP1, FGD4, SECTM1, ST6GAL1, CUBN, HCK, NLGN1, CAPN2, SULF1, PTCH1, MAP6, PHEX, PERP, KIF20A, ATP8A1 | 1.19892318 | 66.47717687 |
| GOTERM_CC_DIRECT | GO:0030669~clathrin-coated endocytic vesicle membrane | WNT5A, AP1S2, LDLR, ROR2, HLA-DPA1, CD74, HLA-DRA | 2.355347944 | 66.80230277 |
| GOTERM_CC_DIRECT | GO:0005791~rough endoplasmic reticulum | TMEM97, CCL2, ADCYAP1R1, SNCA, TP63, PCSK9, ZC3H12A, LIN28A | 2.164017159 | 66.95028226 |
| GOTERM_CC_DIRECT | GO:0031089~platelet dense granule lumen | RARRES2, CLEC3B, FAM3C, TIMP3 | 3.941602682 | 68.13729417 |
| GOTERM_CC_DIRECT | GO:0042734~presynaptic membrane | SYT1, SNCAIP, NRXN2, GRIK2, SYT7, RIMS2, ADORA1, CAMK2A, IL31RA | 2.002588459 | 69.14080234 |
| GOTERM_CC_DIRECT | GO:0016528~sarcoplasm | FABP3, FLNC, PLEC | 5.912404023 | 73.04124206 |
| GOTERM_CC_DIRECT | GO:0043202~lysosomal lumen | GPC4, FMOD, OGN, OMD, BGN, CUBN, GYG2, DCN, CTSS, FUCA1, CD74 | 1.785314156 | 73.22127883 |
| GOTERM_CC_DIRECT | GO:0031410~cytoplasmic vesicle | F11R, NRP1, GBP5, OPRL1, ENPEP, AMOTL2, GCH1, STARD4, MAP1LC3A, FNBP1L, CXCR4, ARRB1, SYBU, AVPR1A, SORT1, IL15RA, TRPV4, ADAMTS1, SCN8A, IGFBP2, MYOF, PRSS12, HTR2A, ARHGDIB | 1.408913299 | 73.23023366 |
| GOTERM_CC_DIRECT | GO:0070382~exocytic vesicle | UNC13D, IGF1, DPYSL3, SYTL2 | 3.67882917 | 74.48338366 |
| GOTERM_CC_DIRECT | GO:0016529~sarcoplasmic reticulum | GSTM2, ATP2A3, MRVI1, THBS1, RASD1, THBS4 | 2.434519304 | 76.93393032 |
| GOTERM_CC_DIRECT | GO:0005884~actin filament | FMN2, ARHGAP6, RAC2, HCK, AMOT, RHOQ, LMOD1, LCP1, TMOD1 | 1.9101613 | 77.05713557 |

1. KEGG_Pathway

| Category | Term | Genes | Fold Enrichment | FDR |
| --- | --- | --- | --- | --- |
| KEGG_PATHWAY | hsa04514:Cell adhesion molecules (CAMs) | CLDN7, CADM1, CLDN6, L1CAM, CDH2, CLDN11, VCAM1, ALCAM, CD4, ICAM1, F11R, PTPRC, NRXN2, PTPRF, HLA-A, NTNG1, NLGN1, HLA-C, HLA-B, ITGA4, PDCD1LG2, HLA-F, NCAM1, NCAM2, ITGA6, CD34, CD58, CNTN1, HLA-DPA1, JAM2, ICOSLG, HLA-DRA | 2.675572334 | 6.86E-04 |
| KEGG_PATHWAY | hsa05164:Influenza A | NXT1, IFIH1, CCL2, OAS3, NFKBIA, RSAD2, TLR3, OAS1, HSPA1A, TLR4, HSPA1B, OAS2, IL33, CCL5, CXCL10, HSPA2, FAS, MX1, PIK3R3, IFNGR2, PRKCA, ICAM1, IL6, FDPS, DDX58, IKBKE, TNFRSF10C, TNFSF10, MAPK13, IRF7, HLA-DPA1, HLA-DRA | 2.183513054 | 0.056304259 |
| KEGG_PATHWAY | hsa04512:ECM-receptor interaction | COL4A4, COL4A3, TNC, ITGA11, ITGB5, ITGA2, ITGA4, CD36, LAMA3, ITGA6, COL6A6, LAMC3, LAMA5, COMP, LAMC1, SV2A, THBS1, COL11A2, THBS4 | 2.592921752 | 0.348371231 |
| KEGG_PATHWAY | hsa04668:TNF signaling pathway | CXCL1, ICAM1, IL6, CCL2, PTGS2, CXCL3, CXCL2, EDN1, NFKBIA, BIRC3, CCL5, JUNB, CXCL10, VCAM1, FOS, TNFRSF1B, MAPK13, RIPK3, FAS, PIK3R3, TNFAIP3 | 2.352168839 | 0.589054031 |
| KEGG_PATHWAY | hsa04510:Focal adhesion | PGF, TNC, ITGA11, ITGB5, MYL9, RAC2, COL6A6, COMP, PIK3R3, COL11A2, SHC3, THBS1, SHC2, THBS4, PRKCA, COL4A4, COL4A3, FLT1, VAV3, ROCK2, MET, IGF1, ITGA2, ITGA4, CAPN2, FLNC, BIRC3, FLNB, LAMA3, ITGA6, LAMA5, LAMC3, LAMC1 | 1.901961766 | 0.601105565 |
| KEGG_PATHWAY | hsa05410:Hypertrophic cardiomyopathy (HCM) | PRKAG3, IL6, CACNG8, CACNG7, PRKAG2, ITGA11, TGFB3, CACNB2, ITGB5, ITGA2, IGF1, ITGA4, CACNB4, ACE, DES, ITGA6, SGCD | 2.587672923 | 0.832673504 |
| KEGG_PATHWAY | hsa04360:Axon guidance | ABLIM1, NRP1, EFNB3, ROCK2, GNAI1, PLXNB3, MET, NTNG1, L1CAM, SLIT2, EPHB6, RAC2, UNC5B, SEMA4G, CXCR4, RGS3, SEMA3G, CFL2, SEMA7A, SEMA3C, ROBO2, SEMA4D, NFATC2 | 2.150201586 | 1.06053914 |
| KEGG_PATHWAY | hsa00100:Steroid biosynthesis | MSMO1, SQLE, CYP51A1, DHCR7, LSS, HSD17B7, FDFT1, DHCR24 | 4.749140893 | 1.104996224 |
| KEGG_PATHWAY | hsa05162:Measles | IL2RB, IL6, IFIH1, OAS3, NFKBIA, OAS1, HSPA1A, TLR4, HSPA1B, OAS2, DDX58, IKBKE, TNFRSF10C, TNFSF10, HSPA2, CD46, IRF7, FAS, JAK3, PIK3R3, MX1, TNFAIP3, IFNGR2 | 2.05320001 | 1.980691994 |
| KEGG_PATHWAY | hsa00900:Terpenoid backbone biosynthesis | MVD, HMGCR, FDPS, HMGCS1, MVK, PCYOX1, ACAT2, IDI1 | 4.317400812 | 2.08597032 |
| KEGG_PATHWAY | hsa05144:Malaria | VCAM1, CSF3, ICAM1, IL6, CD36, CCL2, COMP, MET, TGFB3, TLR4, THBS1, THBS4 | 2.907637282 | 2.674811373 |
| KEGG_PATHWAY | hsa04974:Protein digestion and absorption | COL4A4, COL4A3, ATP1B1, SLC8A1, COL21A1, SLC9A3, ELN, SLC3A2, COL15A1, KCNN4, COL9A2, COL9A3, COL14A1, COL6A6, COL12A1, COL11A2, COL10A1 | 2.293619182 | 3.15277772 |
| KEGG_PATHWAY | hsa01130:Biosynthesis of antibiotics | HSD17B10, MVD, CYP51A1, HMGCR, HMGCS1, HK2, PGAM2, LSS, ACAT2, FDFT1, ACSS1, ISYNA1, ARG2, PGAM4, IDH1, HSD17B7, SHMT1, SHMT2, MSMO1, FDPS, PCK2, PGM2, PYCR1, GCK, SQLE, HAO2, PHGDH, MVK, PCYOX1, PSAT1, IDI1 | 1.736124619 | 3.968471962 |
| KEGG_PATHWAY | hsa00260:Glycine, serine and threonine metabolism | CHDH, SHMT1, SHMT2, PGAM4, MAOA, MAOB, PHGDH, PGAM2, PSAT1, SARDH | 3.044321086 | 5.438547898 |
| KEGG_PATHWAY | hsa05168:Herpes simplex infection | IFIH1, CCL2, C3, OAS3, TLR3, NFKBIA, OAS1, OAS2, CCL5, CD74, POLR2A, FOS, TAP1, FAS, IFNGR2, CDK1, IL6, HLA-A, HLA-C, HLA-B, HLA-F, DDX58, IKBKE, IFIT1, IRF7, HLA-DPA1, HLA-DRA | 1.751732297 | 6.825717368 |
| KEGG_PATHWAY | hsa05412:Arrhythmogenic right ventricular cardiomyopathy (ARVC) | CACNG8, CACNG7, ITGA11, CACNB2, ITGA2, GJA1, ITGB5, ITGA4, CACNB4, CDH2, DES, ITGA6, SGCD, DSP | 2.341125793 | 7.190441839 |
| KEGG_PATHWAY | hsa05323:Rheumatoid arthritis | ICAM1, IL6, FLT1, CCL2, TGFB3, ACP5, TLR4, CXCL6, CCL5, MMP1, IL11, FOS, TNFSF13B, ANGPT1, HLA-DPA1, HLA-DRA | 2.158700406 | 7.801554707 |
| KEGG_PATHWAY | hsa04066:HIF-1 signaling pathway | PRKCA, IL6, FLT1, EDN1, HK2, EGLN3, IGF1, TLR4, TIMP1, EIF4EBP1, SLC2A1, SERPINE1, ANGPT1, PIK3R3, CAMK2A, IFNGR2, ANGPT2 | 2.059576408 | 9.149315648 |
| KEGG_PATHWAY | hsa05200:Pathways in cancer | WNT5A, ADCY4, PTGS2, GNAI1, PGF, PPARG, ARNT2, LPAR4, TGFB3, FGF11, EGLN3, NFKBIA, FOXO1, BDKRB1, ZBTB16, MMP2, MMP1, FOS, EDNRB, RAC2, CDKN2B, CXCR4, SLC2A1, FAS, PIK3R3, GNG7, PRKCA, CEBPA, COL4A4, COL4A3, IL6, PTGER2, PTGER3, ROCK2, MET, ITGA2, IGF1, BIRC5, BIRC3, MECOM, FZD6, LAMA3, ITGA6, LAMA5, LAMC3, PTCH1, LAMC1, PTCH2 | 1.450119357 | 10.08071388 |
| KEGG_PATHWAY | hsa05145:Toxoplasmosis | LDLR, GNAI1, TGFB3, NFKBIA, HSPA1A, TLR4, HSPA1B, BIRC3, LAMA3, ITGA6, HSPA2, LAMC3, MAPK13, LAMA5, HLA-DPA1, LAMC1, PIK3R3, IFNGR2, HLA-DRA | 1.911730444 | 11.57893672 |
| KEGG_PATHWAY | hsa04670:Leukocyte transendothelial migration | PRKCA, ICAM1, CLDN7, F11R, VAV3, ROCK2, GNAI1, CLDN6, ITGA4, CLDN11, MMP2, MYL9, VCAM1, RAC2, CXCR4, MAPK13, RAPGEF3, JAM2, PIK3R3 | 1.911730444 | 11.57893672 |
| KEGG_PATHWAY | hsa04612:Antigen processing and presentation | HLA-A, HSPA1A, HLA-C, HSPA1B, CTSS, HLA-B, CD74, HLA-F, HSPA2, PSME2, TAP1, HLA-DPA1, CD4, HLA-DRA | 2.187104359 | 12.50267199 |
| KEGG_PATHWAY | hsa04610:Complement and coagulation cascades | F10, MASP1, MASP2, CFB, C3, F13A1, BDKRB1, F3, CD46, SERPINE1, CFH, C2, CFD | 2.236914189 | 14.23106631 |
| KEGG_PATHWAY | hsa00460:Cyanoamino acid metabolism | GGT5, SHMT1, SHMT2, GGT1 | 6.784486991 | 19.20259021 |
| KEGG_PATHWAY | hsa04060:Cytokine-cytokine receptor interaction | CSF3, CCL2, TNFRSF12A, LEPR, CTF1, GDF5, TGFB3, CCL8, CCL5, CCL7, IL11, CXCL10, IL17RB, TNFRSF1B, CXCR4, IL15RA, FAS, IFNGR2, IL6, IL2RB, FLT1, IL7, MET, LIFR, ACVR2A, TNFSF10, ACVR2B, TNFSF13B, IL20RB, RELT | 1.5486329 | 20.53126019 |
| KEGG_PATHWAY | hsa05332:Graft-versus-host disease | IL6, HLA-A, HLA-C, HLA-DPA1, HLA-B, FAS, HLA-DRA, HLA-F | 2.878267208 | 21.11632434 |
| KEGG_PATHWAY | hsa04913:Ovarian steroidogenesis | AKR1C3, ADCY4, LDLR, PTGS2, STAR, IGF1, SCARB1, PLA2G4C, HSD17B7, BMP6 | 2.423031068 | 22.73023626 |
| KEGG_PATHWAY | hsa05414:Dilated cardiomyopathy | ADCY4, CACNG8, CACNG7, TGFB3, ITGA11, CACNB2, ITGA2, IGF1, ITGB5, ITGA4, CACNB4, DES, ITGA6, SGCD | 1.978808706 | 25.74207256 |
| KEGG_PATHWAY | hsa04062:Chemokine signaling pathway | CXCL1, ADCY4, CCL2, VAV3, GNAI1, ROCK2, HCK, CXCL3, CXCL2, NFKBIA, CCL8, CXCL6, CCL5, CCL7, CXCL10, DOCK2, RAC2, CXCR4, ARRB1, JAK3, GRK5, PIK3R3, SHC3, SHC2, GNG7 | 1.595813472 | 25.76260529 |
| KEGG_PATHWAY | hsa00480:Glutathione metabolism | GSTM2, GGT5, GSTM3, RRM2, GPX3, IDH1, GGT1, GPX7, GSTM5, MGST2 | 2.328010242 | 28.09946166 |
| KEGG_PATHWAY | hsa05134:Legionellosis | CXCL1, IL6, HSPA2, C3, CXCL3, CXCL2, NFKBIA, TLR4, HSPA1A, HSPA1B | 2.19867634 | 37.11178718 |
| KEGG_PATHWAY | hsa05150:Staphylococcus aureus infection | ICAM1, MASP1, C3, CFB, MASP2, CFH, HLA-DPA1, C2, CFD, HLA-DRA | 2.19867634 | 37.11178718 |
| KEGG_PATHWAY | hsa04978:Mineral absorption | ATP1B1, MT1M, SLC9A3, MT2A, MT1E, HEPH, SLC40A1, MT1X, MT1F | 2.32294935 | 38.31913375 |
| KEGG_PATHWAY | hsa04921:Oxytocin signaling pathway | PRKCA, PRKAG3, ADCY4, PTGS2, ROCK2, GNAI1, CACNG8, CACNG7, PRKAG2, CACNB2, NPR1, CACNB4, MYL9, FOS, RGS2, GUCY1A3, PIK3R3, PLA2G4C, NFATC2, CAMK2A, CAMK1D | 1.578037322 | 42.90075262 |
| KEGG_PATHWAY | hsa04024:cAMP signaling pathway | ADCY4, ATP1B1, GNAI1, ADCYAP1R1, NFKBIA, GRIN3B, ADORA1, MYL9, FOS, BDNF, ATP2B4, RAC2, GRIN2D, RAPGEF3, PIK3R3, CAMK2A, HCN2, PTGER2, VAV3, PTGER3, ROCK2, NPR1, GRIA4, CHRM2, PTCH1 | 1.499097504 | 43.83735683 |
| KEGG_PATHWAY | hsa05132:Salmonella infection | CXCL1, IL6, ROCK2, CXCL3, CXCL2, TLR4, FLNC, FLNB, FOS, PKN3, MAPK13, DYNC2H1, IFNGR2 | 1.859603362 | 44.5732636 |
| KEGG_PATHWAY | hsa00072:Synthesis and degradation of ketone bodies | HMGCS1, OXCT2, ACAT2, BDH1 | 4.749140893 | 45.86930065 |
| KEGG_PATHWAY | hsa00380:Tryptophan metabolism | KYNU, TDO2, MAOA, AOX1, MAOB, IL4I1, ACAT2, INMT | 2.374570447 | 46.90465112 |
| KEGG_PATHWAY | hsa04350:TGF-beta signaling pathway | SMAD9, GDF5, TGFB3, DCN, INHBB, INHBA, ACVR2A, ACVR2B, CDKN2B, ID4, THBS1, PITX2, BMP6 | 1.837465227 | 47.24390001 |
| KEGG_PATHWAY | hsa04151:PI3K-Akt signaling pathway | CSF3, PGF, TNC, LPAR4, ITGA11, FGF11, ITGB5, TLR4, EIF4EBP1, COL6A6, PKN3, COMP, ANGPT1, COL11A2, THBS1, PIK3R3, ANGPT2, THBS4, GNG7, PRKCA, COL4A4, COL4A3, IL6, IL2RB, FLT1, IL7, MET, IGF1, ITGA2, ITGA4, PCK2, DDIT4, LAMA3, ITGA6, LAMA5, LAMC3, CHRM2, JAK3, LAMC1 | 1.342148513 | 49.38813871 |
| KEGG_PATHWAY | hsa05222:Small cell lung cancer | COL4A4, COL4A3, PTGS2, ITGA2, NFKBIA, BIRC3, LAMA3, ITGA6, CDKN2B, LAMA5, LAMC3, LAMC1, PIK3R3 | 1.815847989 | 49.93153021 |
| KEGG_PATHWAY | hsa03320:PPAR signaling pathway | LPL, CD36, SCD, PPARG, FABP3, FADS2, AQP7, PCK2, ACSL3, FABP5, MMP1 | 1.949274247 | 50.5544737 |
| KEGG_PATHWAY | hsa05142:Chagas disease (American trypanosomiasis) | IL6, CCL2, C3, GNAI1, TGFB3, NFKBIA, TLR4, CCL5, FOS, ACE, MAPK13, SERPINE1, FAS, PIK3R3, IFNGR2 | 1.712430611 | 50.70660149 |
| KEGG_PATHWAY | hsa04145:Phagosome | TUBB2B, C3, HLA-A, ITGB5, ITGA2, HLA-C, TLR4, COLEC12, HLA-B, CTSS, HLA-F, CD36, COMP, TAP1, DYNC2H1, SCARB1, HLA-DPA1, THBS1, THBS4, HLA-DRA | 1.552006828 | 52.12301521 |
| KEGG_PATHWAY | hsa04940:Type I diabetes mellitus | CPE, HLA-A, HLA-C, HLA-DPA1, HLA-B, FAS, HLA-DRA, HLA-F | 2.261495664 | 55.00325033 |
| KEGG_PATHWAY | hsa05166:HTLV-I infection | WNT5A, ADCY4, NRP1, TGFB3, NFKBIA, VCAM1, FOS, CDKN2B, XBP1, SLC2A1, IL15RA, PIK3R3, NFATC2, EGR1, ICAM1, IL2RB, IL6, EGR2, RELB, HLA-A, FDPS, HLA-C, CDC20, HLA-B, FZD6, HLA-F, MSX1, HLA-DPA1, JAK3, HLA-DRA | 1.391349871 | 55.3090386 |
| KEGG_PATHWAY | hsa04976:Bile secretion | ADCY4, ATP1B1, AQP9, LDLR, HMGCR, SLC9A3, SLC2A1, SCARB1, SLC4A4, AQP1, ABCB4 | 1.892773544 | 56.67754781 |
| KEGG_PATHWAY | hsa05140:Leishmaniasis | FOS, PTGS2, C3, MAPK13, TGFB3, NFKBIA, TLR4, HLA-DPA1, ITGA4, IFNGR2, HLA-DRA | 1.83945598 | 62.65347907 |
| KEGG_PATHWAY | hsa00750:Vitamin B6 metabolism | AOX1, PDXP, PSAT1 | 5.936426117 | 68.78974501 |
| KEGG_PATHWAY | hsa04623:Cytosolic DNA-sensing pathway | DDX58, POLR3G, IKBKE, IL6, IRF7, RIPK3, NFKBIA, IL33, CCL5, CXCL10 | 1.855133162 | 69.34625891 |
| KEGG_PATHWAY | hsa05330:Allograft rejection | HLA-A, HLA-C, HLA-DPA1, HLA-B, FAS, HLA-DRA, HLA-F | 2.246215287 | 69.47467872 |
| KEGG_PATHWAY | hsa05160:Hepatitis C | CLDN7, LDLR, CLDN6, OAS3, TLR3, NFKBIA, OAS1, OAS2, CLDN11, DDX58, IKBKE, IFIT1, MAPK13, IRF7, IRF1, SCARB1, PIK3R3 | 1.517582616 | 71.68194727 |
| KEGG_PATHWAY | hsa00760:Nicotinate and nicotinamide metabolism | NAMPT, NMNAT2, ENPP1, BST1, AOX1, QPRT | 2.456452186 | 71.79040696 |
| KEGG_PATHWAY | hsa04672:Intestinal immune network for IgA production | IL6, TNFSF13B, CXCR4, IL15RA, HLA-DPA1, ITGA4, ICOSLG, HLA-DRA | 2.020911018 | 73.54976145 |
| KEGG_PATHWAY | hsa04640:Hematopoietic cell lineage | CSF3, IL6, CD36, ITGA6, CD34, IL7, ITGA2, CD4, ITGA4, CD24, HLA-DRA, IL11 | 1.676167374 | 74.57283294 |

**Supplementary Table S3.** List of prokaryotic and eukaryotic cells used in this study.

| Strain name | | Remarks | rRNA sequence accession numbers |
| --- | --- | --- | --- |
| Prokaryote, gram positive. | | |  |
| *Lactobacillus acidophilus* JCM 1021 | Lactic acid bacteria, isolated from mouse intestine. | | AB289011^1a^ |
| *Lactobacillus reuteri* JCM 1112 | Lactic acid bacteria, isolated from cheese. | | L23507^1a^ |
| *Lactobacillus casei* JCM1134 | Lactic acid bacteria, isolated from wine. | | D16551.1^1a^ |
| *Bacillus subtilis* subsp. Subtilis 168 JCM10629 | Fundamental analysis model of gram-positive bacterium. | | AB598744^1a^ |
| *Staphylococcus epidermidis* JCM2414 | Grape-like clustering was commonly observed. | | D83363^1a^ |
| Prokaryote, gram negative. | | |  |
| *Pseudomonas putida* JCM 13063 | This strain is not pathogenic but an opportunistic human pathogen, *P. aeruginosa*, is closely related. | | AF094736.1^1a^ |
| *Mesorhizobium loti* JCM 21590 | Nitrogen fixative plant-microbe symbiont. | | mlo:rrn16Sa^1b^ |
| *Escherichia coli* JE28 | Derivatives of *E. coli* MG1655 genetically modified as Tetra-(His)_6_-tagged in ribosome L12 proteins. | | eco:b3851^1b^ |
| Eukaryote | | |  |
| Saccharomyces cerevisiae S288C (BY20118) | Budding yeast. | | sce:RDN18-1^1b^ |
| *Rattus norvegicus* IEC-6. | Rat small intestine/epithelium. | | rno:100861533^1b^ |
| *Homo sapience*. Human dermal fibroblast. | Normal human adult skin from the facial dermis of a 34-year-old Caucasian female (Cell applications). | | hsa:100008588^1b^ |

^1^ 16S or 18S rRNA Sequences were obtained from (a) NCBI or (b) KEGG.

**Supplementary Table S4.** List of endocytosis inhibitors used in this study.

| Name | Remarks |
| --- | --- |
| Filipin (FP) | Inhibitor of caveolae-dependent endocytosis without functionally disrupting the coated vesicular degradative pathway ^1^. |
| Nystatin (NS) | Inhibitor of caveolae-mediated endocytosis by binding specifically to sterol ^2^. |
| Genistein (GS) | Inhibitor of trafficking to the late endosome / lysosome ^3^. |
| Cytocharasin B (CC) | Sugar transport inhibitor ^4^. |
| 5-(N-Ethyl-N-isopropyl)-amiloride (5NA), | Macropinocytosis inhibitor ^5^. |
| Bafiolomycin A1 (BM) | Inhibitor of vacuolar H (+)-ATPases ^6^. |
| Concanamycin A (CM) | Different type of Inhibitor of vacuolar H (+)-ATPases compared with BM ^7,8^. |
| Chlorpromazine (CP) | Clathrin type endocytosis inhibitor, caused the loss of coated pits ^9^. |
| Methyl β-Cyclodextrin (MβCD) | Inhibitor of clathrin-coated Endocytotic by eliminating the sterol of the plasma membrane ^10^. MβCD also works uptake facilitater by creating a inclusion complex ^11^. |

1 Rothberg, K. G., Ying, Y. S., Kamen, B. A. & Anderson, R. G. Cholesterol controls the clustering of the glycophospholipid-anchored membrane receptor for 5-methyltetrahydrofolate. *J Cell Biol* **111**, 2931-2938 (1990).

2 Rothberg, K. G. *et al.* Caveolin, a protein component of caveolae membrane coats. *Cell* **68**, 673-682 (1992).

3 Mundy, D. I., Li, W. P., Luby-Phelps, K. & Anderson, R. G. Caveolin targeting to late endosome/lysosomal membranes is induced by perturbations of lysosomal pH and cholesterol content. *Mol Biol Cell* **23**, 864-880 (2012).

4 Rampal, A. L., Pinkofsky, H. B. & Jung, C. Y. Structure of cytochalasins and cytochalasin B binding sites in human erythrocyte membranes. *Biochemistry* **19**, 679-683 (1980).

5 Koivusalo, M. *et al.* Amiloride inhibits macropinocytosis by lowering submembranous pH and preventing Rac1 and Cdc42 signaling. *J Cell Biol* **188**, 547-563 (2010).

6 Xu, J. *et al.* Effects of Bafilomycin A1: an inhibitor of vacuolar H (+)-ATPases on endocytosis and apoptosis in RAW cells and RAW cell-derived osteoclasts. *J Cell Biochem* **88**, 1256-1264 (2003).

7 Bowman, E. J., Graham, L. A., Stevens, T. H. & Bowman, B. J. The bafilomycin/concanamycin binding site in subunit c of the V-ATPases from Neurospora crassa and Saccharomyces cerevisiae. *J Biol Chem* **279**, 33131-33138 (2004).

8 Huss, M. *et al.* Concanamycin A, the specific inhibitor of V-ATPases, binds to the V(o) subunit c. *J Biol Chem* **277**, 40544-40548 (2002).

9 Wang, L. H., Rothberg, K. G. & Anderson, R. G. Mis-assembly of clathrin lattices on endosomes reveals a regulatory switch for coated pit formation. *J Cell Biol* **123**, 1107-1117 (1993).

10 Rodal, S. K. *et al.* Extraction of cholesterol with methyl-beta-cyclodextrin perturbs formation of clathrin-coated endocytic vesicles. *Mol Biol Cell* **10**, 961-974 (1999).

11 Mathiron, D. *et al.* Benefits of methylated cyclodextrins in the development of midazolam pharmaceutical formulations. *J Pharm Sci* **102**, 2102-2111 (2013).

**Supplementary Table S5: Primers for bisulfite PCR used in this study.**

| Gene | Primer pair | Forward primer (F) | Annealing temp. (C) |
| --- | --- | --- | --- |
|  |  | Reverse primer (R) |  |
| OCT4 | Oct4-1 | F: TTTTTAGTTTTTTTTAGGTTTAA^a^ | 50 |
|  |  | R: TAAACAAAAAACCCATTCCC^a^ |  |
|  | Oct4-2 | F: TTAGGAAAATGGGTAGTAGGGATTT^a^ | 57 |
|  |  | R: TACCCAAAAAACAAATAAATTATAAAACCT^a^ |  |
|  | Oct4-3 | F: ATTTGTTTTTTGGGTAGTTAAAGGT^a^ | 55 |
|  |  | R: CCAACTATCTTCATCTTAATAACATCC^a^ |  |
|  | Oct4-4 | F: GGATGTTATTAAGATGAAGATAGTTGG^a^ | 55 |
|  |  | R: CCTAAACTCCCCTTCAAAATCTATT^a^ |  |
|  | Oct4-5 | F: AATAGATTTTGAAGGGGAGTTTAGG^a^ | 57 |
|  |  | R: TTCCTCCTTCCTCTAAAAAACTCA^a^ |  |
|  | Oct4-6 | F: GAAGGGGAAGTAGGGATTAATTTT^a^ | 55 |
|  |  | R: CAACAACCATAAACACAATAACCAA^a^ |  |
|  | Oct4-7 | F: TAGTTGGGATGTGTAGAGTTTGAGA^a^ | 55 |
|  |  | R: TAAACCAAAACAATCCTTCTACTCC^a^ |  |
|  | Oct4-8 | F: AAGTTTTTGTGGGGGATTTGTAT^a^ | 55 |
|  |  | R: CCACCCACTAACCTTAACCTCTA^a^ |  |
|  | Oct4-9 | F: GTTAGAGGTTAAGGTTAGTGGGTG^a^ | 57 |
|  |  | R: AAACCTTAAAAACTTAACCAAATCC^a^ |  |
| NANOG | Nanog-1 | F: AGAGATAGGAGGGTAAGTTTTTTTT^b^ | 55 |
|  |  | R: ACTCCCACACAAACTAACTTTTATTC^b^ |  |
|  | Nanog-2 | F: GAGTTAAAGAGTTTTGTTTTTAAAAATTAT^b^ | 55 |
|  |  | R: TCCCAAATCTAATAATTTATCATATCTTTC^b^ |  |
|  | Nanog-3 | F: TTAATTTATTGGGATTATAGGGGTG^a^ | 57 |
|  |  | R: AACAACAAAACCTAAAAACAAACC^b^ |  |
| LMNA | Lmna | F: GAAGGGTGAGTTATATTGATGGGTAT^b^ | 55 |
|  |  | R: ACTCTTAAAAAAACAATCCCAAATC^b^ |  |

Primers were sited as described (a) ^1^ and (b) ^2^.

1 Deb-Rinker, P., Ly, D., Jezierski, A., Sikorska, M. & Walker, P. R. Sequential DNA methylation of the Nanog and Oct-4 upstream regions in human NT2 cells during neuronal differentiation. *J Biol Chem* **280**, 6257-6260 (2005).

2 Freberg, C. T., Dahl, J. A., Timoskainen, S. & Collas, P. Epigenetic reprogramming of OCT4 and NANOG regulatory regions by embryonal carcinoma cell extract. *Mol Biol Cell* **18**, 1543-1553 (2007).

**Supplementary Video S1.** Three-dimensional imaging of cell clusters induced by His-ribosomes. The clusters were stained with Alexa Fluor 488 phalloidin (green) and Hoechst 33342 (blue). Images were captured using FV1200 Olympus confocal microscope software. His-tagged ribosomes were detected by anti-His tag antibody (abcam, ab18184; 1:200).

**Supplementary Video S2.** RICs generated from HDFs. The video begins immediately after the mixing of ribosomes and HDFs. (a) HDF cell cluster formation induced by ribosome treatment. (b) Negative control cultures of HDFs for Video S2A; HDFs were not treated with ribosomes (viz., normal culture).

**Supplementary Video S3.** RIC formation from Fucci2-carrying MEFs. The video begins immediately after the mixing of ribosomes and MEFs. (a) Ribosome-treatment-induced formation of cell clusters in Fucci2-carrying MEFs. (b) Negative control cultures of MEFs for Video S3A; MEFs were not treated with ribosomes (viz., normal culture).

**Supplementary Video S4.** Three-dimensional modeling of RICs based on ultrahigh-resolution observation. RICs induced by treatment with His-ribosomes are indicated in red.
